# Raltegravir-intensified initial antiretroviral therapy in advanced HIV disease in Africa: A randomised controlled trial

**DOI:** 10.1371/journal.pmed.1002706

**Published:** 2018-12-04

**Authors:** Cissy Kityo, Alexander J. Szubert, Abraham Siika, Robert Heyderman, Mutsa Bwakura-Dangarembizi, Abbas Lugemwa, Shalton Mwaringa, Anna Griffiths, Immaculate Nkanya, Sheila Kabahenda, Simon Wachira, Godfrey Musoro, Chatu Rajapakse, Timothy Etyang, James Abach, Moira J. Spyer, Priscilla Wavamunno, Linda Nyondo-Mipando, Ennie Chidziva, Kusum Nathoo, Nigel Klein, James Hakim, Diana M. Gibb, A. Sarah Walker, Sarah L. Pett, on behalf of the REALITY trial team

**Affiliations:** 1 Joint Clinical Research Centre, Kampala, Uganda; 2 Medical Research Council Clinical Trials Unit at University College London, University College London, London, United Kingdom; 3 Moi University School of Medicine, Eldoret, Kenya; 4 Department/College of Medicine, University of Malawi, and Malawi-Liverpool-Wellcome Trust Clinical Research Programme, Blantyre, Malawi; 5 Division of Infection and Immunity, University College London, London, United Kingdom; 6 University of Zimbabwe Clinical Research Centre, Harare, Zimbabwe; 7 Joint Clinical Research Centre, Mbarara, Uganda; 8 KEMRI Wellcome Trust Research Programme, Kilifi, Kenya; 9 Joint Clinical Research Centre, Fort Portal, Uganda; 10 Joint Clinical Research Centre, Gulu, Uganda; 11 University College London Great Ormond Street Institute of Child Health, University College London, London, United Kingdom; 12 Institute for Global Health, University College London, London, United Kingdom; 13 Kirby Institute for Infection and Immunity in Society, University of New South Wales, Sydney, Australia; Boston University, UNITED STATES

## Abstract

**Background:**

In sub-Saharan Africa, individuals infected with HIV who are severely immunocompromised have high mortality (about 10%) shortly after starting antiretroviral therapy (ART). This group also has the greatest risk of morbidity and mortality associated with immune reconstitution inflammatory syndrome (IRIS), a paradoxical response to successful ART. Integrase inhibitors lead to significantly more rapid declines in HIV viral load (VL) than all other ART classes. We hypothesised that intensifying standard triple-drug ART with the integrase inhibitor, raltegravir, would reduce HIV VL faster and hence reduce early mortality, although this strategy could also risk more IRIS events.

**Methods and findings:**

In a 2×2×2 factorial open-label parallel-group trial, treatment-naive adults, adolescents, and children >5 years old infected with HIV, with cluster of differentiation 4 (CD4) <100 cells/mm^3^, from eight urban/peri-urban HIV clinics at regional hospitals in Kenya, Malawi, Uganda, and Zimbabwe were randomised 1:1 to initiate standard triple-drug ART, with or without 12-week raltegravir intensification, and followed for 48 weeks. The primary outcome was 24-week mortality, analysed by intention to treat. Of 2,356 individuals screened for eligibility, 1,805 were randomised between 18 June 2013 and 10 April 2015. Of the 1,805 participants, 961 (53.2%) were male, 72 (4.0%) were children/adolescents, median age was 36 years, CD4 count was 37 cells/mm^3^, and plasma viraemia was 249,770 copies/mL. Fifty-six participants (3.1%) were lost to follow-up at 48 weeks. By 24 weeks, 97/902 (10.9%) raltegravir-intensified ART versus 91/903 (10.2%) standard ART participants had died (adjusted hazard ratio [aHR] = 1.10 [95% CI 0.82–1.46], *p* = 0.53), with no evidence of interaction with other randomisations (*p*_heterogeneity_ > 0.7) and despite significantly greater VL suppression with raltegravir-intensified ART at 4 weeks (343/836 [41.0%] versus 113/841 [13.4%] with standard ART, *p* < 0.001) and 12 weeks (567/789 [71.9%] versus 415/803 [51.7%] with standard ART, *p* < 0.001). Through 48 weeks, there was no evidence of differences in mortality (aHR = 0.98 [95% CI 0.76–1.28], *p* = 0.91); in serious (aHR = 0.99 [0.81–1.21], *p* = 0.88), grade-4 (aHR = 0.88 [0.71–1.09], *p* = 0.29), or ART-modifying (aHR = 0.90 [0.63–1.27], *p* = 0.54) adverse events (the latter occurring in 59 [6.5%] participants with raltegravir-intensified ART versus 66 [7.3%] with standard ART); in events judged compatible with IRIS (occurring in 89 [9.9%] participants with raltegravir-intensified ART versus 86 [9.5%] with standard ART, *p* = 0.79) or in hospitalisations (aHR = 0.94 [95% CI 0.76–1.17], *p* = 0.59). At 12 weeks, one and two raltegravir-intensified participants had predicted intermediate-level and high-level raltegravir resistance, respectively. At 48 weeks, the nucleoside reverse transcriptase inhibitor (NRTI) mutation K219E/Q (*p* = 0.004) and the non-nucleoside reverse transcriptase inhibitor (NNRTI) mutations K101E/P (*p* = 0.03) and P225H (*p* = 0.007) were less common in virus from participants with raltegravir-intensified ART, with weak evidence of less intermediate- or high-level resistance to tenofovir (*p* = 0.06), abacavir (*p* = 0.08), and rilpivirine (*p* = 0.07). Limitations of the study include limited clinical, radiological, and/or microbiological information for some participants, reflecting available services at the centres, and lack of baseline genotypes.

**Conclusions:**

Although 12 weeks of raltegravir intensification was well tolerated and reduced HIV viraemia significantly faster than standard triple-drug ART during the time of greatest risk for early death, this strategy did not reduce mortality or clinical events in this group and is not warranted. There was no excess of IRIS-compatible events, suggesting that integrase inhibitors can be used safely as part of standard triple-drug first-line therapy in severely immunocompromised individuals.

**Trial registration:**

ClinicalTrials.gov NCT01825031.

**Trial registration:**

International Standard Randomised Controlled Trials Number ISRCTN 43622374.

## Abstract 2

In the factorial randomised REALITY trial, Sarah Walker and colleagues document outcomes of intensified initial antiretroviral treatment for people with advanced HIV disease.

## Author summary

**Why was this study done?:**

- Individuals in Africa who are HIV positive and initiating treatment with severe immunosuppression (CD4 < 100 cells/mm^3^) have high risks of dying shortly after starting treatment.
- It would be expected that adding an integrase inhibitor (raltegravir) to standard triple-drug antiretroviral therapy for 12 weeks would reduce HIV viral load faster: we wanted to find out whether this would reduce high early mortality.

**What did the researchers do and find?:**

- We randomly allocated 1,805 adults, adolescents, and older children to receive standard antiretroviral therapy with or without 12 weeks of adjunctive raltegravir.
- We found that the group receiving 12 weeks of adjunctive raltegravir had significantly faster declines in HIV viral load.
- However, we found no significant difference in deaths within 24 weeks of starting treatment between those receiving adjunctive raltegravir (97/902 [10.9%]) or not (91/903 [10.2%]); nor were there differences in clinical disease progression, immune reconstitution, inflammatory syndrome, or adverse events.

**What do these findings mean?:**

- In this study, we found that intensifying the potency of initial antiretroviral therapy did not reduce early mortality on treatment, providing no support for its widespread use in individuals initiating treatment with severe immunosuppression.
- However, it also did not increase the rates of clinically important immune reconstitution inflammatory syndrome, suggesting that integrase inhibitors could replace other components of standard first-line antiretroviral therapy safely.

## Introduction

Despite World Health Organisation (WHO) guidelines recommending universal antiretroviral therapy (ART) regardless of cluster of differentiation 4 (CD4) cell count [1], 20%–25% of individuals infected with HIV in sub-Saharan Africa continue to present for care with severe immunosuppression (CD4 count < 100 cells/mm^3^) [2]. Of these, about 10% die in the first 3 months after ART initiation [3–6]. Causes of early death are multifactorial and are similar for adults and older children [5]. Possible strategies to reduce this excess mortality could aim to accelerate immune restoration by controlling HIV viraemia more rapidly; to control or prevent overt clinical infections; or to provide nutritional support to enhance immune response to infection and reverse metabolic deficiencies.

Standard WHO-recommended ART [1] consists of two nucleos(t)ide reverse transcriptase inhibitors (N(t)RTI), with a non-nucleoside reverse transcriptase inhibitor (NNRTI). Another key drug class is integrase strand-transfer inhibitors (INSTIs), which result in faster decline in HIV viral load (VL) over the first 3 months of combination therapy [7,8]. Several studies have investigated how this might impact chronic inflammation and immune restoration; in some, INSTIs appeared to improve markers of inflammation (including microbial translocation) and/or T-cell activation [9–14], but in others there were no clear differences in inflammatory and other markers (i.e., D-dimer, interleukin 6 [IL-6], and T-cell activation) [15]. The clinical importance of intensified or quadruple ART in patients with VL >100,000 copies/mL or advanced disease remains unclear.

To our knowledge, no randomised trial has been powered to test the hypothesis that the INSTI-associated rapid reduction in HIV viraemia translates into clinical benefit, mortality reductions in particular, or, conversely, is associated with harm through increased risk of immune reconstitution inflammatory syndrome (IRIS). We therefore conducted the Reduction of EArly mortaLITY (REALITY) randomised clinical-endpoint trial (NCT01825031; ISRCTN43622374) to compare three interventions to reduce early mortality in adults and older children initiating ART with CD4 <100 cells/mm^3^ in four sub-Saharan African countries: adjunctive intensification with raltegravir [16] (the first licenced INSTI), enhanced anti-infective prophylaxis, and food supplementation. Here, we report on the impact of adjunctive raltegravir intensification.

## Materials and methods

Adults, adolescents, and children aged ≥5 years infected with HIV, diagnosed through national screening programmes, not on ART and reporting no previous ART, and with CD4 <100 cells/mm^3^ were approached consecutively for screening from inpatient and outpatient facilities at clinics at eight urban/peri-urban regional hospitals in Zimbabwe, Uganda, Malawi, and Kenya. When CD4 counts were not routinely performed at diagnosis, those with new HIV diagnoses were approached consecutively for CD4 testing and study screening. Participants were enrolled if they had screening CD4 count <100 cells/mm^3^, were ART-naive, were not pregnant/breastfeeding, had not used single-dose nevirapine to prevent mother-to-child transmission, had no contraindications to trial drugs, and provided written informed consent. The trial was approved by Ethics Committees in Zimbabwe, Uganda, Malawi, Kenya, and the United Kingdom. The protocol and CONSORT checklist are provided as **S1 Protocol** and **S1 CONSORT checklist.**

Participants were randomised 1:1 to initiate open-label ART with 2NRTI+NNRTI either alone (standard ART) or with 12 weeks’ raltegravir (raltegravir intensification) using standard doses (see **S1 Text**). Standard ART was tenofovir-disoproxil-fumarate+emtricitabine or zidovudine+lamivudine in adults and adolescents and abacavir+lamivudine or zidovudine+lamivudine in children aged 5–<13 years, with nevirapine or efavirenz, according to physician choice and local standard of care. Participants were also factorially randomised 1:1 to 12 weeks enhanced anti-infection prophylaxis versus standard-of-care co-trimoxazole prophylaxis [17], and 1:1 to 12 weeks ready-to-use supplementary food (RUSF) versus no routine supplementation [18] (see **S1 Text** for details).

Randomisation was stratified by centre, age (</≥13 years), and the other factorial randomisations. A computer-generated sequential randomisation list using variably sized permuted blocks was prepared by the trial statistician and incorporated securely into the online trial database. The list was concealed until allocation after eligibility was confirmed by local centre staff, who then performed the randomisation.

Nurse visits at weeks 2, 4, 8, 12, 18, 24, 36, and 48 included symptom checklist, self-reported adherence assessment (with standard adherence support), medication dispensing, and body composition assessment using bioimpedance (TANITA BC-420MA). At weeks 4, 12, 24, 36, and 48, history and examination were undertaken by a physician; haematology, biochemistry, and CD4/CD8 assays were performed at local laboratories; and plasma was stored for retrospective VL and genotyping (results not available in real time). Nurses/physicians were unblinded. Laboratory tests were assayed blind to randomisation. Toxicity substitution and/or second-line switch were at physician discretion, following WHO guidelines [19]. Participants exited the trial after 48 weeks. Consent was obtained to ascertain vital status in all participants at trial closure (May 2016, i.e., beyond 48 weeks from randomisation) from the ART programmes where they had transferred to care at trial exit.

The primary outcome was all-cause mortality to 24 weeks. Secondary outcomes to 48 weeks were all-cause mortality; serious adverse events (SAEs) following International Committee on Harmonization definitions, grade-4 adverse events (AEs), and AEs leading to modification of ART/trial drugs; specific mechanisms of each intervention (CD4 count change; incidence of tuberculosis, cryptococcosis, candidiasis, severe bacterial infections); changes in weight/BMI; hospitalisations; and self-reported ART adherence/acceptability. Other prespecified outcomes included VL suppression (originally planned to be assayed only in a random subset; ultimately assayed in all participants), genotypic resistance (assayed in samples from Kenya, Uganda, and Zimbabwe, see **S1 Text**), and body composition. All AEs were graded following established guidelines [20,21]. HIV integrase genotypes were assayed for raltegravir-intensified participants with VL >1,000 c/mL at 12 weeks, and reverse transcriptase genotypes were assayed for all participants with VL >1,000 c/mL at 48 weeks. An Endpoint Review Committee (ERC) (majority independent members), blind to trial drugs received, adjudicated secondary clinical endpoints and trial-drug relatedness against protocol-defined criteria and assessed compatibility of clinical events with IRIS. Clinical endpoints were predominantly clinically defined, as microbiological and other diagnostic facilities were limited in most centres. Because of this known limitation, IRIS was not a secondary outcome and was considered an exploratory analysis. However, IRIS was part of the standardised ERC assessment and ERC case record form from the start of the trial and was prospectively ascertained on all reviewed events. Events were considered IRIS compatible if there was atypical or exaggerated presentation of an opportunistic infection or tumour soon after ART initiation. VLs were only measured retrospectively on stored plasma and so were not available contemporaneously for assessment of IRIS compatibility; the earliest post-randomisation CD4 count was done at 4 weeks and so was not available for events before this. As access to diagnostic testing was limited, previously published definitions [22,23] were used but in modified form; the ERC adjudication relied heavily on the description of the clinical presentation and any radiology/microbiology/histology provided by site, and the timing of the presentation and its evolution in relation to ART initiation.

A total of 1,800 adults/children provided >80% power to detect 50% relative reductions in 24-week all-cause mortality from 7% to 3.5% (two-sided alpha = 0.05), allowing 5% lost to follow-up. Interim data were reviewed by an independent Data Monitoring Committee (three annual meetings) using the Haybittle-Peto criterion (*p* < 0.001). Randomised groups were compared following the intention to treat principle using log-rank tests for time-to-event outcomes (censoring those lost to follow-up), Fisher exact tests for binary outcomes, and generalized estimating equations (GEEs) with independent working correlation for global tests of repeated measures (logit/normal distributions for binary/continuous outcomes, respectively). Primary analyses were stratified by randomisation stratification factors (details in **S1 Text**). Analyses used Stata v15.1.

## Results

**Fig 1:**
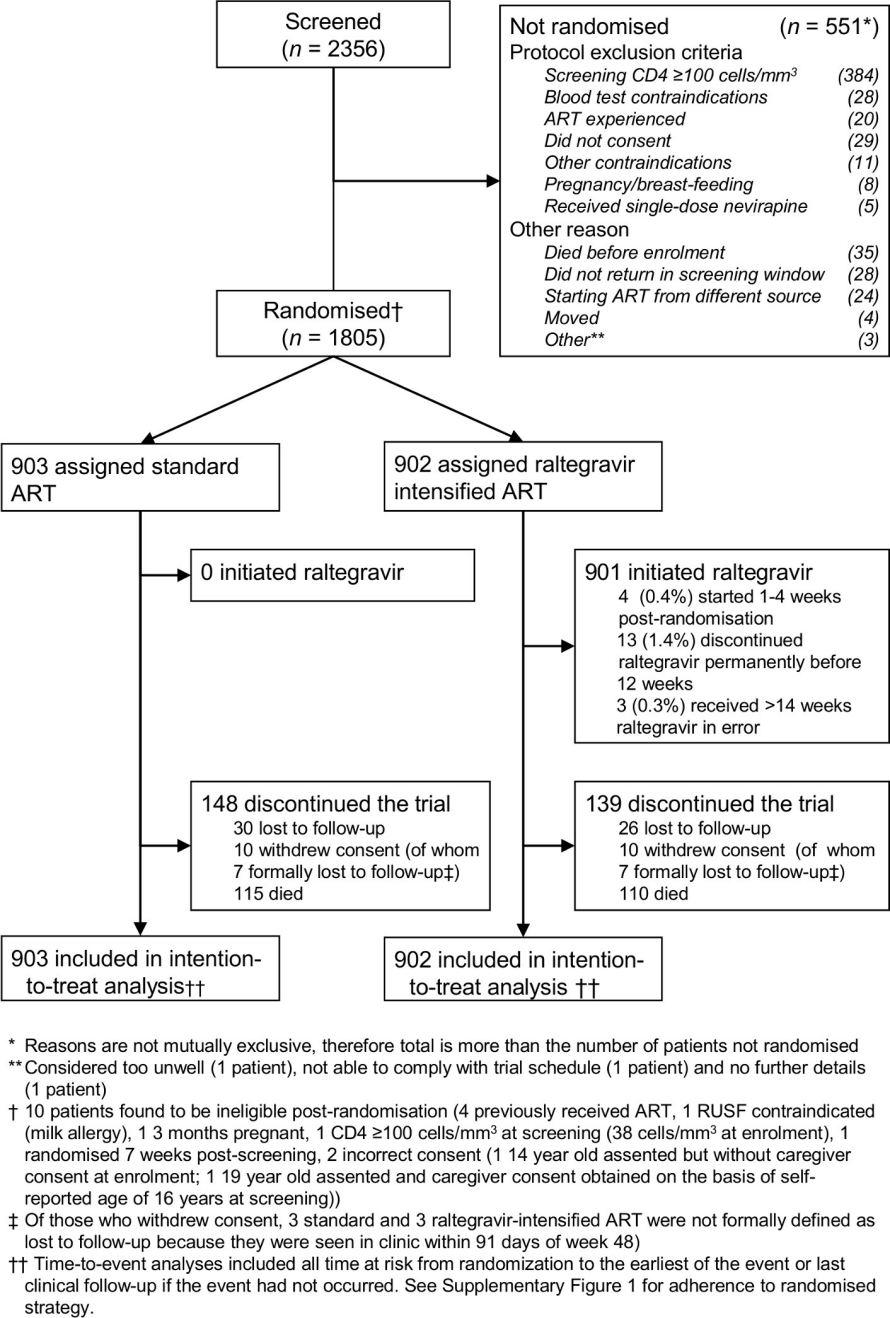
Trial profile. ART, antiretroviral therapy; CD4, cluster of differentiation 4; RUSF, ready-to-use supplementary food.

Between 18 June 2013 and 10 April 2015, 1,805 participants were randomised to standard ART (*n* = 903) or raltegravir-intensified ART (*n* = 902) (**Fig 1**).

Baseline characteristics were well balanced between randomised groups (**Table 1**). Median age was 36 years; 72 (4.0%) participants were aged 5–17 years. Median CD4 count was 37 cells/mm^3^ and VL was 249,770 copies/mL; 1,334/1,804 (73.9%) had VL ≥ 100,000 copies/mL. Despite this, 854 (47.3%) participants were WHO stage 1/2. A total of 56 (3.1%) participants were lost to follow-up (no clinic attendance for >91 days). Before death/loss to follow-up, 12,664/12,944 (97.8%) scheduled visits were completed.

**Table 1:** Characteristics at enrolment.

Factor	Standard ART (*N* = 903)	Raltegravir-intensified (*N* = 902)	All (*N* = 1,805)
Male	482 (53.4%)	479 (53.1%)	961 (53.2%)
Age at last birthday (years)	36 (29–42) [5–77]	35 (29–42) [6–72]	36 (29–42) [5–77]
5–17 years	33 (3.7%)	39 (4.3%)	72 (4.0%)
HIV VL (c/mL) (*N* = 1,804)	250,000 (95,450–631,560)	246,700 (94,000–578,080)	249,770 (95,280–606,360)
≥100,000 c/mL	667/902 (73.9%)	667/902 (73.9%)	1,334/1,804 (73.9%)
<1,000 c/mL**	7/902 (0.8%)	7/902 (0.8%)	14/1,804 (0.8%)
CD4 count* (cells/mm^3^)	36 (16–61)	38 (16–64)	37 (16–63)
0–24 cells/mm^3^	335 (37.1%)	321 (35.6%)	656 (36.3%)
25–49 cells/mm^3^	256 (28.3%)	253 (28.0%)	509 (28.2%)
Weight* (kg) (*N* = 1,800)	52.9 (46.7–59.8)	52.3 (45.8–59.0)	52.5 (46.3–59.3)
BMI (kg/m^2^) (*N* = 1,797)	19.3 (17.5–21.6)	19.0 (17.1–21.2)	19.2 (17.2–21.4)
<18 kg/m^2^	302/900 (33.6%)	323/897 (36.0%)	625/1,797 (34.8%)
WHO stage			
1	145 (16.1%)	155 (17.2%)	300 (16.6%)
2	290 (32.1%)	264 (29.3%)	554 (30.7%)
3	341 (37.8%)	350 (38.8%)	691 (38.3%)
4	127 (14.1%)	133 (14.7%)	260 (14.4%)
Current tuberculosis disease	137 (15.2%)	134 (14.9%)	271 (15.0%)
Haemoglobin (g/L) (*N* = 1,800)	112 (96–127)	111 (95–127)	112 (96–127)
≤80 g/L	86 (9.6%)	90 (10.0%)	176 (9.8%)
NRTIs			
Tenofovir/emtricitabine	719 (79.6%)	703 (77.9%)	1,422 (78.8%)
Zidovudine/lamivudine	154 (17.1%)	169 (18.7%)	323 (17.9%)
Abacavir/lamivudine	30 (3.3%)	30^†^ (3.3%)	60 (3.3%)
NNRTI			
Efavirenz	816^‡^ (90.4%)	803 (89.0%)	1,619 (89.7%)
Nevirapine	87 (9.6%)	99 (11.0%)	186 (10.3%)
Randomised to receive enhanced anti-infection prophylaxis	451 (49.9%)	455 (50.4%)	906 (50.2%)
Randomised to receive RUSF	449 (49.7%)	448 (49.7%)	897 (49.7%)

*Mean of screening and enrolment values. Eligibility required screening CD4 to be <100 cells/mm^3^, so baseline can be above 100, depending on the CD4 at enrolment.

**Potentially indicating undisclosed prior ART: median CD4 was 76 cells/mm^3^ in these participants.

^†^One child was mistakenly initiated on Aluvia (lopinavir/ritonavir) rather than abacavir/lamivudine (plus efavirenz and raltegravir); substituted with abacavir/lamivudine after 4 weeks.

^‡^One adult took tenofovir/emtricitabine alone for 4 days in error before adding efavirenz on day 4.

Note: Showing *n* (%) or median (IQR) [range].

Abbreviations: CD4, cluster of differentiation 4; NNRTI, non-nucleoside reverse transcriptase inhibitor; NRTI, nucleoside reverse transcriptase inhibitor; RUSF, ready-to-use supplementary food; VL, viral load.

### Randomised interventions

A total of 901 (99.9%) participants randomised to raltegravir-intensified ART were prescribed raltegravir. Overall, in the first 12 weeks on ART the raltegravir-intensified ART group spent 98.2% of person-time prescribed raltegravir versus 0.0% in the standard ART group, compared with 0.6% versus 0.1%, respectively, from 12 to 48 weeks (**S1 Fig**). Self-reported ART adherence was high but was significantly poorer in the raltegravir-intensified ART group during the first 12 weeks only (*p* < 0.001) (overall *p* = 0.08, **S2A Fig**), particularly in those taking other ART once daily (**S2B Fig**). At last follow-up, 885 (98.0%) standard ART versus 880 (97.6%) raltegravir-intensified ART participants remained on first-line ART (exact *p* = 0.53), with 67 (7.4%) standard ART versus 52 (5.8%) raltegravir-intensified ART participants having made within-class substitutions (exact *p* = 0.18).

### VL suppression

**Fig 2:**
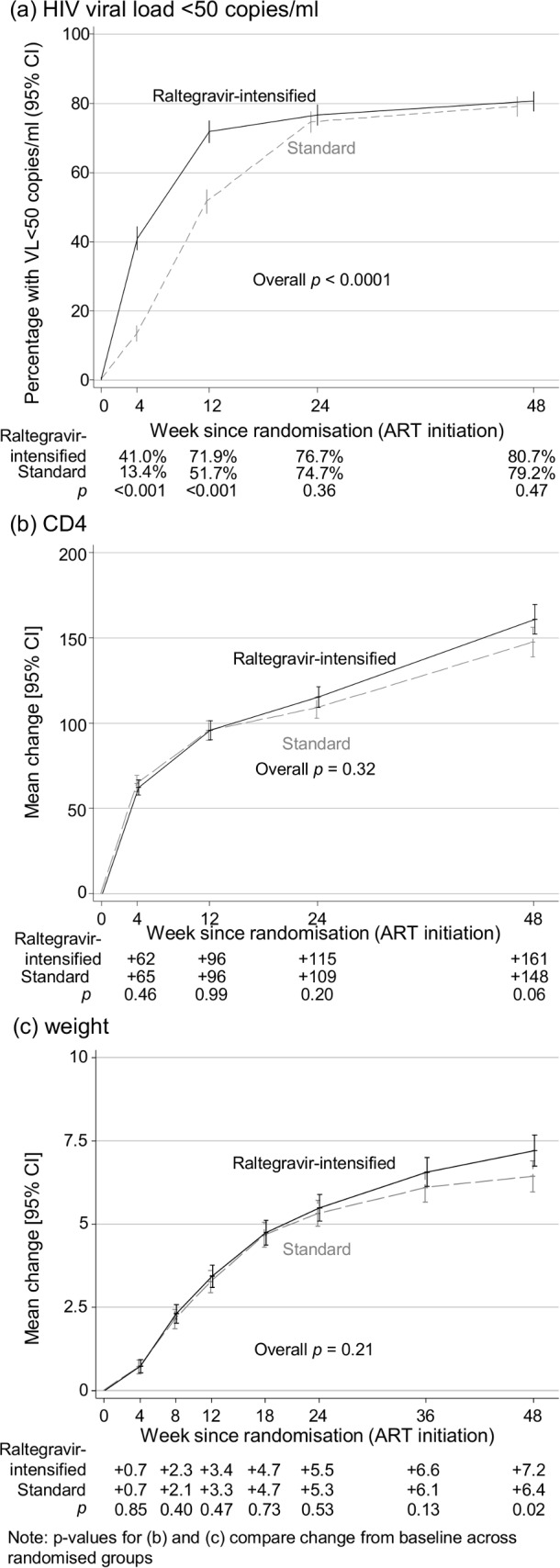
HIV viral load, CD4 cell count, and weight. ART, antiretroviral therapy; CD4, cluster of differentiation 4; VL, viral load.

Despite slightly poorer self-reported adherence, early VL suppression was significantly and substantially faster with raltegravir intensification (**Fig 2A**), with 41.0% (343/836), 71.9% (567/789), 76.7% (594/774), and 80.7% (661/757) participants, respectively, recording <50 copies/mL 4, 12, 24, and 48 weeks after randomisation versus 13.4% (113/841), 51.7% (415/803), 74.7% (586/784), and 79.2% (591/746) in the standard ART group, respectively (*p* < 0.001, *p* < 0.001, *p* = 0.36, and *p* = 0.47, respectively). Overall, therefore, 59.4% versus 43.6% of time at risk through 24 weeks was spent with VL <50 copies/mL in raltegravir-intensified versus standard ART, respectively. At higher thresholds, differences at week 4 were even greater and attenuated more rapidly but persisted through 24 weeks (**S3 Fig**). At 48 weeks, there was no evidence of differential suppression at 50–5,000 copies/mL (*p* > 0.4). Mean change in log_10_ VL to week 4 was −3.4 (standard error [SE] 0.03) versus −2.7 (SE 0.03) in raltegravir-intensified ART versus standard ART (*p* < 0.001), with geometric mean VL 80 c/mL and 480 c/mL, respectively.

### Mortality

**Fig 3:**
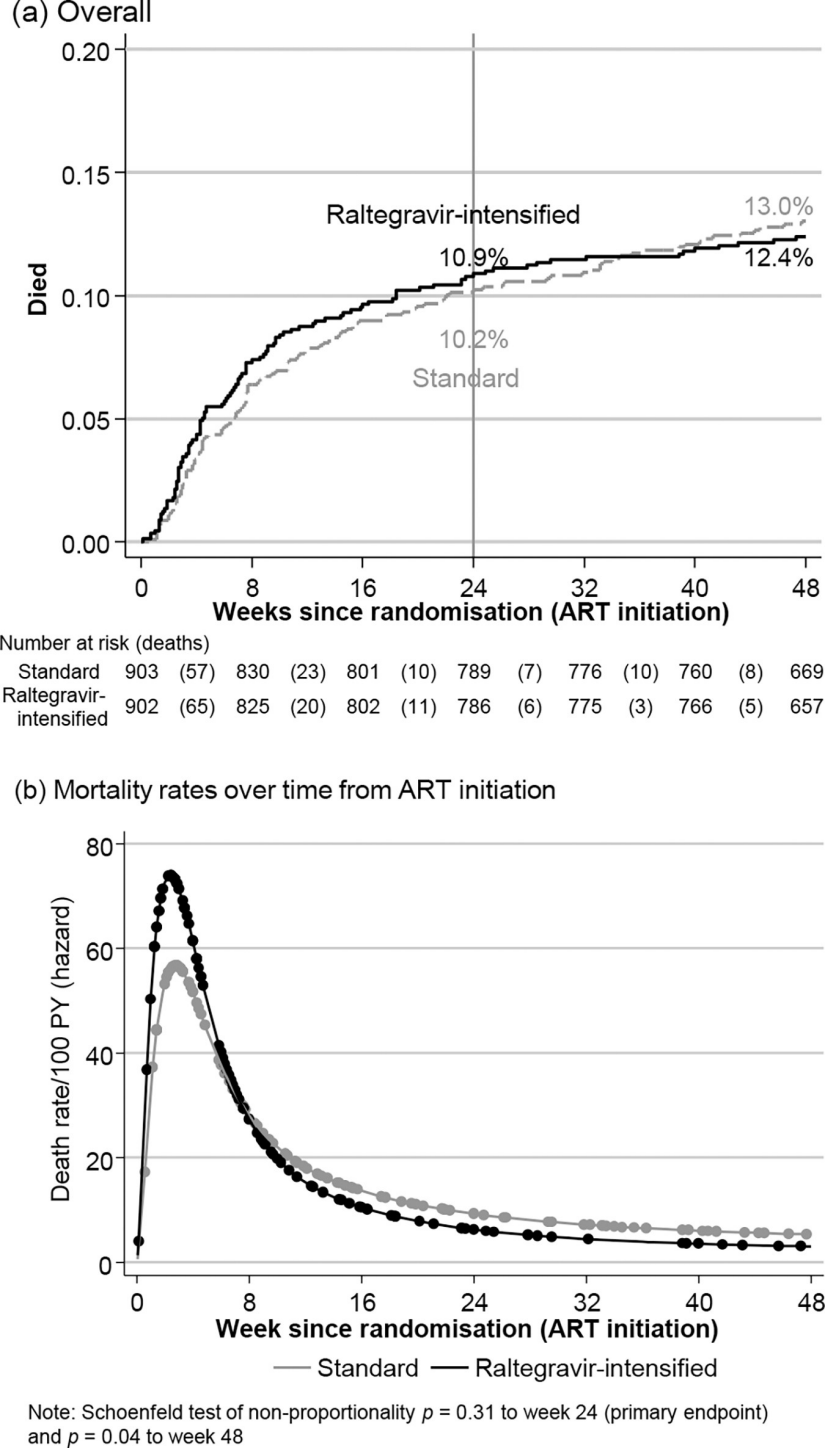
Mortality. ART, antiretroviral therapy; PY, person-years.

By 24 weeks (primary endpoint), 97 (10.9%) raltegravir-intensified ART versus 91 (10.2%) standard ART participants died (adjusted hazard ratio [aHR] = 1.10 [95% CI 0.82–1.46], log-rank *p* = 0.53, **Fig 3A**), with no evidence of interaction with other randomisations (*p*_heterogeneity_ > 0.7). Absolute 24-week mortality difference was +0.6% (−2.2%, +3.5%). By 48 weeks, 110 (12.4%) versus 115 (13.0%) participants, respectively, had died (aHR = 0.98 [0.76–1.28], log-rank *p* = 0.91; absolute difference −0.6% [−3.7%, +2.5%]) with no evidence of differences by cause (**Table 2**). There was no difference in longer-term (after week 48) mortality (aHR = 0.95 [0.74–1.21], log-rank *p* = 0.69, **S4 Fig**). Estimated mortality rates decreased sharply from day 19 through week 12 (**Fig 3B**), with 71 (31.6%) of the 225 deaths occurring by 4 weeks, and 147 (65.3%) by 12 weeks. There was no evidence of early differences in mortality rates (**S5 Fig**) and no evidence of heterogeneity in the impact of raltegravir intensification over time on ART (*p*_heterogeneity_ = 0.14, comparing 0–24 versus 24–48 versus 48+ weeks on ART). Of nine preplanned and five exploratory subgroup analyses (**S6 Fig**; details in S1 Text), only one had weak evidence for variation across dual NRTIs (*p*_heterogeneity_ = 0.04, suggesting harm from raltegravir intensification in combination with tenofovir-emtricitabine; *p*_heterogeneity_ = 0.06 comparing tenofovir-emtricitabine versus zidovudine-lamivudine [excluding abacavir-lamivudine]). Specifically, there was no evidence that mortality differences depended on pre-ART VL or pre-ART CD4 (*p*_heterogeneity_ = 0.84/0.49 and 0.22/0.27, respectively, using categorical/continuous interactions). No subgroup analyses suggested heterogeneity in the impact of raltegravir intensification on suppression <50 copies/mL at week 4 (*p*_heterogeneity_ > 0.05) (**S7 Fig**).

**Table 2:** Secondary and other clinical outcomes through 48 weeks.

**Outcome**	**Standard ART** ***N* participants (% of 903) [events]**	**Raltegravir-intensified ART** ***N* participants (% of 902) [events]**	**Total** ***N* participants (% of 1,805) [events]**	**Hazard ratio**^†^ **(95% CI)**	***p***
Death*	115 (13.0%) [115]	110 (12.4%) [110]	225 (12.5%) [225]	0.98 (0.76, 1.28)	0.91
- from tuberculosis	24 (2.7%) [24]	18 (2.0%) [18]	42 (2.3%) [42]	0.75 (0.40, 1.37)^†^	0.35
- from cryptococcal disease	6 (0.7%) [6]	11 (1.2%) [11]	17 (0.9%) [17]	1.84 (0.68, 4.97)^†^	0.23
- from severe bacterial infections	16 (1.8%) [16]	17 (1.9%) [17]	33 (1.8%) [33]	1.07 (0.54, 2.11)^†^	0.86
- from other causes	23 (2.5%) [23]	22 (2.4%) [22]	45 (2.5%) [45]	0.96 (0.53, 1.71)^†^	0.88
- cause unknown	46 (5.1%) [46]	42 (4.7%) [42]	88 (4.9%) [88]	0.91 (0.60, 1.38)^†^	0.66
New WHO 4 event or death	164 (18.2%) [216]	155 (17.2%) [203]	319 (17.7%) [419]	0.97 (0.77, 1.20)	0.75
New WHO 3 or 4 event or death	197 (21.8%) [310]	206 (22.8%) [301]	403 (22.3%) [611]	1.08 (0.89, 1.31)	0.44
New tuberculosis disease*	80 (8.9%) 103	76 (8.4%) 93	156 (8.6%) [196]	0.95 (0.70, 1.31)	0.77
New cryptococcal disease*	18 (2.0%) 25	14 (1.6%) 26	32 (1.8%) [51]	0.78 (0.38, 1.58)	0.49
New candida disease*	16 (1.8%) 18	17 (1.9%) 18	33 (1.8%) [36]	1.06 (0.54, 2.11)	0.86
New presumptive severe bacterial infection*	33 (3.7%) 49	42 (4.7%) 62	75 (4.2%) [111]	1.28 (0.81, 2.02)	0.28
IRIS	86 (9.5%) [89]	89 (9.9%) [91]	175 (9.7%) [180]	1.04 (0.78, 1.40)	0.79
Any SAE*	207 (22.9%) 287	203 (22.5%) 251	410 (22.7%) [538]	0.99 (0.81, 1.21)	0.88
New hospitalisation*	175 (19.4%) [233]	165 (18.3%) [198]	340 (18.8%) [431]	0.94 (0.76, 1.17)	0.59
Grade-4 AE*	188 (20.8%) [267]	165 (18.3%) [237]	353 (19.6%) [504]	0.88 (0.71, 1.09)	0.29
Grade-3 or -4 AE	327 (36.2%) [550]	331 (36.7%) [503]	658 (36.5%) [1053]	1.03 (0.88, 1.20)	0.72
Grade-4 AE definitely, probably, or possibly related to ART	67 (7.4%) [73]	59 (6.5%) [65]	126 (7.0%) [138]	0.89 (0.63, 1.27)	0.52
Grade-4 AE definitely or probably related to ART	28 (3.1%) [28]	16 (1.8%) [18]	44 (2.4%) [46]	0.57 (0.31, 1.06)	0.07
AE leading to ART modification*	66 (7.3%) [73]	59 (6.5%) [66]	125 (6.9%) [139]	0.90 (0.63, 1.27)	0.54
Grade-4 AE definitely, probably, or possibly related to raltegravir	-	31 (3.4%) [34]		-	-
Grade-4 AE definitely or probably related to raltegravir	-	1^‡^ (0.1%) [1]		-	-
AE leading to raltegravir modification	-	19 (2.1%) [19]		-	-

*Secondary outcome prespecified in the protocol.

^†^For causes of death, competing risks subhazard ratio accounting for other causes of death.

^‡^Stevens-Johnson syndrome that was adjudicated as definitely/probably related to efavirenz, raltegravir, and co-trimoxazole and possibly related to fluconazole, tenofovir, and emtricitabine.

Note: Table shows number of patients with one or more episode (percentage of patients) [number of episodes] (e.g., ‘2 (20.0%) [3]’ would indicate a total of three episodes in two patients). No evidence of interaction with other factorial randomisations (*p*_heterogeneity_ > 0.1; 34 tests; testing not conducted for grade-4 AE definitely or probably related to raltegravir as only one event).

Abbreviations: AE, adverse event; ART, antiretroviral therapy; IRIS, immune reconstitution inflammatory syndrome; SAE, serious adverse event.

### Other clinical efficacy outcomes

There was no evidence of an impact of raltegravir intensification on any disease progression clinical outcome (**Table 2**).

### Genotypic resistance

Integrase genotypes were obtained in 33 raltegravir-intensified ART participants with VL >1,000 (median 47,724) copies/mL at 12 weeks (see **S2 Text**). One participant had predicted intermediate-level (mutations T97A+R263K) and two predicted high-level (N155H, T97A+Y143R/C) raltegravir resistance mutations (0.6% of those randomised, accounting for missing genotypes using probability weights). No patient had predicted intermediate- or high-level dolutegravir resistance.

Reverse-transcriptase genotypes were available for 75 raltegravir-intensified ART versus 87 standard ART participants with VL >1,000 (median 89,815) copies/mL at 48 weeks. The NRTI mutation K219E/Q (*p* = 0.004) and the NNRTI mutations K101E/P (*p* = 0.03) and P225H (*p* = 0.007) were less common in raltegravir-intensified ART, with no evidence of differences for other mutations (*p* > 0.1, **S8 Fig**). There was marginal evidence suggesting less intermediate- or high-level resistance with raltegravir-intensified ART to tenofovir (24.0% [18/75] raltegravir-intensified ART versus 37.9% [33/87] standard ART, *p* = 0.06), abacavir (40.0% [30/75] raltegravir-intensified ART versus 54.0% [47/87] standard ART, *p* = 0.08), and rilpivirine (38.7% [29/75] raltegravir-intensified ART versus 52.9% [46/87] standard ART, *p* = 0.07) (**S9 Fig**) (see S2 Text for details).

### CD4 and CD8 count and body composition

Absolute CD4 count increases were similar in both groups through 24 weeks (*p* = 0.76; **Fig 2B**); however, there was marginal evidence of a small difference at 48 weeks (+161 [standard deviation ±4.4] cells/mm^3^ raltegravir-intensified ART versus +148 [±4.4] cells/mm^3^ standard ART, adjusted difference +11.4 [95% CI −0.4 to +23.1], *p* = 0.06). There was similar weak evidence of slightly (2%–5%) greater percentages with CD4 ≥ 200 cells/mm^3^ at later time points (*p* = 0.049 at week 24, *p* = 0.07 at week 48, **S10 Fig**). Changes in weight were also similar through week 24 (*p* = 0.52; **Fig 2C**), with small but significant differences appearing from 36–48 weeks. Similar late differences were observed for BMI in adolescents/adults (data not shown), fat mass (**S11A and S11B Fig**), and muscle mass (**S11C and S11D Fig**). There was no evidence of heterogeneity in these differences by age (**S12 Fig**). Absolute CD8 count increases were similar in both groups through 48 weeks (*p* = 0.82, **S13 Fig**).

### Safety outcomes

There was no evidence for differences in time to first SAEs (log-rank *p* = 0.88; **S1 Table**), grade-4 AEs (log-rank *p* = 0.29), grade-3/4 AEs (log-rank *p* = 0.72), grade-4 AEs definitely/probably/possibly related to ART (log-rank *p* = 0.52), grade-4 AEs definitely/probably related to ART (log-rank *p* = 0.07), AEs leading to ART modification (log-rank *p* = 0.51) or new hospitalisations (log-rank *p* = 0.59) (**Table 2**). Raltegravir was modified for AEs in 19 (2.1%) participants (**S2 Table**), including renal (*n* = 6) events, hepatic (*n* = 6) events, hypersensitivity reactions (*n* = 3; two discontinued with efavirenz, which was subsequently restarted; one discontinued with tenofovir+emtricitabine+efavirenz, subsequently started lopinavir+ritonavir+tenofovir+emtricitabine+raltegravir), and Stevens-Johnson syndrome in one participant (discontinued lamivudine+zidovudine+efavirenz+raltegravir at week 7). Only one participant experienced a grade-4 AE adjudicated as definitely/probably related to raltegravir (**Table 2**). There was no evidence of any difference in total hospitalisation-days (2,367 raltegravir-intensified ART versus 2,685 standard ART, rank-sum *p* = 0.56) or total hospitalisations (198 versus 233, respectively, Poisson *p* = 0.09).

### IRIS (exploratory analysis)

Fatal or nonfatal events judged compatible with IRIS occurred in 89 (9.9%) raltegravir-intensified ART versus 86 (9.5%) standard ART participants (log-rank *p* = 0.79) (**Table 2**); 36 (4.0%) versus 31 (3.4%), respectively, experienced fatal IRIS events (*p* = 0.54). Tuberculosis IRIS events occurred in 53 (5.9%) participants with raltegravir-intensified ART versus 54 (6.0%) with standard ART (exact *p* = 1.00) and cryptococcal IRIS events in 15 (1.7%) versus 16 (1.8%) participants, respectively (exact *p* = 1.00) (**S3 Table**). IRIS events occurred a median 3.4 (IQR 2.0–6.3) weeks from randomisation, with rates declining from the third week on ART (**S14 Fig**). IRIS was more common in participants initiating ART at older ages (*p* = 0.005), with lower CD4 counts (*p* < 0.001) or with pre-existing TB (*p* = 0.007); IRIS was less common in those initiating ART with enhanced prophylaxis against opportunistic infections (*p* = 0.001) (**S4 Table**). There was no evidence that raltegravir intensification was associated with IRIS after adjusting for these factors (*p* = 0.63).

## Discussion

In this large trial in adults and older children with CD4 <100 cells/mm^3^ in sub-Saharan Africa, we found that 12-week raltegravir intensification of standard ART was well tolerated and reduced plasma HIV VL substantially faster than standard ART alone, but that this had no discernible clinical benefit and no effect on mortality; however, nor was there any evidence of increased rates of IRIS. This is important given the current move to first-line INSTI-based ART; all INSTIs have similarly rapid VL reductions [24], so this result can likely be extrapolated to other INSTIs.

The underpinning hypothesis was that mortality might be reduced by accelerating plasma HIV RNA decline with INSTIs, because initiating ART reduces mortality compared with rates observed pre-ART [25]. One reason this did not occur could be that the differential VL suppression, although significant and substantial, was still too small to reduce mortality. As VL reductions are similar across INSTIs [24], other INSTIs would likely produce similar results. Other explanations for lack of mortality benefit despite significantly faster VL declines may be because there was a lag in, or no, improvement in early functional CD4 recovery or because the disease may simply have been too severe to be reversed despite rapid VL decline. CD4 and CD8 T cells were the only markers of immune restoration measured in real time in all participants, so we cannot investigate further; but there was no differential effect on CD4 (or CD8) counts, at least in the first 24 weeks. This might be expected, as immune restoration does not only depend on HIV viraemia driving inflammation and T-cell turnover [26]. What is intriguing in this context is the small but significant difference in CD4 count at week 48 following only 12 weeks’ raltegravir intensification, which is consistent with previous findings of modestly greater longer-term CD4 increases in HIV seroconverters initiating integrase-inhibitor-containing ART [27] and the STARTMRK trial [28]. We are unable to explore whether CD4 immune restoration continued to diverge after 48 weeks to larger, more clinically relevant differences [29] but plan to measure inflammatory biomarkers [30,31], T-cell subsets, and markers of T-cell turnover and activation in a subset of participants in whom peripheral blood mononuclear cells were stored. However, our results do suggest that quadruple ART is unlikely to provide any clinical benefit over standard triple-drug ART in patients with VL >100,000 copies/mL or advanced disease. Furthermore, the lack of evidence of clinical benefit does not support evaluation of adjunctive INSTIs in less immunocompromised patients, whose risk of clinical events is markedly lower [5].

With increasing moves towards replacing NNRTIs with INSTIs in first-line ART, findings from retrospective observational studies [32,33] have raised major concerns regarding the potential for increased IRIS events with faster VL declines, which may have particularly important consequences in severely immunocompromised patients and where CD4 counts may no longer be available, so that closer clinical monitoring cannot be instigated. Our randomised trial found no evidence of higher rates of clinical IRIS events associated with the significantly faster VL declines with raltegravir-intensified ART. This finding also has relevance for the increasing numbers of treatment-experienced patients returning after previous disengagement from care [34], for whom INSTIs are an attractive option given challenges in accessing resistance testing. Whilst unaffected by raltegravir intensification, IRIS events were significantly reduced by the enhanced-prophylaxis bundle also investigated in this trial [35].

One potential benefit from faster declines in VL is reduced potential for onward transmission, particularly because almost half of the trial participants had minimal symptoms (WHO stage 1 or 2). However, the differential suppression occurred between weeks 4 and 12, with overall only 15.8% less time spent with VL <50 c/mL in the standard ART group through week 24 (albeit predominantly at the time of greatest risk of clinical events). Therefore, benefits at a population level would likely be modest at best. However, such a strategy might have particular value in swiftly reducing VL in women identified as HIV-infected during pregnancy to reduce mother-to-child transmission [36].

Poorer compliance with raltegravir-intensified ART could explain the lack of clinical benefit, although the substantial differences in early VL suppression suggest that adherence to raltegravir-intensified ART was reasonable. However, the additional twice-daily raltegravir pill reduced self-reported adherence to ART in participants receiving other ART once daily by about 4% (**S2B Fig**). Association with twice-daily dosing, rather than pill burden, is supported by the lack of difference in self-reported ART adherence between participants randomised in this trial to enhanced-prophylaxis versus standard-of-care co-trimoxazole (one additional pill once daily from day 5 to 12 weeks) [17]. Although the induction of UGT1A1 by efavirenz (reducing raltegravir levels by about one third) and/or incomplete adherence in those on twice-daily regimens might have reduced efficacy, there was no evidence of heterogeneity in VL suppression at week 4, or mortality, according to initial NNRTI or according to whether other ART was once daily versus twice daily.

At 48 weeks, 20.0% of participants had a VL ≥50 copies/mL and 12.6% had ≥1,000 copies/mL; this may be unsurprising given advanced disease at ART initiation but limits power to investigate drug resistance. Despite similar rates of virological failure ≥1,000 copies/mL, raltegravir-intensified ART was associated with lower rates of the NRTI mutation K219E/Q and the NNRTI mutations K101E/P and P225H. A limitation is that we did not assay samples at ART initiation; however, randomising large numbers should provide balance between raltegravir-intensified ART and standard ART in transmitted drug resistance. This may suggest that these specific mutations arise early in treatment and, even if then suppressed, ultimately re-emerge at failure. However, differences in predicted NRTI/NNRTI resistance were at most marginal for tenofovir, abacavir, and rilpivirine, and raltegravir intensification did not substantially protect against developing clinically meaningful NRTI/NNRTI resistance. Major integrase mutations potentially compromising raltegravir occurred in very small numbers and did not preclude future use of dolutegravir.

The trial’s major strengths were the mortality primary endpoint in a well-defined high-risk population from eight HIV clinics across four African countries, increasing generalisability. Limitations include the fact that some WHO 3/4 and IRIS events were probably misclassified due to limited clinical, radiological, and/or microbiological information for some participants, reflecting available services at the centres, meaning we were also unable to accurately distinguish between IRIS-associated unmasking versus new active disease. Furthermore, the first post-randomisation CD4 was done at 4 weeks only and VL data were retrospective and not available for review. However, adjudications were made blinded to trial drugs, minimising bias in the overall findings, and the primary endpoint, 24-week mortality, was completely objective. IRIS rates were lower than some previous studies but likely included most clinically important events. Ultimately, the trial was well powered to detect differences in mortality, providing confidence that even if some IRIS events were missed, these were not leading to excess deaths given the relatively tight 95% CI around absolute mortality differences. We did not assess VL response before 4 weeks (first stored sample), when one third of the deaths had already occurred. We were unable to genotype Malawian samples (12.5% of the participants) and obtained integrase genotypes in only 73% of samples with VL >1,000 copies/mL at 12 weeks, possibly because of challenges with primers for non-clade-B viruses. We recruited fewer children than planned, limiting our ability to assess effects in this subgroup.

Overall, REALITY trial findings do not support intensification of ART with the INSTI, raltegravir, for the first 12 weeks in severely immunocompromised adults and children infected with HIV. In contrast, the enhanced prophylaxis bundle also tested in the REALITY trial provided substantial reduction in both mortality [17] and morbidity (including IRIS) [35] and could also be practically implemented at primary care level, provided CD4 counts were available to identify those at risk. Successful future interventions would need to improve both the rapidity and functional quality of immunological recovery and/or reduce inflammation and/or further improve prevention and management of IRIS. Earlier diagnosis and initiation of lifelong ART should continue to be a major focus, particularly in sub-Saharan Africa, as, despite recommendations for universal ART, the proportion of ‘late presenters’ appears to have plateaued in both low- and middle-income countries [37] and high-income countries [38], suggesting that substantial numbers will continue to present late for care in low- and middle-income countries for the foreseeable future. Finally and importantly, this study provides no evidence that moving to INSTIs as part of standard first-line therapy, as currently planned across many low- and middle-income countries, will increase rates of clinically important IRIS in such individuals.

## Supporting information

S1 CONSORT Checklist CONSORT checklist. (DOC)

S1 Protocol Protocol. The full trial protocol can be accessed at http://www.ctu.mrc.ac.uk/research/documents/hiv_protocols/reality_protocol [accessed 24 August 2018].
(PDF)

S1 Text Supplementary methods. (DOC)

S2 Text Supplementary results. (DOC)

S1 Table SAEs. SAE, serious adverse event.
(DOC)

S2 Table AEs (any grade) leading to modification of raltegravir. AE, adverse event.
(DOC)

S3 Table IRIS events. IRIS, immune reconstitution inflammatory syndrome.
(DOC)

S4 Table Baseline predictors of IRIS. IRIS, immune reconstitution inflammatory syndrome.
(DOC)

S5 Table Pairwise Spearman correlations of changes in CD4, weight, fat mass, and muscle mass between weeks 0 and 48. CD4, cluster of differentiation 4.
(DOC)

S1 Fig Raltegravir received over time. (TIF)

S2 Fig Self-reported percentage reporting missing doses of any ART in the last 4 weeks (a) overall and (b) by backbone NRTI and NNRTI frequency. ART, antiretroviral therapy; NNRTI, non-nucleoside reverse transcriptase inhibitor; NRTI, nucleoside reverse transcriptase inhibitor.
(TIF)

S3 Fig VL suppression (a) <400 copies/mL and (b) <1,000 copies/mL. VL, viral load.
(TIF)

S4 Fig Long-term mortality through 120 weeks. (TIF)

S5 Fig Absolute difference in mortality risk over time on ART through 48 weeks. ART, antiretroviral therapy.
(TIF)

S6 Fig Subgroup analyses for mortality through 24 weeks (primary endpoint). (TIF)

S7 Fig Subgroup analyses for VL suppression <50 copies/mL at week 4. VL, viral load.
(TIF)

S8 Fig NRTI and NNRTI mutations in participants with VL >1,000 copies/mL at week 48. NNRTI, non-nucleoside reverse transcriptase inhibitor; NRTI, nucleoside reverse transcriptase inhibitor; VL, viral load.
(TIF)

S9 Fig Intermediate–high-level resistance according to the Stanford algorithm in participants with VL >1,000 copies/mL at week 48. VL, viral load.
(TIF)

S10 Fig CD4 distribution over time (cells/mm^3^). CD4, cluster of differentiation 4.
(TIF)

S11 Fig Changes in body composition, (a) fat mass and (b) muscle mass.
(TIF)

S12 Fig Changes in (a) CD4 cell count and (b) weight in children/adolescents (5–17 years) versus adults (18 years or older) at ART initiation. ART, antiretroviral therapy; CD4, cluster of differentiation 4.
(TIF)

S13 Fig CD8 cell count. CD8, cluster of differentiation 8.
(TIF)

S14 Fig Incidence of IRIS events over time. IRIS, immune reconstitution inflammatory syndrome.
(TIF)

## Abbreviations

AE: adverse event
aHR: adjusted hazard ratio
ART: antiretroviral therapy
CD4: cluster of differentiation 4
ERC: Endpoint Review Committee
GEE: generalised estimating equation
IL-6: interleukin 6
INSTI: integrase strand-transfer inhibitor
IRIS: immune reconstitution inflammatory syndrome
NNRTI: non-nucleoside reverse transcriptase inhibitor
NRTI: nucleoside reverse transcriptase inhibitor
REALITY: Reduction of EArly mortaLITY
RUSF: ready-to-use supplementary food
SAE: serious adverse event
SE: standard error
VL: viral load
