# Supplementary material for: Raltegravir-intensified initial antiretroviral therapy in advanced HIV disease in Africa: A randomised controlled trial
Source: PLoS Med. 2018 Dec 4;15(12):e1002706. doi: 10.1371/journal.pmed.1002706 (PMC6279020; doi:10.1371/journal.pmed.1002706)
Supplement: S1 Protocol — The full trial protocol can be accessed at http://www.ctu.mrc.ac.uk/research/documents/hiv_protocols/reality_protocol [accessed 24 August 2018]. (PDF) [file pmed.1002706.s002.pdf]

Sponsor and funder:

Medical Research Council

Collaborating groups:

MRC Clinical Trials Unit

THE COLLEGE OF MEDICINE
Malawi-Liverpool-Wellcome Trust
Clinical Research Programme

Kenya Medical Research Institute | wellcome trust

Other funders:

UKaid from the Department for International Development

wellcome trust

REALITY

Reduction of EArly mortaLITY in HIV-infected adults and children starting antiretroviral therapy

**Version:**	**1.02**
**Date:**	**06 Dec 2013**

**ISRCTN:**	**43622374**
**NCT:**	**01825031**

Authorised by:

**Name:**	Prof Ian Weller
**Role:**	Chair of TSC
**Signature:**	
**Date:**	6/12/2013

**Name:**	Prof A. Sarah Walker
**Role:**	Trial Statistician
**Signature:**	
**Date**	6/12/2013

**Name:**	Prof Diana M. Gibb
**Role:**	Chief Investigator
**Signature:**	6/12/2013

GENERAL INFORMATION

This document was constructed using the MRC CTU Protocol Template Version 4.0. It describes the REALITY trial, coordinated by the Medical Research Council (MRC) Clinical Trials Unit (CTU), and provides information about procedures for entering participants into it. The protocol should not be used as an aide-memoire or guide for the treatment of other patients. Every care has been taken in drafting this protocol, but corrections or amendments may be necessary. These will be circulated to the registered investigators in the trial, but centres entering patients for the first time are advised to contact the Infections Theme, MRC CTU, London, UK, to confirm they have the most up-to-date version.

COMPLIANCE

The trial will be conducted in compliance with the approved protocol, the Declaration of Helsinki (2008), the principles of Good Clinical Practice (GCP) as laid down by the ICH topic E6 (Note for Guidance on GCP) and applicable national regulations.

SPONSOR

The MRC is the trial sponsor and has delegated responsibility for the overall management of the REALITY trial to the MRC CTU. Queries relating to MRC sponsorship of this trial should be addressed to the Director, Regional Centre London, Aviation House, 125 Kingsway, London WC2B 6NH, UK or via the trial team.

FUNDING

REALITY is funded by Joint Global Health Trials Scheme of the Department for International Development, UK (DFID), the Wellcome Trust and the Medical Research Council (MRC UK) [grant number G1100693]. Additional funding support is provided by the PENTA foundation. Trial drugs are supplied by GlaxoSmithKline, Gilead Sciences, Merck and Cipla. Ready to use Supplementary Food is supplied by Valid International.

AUTHORISATIONS AND APPROVALS

This trial will be submitted for approval by Research Ethics Committees/Institutional Review Boards in each of the participating countries (Kenya, Malawi, Uganda, Zimbabwe) and the UK; and by all required regulatory authorities in participating countries.

TRIAL REGISTRATION

This trial has been registered with the International Standard Randomized Clinical Trial Number Register, where it is identified as ISRCTN43622374

SAE REPORTING
Within 1 working day of becoming aware of an SAE, please fax a completed Event Form to the MRC CTU on:
Fax: +44 (0) 20 7670 4814 or email information to reality.mrcctu@ucl.ac.uk

TRIAL ADMINISTRATION

Please direct all queries to the REALITY Trial Manager at MRC CTU in the first instance; clinical queries will be passed to the Medical Experts via the CTU Trial Manager.

COORDINATING CENTRE

MRC Clinical Trials Unit (CTU)<br>Infections Theme<br>Aviation House, 125 Kingsway, London<br>WC2B 6NH, UK	Tel:<br>Fax :<br>Email:	+44 (0) 20 7670 4700<br>+44 (0) 20 7670 4816<br>reality.mrcctu@ucl.ac.uk

MRC CTU STAFF AND AFFILIATES

Chief Investigator:	Prof Diana M Gibb, MD MSc	Tel:<br>Email:	+44 (0) 207 670 4905<br>diana.gibb@ucl.ac.uk
Co-investigators:	Dr Andrew Prendergast, MA DPhil MRCPCH	Tel:<br>Email:	+44 (0) 207 882 2615<br>a.prendergast@qmul.ac.uk
Clinical Project Manager:	Dr Margaret Thomason, PhD MSc	Tel:<br>Email:	+44 (0) 207 670 4615<br>m.thomason@ucl.ac.uk
Trial Manager:	Dr Anna Griffiths, PhD	Tel:<br>Email:	+44 (0) 207 670 4763<br>anna.griffiths@ucl.ac.uk
Laboratory/Sub-studies Trial Manager	Dr Moira Spyer, PhD	Tel:<br>Email:	+44 (0) 207 670 4948<br>moira.spyer@ucl.ac.uk
Data Manager:	Chathurika Rajapakse,	Tel:<br>Email:	+44 (0) 207 670 4633<br>c.rajapakse@ucl.ac.uk
Statisticians:	Prof Sarah Walker, PhD MSc	Tel:<br>Email:	+44 (0) 207 670 4726<br>rmjlasw@ucl.ac.uk
	Dr Angela Crook, PhD	Tel:<br>Email:	+44 (0) 207 670 4751<br>angela.crook@ucl.ac.uk
	Alex Szubert, MSc	Tel:	+44 (0) 207 670 4746

		Email:	a.szubert@ucl.ac.uk
Clinical Reviewer	Dr Sarah Pett,	Tel:<br>Email:	s.pett@ucl.ac.uk

* representing the PENTA Foundation.

OTHER TRIAL INVESTIGATORS

ZIMBABWE: Country Principal Investigator Prof James Hakim			
University of Zimbabwe Clinical Research Centre (UZCRC)<br>1578 Avondale<br>Harare, Zimbabwe			
Principal Investigator:	Prof James Gita Hakim, PhD	Tel:<br>Email:	+263 4 705 986<br>jhakim@mweb.co.zw
Co-Principal Investigator:	Prof Kusum Nathoo, MBChB, DCH, MSc	Tel:<br>Email:	+ 263 4 791 631 ext 2224<br>knathoo@medsch.uz.ac.zw<br>knathoo@mweb.co.zw
Co-investigators:	Dr Andrew Reid, MBChB	Tel:<br>Email:	+263 4 705 986<br>reid@uzcrc.co.zw
	Dr Mutsa Bwakura-Dangarembizi, MBChB, MMed	Tel:<br>Email:	+263 4 705986<br>dangas@zol.co.zw
Trial Manager:	Ennie Chidziva, RGN	Tel:<br>Email:	+ 263 4 705986<br>ennie@uzcrc.co.zw

UGANDA: Country Principal Investigator Prof Peter Mugyenyi			
Joint Clinical Research Centre (JCRC), Kampala<br>Plot 893 Ring Road<br>Butikiro house – Mengo<br>Kampala, Uganda			
Principal Investigator:	Prof Peter Mugyenyi, MBChB, FRCP, DSc	Tel:<br>Email:	+256 414 342 632<br>p.mugyenyi@jcrc.co.ug
Co-investigators:	Dr Cissy Kityo Mutuluuza, MSc ,MBChB<br>Dr Victor Musiime, MBChB, MMed	Tel:<br>Email:<br>Tel:<br>Email:	+256 414 270 283<br>ckityo@jcrc.co.ug<br>+ 256 414 270622<br>vmusiime@jcrc.co.ug
Trial Manager:	Priscilla Wavamunno	Tel:<br>Email:	TBA<br>priswavah@yahoo.com
Joint Clinical Research Centre (JCRC), Gulu<br>Plot 4A Peter Bolo Opok Road<br>P.O. Box 160<br>Gulu, Uganda			

Site Investigators:	Dr George Abongomera, MBChB, MSc	Tel:<br>Email:	+256 471 432 407 or<br>+256 772 587 449<br>gabongomera@jcrc.co.ug
Trial Manager:	James Abach	Tel:<br>Email:	+ 256 772936528abachjames@gmail.com
Joint Clinical Research Centre (JCRC), Mbarara<br>P.O Box 40<br>Mbarara Regional referral Hospital<br>Mbarara, Uganda			
Site Investigator:	Dr Abbas Lugemwa, MBChB	Tel:<br>Email:	+ 256 485 433 545<br>alugemwa@ugabytes.org
Trial Manager:	Sandra Mwebe	Tel:<br>Email:	+ 256 485 433 545<br>sandramwebe@yahoo.com
Mbale Regional Referral Hospital<br>Pallisa Road Zone<br>Mbale, Uganda			
Site Investigator:	Mary Abwola Olwedo	Tel:<br>Email:	TBA<br>maryabwolacherire@yahoo.com
Trial Manager:	Andrew Mukuye	Tel:<br>Email:	TBA<br>mukdre@yahoo.com
Joint Clinical Research Centre (JCRC), Fort Portal<br>PO Box 10,<br>Fort Portal, Uganda			
Site Investigator:	Alexander Amone	Tel:<br>Email:	TBA<br>alexander.amone@yahoo.com
Trial Manager:	Mable Kemigisa	Tel:<br>Email:	TBA<br>mablekemigisa@yahoo.com

MALAWI: Country Principal Investigator Dr Jane Mallewa			
Department/College of Medicine and Malawi-Liverpool-Wellcome Trust Clinical Research Programme<br>University of Malawi<br>Chichiri, Blantyre 3, Malawi			
Principal investigator:	Dr Jane Mallewa, MBBS, MRCP, MD	Tel:<br>Email:	+265 99 371 1274<br>janemallewa@yahoo.com
	Dr Maria del Carmen Gonzalez MD, Msc	Tel:<br>Email:	+265 991 416 730<br>Carmen.Gonzalez@liverpool.ac.uk
	Prof Rob Heydermann, PhD FRCP DTM&H	Tel:<br>Email:	+265 1875918 / +265 1876444<br>R.Heyderman@liverpool.ac.uk
Trial Manager:	Linda Nyondo Mipando, BN-AP, MN-CH	Tel:<br>Email:	TBA<br>lnmipando@mlw.medcol.mw

KENYA: Country Principal Investigator Dr Anthony Etyang			
KEMRI Wellcome Trust Research Programme<br>PO BOX 230<br>Kilifi, 80108 Kenya			
Principal Investigator:	Dr Anthony Etyang, MBChB, MMED	Tel:<br>Email:	+ 254 41 752 2063<br>Aetyang@kilifi.kemri-wellcome.org
Co-investigators:	Dr Jay Berkeley, MBBS MTropMed MD MRCP FRCPCH	Tel:<br>Email:	+ 254 41 522535<br>jayberkley@gmail.com
	Prof Kath Maitland, PhD	Tel:<br>Email:	+ 254 41 752 5044<br>kathryn.maitland@gmail.com
	Dr Patricia Njuguna, MBChB, MMED	Tel:<br>Email:	+ 254 41 752 2063<br>PNjuguna@kilifi.kemri-wellcome.org
	Mr Amin Hassan, MSc	Tel:<br>Email:	+ 254 41 752 2063<br>ahassan@kilifi.kemri-wellcome.org
	Shalton Mwaringa, MBChB Dip Paed	Tel:<br>Email:	+ 254 41 752 2063
Trial Manager:	Dr Clara Agutu, MBChB, MPH	Tel:<br>Email:	+254 720735000<br>CAgutu@kemri-wellcome.org

Department of Medicine<br>Moi University School of Medicine<br>Academic Model for the Prevention and Treatment of HIV/AIDS (AMPATH) Centre at Moi Teaching Referral Hospital (MTRH)<br>P.O. Box 4606, 30100<br>Eldoret, Kenya			
Site Investigator:	Dr Abraham M. Siika, MBChB MMed	Tel:<br>Email:	+ 254 53 2060850 OR +254-721280785<br>amsiika@africaonline.co.ke
Co-investigators:	Dr Kara Wools-Kaloustian, MD	Email:	kwools@iupui.edu
	Dr Winston M Nyandiko, MBChB (Department of Child Health and Paediatrics)	Email:	nyandikom@yahoo.com
Trial Manager:	Priscilla C. Cheruiyot, BSN	Tel:<br>Email:	+ 254 53 2060850<br>pcchepkorir@yahoo.com

**PROTOCOL EXPERT: Immunology**			
UCL Institute of Child Health<br>30 Guilford Street<br>London WC1N 1EH<br>UK			
Co-investigator	Prof Nigel Klein, BSc MBBS MRCP PhD FRCPCH	Tel:<br>Email:	+44 (0) 20 7905 2215<br>n.klein@ich.ucl.ac.uk

**PROTOCOL EXPERT: Pharmacology**			
Dep. of Clinical Pharmacy<br>Internal code 864, Radboud University Nijmegen Medical Centre,<br>Geert Grooteplein 10, 6525 GA Nijmegen<br>The Netherlands			
Co-investigator	Prof David Burger, PharmD PhD	Tel:<br>Email:	+31 24 3616405<br>D.Burger@akf.umcn.nl

**PROTOCOL EXPERT: Social Science**
CeSHHAR Zimbabwe<br>9 Monmouth Road<br>Avondale West<br>Harare

Zimbabwe<br>(Department of Infection & Population Health, Institute of Epidemiology and Health Care, UCL)			
Co-investigator	Dr Frances Cowan, FRCP, MSc, MD	Tel:<br>Email:	+263 77 2257949<br>f.cowan@ucl.ac.uk
Social Science Programme,<br>MRC/UVRI Uganda Research Unit on AIDS, P.O. Box 49,<br>Entebbe, Uganda			
Co-investigator	Prof Janet Seeley, PhD	Tel:<br>Email:	+256 417 704141<br>Janet.seeley@mrcuganda.org

SUMMARY OF TRIAL

SUMMARY INFORMATION TYPE	SUMMARY DETAILS
**Acronym**	REALITY
**Long Title of Trial**	Reduction of EArly mortaLITY in HIV-infected adults and children starting antiretroviral therapy
**Version**	1.02
**Date**	06 Dec 2013
**ISRCTN**	43622374
**Study Design**	A 2x2x2 open-label factorial multi-centre trial, conducted in 9 centres in 4 countries (Kenya, Malawi, Uganda, Zimbawe)
**Type of Participants to be Studied**	1800 HIV-infected patients including adults, adolescents and children aged 5 years or older with low CD4 counts about to initiate combination antiretroviral therapy (ART)
**Interventions to be Compared**	Three methods to reduce early mortality following ART initiation<br>(i) increasing the potency of ART with a 12 week induction period using 4 antiretroviral drugs from 3 classes<br>(ii) augmented prophylaxis against opportunistic/bacterial infections and helminths for 12 weeks<br>(iii) macronutrient intervention using ready-to-use supplementary food for 12 weeks.<br>Each intervention will be compared with standard of care, which in previously untreated patients presenting late with very low CD4 counts is to initiate ART with 3 drugs from 2 classes, together with cotrimoxazole prophylaxis and macronutrient intervention only for those with low BMI (or low weight-for-height/mid-upper arm circumference in children).
**Study Hypotheses**	Each of the interventions (augmented anti-HIV potency, augmented prophylaxis, and macronutrient intervention) will reduce early mortality in those starting ART with severe immuno-deficiency.
**Primary Outcome Measure(s)**	All-cause mortality over the first 24 weeks after starting ART
**Secondary Outcome Measure(s)**	▪ 48 week mortality (all-cause)<br>▪ Safety<br>o serious adverse events<br>o grade 4 adverse events<br>o adverse events leading to modification of ART or other study drugs<br>▪ Endpoints relating to the specific mechanisms of action of each intervention<br>o anti-HIV: CD4 cell counts<br>o anti-infection: incidence of TB, cryptococcal and candida disease, severe bacterial infections

SUMMARY INFORMATION TYPE	SUMMARY DETAILS
	○ nutritional: BMI, weight and body fat assessed by bioimpedance analysis (BIA), height (in children), grip strength<br>▪ Hospital inpatient episodes and total days admitted<br>▪ Adherence to ART and acceptability of each strategy
**Other outcomes (assessed in a subset of the population)**	▪ HIV RNA viral load and resistance<br>▪ The immunological effects of the interventions in relation to control of HIV replication, systemic and gut inflammation, microbial translocation and immune reconstitution inflammatory syndrome, specifically<br>1. Cellular immune activation (detailed immunophenotyping and markers of cellular turnover and senescence, particularly CD3+CD8+CD38+HLA-DR+, CD3+CD8-CD38+HLA-DR+)<br>2. Functional whole blood ex-vivo responses to pathogen challenges, including LPS and viral and fungal antigens<br>3. Markers of systemic inflammation (CRP, cytokines, chemokines, D-dimer)<br>4. Markers of enteric inflammation (fecal calprotectin, a1-antitrypsin, neopterin)<br>5. Markers of microbial translocation (soluble CD14, lipopolysaccharide (LPS), anti-LPS antibodies, pathogen detection by 16s PCR)<br>▪ Skinfold thicknesses
**Randomisation**	Participants will be allocated 1:1 to each of the three factorial randomisations
**Number of Participants to be Studied**	1800; up to 600 children aged 5-12 years (minimum 400), and at least 1200 adolescents and adults aged 13+ years
**Duration**	▪ Participants will be randomised over 1.5-2 years.<br>▪ Each intervention will be administered in addition to standard of care for 12 weeks.<br>▪ Each participant will be followed for 48 weeks in the trial.<br>▪ The overall trial duration is 3 years.
**Ancillary Studies/Substudies**	▪ Economics and cost-effectiveness<br>▪ Molecular diagnostics<br>▪ Phenotypic and genotypic analysis of pathogens isolated<br>▪ Social science to identify the most effective ways to promote acceptance of additional strategies in the community
**Sponsor**	Medical Research Council, UK
**Funder**	Department for International Development, UK (DFID), the Wellcome Trust and the Medical Research Council (MRC) UK, the PENTA foundation.
**Chief Investigator**	Prof Diana M Gibb

TRIAL SCHEMA

Figure 1. Trial Entry, Randomisation and Treatment

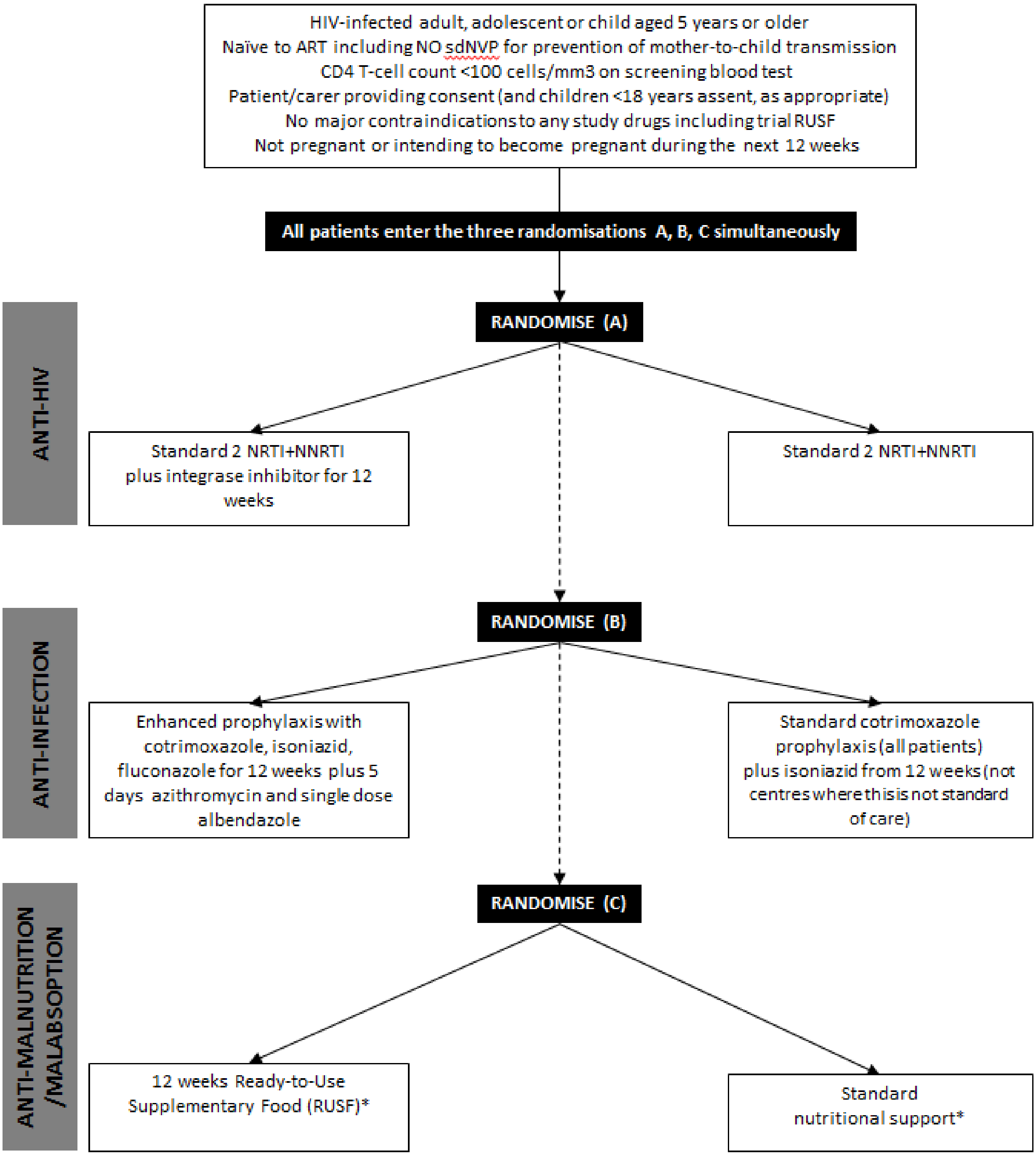

* all patients meeting criteria for Ready to Use Therapeutic Food (RUTF) will receive this, regardless of randomisation, if available locally.

TRIAL ASSESSMENT SCHEDULE

	EVENTS	WEEKS IN TRIAL										
	Doctor/Nurse visit* ▢ Nurse visit ☐	Screening ‡	0	(2)	4	8	12	18	24	36	48	Any acute event (9)
ALL PARTICIPANTS	Drug supply to next visit (4/6/12 weeks)*		X	(X)	X	X	X	X	X	X	X	
ALL PARTICIPANTS	Isoniazid prophylaxis may be given (irrespective of randomisation)†						X	X	X	X	X	
ALL PARTICIPANTS	Routinely de-worm all children (irrespective of randomisation)								X		X	
ALL PARTICIPANTS	Patient information sheet and consent for screening	X										
ALL PARTICIPANTS	Symptom screen for active tuberculosis	X	X									
ALL PARTICIPANTS	Informed consent for trial enrolment		X									
ALL PARTICIPANTS	History & Physical (1)	X	X		X		X		X	X	X	
ALL PARTICIPANTS	Grip strength (measured by handgrip dynamometer)		X		X		X				X	
ALL PARTICIPANTS	Symptom check list and EQ5D		X	(X)	X	X	X	X	X	X	X	
ALL PARTICIPANTS	Peripheral neuropathy assessment		X								X	
ALL PARTICIPANTS	Socioeconomic questionnaire		X								X	
ALL PARTICIPANTS	Acceptability assessment (2)				X		X		X			
ALL PARTICIPANTS	Adherence assessment (3)			(X)	X	X	X	X	X	X	X	
ALL PARTICIPANTS	Pregnancy Test (4)	X			(X)	(X)			X		X	
ALL PARTICIPANTS	Haematology 1.5-2mls (5)	X			X		X		X		X	(X)
ALL PARTICIPANTS	Biochemistry 1.5mls (6)	X			X						X	(X)
ALL PARTICIPANTS	Lymphocyte Subsets (same draw as haematology except w0) (7)	X	X		X		X		X		X	
ALL PARTICIPANTS	Plasma storage 3.5-7ml (8)	X	X		X		X		X	X	X	
ALL PARTICIPANTS	Plasma storage 2-4ml (9)											X
ALL PARTICIPANTS	Store smear/any organism isolated from standard culture (9)											X
SUBSET	Immunophenotyping (using existing BLOOD DRAW) (10)		X		X		X		X		X	
SUBSET	Whole blood stimulation assays (1ml) (11)		X		X		X		X		X	
SUBSET	Stool storage (12)		X		X		X				X	
SUBSET	Quantiferon 3 x 1mls (13) (**)		X		X		X					(**)
SUBSET	Skinfold measurement (14)		X				X				X	

* Participants will return 6-weekly (4-weekly before week 12, 12 weekly after week 24) to see the nurse or doctor, return used drug containers and receive 4/6/12 weeks of antiretroviral therapy (ART) until their next clinic visit. The doctor will prescribe ART and make decisions on any modifications of therapy as necessary. Adults and children taking nevirapine will dose escalate at week 2. An additional week 2 visit may be booked at the physician's discretion, this is recommended for very sick patients.

† Sites will follow national guidelines/local standard of care: where isoniazid is recommended for HIV-infected patients, all patients in REALITY will receive it from 12 weeks on ART. Where it is not recommended/current practice on ART patients randomised to standard prophylaxis will receive only cotrimoxazole.

‡ day -42 to day -1

() indicate tests that may be done, eg if an earlier test is missed, if the patient's condition requires it or allows it, but are not mandatory.

(1) Clinical: including weight, body composition as assessed by bioimedance analysis (BIA), height (at screening and enrolment only for adults aged 18 years or old), mid-upper arm circumference, adverse events and WHO staging for HIV.

(2) Questionnaire to ascertain acceptability of the different types of interventions.

(3) Pill count and patient reported adherence every 4/6 weeks at nurse visits for antiretrovirals and prophylaxis drugs; nurse administered questionnaire at 4, 12, 24 and 48 weeks.

(4) At screening, and subsequently every 24 weeks for girls 12 years or older who have reached menses and pre-menopausal women. Women who have reached menses and are pre-menopausal should also be tested for pregnancy at weeks 4 and 8 if randomised to receive 12 weeks fluconazole at trial enrolment.

(5) Haematology: Hb, WBC, Lymphocytes, Neutrophils, Platelets.

(6) Biochemistry: Creatinine, Albumin, Phosphate at screening, week 4 and week 48. AST or ALT and Bilirubin at screening and week 4 only. After week 4 other biochemistry tests may be performed if clinically indicated, but are not required by the protocol

(7) CD3, CD4, CD8 percentage and absolute, total lymphocyte count.

(8) Plasma (EDTA blood) from a total of 3.5 - 7ml (if Quantiferon bloods are also drawn, only 3.5-4ml EDTA blood to be taken for plasma storage). Store DNA pellet at week –2, 0, 24, 48 if feasible. See Appendix VI - Handling and Storage of Specimens, p120 for instructions about storage. Plasma will be assayed retrospectively in batches for HIV RNA, and for plasma markers of systemic and gut inflammation and microbial translocation, at local laboratories. Note that JCRC sites, Harare and Kilifi do not need to store a DNA/cell pellet at any timepoints where they have been requested to store cells for immunophenotyping (see (10) below).

(9) If children, adolescents or adults become acutely unwell (particularly if they are ill enough to have blood tests done outside of the routine blood sampling schedule) then store plasma (EDTA blood) from a total of 2-4ml and store DNA cell pellet. Store any isolate obtained from any clinical specimen, including sputum, CSF, blood etc. Store any TB smear slides.

Investigations only in a subset of participants

(10) JCRC sites, Harare, Kilifi: Immunophenotyping using the existing blood draw from (8) (no extra bloods) will be done in real-time or on stored cells in local laboratories to subdivide lymphocytes into different populations, according to CD3, CD8,CD38, HLA-DR and other markers of cellular turnover/senescence.

(11) Harare, Kilifi: ex-vivo responses to pathogens in whole blood or peripheral blood mononuclear cells (PBMCs) will be assessed in local laboratories

(12) Harare, Kilifi: stool collection for quantification of faecal markers of enteric inflammation (calprotectin, alpha-1 antitrypsin, neopterin) and storage for later analysis of microbiota. (See Appendix III - Template Patient information sheets and consent for collection of stool samples, p108.) Tests will be done in local laboratories.

(13) JCRC sites and Harare: Quantiferon: a total of 3mls blood drawn in three 1 ml tubes, negative control used for LPS assay. Supernatants stored for subsequent retrospective analysis in local laboratories.

** JCRC sites and Harare: Quantiferon: at any time point where a child, adolescent or adult is diagnosed with TB (pulmonary or extra-pulmonary), supernatant from quantiferon tubes is to be stored at the time of TB diagnosis and 3 months after diagnosis for subsequent retrospective analysis.

(14) Harare: Skinfolds: triceps, subscapular, supra-iliac, mid-thigh skinfold thicknesses will be measured using Holtain calipers and following a standard Manual of Operations.

Therefore the 10-15 mls of blood taken at standard doctor visits is divided as follows: Haematology/lymphocyte subsets/immunophenotyping (1.5 – 2mls); Biochemistry (1.5-2.0 mls) and the remaining 11-12mls divided between Quantiferon and Plasma storage. Priority for blood draw in case of difficulties is (1) CD4 cell count, (2) haematology, (3) biochemistry, (4) plasma storage, (5) Quantiferon storage

CONTENTS

GENERAL INFORMATION 2

SUMMARY OF TRIAL 10

TRIAL SCHEMA 12

TRIAL ASSESSMENT SCHEDULE XIII

CONTENTS 16

ABBREVIATIONS 20

1 BACKGROUND 23

1.1 DISEASE SETTING AND CONTEXT OF THE STUDY 23
1.2 POSSIBLE INTERVENTIONS 23
1.2.1 Increasing ART potency with an induction period using 4 antiretroviral drugs from 3 classes 23
1.2.2 Augmented prophylaxis against Opportunistic Infections (OIs), bacterial infections and helminths 24
1.2.3 Macronutrient intervention 26
1.2.4 Relevant studies underway or planned 27
1.3 RATIONALE FOR THE TRIAL DESIGN 27
1.4 OBJECTIVES AND HYPOTHESES 29

2 SELECTION OF CENTRES AND CLINICIANS 30

2.1 CENTRE/INVESTIGATOR INCLUSION CRITERIA 30
2.1.1 Centre PI Qualifications & Agreements 30
2.1.2 Adequate Resources 30
2.1.3 Centre Assessment 31
2.2 APPROVAL AND ACTIVATION 31

3 SELECTION OF PATIENTS 32

3.1 PATIENT INCLUSION CRITERIA 32
3.2 PATIENT EXCLUSION CRITERIA 32
3.3 NUMBER OF PATIENTS 33
3.4 SCREENING PROCEDURES 33
3.4.1 Screening visit 33

4 RANDOMISATION 35

4.1 RANDOMISATION VISIT 35
4.2 RANDOMISATION PRACTICALITIES 36
4.2.1 Enrolment of multiple participants from the same family into the trial 36
4.3 CO-ENROLMENT GUIDELINES 37

5 TREATMENT OF PATIENTS 38

5.1 INTRODUCTION AND STANDARD OF CARE 38

5.1.1 Standard of care antiretroviral therapy 38
5.1.2 Standard of care OI prophylaxis 42
5.1.3 Standard of care nutritional support....................43
5.2 RANDOMISATION A: +/- 12 WEEKS INDUCTION WITH AN INTEGRASE INHIBITOR 44
5.2.1 Products and dosing schedule....................44
5.2.2 Raltegravir dose modifications, interruptions & discontinuations 44
5.3 RANDOMISATION B: +/- 12 WEEKS ENHANCED OPPORTUNISTIC INFECTION (OI) PROPHYLAXIS....................45
5.3.1 Products and dosing schedule....................45
5.3.2 Fluconazole and azithromycin dose modifications, interruptions & discontinuations....................46
5.4 RANDOMISATION C: +/- NUTRITIONAL SUPPORT 46
5.4.1 Products....................46
5.5 DISPENSING 46
5.6 OVERDOSE OF TRIAL MEDICATION 47
5.7 DOSE MODIFICATIONS, INTERRUPTIONS & DISCONTINUATIONS OF STANDARD ART AND COTRIMOXAZOLE OR ISONIAZID PROPHYLAXIS....................47
5.7.1 Information on specific ART drug toxicities....................48
5.7.2 ART drug substitutions 49
5.7.3 Management of potential ABC or NVP reactions when children are taking both 50
5.8 PROTOCOL TREATMENT DISCONTINUATION 50
5.9 COMPLIANCE & ADHERENCE 51
5.10 NON-TRIAL TREATMENT....................51

6 ASSESSMENTS & FOLLOW-UP 52

6.1 TRIAL ASSESSMENT SCHEDULE 52
6.2 PROCEDURES FOR ASSESSING EFFICACY....................54
6.2.1 Clinical events (All participants) 54
6.2.2 CD4+ T-cell counts (All participants)....................54
6.2.3 Viral load testing (All participants; additional testing in a subset)....................55
6.2.4 Resistance testing (Subset of participants) 55
6.2.5 Weight, body composition, growth and grip strength (All participants; additional testing in a subset) 55
6.2.6 Measures of the rate and quality of the immune restoration (Subset of participants)....................56
6.3 PROCEDURES FOR ASSESSING SAFETY (ALL PARTICIPANTS)....................56
6.4 PROCEDURES FOR ASSESSING ADHERENCE (ALL PARTICIPANTS) 57
6.5 OTHER ASSESSMENTS 57
6.5.1 Health economics (All participants) 57
6.5.2 Molecular diagnostics (Subset of participants) 58
6.5.3 Procedures for assessing socioeconomic status (All participants)....................58
6.6 MANAGEMENT OF PREGNANCY 58
6.6.1 Summary of current drug safety profiles in pregnancy....................59
6.6.2 Treatment modification 59
6.6.3 Pregnancy follow-up and reporting....................59
6.7 MANAGEMENT OF TUBERCULOSIS....................60
6.8 EARLY STOPPING OF FOLLOW-UP....................60
6.9 PATIENT TRANSFERS....................61
6.10 LOSS TO FOLLOW-UP 61
6.11 TRIAL CLOSURE....................61

7 SAFETY REPORTING.. 62

7.1 SAFETY DEFINITIONS...62
7.1.1 Medicinal Products...63
7.1.2 Exempted Adverse Events...63
7.1.3 Disease-related Events...63
7.2 INVESTIGATOR RESPONSIBILITIES...63
7.2.1 Investigator Assessment...64
7.2.2 Notification Procedure...65
7.3 MRC CTU RESPONSIBILITIES...65
7.4 RESPONSIBILITIES OF COUNTRY PRINCIPAL INVESTIGATOR...............................66

8 QUALITY ASSURANCE & CONTROL... 67

8.1 RISK ASSESSMENT...67
8.2 CENTRAL MONITORING AT MRC CTU...67
8.3 ON-SITE MONITORING...67
8.3.1 Direct Access to Patient Records...68
8.3.2 Confidentiality..68

9 STATISTICAL CONSIDERATIONS.. 69

9.1 METHOD OF RANDOMISATION..69
9.2 OUTCOME MEASURES...69
9.3 SAMPLE SIZE..70
9.4 INTERIM MONITORING & ANALYSES..71
9.5 POTENTIAL FOR INTERACTIONS BETWEEN THE TRIAL INTERVENTIONS......................71
9.6 ANALYSIS PLAN (BRIEF)..71

10 ANCILLARY STUDIES.. 73

10.1 HEALTH ECONOMICS (ALL PARTICIPANTS)..73
10.2 MOLECULAR DIAGNOSTICS (ALL PARTICIPANTS WITH ACUTE ILLNESSES AND STORED SPECIMENS).............73
10.3 GENOTYPIC AND PHENOTYPIC CHARACTERISATION OF PATHOGEN ISOLATES.....................74
10.4 SOCIAL SCIENCE SUBSTUDY..74

11 REGULATORY & ETHICAL ISSUES... 75

11.1 COMPLIANCE..75
11.1.1 Regulatory Compliance..75
11.1.2 Centre Compliance...75
11.1.3 Data Collection & Retention..75
11.2 ETHICAL CONDUCT OF THE TRIAL..75
11.2.1 Ethical Considerations...75
11.2.2 Safety profile of Raltegravir...76
11.2.3 Burden of investigations...76
11.2.4 Post-trial treatment...76
11.2.5 Informing potential trial participants of possible benefits and known risks..................77
11.2.6 Confidentiality..77
11.2.7 Ethical Approvals..77
11.3 COMPETENT AUTHORITY APPROVALS..77

12 INDEMNITY 78
13 FINANCE 79
14 OVERSIGHT & TRIAL COMMITTEES 80
14.1 SITE TRIAL MANAGEMENT TEAMS 80
14.2 TRIAL MANAGEMENT GROUP (TMG) 80
14.3 TRIAL STEERING COMMITTEE (TSC) 80
14.4 DATA MONITORING COMMITTEE (DMC) 81
14.5 OTHER COMMITTEES 81
14.5.1 Endpoint Review Committee 81
14.6 ROLE OF STUDY SPONSOR 81
15 PUBLICATION 83
16 PROTOCOL AMENDMENTS 85
17 REFERENCES 86
APPENDIX I - TEMPLATE PATIENT INFORMATION SHEETS 91
APPENDIX II - SCREENING AND TRIAL CONSENT AND ASSENT FORMS 101
APPENDIX III - TEMPLATE PATIENT INFORMATION SHEETS AND CONSENT FOR COLLECTION OF STOOL SAMPLES (HARARE, KILIFI ONLY) 108
APPENDIX IV- DETAILS OF ANTIRETROVIRAL THERAPY 114
APPENDIX V - TABLE OF CLINICAL SIGNS, SYMPTOMS, MONITORING AND MANAGEMENT OF SYMPTOMS OF SERIOUS ADVERSE EFFECTS OF ANTIRETROVIRAL DRUGS THAT REQUIRE DRUG DISCONTINUATION (ADAPTED FROM WHO GUIDELINES ANNEX 11B) 118
APPENDIX VI - HANDLING AND STORAGE OF SPECIMENS 120
APPENDIX VII - EQ-5D QUESTIONNAIRE 122
APPENDIX VIII - WITHDRAWAL FORM 123
APPENDIX IX - CLINICAL PROGRESSION AND WHO DEFINITIONS 125
APPENDIX X - TOXICITY GRADINGS AND MANAGEMENT 136
APPENDIX XI – SOCIAL SCIENCE SUBSTUDY 155

ABBREVIATIONS

3TC	Lamivudine
d4T	Stavudine
ABC	Abacavir
AE	Adverse event
AIDS	Acquired Immune Deficiency Syndrome
AR	Adverse reaction
ART	Antiretroviral therapy
ARV	Antiretroviral
AUC	Area under the curve
BIA	Bioelectrical impedance analysis
BID	Bis in die (twice a day)
BMI	Body Mass Index
bPI	boosted Protease Inhibitor
BSA	Body surface area
CAB	Community Advisory Board
CDC	Centers for Disease Control (USA)
CEA	Cost-effectiveness analysis
CI	Chief Investigator
CI	Confidence interval
Cmax	Maximum concentration
CRF	Case Report Form
CTU	Clinical Trials Unit
DMC	Data Monitoring Committee
DOB	Date of birth
EDTA	Ethylenediaminetetraacetic Acid
EFV	Efavirenz
ERC	Endpoint Review Committee
EQ5D	European Quality of Life 5 Dimensions (EuroQoL 5D)
FDA	Food and Drug Administration (USA)
GCP	Good Clinical Practice
HE	Health economics
HIV	Human Immunodeficiency Virus
HSR	Hypersensitivity reaction

ICH	International Conference on Harmonisation of Technical Requirements for Registration of Pharmaceuticals for Human Use
IRB	Institutional Review Board
IRIS	Immune reconstitution inflammatory syndrome
ISRCTN	International Standard Randomised Controlled Trial Number
ITT	Intention-to-treat
JCRC	Joint Clinical Research Centre
LFTs	Liver function tests
MOP	Manual of Operations
MRC	Medical Research Council
MUAC	Mid-upper arm circumference
MVMM	Multi-vitamin multi-mineral (supplements)
NRTI	Nucleoside reverse transcriptase inhibitor
NNRTI	Non-nucleoside reverse transcriptase inhibitor
NVP	Nevirapine
OD	Once daily
OI	Opportunistic infection
PPD	Purified Protein Derivative
PI	Principal Investigator
PIS	Patient Information Sheet
PK	Pharmacokinetics
QMC	Quality Management Committee
QoL	Quality of life
qPCR	Quantitative polymerase chain reaction
RAL	Raltegravir
RCT	Randomised controlled trial
rDNA	Ribosomal DeoxyriboNucleic Acid
REC	Research Ethics Committee
RUF	Ready to Use Food
RUSF	Ready to Use Supplementary Food
RUTF	Ready to Use Therapeutic Food (for those with severe malnutrition)
SAE	Serious adverse event
SAP	Statistical Analysis Plan
SAR	Serious adverse reaction

SD	Standard deviation
SOC	Standard of Care
SOP	Standard operating procedure
SPC	Summary of Product Characteristics
SUSAR	Suspected unexpected serious adverse reaction
TB	Tuberculosis
TDF	Tenofovir
TMF	Trial Master File
TMG	Trial Management Group
TMT	Trial Management Team
TSC	Trial Steering Committee
TST	Tuberculin skin test
UAR	Unexpected adverse reaction
VL	Viral load
WHO	World Health Organization
ZDV	Zidovudine

1 BACKGROUND

1.1 DISEASE SETTING AND CONTEXT OF THE STUDY

HIV-infected adults and children in resource-limited countries have a clear excess risk of death and disease progression after starting antiretroviral therapy (ART) compared to those in resource-rich countries, which persists after adjusting for disease stage, CD4, age, sex, and ART regimen[1]. Although early mortality has declined at ART programme level in resource-limited settings over the last 5 years, this has been largely driven by greater numbers of less immuno-compromised HIV-infected individuals starting ART; for example, in 7 programmes in South Africa[2], 12 month mortality declined from 9% to 6% over 2002-7 as median CD4 at ART initiation increased from 69 to 113 cells/mm^3. However, early mortality remained almost 6-fold higher in those initiating ART with CD4 <50 versus >200 cells/mm^3 even in the latest period[2]. Data from the DART and ARROW trials (where mortality is likely to be lower than outside research settings) show that children aged 5+ years starting ART with low CD4 are at the same excess risk of death as adults: 24-week mortality in those with pre-ART CD4 0-49 cells/mm^3 was 7.2% in adults and 8.3% in children 5-17 years; 24-week mortality in those with CD4 50-99 cells/mm^3 at ART initiation was 3.4% in adults and 3.5% in children[3].

In 2009, 4.5 million HIV-infected persons with CD4 <200 cells/mm^3 were estimated to still need ART[4]: even if only 20% have pre-ART CD4 <50 cells/mm^3 and experience 8% early mortality, then an intervention with 50% efficacy could save nearly 40,000 lives in this subgroup alone. Thus there remains a major research question: how best to reduce excess early mortality in severely immuno-compromised HIV-infected adults and children initiating ART?

1.2 POSSIBLE INTERVENTIONS

Reasons for high early mortality in severely immuno-compromised HIV-infected adults and children initiating ART are multi-factorial, including high rates of co-infections (TB, bacterial infections, fungal/protozoal infections, parasites), immune reconstitution inflammatory syndrome (IRIS), malnutrition and advanced HIV infection. Several interventions might therefore reduce early mortality in HIV-infected patients starting ART with low CD4 counts, and could form part of a highly effective integrated care "bundle".

1.2.1 Increasing ART potency with an induction period using 4 antiretroviral drugs from 3 classes

A series of recent studies in patients with advanced HIV presenting with tuberculosis have shown a reduction in mortality if ART is started early (within 2-4 weeks of initiation of TB treatment) compared to later (after the intensive phase of TB treatment)[5-8]. If starting ART earlier has clinical benefit, increased potency of initial ART might have a similar impact in patients with low CD4.

HIV integrase inhibitors are a new, highly potent class of antiretrovirals that block integration of proviral DNA into host cell DNA, thereby preventing cellular infection and viral replication. This action is independent of reverse transcriptase and protease enzymes. Raltegravir was the first integrase inhibitor to complete phase III studies, initially in triple-class drug resistant patients[9], and more recently in naïve patients[10], and is now licensed in the US and Europe for use in ART-naive and experienced adults and adolescents. In December 2011, it received FDA approval for use in children aged 2-12 years. Raltegravir has been found to be highly active and well tolerated and is taken twice daily. There is also evidence that the novel mechanism of action may fundamentally alter early viral kinetics following initiation of ART, with a more rapid fall of viral load than with the other classes of

antiretroviral drugs. Its excellent efficacy and more rapid rate of achieving undetectable viral load compared with efavirenz[11] has resulted in it being advocated as a first-line combination in the current US adult guidelines[12]. An appropriate formulation is also safe, well tolerated and effective in children 6 years and older[13]; studies in children <6 years have recently completed[14].

Our hypothesis is that the high viral load *per se* in those initiating ART with low CD4 contributes to early mortality. Adding a fourth drug from this different integrase inhibitor class to triple ART for the first 12 weeks would therefore be likely to control viral replication more rapidly than standard triple ART, and reduction of early mortality/morbidity might follow. This approach would also minimise emergence of drug resistance and safeguard against consequences of adherence lapses at this early critical stage. Conversely, using an additional boosted protease inhibitor (bPI) as an alternative to an integrase inhibitor as a fourth drug would be unlikely to be beneficial as it has been demonstrated that bPI and non-nucleoside reverse transcriptase inhibitor (NNRTI) drugs both reduce viral load at similar rates - or if anything, that NNRTIs reduce viral load faster than bPIs[15].

1.2.2 AUGMENTED PROPHYLAXIS AGAINST OPPORTUNISTIC INFECTIONS (OIS), BACTERIAL INFECTIONS AND HELMINTHS

Key causes of mortality/morbidity in the initial period after starting ART in resource-limited settings are bacterial infections (most common), TB, oesophageal candidiasis and (in adults/older children) cryptococcal disease[16, 17]. All patients should receive cotrimoxazole prophylaxis with ART[18, 19]. However, as patients are already initiating at least 3 antiretroviral drugs, each with a risk of side-effects, the question is whether starting oral azithromycin daily for 5 days, immediate prophylaxis with isoniazid, fluconazole as well as cotrimoxazole at ART initiation, in those not already receiving these drugs, could decrease early mortality/morbidity without increasing toxicity, compared to giving cotrimoxazole alone initially, with isoniazid prophylaxis started 12 weeks later.

1.2.2.A Isoniazid

Active TB is a major contributor to morbidity and mortality in HIV-infected individuals in Africa[20]. Several randomised trials and a systematic review[21] have shown that 6 months of daily or twice-weekly isoniazid prophylaxis significantly reduces the short-term risk of active TB in adults with positive tuberculin skin tests (TST). Similar reductions in mortality and active TB (regardless of TST test positivity) have also been observed in South African children[22]. Despite the robust evidence, isoniazid prophylaxis is poorly implemented even where the trials were done (e.g. Uganda[23], Zambia[24]). Reasons include concerns about under-treating undiagnosed active TB and hence theoretically encouraging isoniazid resistance (refuted in the recent Botswana trial[25]), logistical barriers to administering TST at ART initiation (which are anyway difficult to interpret in the context of advanced immunosuppression and previous BCG vaccination), concerns regarding overlapping drug toxicities and poor coordination between TB and HIV programmes. Furthermore, the optimal timing of isoniazid initiation in severely immunocompromised and symptomatic patients starting ART has not been studied. As the goal of REALITY is to identify effective and safe interventions to reduce early mortality in settings where ascertaining TB diagnosis is challenging and could delay lifesaving interventions, we consider that immediate isoniazid prophylaxis is a potentially ethical and appropriate intervention which could decrease the risk of TB disease. Delaying prophylaxis because of concerns about isoniazid resistance in those who may already have TB has recently been challenged, and doing additional X-rays to diagnose TB before initiation of prophylaxis was shown not to be cost-effective[25]. However, a potential risk of initiating this drug at the same time as effective ART is increased toxicity, particularly as antiretroviral drugs themselves have known toxicities.

Whilst some have suggested that isoniazid prophylaxis is effective only in TST positive individuals, there is no definitive evidence for complete absence of effect in TST-negative individuals. For example, in the Botswana trial[26], although not statistically significant in an under-powered within-subgroup analysis, data suggested that efficacy of isoniazid prophylaxis in TST negative individuals could be as high as 25%. Efficacy might be even higher in HIV-infected individuals with low CD4 counts where interpretation of TST is much less clear and false negative rates are likely to be much higher. Doing TST on all patients is not a practical approach, as stated in WHO guidelines. The same Botswana trial also demonstrated conclusively that enhanced and complex TB screening before initiation of isoniazid prophylaxis did not reduce TB and TB-associated deaths compared to a simple symptom screen[25]. Data to support these findings in children are still weak but a pragmatic approach was taken to harmonise adult and paediatric algorithms for TB screening[27]. Thus, in summary, evidence to date suggests that providing isoniazid prophylaxis to HIV-infected individuals with low CD4 counts, regardless of TST status, and having excluded active TB on the basis of a simple symptom screen is a reasonable approach for both children and adults. The symptom-based screening tools used in REALITY will follow those already in routine use in each participating centre.

1.2.2.B Fluconazole

The prevalence of HIV-associated cryptococcal disease varies widely from ~2%-5% in high-income settings to up to 38% in some African countries[28]; cryptococcal disease is a significant cause of mortality/morbidity in African adults and older children initiating ART, particularly in advanced HIV disease. Primary antifungal prophylaxis with itraconazole or fluconazole reduces cryptococcal disease in HIV-infected adults[29] and a recent randomised controlled trial in Uganda found fluconazole prophylaxis was safe and effective against cryptococcal disease in a mix of HIV-infected adults before and on ART[30]. However, this has not been widely implemented, and toxicity could be greater specifically if started at the same time as ART. In children, fluconazole is widely used for candida infections but there are few data on use as prophylaxis for cryptococcal disease, which is seen clinically in older children. Primary fluconazole prophylaxis would also decrease occurrence of early oral/oesophageal candidiasis after ART initiation which is likely to impact ART adherence (occurring in ~11% of both adults in the DART trial and children in the ARROW trial within 24 weeks of ART initiation). In addition, both cryptococcal disease and candida oeseophagitis are among the most common causes of IRIS following ART intiation[31].

1.2.2.C Azithromycin

Sepsis is the commonest cause of early mortality after ART initiation. For example, among ~100 deaths in the first 90 days on ART in DART (median CD4 86 cells/mm^3), ~10% were due to TB, ~15% to cryptococcal meningitis and >30% attributable by independent endpoint review to sepsis/pneumonia; patterns were similar in the paediatric ARROW trial, with even more sepsis (despite concurrent cotrimoxaozole prophylaxis in all children). Blood culture data are frequently lacking or unreliable in Africa; S. pneumoniae, S. aureus, non-typhi salmonella and other gram negative organisms have all been reported, varying by country. Azithromycin is a broad spectrum antibiotic, which is taken once daily and is available in generic formulations for adults and children. It has been reported to have anti-inflammatory activity[32] and also halved all-cause one-year mortality in children aged 1-9 years in a community mass treatment trial for trachoma control in Ethiopia after only one dose[33]. A 5-day oral course would be likely to adequately treat bacterial infections and minimise risk of resistance.

One of the advantages of azithromycin over other macrolides such as clarithromycin and erythromycin is its lack of clinically relevant drug interactions, since, unlike these drugs, it does not affect the CYP3A4 cytochrome. Almost all studies specifically looking at drug-drug interactions have failed to show any effect of azithromycin on the pharmacokinetics (PK) of other drugs, or vice versa.

Sometimes small changes in levels have been noted, but these are judged more likely to be caused by intrasubject variability than by a true drug-drug interaction. Whilst some case reports have suggested drug-drug interactions, these have never been confirmed to be caused by a genuine PK interaction[34]. Alternatives such as ceftriaxone are only available as intravenous or intramuscular injections, quickly promote resistance and do not have anti-inflammatory activity, which may plausibly be an important mode of action in this trial.

1.2.2.D Albendazole

Helminth infections may adversely affect HIV-1 disease progression for two main reasons: firstly because they impair appetite and cause malnutrition which may act synergistically with underlying HIV disease, and second because they are likely to increase underlying immune activation and microbial translocation[35]. Beneficial effects of helminth eradication on HIV-1 RNA and/or CD4 counts have been shown in 3 randomised controlled trials[36-40]. However, anti-helminthics are not routinely given at ART initiation in either adults or children and standard practice varies widely across Africa.

1.2.2.E Prophylaxis Summary

All these prophylactic drugs are in widespread use across the world, and all have been shown to offer benefit as prophylaxis or treatment in specific circumstances. However, the question here is whether a unified strategic approach to their combined use at the same time as ART initiation in severely immuno-compromised patients with HIV-infection reduces mortality. Both the potential risks (in terms of increased toxicity, pill burden, poor adherence and, for TB, resistance) and the potential benefits (in terms of efficacy in reducing mortality and morbidity during the time of maximum risk after ART initiation) from universal wide prophylaxis may be greatest at the time of ART initiation. In contrast, starting more limited prophylaxis slightly later, eg 12 weeks after ART initiation, might be a safer strategy and could still provide benefit.

1.2.3 Macronutrient intervention

Higher mortality after starting ART occurs in individuals with lower BMI[41, 42], even outside levels indicating moderate-severe malnutrition. Intervention with a high calorie supplement (in addition to standard therapeutic interventions for individuals with severe malnutrition) could therefore potentially decrease mortality in patients starting ART with low CD4 counts, even in the absence of severe malnutrition[43]. This approach could be important for drug absorption, which is known to be impaired in severe HIV disease. Ready-to-Use high energy, low protein therapeutic Food (RUTF) is a lipid-based paste which does not require cooking. It was originally manufactured in France using peanut base, but is now made by generic companies, including in Africa, with other ingredients such as maize-soya-sorghum. However RUTF is often only recommended in guidelines for patients with severe malnutrition. A randomised trial of 491 adults with BMI <18.5 (severe malnutrition) starting ART demonstrated more weight, BMI, and CD4 gain with RUTF and soya food fortified with micronutrients at 3 but not 6 months[44], with no significant effect on mortality; similar effects have been described in patients with TB[28, 45]. Although power to detect mortality differences was very low (see sample size calculation in Section 9.3 - Sample Size, p70), these studies clearly identify that RUTF can increase weight. Further research is important because provision of additional energy in this way may increase fat instead of lean body mass, and could theoretically be counter-productive. RUTF is already widely used, despite lack of evidence even for patients with severe malnutrition. Whilst RUTF contains high levels of potassium to address underlying deficiences in severe malnutrition, the same base high-energy low-protein paste can be used as ready to use **supplementary** food (RUSF) in a wider population.

A further issue is that many patients report acute hunger after starting ART, and may be unable to increase their calorie intake[30, 46]. This may reflect a profound catabolic state induced by severe HIV

infection, and reversed with ART. Providing short-term RUSF may be a mechanism for achieving global well-being, and could also potentially improve adherence, thus improving outcomes.

1.2.4 Relevant studies underway or planned

Several studies in resource-rich countries have investigated the impact of additional raltegravir as intensification of ongoing current therapy and have observed little impact on low level viraemia[47, 48], but no studies have yet reported effects of intensified therapy at ART initiation. Furthermore, mortality rates are extremely low in these settings, as expected; no trial has examined the effect of this approach on morbidity/mortality (ClinicalTrials.gov, controlled-trials.com Jan 2011). For enhanced prophylaxis, trials have demonstrated the utility of individual drugs (see Section 1.2.2 above), but the question to be addressed in this trial is whether benefits from starting broad prophylaxis at the same time as ART outweigh the potential risks of starting several drugs together. One recently started ACTG study (NCT01380080: The REMEMBER trial - Reducing Early Mortality & Morbidity by Empiric Tuberculosis (TB) Treatment) is investigating the use of empiric TB treatment (rifampicin/isoniazid/pyrazinamide/ethambutol then rifampicin/isoniazid) in HIV-infected adults and adolescents initiating ART with CD4 <50 cells/mm^3 (results expected 2015). This is a complementary approach to the one proposed here. One ongoing trial (ISRCTN04084517) is investigating the impact of 3 months of Ready-to-Use Therapeutic food (RUTF) within 2 weeks of ART initiation on lipid profiles in 168 adults; our much larger trial in adults and children would provide power to demonstrate an impact on mortality. We are unaware of any trial powered to address mortality reduction in resource-limited settings as the primary endpoint, using a combined approach to assessing the different interventions above.

1.3 RATIONALE FOR THE TRIAL DESIGN

Whilst WHO guidelines now advocate earlier initiation of ART in HIV-infected adults and children 5 years or older (at <350 rather than <200 cells/mm^3 in WHO 2010 guidelines[49], and more recently at <500 cells/mm3 in WHO 2013 guideline[82]), in clinical practice, substantial numbers continue to present for care late in HIV infection - in both resource-rich and resource-limited settings. Given experience in resource-rich countries, this will continue to be a feature of ART rollout programmes for the forseeable future. However, the consequences of such late presentation, in terms of early morbidity/mortality on ART, are far more severe in resource-limited settings. To maximise benefits of ART, it is essential to identify which interventions, if any, in addition to ART could reduce high early death rates. Data from both adults and older children demonstrate the highest early mortality in those initiating ART with CD4 cell counts <50 cells/mm^3 (see Section 1.1 above): early mortality rates approximately halve in those with CD4 cell counts 50-99 cells/mm^3 and halve again with pre-ART CD4 cell counts >100 cells/mm^3. Thus those with CD4 <100 cells/mm^3 have substantial risks, and present in sufficient numbers, that interventions of proven benefit in this group could rapidly improve ART outcomes.

Results of studies on early response to ART and cause of early deaths do not identify a single plausible mechanism and thus there is no identifiable single most plausible intervention to evaluate in a traditional two-group randomised controlled trial. By including three different interventions in one factorial trial, and including adults and older children, REALITY will provide a comprehensive pragmatic assessment of the most cost-efficient way to save lives, and will be able to identify a highly effective integrated care “bundle”. Each intervention targets a different mechanism, so interaction - the effect of one intervention varying according to receipt or not of a second intervention - is less likely. It is thus more efficient to run one large factorial trial addressing all 3 questions rather than 3 separate trials addressing each individually. Furthermore, if an unanticipated interaction between

the interventions does exist, a factorial design allows such interactions to be identified and the relative contribution of each to be explored. This is more difficult (or impossible) in a single comparison trial where the effect modifier will have been used at the discretion of the treating physician, and will likely depend on patient characteristics. The randomisation will be open because of logistical constraints with multiple different pills and modalities (drugs and trial RUSF), and because a further key objective is to identify the acceptability of enhanced interventions around ART initiation which would not be possible with use of dummy pills.

The proposed trial design is pragmatic, aiming to recruit those as typical as possible of future patients, and treat them with practical interventions which could be implemented in ART programs in Africa. Exclusion of active TB will be based on symptom screening, as the feasibility of widespread implemention of molecular methods for TB diagnosis such as GeneXpert, is unclear particularly in rural areas, and it is essential not to do this so vigorously within the trial so as to jeopardise generalisability of results outside the trial. Inclusion criteria are broad, further increasing generalisability, and prophylaxis and nutritional control groups are based on standard of care in national guidelines, meaning that policymakers will be able to directly apply trial results to their current situation. The primary outcome measure is all-cause mortality, arguably the most important outcome for patients, their families and ART programs. Numerous studies in both adults and children have shown that mortality rates are far higher during the first few months on ART; if additional interventions can help patients to survive this period, they have a good chance of doing well on long-term ART. The use of centres which are strongly allied with treatment provision as well as research, and the involvement of representatives of Ministries of Health and patient representatives on the Trial Steering Committee, will ensure continued relevance to general clinical practice.

Although paediatric and adult trials have generally been run separately, a trial addressing issues at ART initiation provides a real opportunity for including both for the following reasons:

1. There is no evidence of heterogeneity in early mortality at low CD4 in the DART and ARROW trials (see Section 1.1 above): high early mortality is an equal problem in adults and children initiating ART with low CD4 counts.
2. Substantial heterogeneity between adults and children in response to the interventions proposed is not biologically plausible.
3. The predictive value of CD4 for subsequent disease progression/death is similar in untreated children aged 5 and over compared to adults, and the pattern of events is generally similar (including occurrence of cryptococcosis in older children); TB is a parallel major problem in adults and children.
4. Malnutrition is a major issue across all ages; whilst management of acute malnutrition is different in children, current WHO guidelines recommend treating this before starting ART; children would only be included in this trial after severe acute malnutrition has been treated.
5. ART programmes in Africa are increasingly treating adults and children in combined family clinics. **Integrating research as well as clinical care will provide the most relevant results for future patients.**
6. Finally, children have the same rights to benefit from research as adults - treatment of children in many diseases lags far behind adults because of absence of evidence supporting their treatment. Research is a major driver towards improvements in clinical practice: rather than asking why children should be included, the question should be why should children not be included? This question is being asked in areas such as TB; e.g. the large TBTC26 trial evaluating rifapentine for TB prophylaxis has successfully enrolled both adults and children over 2 years of age and efficiently incorporated relevant and timely pharmacokinetic substudies in children[50]. **Family centred ART and care is a paradigm to promote; family-centred research - where**

scientific questions of importance to both adults and children are promoted together- should provide an evidence base to support this.

1.4 OBJECTIVES AND HYPOTHESES

The primary objective of the trial is to identify effective, safe and acceptable interventions to reduce early mortality (all-cause) in HIV-infected adults, adolescents, and older children (5 years or more) initiating ART. There are three hypotheses:

1. Addition of 12 weeks of an integrase inihibitor (raltegravir) to initial antiretroviral therapy will reduce all-cause mortality, compared to standard of care first-line 3-drug 2-class ART.
2. Addition of short-term enhanced opportunistic infection (OI) prophylaxis with immediate isoniazid/pyridoxine (and cotrimoxazole), 12 weeks fluconazole, plus single-dose albendazole and 5 days of azithromycin at ART initiation, will reduce all-cause mortality, compared to standard of care cotrimoxazole prophylaxis plus continuation of any pre-existing prophylaxis (ie only starting cotrimoxazole) for the first 12 weeks followed by isoniazid prophylaxis from 12 weeks onwards.
3. Supplementation with 12 weeks of Ready-to-Use Supplementary Food (RUSF) will reduce all-cause mortality, compared to standard of care nutritional support to those with poor nutritional status according to local guidelines.

Secondary objectives include:

- identifying the most cost-effective interventions to reduce early all-cause mortality, and assessing their budget impact
- identifying the mechanism of action of the most effective interventions through focussed investigations of viral load and resistance, molecular diagnostics, measures of immune activation, immune responses to pathogens, microbial translocation and enteropathy, and body composition. (See Trial Assessment Schedule page xiii for details of assays and where they would be performed.)
- identifying the most effective ways to promote acceptance of additional strategies in the community, particularly with regard to potential stigmatising impact of increased pill burden

2 SELECTION OF CENTRES AND CLINICIANS

Nine clinical centres caring for adults and children in four countries, will participate:

- Zimbabwe: University of Zimbabwe, Harare
- Uganda: Joint Clinical Research Centre (JCRC) Kampala plus JCRC satellites participating in other MRC CTU trials (eg EARNEST) at Gulu, Mbarara, Mbale and Fort Portal
- Malawi: Queen Elizabeth Central Hospital/Wellcome Trust Unit, College of Medicine, University of Malawi, Blantyre
- Kenya: the KEMRI-Wellcome Trust Centre for Geographic Medicine (Coast) Kilifi; and the Academic Model for the Prevention and Treatment of HIV/Aids (AMPATH) Centre at Moi Teaching Referral Hospital (MTRH).

These centres have been chosen on the basis of criteria below.

2.1 CENTRE/INVESTIGATOR INCLUSION CRITERIA

To participate in the trial, investigators and clinical centres must fulfil a set of basic criteria defined below.

2.1.1 Centre PI Qualifications & Agreements

1. The centre PI should be qualified by education, training, and experience to assume responsibility for the proper conduct of the trial at their centre and should provide evidence of such qualifications through an up-to-date curriculum vitae and/or other relevant documentation requested by the Sponsor, the REC, the IRB, and/or the regulatory authority(ies).

2. The centre PI should be aware of, and should comply with, the principles of ICH GCP and the applicable regulatory requirements. A record of GCP training should be accessible for all investigators at the centre.

3. The centre PI should permit monitoring and auditing by the Sponsor, and inspection by the appropriate regulatory authority(ies)

4. The centre PI should maintain a delegation log of appropriately-qualified persons to whom the investigator has delegated significant trial-related duties.

5. The centre PI should sign an investigator statement, which verifies that the centre is willing and able to comply with the requirements of the trial.

2.1.2 Adequate Resources

1. The centre PI should be able to demonstrate a potential for recruiting the required number of suitable subjects within the agreed recruitment period (that is, the centre provides ART to an existing cohort of adults and children, typically in joint family clinics where adults and children are seen together at the same locations).

2. The centre PI should have sufficient time to properly conduct and complete the trial within the agreed trial period.

3. The centre PI should have available an adequate number of qualified staff and adequate facilities for the foreseen duration of the trial to conduct the trial properly and safely.

4. The centre PI should ensure that all persons assisting with the trial are adequately informed about the protocol, the investigational product(s), and their trial-related duties and functions.

2.1.3 Centre Assessment

Each selected clinical trial centre must provide a completed Investigator Statement, Signature and Delegation of Responsibilities Log, and staff contact details. The Investigator Statement verifies that the centre is willing and able to comply with the requirements of the trial. This will be signed by the Principal Investigator at the centre. In addition, and in compliance with the principles of ICH GCP, all centre staff participating in the trial must complete the Signature and Delegation of Responsibilities Log and forward this to the MRC CTU. The MRC CTU must be notified of any changes to trial personnel and/or their responsibilities. An up-to-date copy of this log must be stored in the Trial Master File (TMF) at the centre and also at the MRC CTU.

2.2 APPROVAL AND ACTIVATION

On receipt of the above documents at the MRC CTU, written confirmation will be sent to the PI.

1. The centre should conduct the trial in compliance with the protocol as agreed by the Sponsor and, if required, by the regulatory authority(ies), and approved by the REC and/or IRB.

2. The centre PI or delegate should document and explain any deviation from the approved protocol, and communicate this with the trial team at the MRC CTU.

A list of activated centres may be obtained from the Trial Manager.

3 SELECTION OF PATIENTS

There will be **no exceptions** to eligibility requirements at the time of randomisation. Questions about eligibility criteria should be addressed prior to attempting to randomise participants.

The eligibility criteria for this trial have been carefully considered. The eligibility criteria are the standards used to ensure that only medically appropriate patients are considered for this study. Patients not meeting the criteria should not join the study. For the safety of the patients, as well as to ensure that the results of this study can be useful for making treatment decisions regarding other patients with similar diseases, it is important that no exceptions be made to these criteria for admission to the study.

Participants will be considered eligible for enrolment in this trial if they fulfil all the inclusion criteria and none of the exclusion criteria as defined below.

3.1 PATIENT INCLUSION CRITERIA

1. Aged 5 years or older
2. Documented HIV infection by HIV ELISA or HIV rapid test
3. Naive to ART
4. CD4 T-cell count <100 cells/mm^3 on blood test taken at screening for REALITY
5. Results of screening haematology and biochemistry tests available and no contraindications to planned ART according to national guidelines
6. Patient/carer provide informed consent (and children <18 years assent, as appropriate according to their age and knowledge of HIV status)

The lower age limit is because CD4 counts are less reliable predictors of immunodeficiency under 5 years: CD4 counts are recommended by guidelines in older children[51].

No patient with a CD4 count above 100 cells/mm^3 should have ART delayed in order to subsequently meet eligibility criteria. Rather, patients eligible for REALITY will be those testing HIV positive for the first time with a low CD4 count (i.e. those delaying presentation to care), or those who have defaulted before initiating ART and only return to care at an advanced stage of immuno-deficiency.

3.2 PATIENT EXCLUSION CRITERIA

7. Contraindications to any proposed antiretroviral drugs (including integrase inhibitors), isoniazid, fluconazole, albendazole, azithromycin or trial RUSF
8. Pregnant or breastfeeding or intending to become pregnant during the first 12 weeks of the study
9. Ever **known** to have previously received single-dose nevirapine for prevention of mother-to-child transmission (mother or child).

Patients with acute illnesses but whose clinicians judge that – for their own health – it is important for them to initiate ART now are eligible for REALITY, because it is precisely these patients who are at highest risk of early mortality on ART. These patients are eligible regardless of what other medications are being used to treat their underlying illness, provided there are no contraindications

to the study drugs ((7) above). **In particular, patients receiving isoniazid (alone or together with other anti-tuberculous drugs for the treatment of active tuberculosis), fluconazole or azithromycin for treatment of acute illnesses are eligible for randomisation in the trial** – they will continue pre-existing medications for treatment, and start any other prophylaxis drugs as randomised. Patients already receiving anti-tuberculous therapy who are randomized to receive isoniazid in REALITY will continue isoniazid once their TB treatment regimen finishes (if this occurs within 12 weeks of randomization). Patients receiving isoniazid preventative therapy as part of TB contact tracing are also eligible: they will continue pre-existing isoniazid for prophylaxis, and start any other prophylaxis drugs as randomised. Patients requiring therapeutic feeding for severe malnutrition would receive this, and start any other prophylaxis drugs as randomised. This reflects the fact that REALITY is a pragmatic strategy trial and that patients presenting sick receiving concomitant medications are the target population to which REALITY results must be generalisable.
Patients who are allergic to cotrimoxazole are eligible if a suitable alternative can be provided.

As women and older children will be presenting acutely with CD4<100 cells/mm3 it is extremely unlikely that any will have taken single dose nevirapine for the the prevention of mother-to-child transmission. However, as NNRTI-based regimens could have suboptimal efficacy, any such women and children who know they have received single-dose nevirapine will not be eligible. Any women or child who does not know whether or not they have received single-dose nevirapine is eligible.

On the screening CRF degree and frequency of alcohol consumption will be assertained. Clinicians should make a case-by-case assessment about the possibility of excess consumption and potential for poor trial compliance, and should not enrol any patients for whom they have significant concerns (as for other patient-specific characteristics). However, alcohol consumption per se is not an exclusion critieria.

3.3 NUMBER OF PATIENTS

1800 individuals will be enrolled over 18 months. The target is to enrol up to 600 children aged 5-12 years and at least 1200 adolescents and adults aged 13 years and older. A minimum of 400 and a maximum of 600 children aged 5-12 years will be enrolled to ensure that the trial has sufficient power to address the important question of growth in children, but also sufficient adolescents and adults for generalisability to other adult populations (see Section 9.3 - Sample Size, p70).

The aim is to recruit 450 individuals from each country (300 adults and 150 children). However, more patients may be recruited in some countries in order to achieve the overall recruitment target of 1800 individuals over 18 months, as recruitment may be more challenging in some sites than others.

3.4 SCREENING PROCEDURES

3.4.1 SCREENING VISIT

At screening, patients and carers of HIV-infected children and adolescents will be given an information sheet about the REALITY trial and asked to give written consent **for screening** (see Appendix I - Template Patient information sheets, p91; Appendix II - Screening and Trial Consent and Assent forms, p101). Children will also be given information and counselling appropriate to their age but will not be asked specifically to assent **to screening** as this involves only standard blood draw (as would happen anyway) and a routine examination (see Section 4.1 - Randomisation visit, p35 for

consent/assent to trial participation). Signed screening consent forms must be kept by the investigator in the Trial Master File and a copy given to the participant or family.

After consenting to screening, the HIV-infected adult, adolescent or child will have clinical information including medical history and assessment of alcohol consumption, examination including WHO staging of HIV infection (1, 2, 3, or 4), and weight/height/MUAC recorded, and blood taken for T-cell subsets (CD4, CD8, CD3 and total lymphocyte count), haematology and biochemistry (see Trial Assessment Schedule, page xiii). Individuals with clinical evidence of acute infections at screening will be investigated; all individuals will be screened for active tuberculosis using a symptom checklist, following those already in routine use in participating centres (eg in Kilifi the Kenyan MOH Symptom Screen for Tuberculosis will be used). A pregnancy test will be performed if considered appropriate by the trial clinician according to age and Tanner/menopausal status. Plasma and DNA pellets will be saved. Girls and women of reproductive age will be given information about the risks of pregnancy in the trial and encouraged to avoid pregnancy.

Patients presenting with acute illness and receiving treatment are eligible for inclusion if they are being considered for starting ART, even if they are also receiving treatment for one or more of the OIs (see Section 5.1.2 - Standard of care OI prophylaxis, p42).

4 RANDOMISATION

4.1 RANDOMISATION VISIT

As individuals joining REALITY need to start ART urgently because of their low CD4 cell counts, the interval between the screening and randomisation visits may be as little as 1 day, providing that all laboratory test results from the screening visit are available, and the participant/carer feels they have had adequate time to consider trial participation. Wherever possible the interval between screening and randomisation visits should be less than 4 weeks; it must be less than 6 weeks.

Timing of ART initiation with respect to onset of acute illness is not mandated by this protocol, but left to individual clinicians/centres. Whilst several trials (eg ACTG5164[52] amongst others) have demonstrated benefits from rapid ART initiation in those with severe immunodeficiency and acute opportunistic infections, clinicians will consider each patient's individual circumstances in accordance with local guidelines, whilst aiming to initiate ART as promptly as possible. In particular, individuals should receive whatever management is considered clinically necessary for malnutrition and other infections, but would be considered for REALITY at the point of ART initiation. To meet eligibility criteria at this timepoint, each possible intervention they could be randomised to in REALITY would need to be a reasonable clinical choice, subject to Section 3.2 p32 above .

The participant’s eligibility for enrolment will be confirmed, including the results of screening laboratory tests. Participants or carers must confirm that they have read the relevant patient information sheets. Written informed consent to enter into the trial and be randomised must then be obtained from participants or parents/guardians/person with legal responsibility (including legal authorities) for children, if appropriate, after explanation of the aims, methods, benefits and potential hazards of the trial and BEFORE any trial-specific procedures are performed or any blood is taken for the trial (see Appendix II - Screening and Trial Consent and Assent forms, p101). Children should give assent to trial particpation if they are older than 8 years and know their HIV status. Signed trial consent forms must be kept by the investigator and documented in the CRF and a copy given to the participant or family.

It must be made completely and unambiguously clear that the participant (or parent or guardian of a child) is free to refuse to participate in all or any aspect of the trial, at any time and for any reason, without incurring any penalty or affecting their treatment (or that of their child).

For children and adolescents aged under 18 years, the primary carer providing consent will be asked to nominate another one or two carers (depending upon local practice) who will be responsible for the child’s welfare in the event that they are unable to continue caring for them. Whilst the carer who consented is alive, counselling of any other person who brings the child to the REALITY clinic is necessary to explain the trial, but re-consent is not needed. If the carer who originally gave consent dies, it will be necessary to obtain re-consent from the next nominated primary carer.

After consenting to trial participation (day 0), enrolment assessments will be performed as summarised in the Trial Assessment Schedule, page xiii. Blood samples will be taken for lymphocyte subsets (CD4, CD8, CD3 and total lymphocyte count) and plasma and DNA pellets will be saved. When possible for children under 13 years, and particularly if there is doubt about the place of residence of a child and his/her family, a study nurse/field worker, preferably the same nurse who gave information about the trial to the carer/child, will accompany the trial participant to their home and draw a map indicating the place of residence.

A trial register will be kept at the clinical site and will record all persons who are eligible and invited to join the trial. Those accepting will have name, date of birth (DOB), randomisation date and trial number recorded. Those who refuse will have name, DOB, and reason for refusal recorded. The register will be kept in a secure place in each clinical site and will be the responsibility of the Principal investigator at that site.

4.2 RANDOMISATION PRACTICALITIES

The patient should be physically present together with a study clinician in either the centre clinic or a hospital ward at the time of randomisation.

Further details on the process of randomisation can be found in Section 9.1 - Method of Randomisation, p69. To randomise a patient the information contained on a completed randomisation CRF will be entered into the online trial database accessible from the local clinical sites that will automatically check for eligibility. Only those with completed and verified screening forms on the database will be able to be randomised. The generated randomisation lists will be securely incorporated within this web-enabled trial database, and allocation concealed until the point of the next randomisation. The details of the patient's treatment allocation and REALITY trial number will be notified onto to clinical staff, and the trial number and allocation cross checked between those randomising and those managing the patient clinically.

If the centre's internet connection is unavailable at the time of randomisation, the patient's details can be provided to staff at the MRC CTU by fax or phone. At the MRC, staff will verify eligibility and perform the randomisation using the online system. The details of the patient's treatment allocation and REALITY trial number will be notified to the trial team at the site by fax and by phone within one hour of the receipt of the randomisation form.

The patient's trial number, treatment allocations and the date of randomisation will be entered into the Trial Register at the centre. The clinician should complete a prescription with the patient's details and trial medications as allocated. The prescription will be for one month until the week 4 visit. The pharmacist or pharmacy technician should ensure that the patient knows how to take the different drugs before they leave the clinic.

4.2.1 ENROLMENT OF MULTIPLE PARTICIPANTS FROM THE SAME FAMILY INTO THE TRIAL

The duration of the intervention in REALITY is 12 weeks after which all participants will receive standard of care first-line ART. Therefore, if an eligibile patient is to be randomised when there is already another person (adult, adolescent or child) from the same household already randomised and still in the first 12 weeks of the trial follow-up, then the new household member will receive the same allocations for all three randomisations as the original household member, via a specific procedure in the electronic database. However, after the 12 week intervention phase, all patients receive standard of care ART and other treatment, and so multiple family members may be enrolled (just as multiple members would receive treatment at the same time from an ART programme). It must be fully explained to family/household members where more than one member is recruited into the trial after the 12 week intervention phase that different participants may be randomised to receive different interventions. Participants should all be counselled that they should on no account share their trial drugs with any other household member in REALITY or not in REALITY.

4.3 CO-ENROLMENT GUIDELINES

Patients will not ordinarily be permitted to participate in any other clinical intervention trial or research protocol while on the REALITY trial. Participation in other studies that do not involve an intervention may be acceptable, but should be discussed with the REALITY TMG via the MRC CTU. The REALITY TMG will consider co-enrolment of REALITY participants onto other trials where the interventions do not conflict with the REALITY objectives on a case-by-case basis.

5 TREATMENT OF PATIENTS

5.1 INTRODUCTION AND STANDARD OF CARE

All participants will receive standard of care (SOC) HIV treatment consisting of ART and immediate cotrimoxazole prophylaxis, together with isoniazid prophylaxis after 12 weeks where this is part of national guidelines. In addition participants will be randomised to receive or not receive each of the three additional interventions for 12 weeks, as detailed in Sections 5.2 to 5.4 below. Treatment will be open-label and will be distributed at 4 to 6 weekly intervals before 24 weeks (then 12-weekly) from a dedicated supply of study drugs for both SOC HIV treatment and the additional study interventions. These will be stored separately from routine clinic drug supplies in a designated section of the research pharmacy at the study site.

Standard of care HIV treatment includes the following three components.

5.1.1 STANDARD OF CARE ANTIRETROVIRAL THERAPY

All participants will receive ART including 1 non-nucleoside reverse transcriptase inhibitor (NNRTI) plus 2 nucleoside reverse transcriptase inhibitors (NRTIs) as first-line therapy following WHO recommendations[49, 51]. Across the countries in REALITY, standard of care first-line ART includes either nevirapine or efavirenz plus lamivudine plus either stavudine, zidovudine, abacavir (for children) or tenofovir (for adults/adolescents, and for children when licensed and appropriate formulations become available). **Stavudine-based regimens will not be used in initial first-line in REALITY.** (They may be used as a first-line substitution in children only where no other drugs are available, see Section 5.7.2 - ART drug substitutions, p49 below.) Choice of NNRTI will be made by the treating clinician.

As participants will move to national programmes after being followed for 48 weeks in REALITY, preferred standard of care ART in REALITY is based on what is being used in national programmes, as this will ensure that participants can stay on the same regimens long-term. Inclusion of different first-line regimens increases generalisability, and will form a pre-planned subgroup analysis population.

Table 5.1: Current first-line ART in adults, adolescents and children

COUNTRY	CURRENT FIRST-LINE IN ADULTS/ADOLESCENTS	CURRENT FIRST-LINE IN CHILDREN*
Kenya	ZDV/3TC/NNRTI or TDF/3TC/NNRTI or TDF/FTC/NNRTI	ABC/3TC/NNRTI or ZDV/3TC/NNRTI
Malawi	*(d4T/3TC/NNRTI**)* or TDF/3TC/NNRTI if already on TB treatment, and from 2012-2013	ZDV/3TC/NNRTI
Uganda	ZDV/3TC/NNRTI or TDF/FTC/NNRTI	ZDV/3TC/NNRTI
Zimbabwe	TDF/FTC/NNRTI	ZDV/3TC/NNRTI or TDF/3TC/NNRTI

ABC=abacavir, d4T=stavudine, 3TC=lamivudine, FTC=emcitrabine, TDF=tenofovir, ZDV=zidovudine, NNRTI=nevirapine or efavirenz. 3TC and FTC are considered exchangeable.
* any children aged 5 years or older exposed to single dose NVP are not eligible.
** Stavudine-based regimens are still included in current Malawi guidelines: tenofovir-based regimens will be provided for REALITY in Malawi.

These drugs will be provided by GlaxoSmithKline, Gilead Sciences and Cipla during the REALITY trial. For adults, recommended doses are

- tenofovir - 300 mg once daily

- emtricitabine - 200 mg once daily
- lamivudine - 150 mg twice daily or 300 mg once daily
- zidovudine - 300 mg twice daily
- nevirapine - 200 mg twice daily (after a 14 day induction phase of 200mg once daily)
- efavirenz - 600 mg once daily

For children, WHO recommended dosing tables will be used, except for efavirenz dosing in the 20-25kg weight band. The WHO 2010 dose for 20-25 kg (300mg daily) was evaluated in the ARROW PK sub-study Part 1 and was associated with substantial proportions failing to reach target AUC[53], and so is increased slightly in the dosing table below.

Table 5.2: Dosing table of first-line antiretroviral drugs to be used in REALITY (based on WHO Annex E[54] except for shaded boxes, see below)

DRUG	STRENGTH OF CHILD TAB (MG)	NUMBER OF TABLETS BY WEIGHT BAND (KG) ONCE DAILY (EFV); TWICE DAILY (ALL OTHER PRODUCTS)					STRENGTH OF ADULT TAB (MG)	NUMBER OF TABLETS BY WEIGHT BAND (KG). ONCE DAILY (EFV); TWICE DAILY (ALL OTHER PRODUCTS)	
		3.0-5.9	6.0-9.9	10.0-13.9	14.0-19.9	20.0-24.9		25.0-34.9	35+
ABC/3TC*	60/30 (Baby)	1	1.5	2	2.5	3			
ABC/3TC/NVP	60/30/50 (Baby)	1	1.5	2	2.5	3	300/150/200	1	1
ZDV/3TC	60/30 (Baby)	1	1.5	2	2.5	3			
ZDV/3TC/NVP	60/30/50 (Baby)	1	1.5	2	2.5	3			
D4T/3TC	6/30 (Baby)	1	1.5	2	2.5	3			
D4T/3TC/NVP	6/30/50 (Baby)	1	1.5	2	2.5	3			
EFV**	200			1	1.5	2			
EFV**	600			0.33	0.5	0.66**	600	0.66	1
NVP	50	1	1.5	2	2.5	3	200	1	1

* Both doses can also be taken together once daily

** Revised dosing follows WHO 2010 guidelines[54], except for the 20-25kg weight band. The WHO 2010 dose for 20-25 kg (300mg daily) was evaluated in the ARROW PK sub-study Part 1 and was associated with substantial proportions failing to reach target AUC[53], and so is increased slightly in the above.

Note: where fixed dose combination products can be split then an equal dose morning and evening should be given. If the fixed dose combination cannot be split then the larger dose should be given in the morning.

Note: the entire table of doses from WHO Annex E is included[54], although it would be very unusual for a 5 year old to weigh under 6kg. Stavudine (d4T) may be used as a substitution in children where no other drug option is available.

Table 5.3: Dosing table for Tenofvoir to be used in REALITY (Zimbabwe only)

BODY WEIGHT (KG)	NUMBER OF TABLETS ONCE DAILY
17 to <22	1 x 150 mg
22 to < 28	1 x 200 mg
28 to < 35	1 x 250 mg
≥35	2 x 150 mg

5.1.2 STANDARD OF CARE OI PROPHYLAXIS

All participants not already receiving cotrimoxazole prophylaxis will be prescribed cotrimoxazole prophylaxis at screening, or at randomisation at the very latest. 960mg trimethropim(160mg)/sulfamethoxazole(800mg) once daily will be prescribed to adults and adolescents aged 13 years or older, and 480mg trimethropim(80mg)/sulfamethoxazole(400mg) to children aged 5-12 years.

Isoniazid prophylaxis has been demonstrated to have benefit in HIV-infected patients, but has been variably included in national guidelines and is variably used within programmes even when national guidelines suggest it could be used. In centres in countries where isoniazid is being used in participants on ART in the national programme, all REALITY participants will be prescribed isoniazid prophylaxis from 12 weeks after initiating ART. 300mg isoniazid+25mg pyridoxine will be prescribed to adults and adolescents aged 13 years or older, and 150mg isoniazid+12.5mg pyridoxine to children aged 5-12 years. Although isoniazid prophylaxis is recommended by WHO, it is currently not being used routinely from ART initiation in the proposed centres, and there is a specific question about starting it for the first-time **at the same time as initiating ART** - this is the question addressed by the randomisation below. In centres where isoniazid is not being routinely used in participants on ART, REALITY participants randomised to standard of care OI prophylaxis will receive cotrimoxazole alone. This is to ensure consistency between standard of care in the local centre and the standard of care OI prophylaxis group. Where isoniazid prophylaxis is being taken as standard of care, REALITY participants will take it continuously for the duration of REALITY once they have started it, unless national or local guidelines recommend a maximum duration (eg 9 months in Eldoret). Thus the randomised question addressed in the trial will be "should isoniazid be initiated at the same time as ART in highly immunocompromised patients starting ART or not", otherwise following national/local guidelines for use of isoniazid preventative therapy.

In addition, all participants will continue any pre-existing prophylaxis **or treatment** that was already being prescribed at the screening visit, for example

- Those already receiving tuberculosis treatment and courses of antibiotics for lung disease at randomisation would continue these regardless of randomised allocation. If randomised to enhanced OI prophylaxis they would receive additional fluconazole, azithromycin and albendazole from ART initiation. If their tuberculosis treatment stopped before 12 weeks in REALITY, they would continue with isoniazid prophylaxis from this timepoint. If their tuberculosis treatment stopped after 12 weeks in REALITY, they would receive isoniazid prophylaxis if standard of care in the local centre.
- Those already receiving fluconazole for secondary cryptococcal prophylaxis at randomisation would continue to receive this throughout trial participation, regardless of randomised allocation. If randomised to enhanced OI prophylaxis they would receive additional isoniazid, azithromycin and albendazole from ART initiation, and would continue fluconazole after 12 weeks in REALITY.
- Those already receiving isoniazid prophylaxis, for example due to TB case contact would continue these regardless of randomised allocation. If randomised to enhanced OI prophylaxis they would receive additional fluconazole, azithromycin and albendazole from ART initiation.

That is, patients already receiving treatment or prophylaxis for TB or cryptococcal disease or azithromycin for treatment of sepsis can be enrolled but would continue their pre-existing treatment/prophylaxis, and only receive other prophylaxis according to randomisation.

Furthermore, any patient who becomes eligible for immediate isoniazid prophylaxis after trial recruitment, for example, if another household contact is diagnosed with tuberculosis, would start it immediately and not wait until 12 weeks.

It is also important to note that in Malawi, patients being followed in pre-ART clinics will routinely receive isoniazid prophylaxis pre-ART. However, these patients are extremely unlikely to be eligible for REALITY as they will have been being followed with high CD4, whereas REALITY participants will present newly HIV-diagnosed with advanced immuno-deficiency (CD4<100 cells/mm^3).

Following national guidelines, all children will receive routine de-worming at 24 and 48 weeks post-enrolment irrespective of randomisation.

5.1.3 Standard of care nutritional support

Standard of care nutritional support will follow current local practice in each centre (and centre will be a planned subgroup analysis). Adults and children with mild-moderate malnutrition do not currently receive any nutritional support following national guidelines, except in Kilifi where those with BMI<18.5 receive Plumpy Soy or Acha Mum (2 sachets daily). Management of adults with severe malnutrition initiating ART varies across centres: most are provided with micronutrients and advice on food. Additional RUTF is provided to severely malnourished adults with BMI<16-18 or MUAC<16cm in some centres (see table below for current practice). Severely malnourished children with weight-for-height z-score <-3 (or weight-for-height<70-80% expected), or low MUAC, severe visible wasting or bilateral pitting oedema are generally stablised during hospital admission if they have no appetite, or medical complications; they receive therapeutic feeding with milk feeds and then RUTF on discharge. Severely malnourished children who have good appetite and are clinically stable are generally treated as outpatients with Ready to Use **Therapeutic** Food (RUTF). ART is generally started once the child is clinically stable. Standard of care includes prescription of multi-vitamin multi-mineral (MVMM) supplements only for children starting ART in the JCRC Kampala site (not adults or adolescents); and food support and nutritional supplements for 6 months for adults and children in need of these (eg due to food insecurity) in Eldoret.

In this trial we will not mandate timing of ART initiation in those with severe malnutrition, but will leave this to individual clinicians/centres. Individuals should receive whatever management is considered clinically necessary for malnutrition (and other infections), but would be considered for the trial at the point of ART initiation.

Any adult, adolescent or child requiring therapeutic feeding according to the criteria below would receive this regardless of randomisation (estimated <5%). Randomisation will be stratified by centre, so that minor differences in eligibility criteria for therapeutic feeding will not affect the comparison of randomised groups. Participants eligible for or receiving food products from national programmes would transition onto the study product when they finish it if randomised to the enhanced nutritional support and still <12 weeks from randomisation.

Table 5.4: Eligibility for therapeutic feeding

Country	Adults/adolescents: current eligibility for therapeutic feeding	Children: current eligibility for therapeutic feeding in children aged 5 years or older
Kenya	Kilifi: BMI<16<br>Eldoret: BMI<16 or MUAC<16cm	weight-for-height z-score<-3
Malawi	BMI<17	MUAC<12cm, weight<80% expected for height

Uganda	BMI<18	Weight for height < 70% for children of all ages<br>MUAC <11.5 cm for children 6months to <6yrs<br>MUAC <13.5 cm for children 6yrs to <10yrs<br>MUAC <15.7 cm for children 10yrs and older
Zimbabwe	BMI<17	BMI for age z-score<-3

5.2 RANDOMISATION A: +/- 12 WEEKS INDUCTION WITH AN INTEGRASE INHIBITOR

RANDOMISATION A 1:1 to

- Raltegravir (integrase inhibitor) BD for 12 weeks from ART initiation in addition to 3 standard ARVs OR
- 3 standard ARVs only

5.2.1 PRODUCTS AND DOSING SCHEDULE

Raltegravir will be provided as trial supplies by Merck, and will be taken orally twice daily for 12 weeks. Dosing is as follows

- adults: 400mg twice daily (film coated tablet)
- adolescents 12-18 years[55]: 400mg twice daily (film coated tablet)
- children 6-11 years weighing ≥25 kg[56]: 400mg twice daily (film coated tablet)
- children 5-11 years[14, 57]: 6 mg/kg twice daily (chewable tablet, see below)

Both the film-coated tablet and the chewable tablet are licensed for administration to HIV-infected adults, adolescents and children in combination with other antiretrovirals. However, the chewable tablet and the film-coated tablet formulations are not bioequivalent. Paediatric dosing of chewable tablets is:

BODY WEIGHT (KG)	DOSE	NUMBER OF CHEWABLE 100MG TABLETS	
		AM	PM
10 to <14kg	75 mg twice daily	0.5	1
14 to <20kg	100 mg twice daily	1	1
20 to <28kg	150 mg twice daily	1.5	1.5
28 to <40kg	200 mg twice daily	2	2
40+kg	300 mg twice daily	3	3

The 100 mg chewable tablet can be divided into equal halves.

5.2.2 RALTEGRAVIR DOSE MODIFICATIONS, INTERRUPTIONS & DISCONTINUATIONS

Raltegravir should be permanently discontinued following a grade 4 adverse reaction judged possibly, probably or definitely related to raltegravir. For other toxicity, raltegravir may be interrupted and restarted as for other antiretrovirals (see Section 5.7 - Dose Modifications, Interruptions & Discontinuations of standard ART and cotrimoxazole or isoniazid prophylaxis, p47). If raltegravir is interrupted during the first 12 weeks on ART, then it should still be stopped permanently at the week 12 visit, because the hypothesis concerning the effect of raltegravir on early mortality is related to the number of weeks since ART initiation, not the total duration of raltegravir-containing ART.

Raltegravir should be given at full dose (adults: 400mg BD, children as above) or not at all. Due to the potential for increased development of resistance, dose reduction should not occur. If raltegravir is stopped at the same time as all other ART drugs (which will include an NNRTI), then a 10-14 day NRTI "tail" should be given wherever possible (see Section 5.7 - Dose Modifications, Interruptions & Discontinuations of standard ART and cotrimoxazole or isoniazid prophylaxis, p47).

5.3 RANDOMISATION B: +/- 12 WEEKS ENHANCED OPPORTUNISTIC INFECTION (OI) PROPHYLAXIS

RANDOMISATION B 1:1 to

- immediate isoniazid/pyridoxine and cotrimoxazole, plus 12 weeks fluconazole, 5 days azithromycin and a single dose of albendazole OR
- immediate cotrimoxazole (if not already taking this) in all patients, plus isoniazid/pyridoxine after 12 weeks in centres where this is current standard of care following national guidelines (together with any prophylaxis and/or treatment prescribed at screening)

5.3.1 PRODUCTS AND DOSING SCHEDULE

Isoniazid, cotrimoxazole, fluconazole, azithromycin and albendazole will be provided by Cipla, and are all taken orally once daily.

The aim would be to use a fixed dose combination (FDC) scored tablet of cotrimoxazole/isoniazid/pyridoxine to provide correct dosing to adults and older children, to reduce pill burden and ensure all drugs are taken, as well as simplify administration, procurement and production for post-trial uptake. The formulation would be a scored whole tablet for adults and adolescents aged 13 years or more, and half a tablet for children age 5-12 years, taken continuously from randomisation.

- 960mg trimethropim(160mg)/sulfamethoxazole(800mg) + 300mg isoniazid+ 25mg pyridoxine (co-formulated or co-packaged) plus 100mg fluconazole for adults
- 480mg trimethoprim(80mg)/sulfamethoxazole(400mg)+ 150mg isoniazid + 12.5mg pyridoxine plus 50mg fluconazole for children aged 5-12 years (1/2 tablet daily).

This co-formulated pill is currently being evaluated in a bioequivalence study in healthy volunteers against the individual components taken simultaneously, in a collaboration between WHO and Cipla with the eventual goal of WHO pre-qualification/FDA licensing. Providing results from this study demonstrate bioequivalence, then the co-formulated tablets would be used in REALITY. Until this timepoint, Cipla have agreed to provide appropriate simple co-packaging for the trial initially to make taking multiple pills easier. In centres where isoniazid prophylaxis is not being routinely used in patients initiating ART, single scored tablets of 960mg trimethropim(160mg)/ sulfamethoxazole(800mg) will be provided for use from 12 weeks after randomisation in the intervention group (and for those randomised to standard of care).

For fluconazole, adults and adolescents aged 13 years and older would take a whole 100mg fluconazole tablet once daily for 12 weeks, and children aged 5-12 years would take 50mg as half a (scored) tablet for 12 weeks.

For azithromycin, adults and adolescents would take a whole 500 mg tablet once daily for 5 days starting at randomisation, and children aged 5-12 years would take 250mg as half a (scored) tablet for 5 days starting at randomisation.

A single dose of 400mg albendazole will be given to adults and children 5 years and older as a 400mg tablet on the day of randomisation.

5.3.2 Fluconazole and azithromycin dose modifications, interruptions & discontinuations

Fluconazole should be permanently discontinued following a grade 4 adverse reaction judged possibly, probably or definitely related to fluconazole. Azithromycin should be permanently discontinued following a grade 4 adverse reaction judged possibly, probably or definitely related to azithromycin. For other toxicity, fluconazole and/or azithromycin may be interrupted and restarted (following the principles in Section 5.7 - Dose Modifications, Interruptions & Discontinuations of standard ART and cotrimoxazole or isoniazid prophylaxis, p47). If fluconazole is interrupted during the first 12 weeks on ART, then it should still be stopped permanently at the week 12 visit, because the hypothesis concerning the effect of enhanced OI prophylaxis on early mortality is related to weeks since ART initiation, not the total duration of fluconazole prophylaxis.

See Section 5.7 - Dose Modifications, Interruptions & Discontinuations of standard ART and cotrimoxazole or isoniazid prophylaxis, p47, below.

5.4 RANDOMISATION C: +/- NUTRITIONAL SUPPORT

RANDOMISATION C 1:1 to

- Supplementation with trial ready-to-use supplementary food (RUSF) for 12 weeks OR
- Supplementation for those with severe malnutrion as local practice

5.4.1 Products

Ready-to-Use high energy, low protein Food is a lipid-based paste which does not require cooking. It was originally manufactured in France using peanut base, but is now made by generic companies, including in Africa, with other ingredients such as maize-soya-sorghum. In REALITY, the Ready-to-Use high energy, low protein **Supplementary** Food (RUSF) intervention will be based on this paste with the goal of providing an additional 1000 kCal daily in adults and adolescents (2 foil packets daily; 2x92g), and an additional 500 kCal daily in children (1 foil packet daily; 92g). It will be fortified with multi-vitamins and multi-minerals (approx 1xRecommended Daily Allowance (RDA)), so that centres which provide these routinely to children (JCRC Kampala) will not need to provide additional tablets for the 12 weeks in which trial RUSF is used. The trial RUSF will be provided by Valid International.

5.5 DISPENSING

Throughout the trial adults, adolescents and children or their carers will be provided with a supply of drugs sufficient to last until their next clinic visit and will be requested to return all empty bottles and to bring any bottles in use or unused to the follow-up clinic (for ART and prophylaxis drugs). Drugs will be provided for the trial as tablets and scored tablets, and trial RUSF as foil packets (which should also be returned to clinic if unused).

On no account should any drug assigned to a patient be used by anyone else. Unused drug must be returned to the site if a patient withdraws from treatment.

All drug dispensed and returned to the site should be documented on a treatment log for each patient. At each site, a named person (trial pharmacist or research nurse) will be required to maintain complete records of all study medication dispensed. For all REALITY trial drugs (ARVs, prophylaxis,trial RUSF), the designated trial pharmacist/nurse will, on receipt of supplies prior to the commencement of the trial, conduct an inventory and complete a receipt. All trial drugs dispensed to participants will be recorded on a Dispensing Log. Inventories will be conducted monthly, and logs returned to MRC CTU.

Procedures for drug shipping, labelling, accountability and destruction will be detailed in the REALITY Pharmacy Manual of Operations. MRC CTU will monitor drug accountability at site visits.

5.6 OVERDOSE OF TRIAL MEDICATION

All participants should be counselled about the importance of taking the medications as prescribed. It is particularly important that participants understand how many pills of which type they should be taking. Participants must be told to come to the clinic immediately if they take too many pills. As no specific adverse consequences of overdose with any of the trial medications has been described, patients will be managed on a case by case basis.

5.7 DOSE MODIFICATIONS, INTERRUPTIONS & DISCONTINUATIONS OF STANDARD ART AND COTRIMOXAZOLE OR ISONIAZID PROPHYLAXIS

Toxicity will be managed in all randomised groups according to standard clinical practice. Blood tests additional to those described in the trial schedule may be requested at any time for clinical management of the patient: for example, a patient receiving zidovudine who has anaemia may have haemoglobin monitored more frequently. Wherever possible, any side effects will initially be managed by symptomatic measures and administration of appropriate (non-contraindicated) medication. In particular, Grade 1-2 gastrointestinal side effects such as nausea (with or without vomiting), and diarrhoea will be managed by anti-emetics and or anti-diarrhoeal agents in the first instance. Interruption of or changes in ART will be avoided except in the event of Grade 3 or 4 toxicity that is considered at least possibly related to one or more of the ART drugs. Wherever possible, alternative ART drugs will be substituted from the same class in order to maintain the patient's randomised treatment strategy.

Alternate explanations for clinical or laboratory abnormalities that may at first appear to be related to a specific drug should be sought. The use of reduced doses of any drug is discouraged, unless this is specifically recommended in the Summary of Product Characteristics (eg tenofovir dose reduction for estimated creatinine clearance <50ml/min) .

Management of any other adverse events/toxicity should generally follow the criteria below, but clinicians should use their clinical judgement as to the best management for the individual patient.

- Grade 1:
    - Continue study drugs
    - Routine monitoring

  - Manage using symptomatic measures and other concomitant medication, if appropriate

- Grade 2:
  - Continue study drugs
  - Monitor closely with more frequent visits (i.e. every two weeks) and consider more frequent laboratory assessments
  - Manage using symptomatic measures and other concomitant medication, if appropriate
  - Work-up to exclude other causes

- Grade 3 or 4:
  - Request laboratory results if relevant, obtain repeat confirmatory laboratory results within 72 hours
  - Continue study drugs pending receipt of the confirmatory laboratory tests/repeat observations, unless immediate need to substitute
  - Work-up to exclude other causes
  - Following confirmation of toxicity, and lack of other cause data:
    - if not too sick, substitute immediately
    - otherwise, stop all drugs (stagger stop NNRTIs or integrase) and restart with substituted drugs when better

Note that staggered stop is essential for NNRTIs (and may also be important for integrase inhibitors) because these drugs have long half lives and low genetic barriers to HIV resistance. Therefore stopping NNRTIs + 2NRTIs together (or stopping raltegravir at the same time as all other ART drugs) will lead to a period of time where only NNRTI (or integrase) remains in the adult, adolescent or child's blood, with the potential for development of resistance. Therefore, if a participant is taking an NNRTI-containing regimen (with or without integrase), stop the NNRTI (and integrase) immediately and continue the remaining ART drugs for a further 10 days for nevirapine and 14 days for efavirenz. Stop all remaining ART drugs together after 10 or 14 days respectively.

For severe reactions where the cause may be attributed to one or more trial drugs, all drugs should be stopped temporarily and may be restarted if the symptoms resolve and this is appropriate (stagger stop for NNRTIs and integrase wherever possible).

5.7.1 Information on specific ART drug toxicities

Also see Appendix IV- Details of Antiretroviral Therapy, p114, and Appendix V - Table of Clinical Signs, Symptoms, Monitoring and Management of Symptoms of Serious Adverse Effects of Antiretroviral Drugs that Require Drug Discontinuation, p118.

Raltegravir is generally well-tolerated, and overall adverse events were no more common than with placebo in treatment-experienced patients, and occurred less often than with efavirenz in studies involving a treatment-naïve population[9, 58].

The tolerability of the NRTIs will depend on the particular combination used. Tenovovir is very well tolerated, the main concern being renal toxicity which is rare. The main concern with abacavir is the risk of a hypersensitivity reaction (occurring in around 2% of Ugandan adults in the NORA substudy of the DART trial[59], but only 0.4% of 1206 Ugandan/Zimbabwean children in the ARROW trial[60]). Subjects receiving abacavir as part of their treatment regimen who develop rash of any grade, and/or any other symptom suggestive of allergic reaction, will be evaluated for the possibility of a clinically suspected abacavir hypersensitivity reaction (HSR) or a serious skin reaction such as Stevens Johnson Syndrome, Toxic Epidermal Necrolysis or Erythema Multiforme, and managed appropriately as

outlined in the local prescribing information. In the event of discontinuation of abacavir for any reason, the re-initiation of this drug will be undertaken with caution, and abacavir will not be re-initiated if a potential abacavir HSR is considered to have taken place. Health care providers will obtain a complete history of the events surrounding the discontinuation of abacavir and evaluate for the possibility of a clinically suspected hypersensitivity reaction, and the participant will be managed appropriately as outlined in the local prescribing information.

The use of zidovudine should be carefully considered in patients with WHO 2007 grade 3 neutropenia and/or anaemia – and other ART drugs should be used wherever possible. If no other options are available, clinicians should use their clinical judgement as to whether starting ART with zidovudine is still in the patient's best interests, given their advanced HIV disease and the likely improvements in haemoglobin on ART. A reduction of the zidovudine dose to 200 mg twice daily and/or use of an alternative regimen to zidovudine should be considered for patients with WHO 2007 grade 2 neutropenia and/or WHO 2007 grade 2 anaemia at screening, also taking account of local guidelines. If zidovudine is dose-reduced, the haemoglobin and/or neutrophil level should be rechecked after 4 weeks of treatment in participants taking low dose zidovudine, with consdieration to increase the dose. Clinicians should always be alert to clinical signs of anaemia, neutropenia, and thrombocytopenia, and participants should receive education or counselling about the associated symptoms. Alternative causes for anaemia and/or neutropenia, such as concurrent bacterial, mycobacterial or fungal infection, malaria, helminthiasis, malignancy and/or malnutrition should be investigated as appropriate, and treatment commenced if possible, prior to starting any zidovudine-containing products. If appropriate, participants who develop anaemia, neutropenia, and/or thrombocytopenia on study should also be evaluated for non-drug related causes and treated appropriately.

5.7.2 ART DRUG SUBSTITUTIONS

If a participant does not tolerate an individual drug, an alternative drug may be substituted if this is considered appropriate by the investigator (see Table 5.4, below) and other drugs restarted. Wherever possible, substitutes should be made within class.

Table 5.5 Guidelines for Substituting for Toxicity

EVENT	SUBSTITUTION*	
3TC/FTC toxicity	Adults:<br>Children:	Substitute 3TC /FTCwith ZDV or TDF<br>Substitute 3TC /FTCwith ZDV or TDF or d4T or ABC
ZDV toxicity	Adults:<br>Children:	Substitute ZDV with TDF<br>Substitute ZDV with TDF or d4T or ABC
TDF toxicity	Adults:<br>Children:	Substitute TDF with ZDV<br>Substitute TDF with ZDV or d4T or ABC
ABC toxicity	Adults:<br>Children:	Substitute ABC with ZDV or TDF<br>Substitute ABC with ZDV or TDF or d4T
NVP toxicity	All:	Substitute NVP with EFV
EFV toxicity	All:	Substitute EFV with NVP

* Choice of substitution depends upon:
(i) other drugs being taken
(ii) available formulations (e.g. size of tablet).
If possible, preferred substitution would be within the same class. TDF has only recently been licensed for use in children, and paediatric formulations may not be available.

NOTE: In the event of discontinuation of ABC for any reason, re-initiation of this drug should be undertaken with caution. Health care providers should obtain a complete history of the events surrounding the discontinuation of ABC. If there are any symptoms consistent with a hypersensitivity reaction, ABC should not be reinitiated. If there is no evidence of a prior reaction, the subject may restart treatment with ABC. The subject and health care provider should be aware of the possibility of a rapid-onset hypersensitivity reaction upon re-initiation of ABC, which may be life-threatening, and the subject should be able to, if necessary, receive prompt medical evaluation. All carers of children receiving abacavir will receive warning cards and additional information about this drug (Appendix I - Template Patient information sheets, p91).

5.7.3 MANAGEMENT OF POTENTIAL ABC OR NVP REACTIONS WHEN CHILDREN ARE TAKING BOTH

If children are taking both ABC and NVP, it may be more difficult to isolate the causal agent for a suspected severe reaction as both drugs have overlapping signs/symptoms. Clinicians should first check Liver Function Tests (LFTs) as raised LFTs would suggest that a reaction is more likely to be due to NVP. However, regardless of LFT levels the first action in the case of a severe reaction should be to stop NVP and monitor the child. If the reaction subsides after stopping NVP, substitute for NVP according to Table 5.4, above. If the reaction continues, stop 3TC+ABC immediately: after it has subsided, restart NVP (with dose escalation) +3TC+ZDV.

5.8 PROTOCOL TREATMENT DISCONTINUATION

In consenting to the trial, patients are consenting to trial treatment, trial follow-up and data collection. However, an individual patient may stop treatment early or be stopped early for any of the following reasons:

- Unacceptable toxicity or adverse event

- Intercurrent illness that prevents further treatment
- Any change in the patient's condition that justifies the discontinuation of treatment in the clinician's opinion
- Inadequate compliance with the protocol treatment in the judgement of the treating physician
- Withdrawal of consent for treatment by the patient

As the patient's participation in the trial is entirely voluntary, they may choose to discontinue the trial treatment at any time without penalty or loss of benefits to which they are otherwise entitled. Although the patient is not required to give a reason for discontinuing their trial treatment, a reasonable effort should be made to establish this reason while fully respecting the patient's rights.

Patients should remain in the trial for the purpose of follow-up and data analysis (unless the patient withdraws their consent from all stages of the trial). If a patient is withdrawn from follow-up, refer to Section 6.8 Early Stopping of Follow-up Figure 1 p60. Data will be kept and included for patients who stop follow-up early, up to the point of withdrawal.

5.9 COMPLIANCE & ADHERENCE

As the interventions will start immediately following randomisation, suitable patient information and fully informed consent procedures will ensure that participants understand the trial requirements. Therefore any non-compliance will likely be a consequence of the interventions themselves (eg drug intolerance or toxicity) which would also likely occur if the interventions were incorporated within clinical practice, ie are part of the pragmatic strategy being evaluated. Adults or children developing toxicity will be able to either drop the induction integrase drug or switch to an alternative antiretroviral, or drop the enhanced prophylaxis drugs. Any participants needing treatment for oral/oesophageal candidiasis or TB will receive treatment, regardless of randomisation to enhanced prophylaxis or not, although the numbers are not expected to be >5% based on data from DART/ARROW. The intention-to-treat comparison will therefore incorporate the level of non-compliance as would be anticipated in general clinical practice.

Compliance to ARVs and prophylaxis drugs will be assessed by pill counts and short patient-reported adherence questions at every visit. At weeks 4, 12, 24 and 48 a longer adherence questionnaire eliciting both understanding of drug dosing regimens and reasons for non-adherence will be completed. An acceptability questionnaire at weeks 4, 12 and 24 will elicit participant opinions about the different regimens, particularly with regard to pill burden, complexity and stigma.

5.10 NON-TRIAL TREATMENT

All necessary concomitant medications are allowed. If a medication with a known drug interaction to one of the trial medications is essential for a patient's management, then, if appropriate dose adjustment is not possible, the trial medication should be stopped and the concomitant medication used. (See Section 6.7 - Management of tuberculosis, p60, for specific issues relating to tuberculosis.)

6 ASSESSMENTS & FOLLOW-UP

All participants will be followed for a minimum of 48 weeks for evaluation of all-cause mortality, morbidity and toxicity. All participants will be seen at clinic at screening, ART initiation and then at 4, 8, 12, 18, 24, 36 and 48 weeks later, when they would exit the trial and rollover into national ART programs. When the last patient reaches 48 weeks, current vital status of all participants would be ascertained from local programmes (consent will be sought for this together with consent for trial participation, see Appendix II - Screening and Trial Consent and Assent forms, p101).

Trial visit schedules will be prepared for each patient at randomisation, and patients should be followed on the same schedule even if their trial medication is discontinued. The target dates for trial visits are determined by the date of randomisation and are not affected by subsequent events. The schedule defines visit dates (with windows) necessary for data collection, but the patient may be seen more frequently for clinical care as needed.

Patients will be expected to attend on the scheduled day unless agreed in advance with the clinic. If they are unable to attend on the day, every effort should be made to complete the visit within 7 days of the scheduled date. If a scheduled visit is missed without notice then the clinic should endeavour to contact the patient by phone or by home visit. Scheduled assessments should be carried out no more than 7 days before the scheduled visit. Clinics may choose to re-schedule visits to allow for public holidays or other unavoidable circumstances that affect the scheduled visit date, but the re-scheduled visit should be no more than 7 days from the originally scheduled visit date. Patients will also be given a card with the contact details for the trial research team.

If a patient is more than 7 days late for a scheduled study visit, an additional visit will be performed as soon as possible, including the appropriate assessments that were specified in the trial schedule for the visit week that was missed.

Additional study visits may need to be arranged, for example if the patient develops drug toxicity or other clinical events. These visits will usually be clinician and nurse visits. Routine assessments as for a standard clinician and nurse visit will be performed (as for week 24, although plasma storage and CD4+ count are optional). Other laboratory tests will be performed as clinically indicated. Where pregnancy is suspected a pregnancy test should be performed. At the time of any tuberculosis diagnosis (pulmonary or extra-pulmonary) during REALITY a specific blood draw will be taken using the QuantiFERON®-TB Gold In-Tube system (3 ml in 3 x 1 ml tubes) (JCRC/Harare only) (see Appendix VI - Handling and Storage of Specimens, p120).

6.1 TRIAL ASSESSMENT SCHEDULE

See page xiii for the Trial Assessment Schedule.

At each full doctor assessment (weeks 4 , 12, 24, 36, 48), the following will be undertaken:

- Administration of a symptom checklist by a nurse to detect intercurrent illness, HIV disease progression or adverse events to ART. The severity and likely relationship of any adverse events will be documented by a doctor.
- Medical history since last visit including adverse events to ART, signs and symptoms of HIV disease and WHO stage.

- Weight, body composition by bioelectrical impedance analysis (BIA), and mid-upper arm circumference in all participants, plus height in those under 18 years.
- Haematology and CD4 cell count and percent (not week 36)
- Plasma store and DNA pellets (see Appendix VI - Handling and Storage of Specimens, p120); stored plasma samples and DNA pellets may be used subsequently for HIV RNA viral load measurements, measurements of cell associated HIV DNA, HLA typing and analyses of drug levels.
- EQ-5D (see Appendix VII - EQ-5D questionnaire, p122)
- Assessment of adherence to ART and prophylaxis drugs by pill counts and nurse administered questionnaire.
- Changes in ART, OI prophylaxis and other concomitant medication.
- All girls and women of childbearing age (aged > 12 years and who have reached menses) will be given continuing advice about avoiding pregnancy.
- Children and adults with fever and/or other symptoms suggestive of malaria should all have thick and thin films performed. Inpatient and outpatient attendances should be recorded on CRFs, both with appropriate laboratory forms completed to document test results.

Although not required by the protocol, an additional visit 2 weeks after initiating ART may be scheduled at the discretion of the study clinician, particularly in those participants enrolling with acute illnesses.

At specific designated 4 or 12 weekly visits, or in specific subgroups of participants, the following will be undertaken:

- At weeks 0, 4, 12 and 48, grip strength will be measured using a digital handgrip dynamometer.
- At screening, week 4 and week 48 Creatinine, Albumin[61], and Phosphate[62] will be measured. AST or ALT and Bilirubin will be measured at screening and week 4 only. After week 4 other biochemistry tests may be performed if clinically indicated, but are not required by the protocol
- Women who have reached menses and are pre-menopausal should have a routine pregnancy test at weeks 24 and 48. All such women randomised to receive 12 weeks of fluconazole at trial entry should also have a routine pregnancy test at 4 and 8 weeks after enrolment.
- For those screened and enrolled at Harare , JCRC Kampala and Kilifi, immunophenotyping to identify additional T-cell subsets (defined by CD38 and HLA-DR) and markers of cellular turnover and senescence will be measured at screening and weeks 4, 12, 24, 48 (as standard flow cytometry). These tests will be done on the same blood draw as for routine haematology and lymphocyte subsets.
- For participants enrolled at Harare and JCRC Kampala, QuantiFERON®-TB Gold In-Tube system supernatants will be stored at weeks 0, 4, 12 and 24 for later research into cytokine and lipopolysaccharide (LPS) levels, and its value for predicting development of subsequent TB. Therefore, in addition to this, all participants at Harare and JCRC Kampala sites diagnosed with TB will have Quantiferon stored at the time of TB diagnosis and 3 months after diagnosis. Quantiferon tubes are taken from the same total blood draw as for routine plasma storage. Consent for the future use of samples is included in the trial consent form (see Appendix II - Screening and Trial Consent and Assent forms, p101).
- For participants at Harare and Kilifi, at weeks 0, 4, 12, 24 and 48, stored plasma will be assayed for plasma markers of systemic inflammation (CRP, cytokines, chemokines, D-dimer, hyaluronic acid) and markers of microbial translocation including LPS, soluble CD14 and anti-LPS antibodies. Fresh blood will be used for functional assays to evaluate responses to pathogen challenge (e.g. LPS) in ex-vivo whole blood stimulation assays. Stool samples will be colected to determine markers of intestinal inflammation (neopterin, calprotectin, a1-antitrypsin) at 0, 4, 12 and 48

weeks. Additional consent will be collected for collection of stool samples (See Appendix III - Template Patient information sheets and consent for collection of stool samples p108.)
- At Harare site, body fat will be assessed by triceps, subscapular, supra-iliac, mid-thigh skinfold thicknesses at weeks 0, 12, 48, measured using Holtain calipers following a standard Manual of Operations.

At enrolment and week 48 the following will be undertaken:
- clinical examination to assess peripheral neuropathy
- assessment of socioeconomic status (and the impact of ART on this at week 48)

At each nurse visit, the study nurse will:
- Administer the symptom check-list
- Give advice on avoiding pregnancy (for those who have reached puberty)
- Administer the EQ-5D (see Appendix VII - EQ-5D questionnaire, p122)
- Assess adherence to ART and prophylaxis drugs using a few short patient-reported measures and recording numbers of returned pills/ bottles.
- Administer the next 4/6/12 weeks supply of drugs to last until the next clinic visit
- If the study nurse has any clinical concerns at these visits, the patient should be referred to see a study doctor.

Participants in all groups may undergo all necessary diagnostic tests for clinical management of illness. If children, adolescents or adults become acutely unwell (particularly if they are ill enough to have blood tests done outside of the routine blood sampling schedule) then plasma (EDTA blood) and DNA cell pellet should be stored, for subequent molecular diagnostics assessment (see Section 10.2 - Molecular diagnostics, p73). Participants in Harare and JCRC Kampala who develop tuberculosis should have Quantiferon supernatants stored at diagnosis and 3 months subsequently.

6.2 PROCEDURES FOR ASSESSING EFFICACY

The primary outcome is all-cause mortality, reportable on an Event form (see Section 7.1 - Safety Definitions, p62 below).

6.2.1 Clinical events (All participants)

A symptom checklist and targeted physical examination (to evaluate any reported symptoms) will be performed at each visit; if there are problems, patients will be referred to a doctor. Hospital admissions will be solicited at all visits. Where there is any clinical suspicion of a WHO Stage 3 or 4 disease event, centres will endeavour to investigate the patient to the fullest extent possible given local availability of imaging and laboratory investigations (particularly microbiology) in order to establish a clear diagnosis of the event. The list of these diseases and diagnostic criteria are provided in Appendix IX - Clinical Progression and WHO Definitions, p125. The site will report the event and the clinical investigations that support the diagnosis in order to enable an independent evaluation of the event. A clinical expert review committee will review all clinical endpoints to ensure that they satisfy the diagnostic criteria. All pathogen isolates, and any TB smears, will be stored for further analysis (see Section 10.3, p74).

6.2.2 CD4+ T-cell counts (All participants)

Blood will be collected at 4, 12, 24 and 48 weeks for determination of total and percentage CD4 T-cell counts. The CD4 count will be done using the standard assay in operation at each site laboratory according to quality-assured procedures.

6.2.3 VIRAL LOAD TESTING (ALL PARTICIPANTS; ADDITIONAL TESTING IN A SUBSET)

Plasma HIV viral load (VL) will not be measured in real-time, but will be assayed retrospectively in all participants in batches of stored plasma samples taken at enrolment, week 12 and week 48 (or the final trial visit). Additional measurements of VL will be performed as a sub-study on stored plasma samples from intervening time points in a smaller number of patients (n=600) to assess the rapidity of initial virological response and to define more precisely the timing of VL rebound in those patients who do not have virological suppression at week 48. Additional funding will be sought for viral load testing.

All batched VL measurements done after randomisation will be performed using the same assay (lower limit of detection of no greater than 80 copies/ml) in one or more designated laboratories using the same assay platform. The selection of laboratories will be made based on the availability of the appropriate VL assay, and the presence of adequate quality assurance procedures. The treating clinicians and patients will remain blind to VL test results throughout the period of follow-up. However, the Data Monitoring Committee (DMC) (Section 14.4) will review the batched week 48 VL results in conjunction with the resistance test results when these are available. The results of all the viral load tests performed in individual patients will be given to the treating physician after the last participant has completed 48 weeks follow-up, or as soon thereafter as results become available.

6.2.4 RESISTANCE TESTING (SUBSET OF PARTICIPANTS)

Batched genotypic resistance testing will be performed retrospectively on week 48 samples from all patients who have a VL >10,000 copies/ml at week 48. Additional resistance tests will be performed on stored plasma samples in selected patients at the end of the trial to define in more detail the pattern of resistance development. Additional funding will be sought for resistance testing.

All resistance test measurements will be performed using the same assay in a laboratory or combination of laboratories that have the most experience of HIV resistance testing and that have stringent quality assurance procedures in place. Drug resistance mutations will be classified using the Stanford database algorithm.

The treating physicians and patients will remain blind to resistance test results throughout the period of follow-up but the DMC (Section 14.4) will review batched week 48 resistance test results in conjunction with the VL results when these are available. The results of all resistance tests performed in individual patients will be given to the treating physician after the last participant has completed 48 weeks follow-up, or as soon thereafter as results become available.

6.2.5 WEIGHT, BODY COMPOSITION, GROWTH AND GRIP STRENGTH (ALL PARTICIPANTS; ADDITIONAL TESTING IN A SUBSET)

All participants will be weighed by the nurse at every study visit, and have body composition estimated using bioelectrical impedance analysis (BIA). BIA requires patients to stand on a machine very similar to weight scales - a small electrical current which cannot be felt is run through the body and used to estimate the proportion of lean body mass and fat. Mid-upper arm circumference will also be measured at every visit. Height will be measured at screening in all participants: those under 18 years will have height measured at every visit, following standard procedures which will be detailed in the trial Manual of Operations.

The distribution of body fat will be assessed by skinfold thicknesses at enrolment, week 12 and 48 in a subset of participants (those enrolled at Harare).

A major challenge for HIV-infected people with advanced immuno-deficiency is lack of strength. We will therefore measure grip strength at enrolment, and weeks 4, 12 and 48 using a digital handgrip dynamometer, which will also inform cost-effectiveness modelling. These handgrip dynamometers have previously been used successfully in older[63] and younger children[64].

6.2.6 Measures of the rate and quality of the immune restoration (Subset of participants)

Immune restoration comprises several processes: i) controlling HIV replication; ii) controlling co-infections and OIs; iii) controlling gut inflammation and microbial translocation; and iv) uncontrolled inflammation during immune reconstitution inflammatory syndrome (IRIS). All of these factors act as both cause and effect with a highly proinflammatory milieu being assocated with HIV transcription, inadequate immune responses and infection. The first three of these lead to improved ability to respond appropriately to pathogen challenges, ultimately reflected by a reduced incidence of infections; to further control of HIV replication which is itself driven by immune activation of T cells containing pro-virus; and potentially to improved nutritional status by avoiding nutrient 'wastage' on inflammation. All three interventions may potentially influence these processes. We therefore intend to examine the effects of each intervention on the relative contribution of each of these mechanisms, and the funtional consequences.

In a subset of participants (those enrolled at the Harare, JCRC Kampala and Kilifi sites), further immunophenotyping will be carried out on the blood sample taken for haematology and lymphocyte subsets at screening, 4, 12, 24 and 48 weeks, in order to ascertain T-cell immune activation status resulting from pre-existing inflammation and IRIS, turnover and senescence using a panel of antibodies (including CD38 and HLA-DR). Stimulation assays will be conducted using either whole blood to determine the efficacy of responses to pathogen challenge (e.g. LPS, viral and fungal antigens). Markers of systemic and gut inflammation (CRP, cytokines and chemokines, and plasma beta-2 microglobulin) and plasma markers of microbial translocation (LPS, soluble CD14, 16s PCR and anti-endotoxin antibodies) will be measured.

Supernatants from the QuantiFERON®-TB Gold In-Tube (IT) system will also be stored at week 0, 4 and 12. This system consists of three vacutainer type tubes, a Nil Control (Grey cap), TB Antigen (Red cap) and Mitogen Control (Purple cap). Antigens have been dried onto the inner wall of the blood collection tubes so it is essential that the contents of the tubes be thoroughly mixed with the blood. 1ml of blood is required for each tube - these 3mls of blood are to be taken from the amount originally allocated for plasma storage. The tubes must be transferred to a 37°C incubator as soon as possible and within 16 hours of collection and then incubated and supernatants collected and stored as instructed in the REALITY Manual of Operations (MOP).

6.3 PROCEDURES FOR ASSESSING SAFETY (ALL PARTICIPANTS)

The symptom checklist used at each visit will explicitly prompt for symptoms relating to possible drug toxicities. The checklist will be part of the CRF completed by the nurse at each visit.

Patients will be asked about symptoms of peripheral neuropathy at baseline and week 48 (or the final trial visit), and examined for neuropathy using simple tests with a tuning fork.

Blood will be drawn at trial visits to assess laboratory safety parameters according to standard-of-care as indicated in the schedule of trial assessments. Additional safety blood tests or investigations may be performed to investigate symptoms or monitor emergent laboratory test abnormalities as clinically indicated. This clinically-driven approach to monitoring toxicities of antiretroviral therapy

was demonstrated to be equivalent in terms of adverse event outcomes to routine laboratory monitoring for toxicity in the DART trial[65].

All adverse events will be reported on the case report form. Adverse events (clinical and laboratory) will be graded using the 2004 Division of AIDS toxicity grading scale (see Appendix X - Toxicity Gradings and Management, p136), with a minor modification of the neutrophil count grading to reflect norms in the African HIV infected population. Serious adverse events will be defined according to ICH GCP, and will be reported to the MRC CTU according to standard timelines (see Section 7 - Safety Reporting, p62). All adverse events meeting the definitions above should be reported on study CRFs, regardless of their relationship to HIV. Serious non-AIDS events (HIV-related events which have not traditionally been included in WHO staging) will also be collected as serious adverse events; these include

- End-stage renal disease
- Cirrhosis
- Hepatic failure
- Oesophageal bleeding with end stage liver disease
- Myocardial infarction
- Stroke
- Clinical severe pancreatitis
- Non-AIDS malignancies

6.4 PROCEDURES FOR ASSESSING ADHERENCE (ALL PARTICIPANTS)

Adherence to ART and prohylaxis drugs will be assessed in all participants at each visit by pill counts for tablets, and short nurse-administered adherence questions. Every 12 weeks, a more detailed adherence questionnaire will be administered to the adult, adolescent or child’s carer, and where appropriate to the child (at the discretion of the nurse or doctor). This will include questions about adherence to all the trial inteventions (ie ART, OI and other prophylaxis and trial RUSF).

6.5 OTHER ASSESSMENTS

6.5.1 HEALTH ECONOMICS (ALL PARTICIPANTS)

The trial will measure healthcare-related costs in trial participants, starting at randomisation and continuing for the duration of follow-up. Costs incurred by the patients and their families (transport, indirect and companion person’s costs) will be obtained by patient reports. Reported transport costs will be confirmed using local information on distance and cost of transport. Information on hospitalisations (number, reason, and duration of stay) will be collected from hospital records, and data on other healthcare resource utilisation (outpatient visits, medications, and procedures) will be collected by abstraction of patient medical notes and by patient interview.

A simple quality of life instrument (EQ-5D) will be used to permit cost-utility analysis (seeAppendix VII - EQ-5D questionnaire, p122). Translations of the questionnaire will be made into appropriate local languages and verified using standard forward and back translation procedures. The questionnaire will be administered in the patient's own language by a member of the research team. The language in which the questionnaire was administered will be recorded on the CRF.

6.5.2 MOLECULAR DIAGNOSTICS (SUBSET OF PARTICIPANTS)

There are few robust data on bacterial and other infections in patients with severe immuno-deficiency initiating ART, despite sepsis being the major cause of early mortality. Lack of knowledge about specific causes of morbidity/mortality hinders identification of interventions for evaluation. Therefore plasma samples from any discarded blood taken at the time of any acute event will also be stored for evaluation in the molecular diagnostics substudy. Furthermore, any bacterial isolate grown from standard culture will also be stored. See Section 10 - Ancillary Studies, p73 for more details. In brief, stored samples will be analysed retrospectively using broad-range 16s ribosomal DNA PCR and specific bacterial qPCR panels, and associations with clinical events and immune/gut function (see Section 6.2.6 - Measures of the rate and quality of the immune restoration (Subset of participants) , p56 above) will be explored. As with other assays (see Trial Assessment Schedule page xiii) the plan is for these assays to be performed locally; however some of these samples may need to be shipped to the UK for assays which cannot be performed in country.

6.5.3 PROCEDURES FOR ASSESSING SOCIOECONOMIC STATUS (ALL PARTICIPANTS)

The impact of the different treatment groups on the socio-economic status of trial participants will be assessed using a simple instrument (survey). The survey is based on standard indicators of socioeconomic status of both individuals and their household and will be conducted at enrolment and week 48 (or the final trial visit) to detect changes over the course of the trial.

The instrument contains a series of questions that differentiate between individuals, and the household they belong to, on various socioeconomic outcomes that can be linked to representative household surveys of income and health in African populations. The questions focus on monthly household income, income proxies (including type of housing, access to water, nature of occupation), number of children at school and access to adequate food.

The aim will be to be able to track changes in socioeconomic outcomes after treatment and across various treatment groups at the level of the household. Side effects of certain treatments could lead to standards of living dropping, children dropping out of school, adults in good health dropping out of work to care for the sick and a worsening of food security within the household. Other treatments could improve these outcomes and it is hence important to quantify these changes as part of the REALITY trial.

The data collected will be transformed into both continuous and binary classifications of socioeconomic status with living standards being categorised into high, medium and low outcomes and, if possible, by also using actual income levels.

6.6 MANAGEMENT OF PREGNANCY

Patients currently pregnant or breastfeeding are not eligible for enrolment in REALITY. As eligible participants will have very low CD4 counts, early counselling will stress that any planned pregnancies would be better deferred until immune reconstitution on ART has occurred after the first year on ART, and will refer patients to family planning services as appropriate. However, it will also be made clear in the initial counselling about the trial that women who nevertheless become pregnant during the course of the trial will be able to continue in the trial, and will be able to continue to receive the best available antiretroviral therapy during their pregnancy and subsequent period of breastfeeding. If a woman gets pregnant, she will be given counselling on the risk of continuing the pregnancy, the risk of mother-to-child transmission of HIV infection, and the risks and benefits of antiretroviral treatment regimens during pregnancy and breastfeeding.

Pregnancy testing will be undertaken in all girls aged 12 years or older who have reached menses and all pre-menopausal women at 24 and 48 weeks, and also whenever such a girl/women has missed a period. Any such women randomised to receive 12 weeks of fluconazole at trial entry will also undergo routine pregnancy testing at 4 and 8 weeks after enrolment (whilst they are still being prescribed fluconazole).

6.6.1 SUMMARY OF CURRENT DRUG SAFETY PROFILES IN PREGNANCY

The nucleoside combinations that will be most commonly used in this trial (tenofovir, emtricitabine/ lamivudine, zidovudine, abacavir) and the non-nucleoside, nevirapine, are generally considered to be safe in pregnancy and are widely used. Modifications to the NRTI combination may be made by the treating physician based on the assessment of risks of particular drugs in pregnancy and their likely antiretroviral efficacy.

Efavirenz has been previously classified by the FDA as a pregnancy category D drug (Positive Evidence of Fetal Risk) on the basis of animal studies. However, recent data on infants exposed to efavirenz during the first trimester from the international pregnancy register has not shown significant additional risks of adverse effects in babies exposed to efavirenz compared to infants exposed to other antiretrovirals[66]. Therefore, guidelines have relaxed their advice about use of efavirenz in pregnancy[49] and now recommend that women getting pregnant should stay on the same ART regimen.

At present there are inadequate data regarding the use of raltegravir in pregnant women. Raltegravir is in FDA Pregnancy Category C (Risk of Fetal Harm Cannot Be Ruled Out). The manufacturer’s summary of product characteristics states that in animal studies, no treatment-related effects on embryonic/foetal survival or foetal weight were observed in rabbits (up to 1000 mg/kg/day) or rats (up to 600 mg/kg/day) receiving up to 3- to 4-fold the exposure at the recommended human dose. No treatment-related external, visceral, or skeletal changes were observed in rabbits. Pharmacokinetic studies have shown that raltegravir exposure is not lowered in pregnancy and that it efficiently crosses the placenta[67, 68].

Fluconazole has recently been reclassified from pregnancy category C to D for doses of >400mg daily. Fluconazole will be dosed at 100mg daily for 12 weeks in REALITY; however, additional pregnancy testing will be done 4 and 8 weeks after enrolment in all women randomised to receive 12 weeks fluconazole who have reached menses and are pre-menopausal.

6.6.2 TREATMENT MODIFICATION

As raltegravir is being used as an additional drug as well as standard first-line 3-drug ART, any woman identified as pregnant whilst receiving raltegravir will stop the drug immediately, as it is being given in addition to standard of care ARVs.

Isoniazid, fluconazole and cotrimoxazole should all be continued if a women becomes pregnant.

6.6.3 PREGNANCY FOLLOW-UP AND REPORTING

Pregancy will be reported on standard follow-up CRFs. Pregnancy related events do not constitute SAEs unless they result in a condition that meets the seriousness criteria defining SAEs (eg septic abortion) (see Section 7.1 - Safety Definitions, p62). All pregnancies occurring in REALITY will be followed until their outcome is known. All pregnancies and their outcomes will be reported by the MRC CTU to the Antiretroviral Pregnancy Registry. All infants will receive infant prophylaxis according to the current local standard of care. This may include single dose nevirapine.

6.7 MANAGEMENT OF TUBERCULOSIS

Tuberculosis is common in Africa, and is likely to account for many of the opportunistic infections seen during the course of this trial. Rifampicin, a cornerstone of the treatment of tuberculosis, has interactions with many drugs including nevirapine and raltegravir.

Patients who develop tuberculosis during the course of the study whilst taking nevirapine should substitute with efavirenz. Whilst rifampicin decreases raltegravir AUC and Cmax by approximately 40%, **raltegravir dose changes are not currently recommended** by CDC[69] or others[69, 70], or by the innovator company, Merck, due to the wide therapeutic index of raltegravir. If a patient taking raltegravir developes tuberculosis during the course of the study, or a patient taking rifampicin as part of treatment is allocated to the raltegravir containing ART strategy, they should continue raltegravir alongside anti-tuberculosis medications; raltegravir will in any case always be an additional fourth ARV.

Patients developing TB on any randomised group in REALITY should immediately commence TB treatment with rifampicin, isoniazid, ethambutol and pyrazinamide, following national guidelines. Cultures should be tested for sensitivity to first-line drugs and regimens modified on the basis of these results. These assays will be conducted locally.

Patients may need to be referred to the national TB treatment programme in order to access anti-tuberculosis medication, and close liaison between the trial team and the TB treatment providers will be established. Any patient randomised to the enhanced OI prophylaxis group and completing TB treatment during the first 12 weeks of the trial should continue to take isoniazid prophylaxis for the rest of the trial.

6.8 EARLY STOPPING OF FOLLOW-UP

In consenting to the trial, patients are consenting to treatment according to the allocated treatment strategy as well as to trial follow-up visits and data collection. If a patient chooses to discontinue their trial treatment, the clinician or nurse will explain the importance of remaining on trial follow-up or, failing this, of allowing routine clinic follow-up data to be used for trial purposes. The patient should always be followed up providing they are willing; that is, they should be encouraged to not leave trial follow-up even if they no longer wish to take trial treatment. If they do not wish to remain on trial follow-up, however, their decision must be respected and the patient will be withdrawn from the trial completely. The MRC CTU should be informed of this using the Consent Withdrawal CRF and the patient should complete the withdrawal of consent form (Appendix VIII - withdrawal form, p123). The reason for the patient withdrawing should be ascertained wherever possible.

If a patient withdraws from the trial, the medical data collected during their participation in the trial will be kept and used in analysis, as consent cannot be withdrawn for data already collected. Consent for future use of stored samples already collected can be refused when leaving the trial early (but this should be discouraged and should follow a discussion with the patient).

Prior to transferring to routine clinic follow-up or withdrawing completely from the trial, the patient will be asked to have assessments performed as appropriate for a final study visit although they would be at liberty to refuse any or all individual components of the assessment.

Patients may change their minds about stopping trial follow-up at any time and re-consent to re-enter participation in the trial.

Patients who stop trial follow-up early will not be replaced, as the total sample size includes adjustment for losses to follow-up.

6.9 PATIENT TRANSFERS

If a patient moves from the area, reasonable efforts should be made to continue their follow-up, eg by them continuing to come to clinic providing that the centre has sufficient funds to cover the necessary transport refund, or for example by conducting nurse only visits over the telephone. If this is not possible (eg if the patient moves to a different country), then the patient should be considered as lost to follow-up. The patient may or may not choose to formally register this as a withdrawal of consent.

6.10 LOSS TO FOLLOW-UP

In the statistical analysis, a patient will be classified as ‘lost to follow-up’ if they have not been seen in clinic for more than 3 months. For operational management in the clinic, a patient will be classified as 'lost to follow-up' (ie no further efforts to trace the patient are being made) when they have missed 3 scheduled clinic visits. During this time period attempts should be made to contact the patient via phone (if available) and to follow-up with home visits, if at all possible. Subsequently, if the patient attends clinic and a CRF is received by the MRC CTU, the ‘lost to follow-up’ status will be reversed.

6.11 TRIAL CLOSURE

The trial will end 48 weeks after the last patient has been randomised and all data queries have been resolved. At the end of the trial, vital status of all participants will be ascertained, and consent will be sought for this as it will be after the 48 week follow-up period for all but the last participant enrolled. The primary and secondary mortality comparisons will only be based on data collected during active follow-up (0-48 weeks), but a sensitivity analysis will consider longer-term mortality to investigate whether or not any early differences between randomised groups remain. Most participants will transfer care into the routine clinics at the centre where the trial is being conducted and so expected data completeness is high.

7 SAFETY REPORTING

The principles of ICH GCP require that both investigators and sponsors follow specific procedures when notifying and reporting adverse events or reactions in clinical trials. These procedures are described in this section of the protocol. Section 7.1 - Definitions lists definitions, Section 7.3 - Investigator Responsibilities gives details of the investigator responsibilities and Section 7.4 - MRC CTU Responsibilities provides information on MRC CTU responsibilities.

7.1 SAFETY DEFINITIONS

The definitions of the EU Directive 2001/20/EC Article 2 based on the principles of ICH GCP apply to this trial protocol. These definitions are given in Table 7.1: Definitions.

Table 7.1: Definitions

TABLE	DEFINITION
Adverse Event (AE)	Any untoward medical occurrence in a patient or clinical trial subject to whom a medicinal product has been administered including occurrences that are not necessarily caused by or related to that product.
Adverse Reaction (AR)	Any untoward and unintended response to an investigational medicinal product related to any dose administered.
Unexpected Adverse Reaction (UAR)	An adverse reaction, the nature or severity of which is not consistent with the information about the medicinal product in question set out in the Summary of Product Characteristics (SPC) or Investigator Brochure (IB) for that product.
Serious Adverse Event (SAE) or Serious Adverse Reaction (SAR) or Suspected Unexpected Serious Adverse Reaction (SUSAR)	Respectively any adverse event, adverse reaction or unexpected adverse reaction that:<br>▪ Results in death<br>▪ Is life-threatening*<br>▪ Requires hospitalisation or prolongation of existing hospitalisation**<br>▪ Results in persistent or significant disability or incapacity<br>▪ Consists of a congenital anomaly or birth defect<br>▪ Is another important medical condition***

*The term life-threatening in the definition of a serious event refers to an event in which the patient is at risk of death at the time of the event; it does not refer to an event that hypothetically might cause death if it were more severe, for example, a silent myocardial infarction.

**Hospitalisation is defined as an inpatient admission, regardless of length of stay, even if the hospitalisation is a precautionary measure for continued observation. Hospitalisations for a pre-existing condition (including elective procedures that have not worsened) do not constitute an SAE.

*** Medical judgement should be exercised in deciding whether an AE or AR is serious in other situations. The following should also be considered serious: important AEs or ARs that are not immediately life-threatening or do not result in death or hospitalisation but may jeopardise the subject or may require intervention to prevent one of the other outcomes listed in the definition above; for example, a secondary malignancy, an allergic bronchospasm requiring intensive emergency treatment, seizures or blood dyscrasias that do not result in hospitalisation or development of drug dependency.

7.1.1 MEDICINAL PRODUCTS

An investigational medicinal product is defined as the tested investigational medicinal product and the comparators used in the study. This therefore includes

- any antiretroviral drug, including raltegravir
- cotrimoxazole
- isoniazid/pyridoxine
- fluconazole
- azithromycin
- albendazole
- trial RUSF

Adverse reactions include any untoward or unintended response to any of the above medicinal products.

7.1.2 EXEMPTED ADVERSE EVENTS

Adverse Events include:

- An exacerbation of a pre-existing illness
- An increase in frequency or intensity of a pre-existing episodic event or condition
- A condition (even though it may have been present prior to the start of the trial) detected after trial drug administration
- Continuous persistent disease or a symptom present at baseline that worsens following administration of the study treatment

Adverse Events do not include:

- Medical or surgical procedures; the condition that leads to the procedure is the adverse event
- Pre-existing disease or a condition present before treatment that does not worsen
- Hospitalisations where no untoward or unintended response has occurred, eg, elective cosmetic surgery, social admissions
- Overdose of medication without signs or symptoms

7.1.3 DISEASE-RELATED EVENTS

All adverse events meeting the definitions above should be reported on study CRFs, regardless of their relationship to HIV. In particular, all deaths should be reported as fatal SAEs.

7.2 INVESTIGATOR RESPONSIBILITIES

All non-serious AEs and ARs, whether expected or not, should be recorded in the patient's medical notes and reported in the toxicity (symptoms) section of the Follow-up. SAEs and SARs should be notified to the MRC CTU within 1 working day of the investigator becoming aware of the event by emailing or faxing an Event form.

7.2.1 INVESTIGATOR ASSESSMENT

7.2.1.A Seriousness

When an AE or AR occurs, the investigator responsible for the care of the patient must first assess whether or not the event is serious using the definition given in Table 7.1: Definitions. If the event is serious, then an Event Form must be completed and the MRC CTU notified within 1 working day.

7.2.1.B Severity or Grading of Adverse Events

The severity of all AEs and/or ARs (serious and non-serious) in this trial should be graded using the toxicity gradings in Appendix X - Toxicity Gradings and Management, p136**.**

7.2.1.C Causality

The investigator must assess the causality of all serious events or reactions in relation to the trial therapy using the definitions in Table 7.2: Assigning Type of SAE Through Causality. There are five categories: unrelated, unlikely, possible, probable, and definitely related. If the causality assessment is unrelated or unlikely to be related, the event is classified as an SAE. If the causality is assessed as possible, probable or definitely related, then the event is classified as an SAR.

Table 7.2: Assigning Type of SAE Through Causality

RELATIONSHIP	DESCRIPTION	SAE TYPE
Unrelated	There is no evidence of any causal relationship	Unrelated SAE
Unlikely	There is little evidence to suggest that there is a causal relationship (for example, the event did not occur within a reasonable time after administration of the trial medication). There is another reasonable explanation for the event (for example, the patient's clinical condition, other concomitant treatment).	Unrelated SAE
Possible	There is some evidence to suggest a causal relationship (for example, because the event occurs within a reasonable time after administration of the trial medication). However, the influence of other factors may have contributed to the event (for example, the patient's clinical condition, other concomitant treatments).	SAR
Probable	There is evidence to suggest a causal relationship and the influence of other factors is unlikely.	SAR
Definitely	There is clear evidence to suggest a causal relationship and other possible contributing factors can be ruled out.	SAR

If an SAE is considered to be related to trial treatment and drug is stopped or the dose modified, refer to Section 5.8 - Dose Modifications, Interruptions & Discontinuations.

7.2.1.D Expectedness

If there is at least a possible involvement of the trial treatment (or comparator), the investigator must assess the expectedness of the event. An unexpected adverse reaction is one not previously reported in the current Summary of Product Characteristics (SPC) or one that is more frequent or more severe than previously reported. The definition of an unexpected adverse reaction (UAR) is given in Table 7.1: Definitions. Please see Appendix IV- Details of Antiretroviral Therapy, p114, for a

list of expected toxicities associated with the antiretroviral drugs being used in this trial. If a SAR is assessed as being unexpected, it becomes a SUSAR.

7.2.1.E Notification

The MRC CTU should be notified of all SAEs within 1 working day of the investigator becoming aware of the event.

Investigators should notify the MRC CTU of all SAEs occurring from the time of randomisation until the patient exits the trial.

7.2.2 Notification Procedure

1. The Event Form must be completed by the investigator (the physician named on the Signature List and Delegation of Responsibilities Log who is responsible for the patient's care), with due care being paid to the grading, causality and expectedness of the event as outlined above. In the absence of the responsible investigator, the form should be completed and signed by a member of the centre trial team and faxed or emailed as appropriate. The responsible investigator should subsequently check the Event Form, make changes as appropriate, sign and then re-fax or email to the MRC CTU as soon as possible. The initial report must be followed by detailed, written reports as appropriate.

The minimum criteria required for reporting an SAE are the trial number and date of birth, name of investigator reporting and why the event is considered serious.

2. The Event Form must be sent by fax to the MRC CTU
   Fax: +44 (0) 20 7670 4814 or email information to reality.mrcctu@ucl.ac.uk

3. Follow-up: patients must be followed up until clinical recovery is complete and laboratory results have returned to normal or baseline, or until the event has stabilised. Follow-up should continue after completion of protocol treatment if necessary. A further Event Form, indicated as 'Follow-up' should be completed and faxed to the MRC CTU as information becomes available. Extra, annotated information and/or copies of test results may be provided separately. The patient must be identified by trial number, date of birth and initials only. The patient's name should not be used on any correspondence and should be deleted from any test results.

4. Staff should follow their institution's procedure for local notification requirements.

SAE REPORTING
Within 1 working day of becoming aware of an SAE, please fax a completed Event form to the MRC CTU on:
Fax: +44 (0) 20 7670 4814 or email to reality.mrcctu@ucl.ac.uk

7.3 MRC CTU RESPONSIBILITIES

Medically-qualified staff at the MRC CTU and/or the Chief Investigator (or a medically-qualified delegate) will review all SAE reports received. The causality assessment given by the local investigator at the hospital cannot be overruled; in the case of disagreement, both opinions will be provided in any subsequent reports.

The MRC CTU is undertaking the duties of trial sponsor and is responsible for the reporting of SAEs to the relevant regulatory authorities, as appropriate. This responsibility is delegated to country PIs for relevant reporting requirements in individual countries (see Section 7.4 below).

The MRC CTU will also keep all investigators informed of any safety issues that arise during the course of the trial.

The MRC CTU, as Sponsor, will submit Annual Safety Reports to the UK Ethics Committee, and will provide these for country PIs to submit to their national agencies.

All drug companies involved will also be notified of events according to the agreed contractual arrangements.

7.4 RESPONSIBILITIES OF COUNTRY PRINCIPAL INVESTIGATOR

The country Principal Investigator's centre is responsible for the reporting of SAEs to the regulatory authorities in that country.

8 QUALITY ASSURANCE & CONTROL

8.1 RISK ASSESSMENT

The Quality Assurance (QA) and Quality Control (QC) considerations have been based on a formal initial Risk Assessment, which acknowledges the risks associated with the conduct of the trial and how to address them with QA and QC processes. QA includes all the planned and systematic actions established to ensure the trial is performed and data generated, documented and/or recorded and reported in compliance with the principles of ICH GCP and applicable regulatory requirements. QC includes the operational techniques and activities done within the QA system to verify that the requirements for quality of the trial-related activities are fulfilled. This Risk Assessment has been reviewed by the MRC CTU Quality Management Committee (QMC) and has led to the development of a Quality Management Plan (QMP), which will be kept separately.

The safety profiles of the drugs used in this study are well-known and acceptable. The drugs have been given to large numbers of patients in clinical trial settings and in clinical practice, although there is limited experience with one of the drugs (raltegravir) in African patients. Raltegravir is being used in the EARNEST trial which is currently in follow-up at three of the centres in REALITY, so most trial centres do have experience of using it. The trial will be recruiting patients with advanced HIV disease with relatively high rates of clinical events, but the trial sites in Africa have considerable experience with this population and this will serve minimise the risks to the patients and the trial. A detailed risk assessment will be conducted prior to starting the trial.

8.2 CENTRAL MONITORING AT MRC CTU

Each site will be responsible for its own data entry and local trial management. Data will be entered into the trial database directly at the site. The site will retain the original CRF. Data stored on the central database will be checked at MRC CTU for missing or unusual values (range checks) and checked for consistency within participants over time. If any such problems are identified, the site will be contacted and asked to verify or correct the entry. Changes will be made on the original CRF and entered into the database at the site. MRC CTU will also send reminders for any overdue and/or missing data with the regular inconsistency reports of errors.

Other essential trial issues, events and outputs will be detailed in the Monitoring and Quality Management Plans that are based on the trial-specific Risk Assessment.

8.3 ON-SITE MONITORING

Staff from MRC CTU will visit clinical sites to validate and monitor data and an independent local monitor will be employed in each country to make regular visits to trial sites. The frequency, type and intensity for routine monitoring and the requirements for triggered monitoring will be detailed in the Monitoring and Quality Management Plans. The Monitoring plan will also detail the procedures for review and sign-off.

A detailed site initiation visit with training will be performed at each study site by staff from the MRC CTU as well as African staff who will be specifically trained for this role. The site initiation visits will include training in the administration and side effects of study drugs, as well as the trial procedures.

Monitoring and site training will be carried out at least twice a year at each site by MRC CTU staff or Africa-based monitors. The monitoring will adhere to the principles of ICH GCP. The CRFs of all patients enrolled will be reviewed at the first monitoring visit. A sample of no less than 5 patients will be selected by MRC CTU for review at subsequent visits. Priority will be given to selecting patients for whom the CRFs have not been previously monitored.

Monitors will:

- verify completeness of Trial Master File
- confirm adherence to protocol
- review eligibility verification and consent procedures
- look for missed clinical event reporting
- verify completeness, consistency and accuracy of data being entered on CRFs
- evaluate drug accountability
- provide additional training as needed

The monitors will require access to all patient medical records including, but not limited to, laboratory test results and prescriptions. The investigator (or delegated deputy) should work with the monitor to ensure that any problems detected are resolved.

8.3.1 Direct Access to Patient Records

Participating investigators should agree to allow trial-related monitoring, including audits, ethics committee review and regulatory inspections by providing direct access to source data and documents as required. Patients' consent for this must be obtained. Such information will be treated as strictly confidential and will in no circumstances be made publicly available.

The following data should be verifiable from source documents:

- all signed consent forms
- dates of visits including dates specimens were taken and processed in the laboratory
- eligibility and baseline values for all participants
- all clinical endpoints
- all serious/severe adverse events
- an ongoing random 5% sample of routine patient clinical and laboratory data
- drug compliance
- dates drug dispensed and (if necessary) drugs returned
- pharmacy/clinic drug logs
- concomitant medication.

8.3.2 Confidentiality

The investigator must assure that patients' anonymity will be maintained and that their identities are protected from unauthorised parties. Patients will be assigned a trial identification number and this will be used on CRFs; patients will not be identified by their name. The investigator will keep securely a patient trial register showing identification numbers, names and date of birth. This unique trial number will identify all laboratory specimens, case record forms, and other records and no names will be used, in order to maintain confidentiality. All records will be kept in locked locations. Clinical information will not be released without written permission, except as necessary for monitoring by the trial monitors.

9 STATISTICAL CONSIDERATIONS

9.1 METHOD OF RANDOMISATION

Randomisation will be stratified by centre, age (adults and adolescents aged 13 years or more vs children aged 5-12 years) and the other randomisations in the factorial design; that is, effectively each participant will be randomised 8-ways (2x2x2). This is to ensure balance for the other randomisations in each separate randomisation - if there were 3 separate randomisation lists for A, B, C by chance randomised groups for C could be imbalanced with regard to the C randomisation. The randomisation list will be prepared by staff at the MRC CTU under the direction of the trial statistician, separately for each centre and further stratified within centre by age (5 to 12 years; 13 years and older) using permuted blocks with variable block sizes. The generated randomisation lists will be securely incorporated within the web-enabled trial database, and allocation concealed until the point of the next randomisation. A single member of staff at each site who is not directly involved in patient care will be responsible for carrying out the randomisation process using a secure electronic system within the trial database. A reliable manual back-up system will also be available. Randomisation will not take place until after informed consent has been given and the participant is ready to receive therapy.

9.2 OUTCOME MEASURES

The primary outcome measure will be all-cause mortality to 24 weeks, because this is of major importance to patients, their families and policymakers. Interventions which do not benefit mortality are less likely to be highly cost-effective which is a key determinant for health systems in resource-limited settings given multiple competing priorities. The primary analysis will only include time through 24 weeks in the time-to-event analysis as this is the period when the greatest differences between groups are likely to occur.

The secondary outcomes will be
- 48 week mortality (all-cause)
- safety through 48 weeks
    - serious adverse events
    - grade 4 adverse events
    - adverse events leading to modification of ART or other study drugs
- endpoints relating to the specific mechanisms of action of each intervention
    - anti-HIV: changes in CD4 cell count through 48 weeks
    - anti-infection: incidence of TB, cryptococcal and candida disease, severe bacterial infections through 48 weeks
    - anti-malnutrition: changes in weight and BMI, and growth in children, through 48 weeks
- hospital inpatient episodes and total days admitted through 48 weeks
- adherence to ART and acceptability through 48 weeks

Other outcomes will be
- HIV RNA viral load and resistance
- immune activation (CD3+CD8+CD38+HLA-DR+, CD3+CD8-CD38+HLA-DR+)
- body composition as measured by bioelectrical impedance analysis, BIA

Protection against bias is principally by the use of a completely objective outcome measure (all-cause mortality). Any patient lost to follow-up before 48 weeks without withdrawing consent will be traced for vital status. The primary and secondary mortality comparisons will only be based on data collected during active follow-up (0-48 weeks), but a sensitivity analysis will consider longer-term mortality to investigate whether or not any early differences between randomised groups remain.

Cause of death (and other clinical secondary endpoints) will be adjudicated by an Endpoint Review Committee, blinded to randomised allocations: the ERC will be asked to adjudicate a relationship to all possible interventional drugs (integrase inhibitor, anti-infectives and trial RUSF) to avoid providing them with details of drugs actually received which would unblind them.

BMI and weight will be analysed as absolute values in those aged 13 years and old, and as BMI-for-age and weight-for-age z-scores in children and adolescents (with sensitivity analysis including adults with z-score calculated for age 18 years).

9.3 SAMPLE SIZE

1800 adults and children provides at least 80% power to detect a 50% relative reduction in 24 week all-cause mortality from 7% to 3.5%, or a 60% relative reduction from a lower mortality of 5% to 2% (2 sided alpha=0.05) allowing 5% lost to follow-up by 24 weeks, and incorporating a single inflation factor[71] to allow for the factorial design (rates in multiple groups should be lower than that in any single group). REALITY will test 3 interventions; for the sample size assumption we assume that one of these will be ineffective and will not therefore impact sample size. For 90% power, the detectable reductions are 7% to 3% and 6% to 2.2% respectively.

Whilst we expect more patients to be recruited with the lowest CD4s, even if approximately equal numbers are recruited with CD4 cell counts between 0-49 and 50-99, overall all-cause mortality to 24 weeks would be ~5%; we consider that a 60% relative reduction (to 2%) is clinically meaningful given the additional logistical impact of introducing different interventions at ART initiation in national ART programmes.

If some patients (~10%) are already receiving isoniazid/fluconazole prophylaxis or trial RUSF at randomisation, the effective sample sizes for these interventions will be reduced, but the study design will retain >80% power to detect slightly smaller reductions from 7% to 3% (57% reduction).

600 children aged 5-12 years would also provide at least 80% power to detect a 0.24 greater absolute increase in weight-for-age from 0 to 24 weeks (SD 1.0[17], 2 sided alpha=0.05) allowing 10% died/lost to follow-up by 24 weeks (>90% power for a 0.28 greater absolute increase in weight-for-age). 400 children provide at least 80% power to detect a 0.29 greater absolute increase in weight-for-age from 0 to 24 weeks.

Sample size assumptions are based on a generous 5% lost to follow-up by 24 weeks. As the trial is relatively short (48 weeks follow-up) with interventions focussed around ART initiation, we expect lower loss to follow-up than this worst-case scenario. Loss to follow-up in DART and ARROW trials conducted in some of the same centres was 1.1% and 0.9% respectively at 48 weeks.

9.4 INTERIM MONITORING & ANALYSES

A DMC Charter will be drawn up that describes the membership of the DMC, relationships with other committees, terms of reference, decision-making processes, and the timing and frequency of interim analyses (with a description of stopping rules and/or guidelines). The DMC will meet within 6 months after the trial opens; although the DMC will in general meet annually, the frequency of subsequent meetings will be determined by the DMC and could be more frequent if they deem necessary. The DMC can recommend premature closure or reporting of the trial, or that recruitment to any research group be discontinued or modified. Such recommendations would be made if, in the view of the DMC, there is proof beyond reasonable doubt that one of the allocated strategies is better than its comparator in terms of a difference of clinically significant magnitude in a primary outcome. The guiding statistical criteria for "proof beyond reasonable doubt" is a Haybittle-Peto type rule based on the 99.9% confidence interval of the relative hazard of death in each interim analysis. See Section 14.4 - Data Monitoring Committee (DMC), p81 for details on membership.

9.5 POTENTIAL FOR INTERACTIONS BETWEEN THE TRIAL INTERVENTIONS

Because the three interventions above are approaching different mechanisms for reducing early all-cause mortality on ART (anti-HIV, anti-infection, anti-malnutrition/malabsorption), important interactions between randomised groups are unlikely. Furthermore, any interactions that do exist are likely to be quantitative (slightly smaller/larger effects) rather than qualitative (effect on one background, no effect on another).

The most plausible possible quantitative interactions are:

1. enhanced OI prophylaxis itself improves appetite and thus weight gain as much as the provision of trial RUSF through the prevention of oral/oesophageal candidiasis, TB and treatment of worms (although whether such patients would have access to additional calories is unclear, and whether this would similarly impact mortality is unknown).

2. greater antiretroviral efficacy and also greater potential for side-effects of nevirapine would occur with enhanced OI prophylaxis because fluconazole (at least at treatment doses) may increase nevirapine levels. However, no studies in adults or children have ever identified an association between higher plasma levels of nevirapine and toxicity, suggesting that this is probably driven by patient-level factors (ie pharmacogenetics). In the case of drug toxicity, fluconazole, as an additional intervention, would be stopped first.

No other drug-drug interactions have been identified between the study interventions.

The DMC will consider evidence for interactions at each of their meetings, and may make recommendations to the TSC on the basis of any findings.

9.6 ANALYSIS PLAN (BRIEF)

The analyses will be described in detail in a full Statistical Analysis Plan. This section summarises the main issues.

Each intervention is hypothesised to be superior to standard of care, and therefore the proposed analysis is intention to treat, including all randomised patients with all participants analysed according to the study group to which they were randomised. Primary analyses will use time-to-

event methods (Kaplan-Meier plots, Cox proportional hazards regression) censoring all participants at 24 weeks, stratified by randomisation stratification factors (which include the other randomisations in the factorial).

Pre-specified subgroup analyses would include each of the other randomised allocations (ie exploration of interactions in the factorial design), together with the other randomisation stratification factors (age 5-12 vs 13+ years; and centre). We would also a priori investigate whether there was any evidence for a different impact of the interventions depending on baseline CD4 (0-24, 25-49, 50-99 cells/mm^3), backbone NRTI in the initial ART regimen, by TST status (positive vs negative), and by BMI<20 (adults) or weight-for-age<-1 (children).

Secondary outcome measures will be analysed using time-to-event methods or normal linear regression for continuous variables. The frequency of hospital admissions and adverse events will be tabulated by body systems and by randomised groups, and the number of events experienced by each participant will be compared across randomised groups using Fisher's exact test.

For the within-trial analysis, the differential cost of the treatment interventions will be related to their differential outcomes in terms of the primary outcome. The relative cost-effectiveness of the alternative forms of management will then be assessed using standard decision rules and a full stochastic analysis will be undertaken. A cost-utility analysis will also be conducted using a standard approach. The within-trial analysis will be augmented by extrapolation beyond the trial follow-up using decision-analytic modelling. The aim of this analysis will be to predict the implications of any difference in clinical endpoints in the trial for subsequent quality-adjusted survival duration and long-term resource costs. This will inform the question of whether any differences in drug costs between the treatment groups are offset by reduction in other treatment costs or health improvements in the long-term.

10 ANCILLARY STUDIES

10.1 HEALTH ECONOMICS (ALL PARTICIPANTS)

The economics substudy will include cost-effectiveness analyses (CEA) of the trial interventions. This will involve an evidence synthesis and modelling exercise of different treatment strategies for ART, and will build upon previous CEAs (of the DART, CHAPAS and ARROW trials). Resource use data will be collected in this trial and will be supplemented by data collected in previous work.

The economic evaluation will be conducted from the health services perspective. Costs will cover the use of medication and laboratory tests as well as hospital, primary care and community health services. Unit costs will be attached to resource use, using the best available estimates of long run marginal opportunity cost, to obtain a cost per patient over the period of follow-up. Routinely available national unit costs will be used where possible with local estimations where necessary. There will also be a budget impact analyses of the consequences of adopting the interventions on the health sector budgets, in each of the countries of the trial.

10.2 MOLECULAR DIAGNOSTICS (ALL PARTICIPANTS WITH ACUTE ILLNESSES AND STORED SPECIMENS)

A major question that has hindered identification of relevant interventions for this patient population to date is what are the underlying causes of morbidity and mortality in patients initiating ART with advanced immunodeficiency. Whilst the REALITY randomised comparisons will provide data to support particular mechanisms, stored samples also provide a valuable resource for investigating new methods of molecular diagnostics.

Two molecular methods are being used increasingly in research and clinical practice to identify bacteria. 16S ribosomal DNA (16S rDNA), common to all species of bacteria, can be detected with a broad-range polymerase chain reaction (PCR); specific quantitative PCR (qPCR) can also be used to quantify the 16S rDNA subunit to measure directly the number of bacteria. However broad range 16S rDNA PCR is subject to artefact from endogenous and exogenous bacterial products[72-74] and therefore without either sequencing the PCR product, or carrying out more sensitive qPCR, there is concern that changes in the qPCR may not be due to circulating organisms. Unfortunately sequencing the 16S rDNA has so far yielded results compatible with environmental contamination rather than recognised gut commensals[75-77]. The use of specific primers renders qPCR less vulnerable to background contaminants than broad-range 16S rDNA PCR, and therefore more sensitive. The disadvantage of qPCR is the need to predict which bacterial species are likely to be relevant.

The goal of this study would be to identify the role of bacteria in early mortality on ART. The design would be a case-control study, matching every participant who dies during the first 48 weeks on ART (n=90-130) to an age and centre-matched control. Previous studies have shown comparable results between frozen EDTA plasma and whole blood, and so we would assay standard 16S rDNA PCR, and a panel of 10 qPCR reactions (including Enterobacteriacae, 6 anaerobes, Streptotoccus pneumoniae, Staphylococcus aureus and group A streptococcus) in plasma samples taken at enrolment, and closest before death (or the corresponding visit week in controls) in all cases and controls.

10.3 GENOTYPIC AND PHENOTYPIC CHARACTERISATION OF PATHOGEN ISOLATES

The enhanced prophylaxis randomisation B includes giving additional prophylactic antibiotics (azithromycin), anti-fungals (fluconazole) and anti-tuberculosis drugs (isoniazid). Whilst restricting use of these prophylactics to 12 weeks should reduce the risk of resistance occurring, comparing the genotypes and phenotypes of the key pathogens occurring in severely immuno-compromised adults, adolescents and older children initiating ART will be important to reassure Ministries of Health that any use outside the trial is unlikely to have public health consequences. In particular, given their likely frequency in the trial, we will conduct a genotypic and phenotypic analysis of all invasive bacterial isolates (particularly the pneumococci and salmonella) across the sites (led by Prof Heydernann, Malawi), and will also conduct fluconazole resistance testing on cryptococcal isolates. These comparisons would focus on markers of resistance, but also molecular epidemiology. Other groups of pathogen isolates would also be considered depending on how often they occur during the trial.

10.4 SOCIAL SCIENCE SUBSTUDY

A social science substudy will investigate two major areas relating to the proposed interventions. First, even if the interventions reduce mortality, they may not be widely acceptable in the community if they make patients feel ill when starting ART, and poorer adherence could have long-term consequences. Perceptions about receipt of large numbers of potentially stigmatising pills that make patients feel ill could also prevent patients presenting for treatment. We will therefore explore participants' experiences around pill burden when starting ART, and particularly increased pill burden from taking additional drugs in relation to feelings of increased well-being on ART. Second, we will explore issues around "where does additional RUSF actually go?", particularly in areas of low food security. See Appendix -XI Social Science Substudy for further details.

11 REGULATORY & ETHICAL ISSUES

11.1 COMPLIANCE

The trial end is 48 weeks after the last patient is randomised (end of follow-up for the last randomised patient) and after all data queries have been resolved.

11.1.1 Regulatory Compliance

The trial complies with the principles of the Declaration of Helsinki (2008).

It will also be conducted in compliance with the approved protocol, the principles of Good Clinical Practice (GCP) and the UK Data Protection Act (DPA number: Z5886415).

11.1.2 Centre Compliance

The centres will comply with the principles of GCP as laid down by the ICH topic E6 (Note for Guidance on GCP), and applicable national regulations. An agreement will be in place between each centre and the MRC CTU, setting out respective roles and responsibilities (see Section 13 – Finance, p79).

The centre will inform the Trials Unit as soon as they are aware of a possible serious breach of compliance, so that the Trials Unit can report this breach to the trial sponsor with onward reporting to Ethics and Regulatory bodies as necessary. For the purposes of this regulation, a 'serious breach' is one that is likely to affect to a significant degree:

- The safety or physical or mental integrity of the subjects in the trial, or
- The scientific value of the trial

11.1.3 Data Collection & Retention

CRFs, clinical notes and administrative documentation should be kept in a secure location (for example, locked filing cabinets in a room with restricted access) and held for 15 years after the end of the trial. During this period, all data should be accessible to the competent or equivalent authorities, the Sponsor, and other relevant parties with suitable notice.

11.2 ETHICAL CONDUCT OF THE TRIAL

11.2.1 Ethical Considerations

As described above, the main risks to the patients are from the additional interventions, in particular the risk of additional toxicity from extra drugs (integrase inhibitors or enhanced prophylaxis), and the risk that additional toxicity or pill burden could impair adherence to ART which could lead to increased risk of ART failure in the longer term. All the drugs used for OI prophylaxis and the trial RUSF food supplementation are in widespread use in clinical practice, and their toxicity profiles are well known. Raltegravir is a new drug, but has been used in adults worldwide and in children in the US and Europe.

The trial will directly evaluate whether these potential risks are outweighed by improved survival.

The reason for using an integrase inhibitor as the fourth 'induction drug' is because this drug class has the property of reducing viral load much more rapidly after ART initiation than all the other

antiretroviral drug classes. Our hypothesis is that the high viral load per se in those initiating ART with low CD4 contributes to early mortality and if viral load is reduced rapidly, reduced mortality may follow. The other drug class not used in standard first-line therapy is the protease inihbitor (PI) class. Using a PI would be unlikely to be beneficial as it has been demonstrated that PI and NNRTI drugs both reduce viral load at similar rates. Of note, in the FORTE 4-drug induction-maintenance trial, use of an additional PI drug improved long term but not short-term viral load suppression[78]; therefore it is difficult to see what additional benefit on early morbidity/mortality a short-term PI could add. Whilst integrase inhibitors are currently costly, this is likely to change over the next 5 years with many antiretrovirals being priced at cost for resource-limited settings. However, an integrase inhibitor would only be used for 12 weeks - if beneficial in reducing mortality, this might well be cost-effective, hence the need for economic analysis.

We would also not expect addition of an integrase inhibitor to ART for 12 weeks to lead to early resistance to this drug class. However, to confirm this viral load and resistance will be measured in a substudy on retrospectively collected stored plasma samples using a 'walk-back' system i.e. identify viral load failures at the end of the trial and test these first for resistance. This would be efficient and less costly.

11.2.2 SAFETY PROFILE OF RALTEGRAVIR

Raltegravir has been taken by large numbers of adults in phase III trials, and is licensed for the treatment of HIV infection in adults and widely used in most resource-rich countries. An application for paediatric licensing has been submitted to the FDA. However, at the present time there is limited experience with the use of this drug in the African population, although three of the four REALITY trial centres have been using raltegravir in adults and adolescents enrolled in the EARNEST trial. Standard procedures are in place to record serious adverse events arising from the use of raltegravir (or any study drug) in the REALITY trial, and such events will be evaluated and reported to ethics committees and regulatory agencies within specified timelines. The DMC will closely monitor serious adverse events occurring during the trial in all treatment groups and will be able to advise appropriate action if there is a pattern of serious toxicity emerging from one or more of the drugs.

11.2.3 BURDEN OF INVESTIGATIONS

The study visits coincide with the usual frequency of visits for routine clinical care and the amount of extra blood taken is modest.

11.2.4 POST-TRIAL TREATMENT

The drugs used in this trial are already licensed or, in the case of raltegravir for children, are likely to be licensed imminently. However, raltegravir and other interventions are given only for the first 12 weeks, that is patients will be receiving standard of care when they leave the REALITY trial at 48 weeks.

The trial investigators will negotiate with the Ministry of Health in each of the participating countries and obtain an undertaking from them that they would support the standard ART requirements of the study participants after the trial ends. An agreement covering post-trial treatment must be in place with each of the national ministries prior to commencing the study in that country. The contract with supporting pharmaceutical companies will specify that the supply of study drugs would be limited to the duration of the trial in each individual patient, although provision may be available for a short term extension to cover the period while the patient makes the transition to the national treatment programme.

11.2.5 INFORMING POTENTIAL TRIAL PARTICIPANTS OF POSSIBLE BENEFITS AND KNOWN RISKS

Participants will be informed fully of known risks and possible benefits by means of a patient information sheet (Appendix I - Template Patient information sheets, p91) and this will be reinforced by discussions with the trial research teams at the individual sites prior to enrolment.

11.2.6 CONFIDENTIALITY

Patients' confidentiality will be maintained throughout the trial. Data submitted to MRC CTU and samples sent to central testing facilities will be identified only by the trial number and patient initials.

11.2.7 ETHICAL APPROVALS

Before initiation of the trial at each clinical centre, the protocol, all informed consent forms and information materials to be given to the prospective participant will be submitted to each ethics committee for approval. Any further amendments will be submitted and approved by each ethics committee.

The rights of the participant to refuse to participate in the trial without giving a reason must be respected. After the participant has entered into the trial, the clinician must remain free to give alternative treatment to that specified in the protocol, at any stage, if he/she feels it to be in the best interest of the participant. The reason for doing so, however, should be recorded; the participant will remain within the trial for the purpose of follow-up and for data analysis by the treatment option to which they have been allocated. Similarly, the participant must remain free to change their mind at any time about the protocol treatment and trial follow-up without giving a reason and without prejudicing his/her further treatment.

11.3 COMPETENT AUTHORITY APPROVALS

This protocol will be submitted to the national competent or equivalent authority, as appropriate in each country where the trial will be run.

The progress of the trial and safety issues will be reported to the regulatory agency or equivalent in accordance with local requirements and practices in a timely manner.

Safety reports will be submitted to the competent authority in accordance with each authority's requirements in a timely manner.

12 INDEMNITY

University College London holds insurance against claims from participants for injury caused by their participation in the clinical trial. Participants may be able to claim compensation if they can prove that UCL has been negligent. However, as this clinical trial is being carried out in a research facility/clinic, the research facility/clinic continues to have a duty of care to the participant of the clinical trial. University College London does not accept liability for any breach in the research facility/clinic's duty of care, or any negligence on the part of research facility/clinic employees. Participants may also be able to claim compensation for injury caused by participation in this clinical trial without the need to prove negligence on the part of University College London or another party. Participants who sustain injury and wish to make a claim for compensation should do so in writing in the first instance to the Chief Investigator, who will pass the claim to UCL's Insurers, via the MRC CTU at UCL.

Research facility/clinics selected to participate in this clinical trial shall provide clinical negligence insurance cover for harm caused by their employees and a copy of the relevant insurance policy or summary shall be provided to University College London, upon request.

13 FINANCE

The trial is supported by grant funding from the Joint Global Health Trials Scheme of the Department for International Development, UK (DFID), the Wellcome Trust and the Medical Research Council (MRC UK). Additional funding support is provided by the PENTA foundation.

The trial will be coordinated by the MRC CTU. A written agreement with the site principal investigator and the MRC CTU will outline the funding arrangements to sites.

A finance committee will be chaired by the independent TSC chair. This will meet and review the financial aspects of the trial at least 12-monthly and report to the sponsor. Terms of reference will be developed for this committee, whose membership will include PIs and their financial advisors.

14 OVERSIGHT & TRIAL COMMITTEES

There are a number of committees involved with the oversight of the trial. These committees are detailed below and the relationship between them is expressed in the figure.

14.1 SITE TRIAL MANAGEMENT TEAMS

A Trial Management Team will be formed at each site to conduct the day-to-day management of the trial at the site ("Site TMT"). This will include the investigators and trial staff at the site. These groups will meet every one to two weeks and will be chaired by the Principal Investigator or Co-Principal Investigator at the site. The group will discuss issues related to the progress of the trial at the site and to ensure that the trial is running well.

There will be a similar trial management team formed to conduct the day-to-day management of the trial at the MRC ("MRC TMT"). This will include the Chief Investigator, trial statistician, trial physician, clinical project manager, trial manager and data manager. The group will meet at least once per month, although may meet more often if required.

14.2 TRIAL MANAGEMENT GROUP (TMG)

A Trial Management Group (TMG) will be formed comprising the Chief Investigator; centre Principal Investigators, co-investigators and Trial Managers, other lead investigators (clinical and non-clinical) and members of the MRC Clinical Trials Unit (CTU). The TMG will be responsible for the day-to-day running and management of the trial. It will meet approximately once a year in-person and will hold a regular teleconference at approximately monthly intervals at which sites will summarise progress and challenges and bring up for discussion any difficulties, as well as discuss and decide matters of general importance for the trial. This group will be chaired by the Chief Investigator and all decisions regarding the overall running of the trial will be made in this forum with the exception of matters of fundamental importance to the viability of the trial or that require major changes to the protocol. These will be referred to the Trial Steering Committee (TSC). The full details can be found in the TMG Charter.

14.3 TRIAL STEERING COMMITTEE (TSC)

The Trial Steering Committee (TSC) has membership from the TMG plus independent members, including the Chair. The role of the TSC is to provide overall supervision for the trial and provide advice through its independent Chair. The ultimate decision for the continuation of the trial lies with the TSC. Further details of TSC functioning are presented in the TSC Charter.

Prof Ian Weller (London) will Chair of the REALITY Trial Steering Committee; Prof Weller has substantial experience as Chair of the DART, ARROW and EARNEST Trial Steering Committees. Two independent community members of the Trial Steering Committee will represent patient advocate groups; Ms Effie Malianga from Zimbabwe and Ms Flavia Miiro from Uganda. They would have input throughout all stages of the trial, including liaison with the HIV community. For the three other independent members of the TSC, we propose the following: Dr Charles Mwansambo (Permanent Secretary of Health, MOH, Malawi, also on ARROW TSC); Dr Peter Elyanu (Program Officer Paediatric HIV/AIDS Care, MOH, Uganda); Dr Elizabeth Bukusi (Program Co-Director for a PEPFAR–CDC funded

care program, “Family AIDS Care and Education Services (FACES)”, Kenya). The five country PIs for REALITY (Gibb, Hakim, Etyang, Mugyenyi, Mallewa) will constitute the remaining members (total 10, 5 independents).

Each centre would either use their existing Community Advisory Board (CAB) or form a specific patient liaison group who would be responsible for liaising with the patient representatives on the TSC, would feedback concerns and questions from the community and also hear about the latest developments in the trial and the wider scientific community. All the centres currently have active patient participation groups.

14.4 DATA MONITORING COMMITTEE (DMC)

An independent Data Monitoring Committee (DMC) will be formed. The DMC will be the only group who sees the confidential, accumulating data for the trial separately by randomised group. Reports to the DMC will be produced by the MRC CTU statisticians. The DMC will review trial data on recruitment, safety, adherence to randomised strategies and efficacy, as well as consider findings from any other relevant studies. The DMC will advise the TSC. Further details of DMC functioning and the procedures for interim analysis and monitoring are provided in the DMC Charter.

The Chair of the REALITY DMC will be Prof Tim Peto. Other independent members will be Prof Philippa Musoke (Mulago Hospital, Uganda), Prof Jon Matenga (University of Harare, Zimbabwe) and Dr Sam Phiri (Lighthouse Trust,Malawi).

14.5 OTHER COMMITTEES

14.5.1 ENDPOINT REVIEW COMMITTEE

An Endpoint Review Committee will be appointed whose remit will be to determine the validity of potential clinical endpoints in terms of meeting the standard criteria, as defined by the protocol (Appendix IX - Clinical Progression and WHO Definitions, p125). It will have an independent Chair and will include Project Leaders from each site as well as other independent clinicians. No member will review endpoints from their own site. Terms of reference for the Endpoint Review Committee will be drawn up.

14.6 ROLE OF STUDY SPONSOR

The MRC is the Sponsor of REALITY and delegates this responsibility to the MRC CTU to oversee the implementation of the study by ensuring that arrangements are put into place for adequate management, monitoring, analysis and reporting of the trial.

Figure 14.1 Trial Organogram

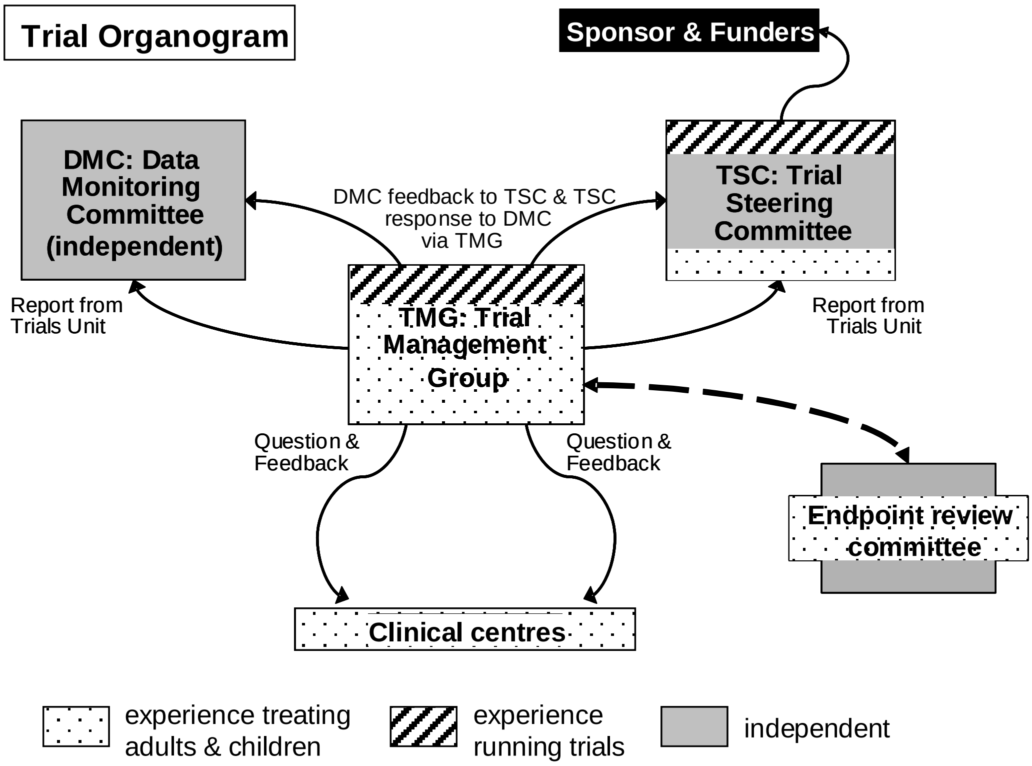

15 PUBLICATION

The REALITY TSC is the custodian of the data and specimens generated from the REALITY trial; REALITY trial data are not the property of individual participating investigators or health care facilities where the data were generated.

It is anticipated that a number of opportunities will arise for publication during the course of and following completion of the REALITY trial. Publications includes papers (including abstracts) for presentation at national and international meetings, as well as the preparation of manuscripts for peer-reviewed publication. In order to avoid disputes regarding authorship, it is important to establish a consensus approach that will provide a framework for all publications derived in full or in part from this clinical trial. The following approach is derived from the *Lancet* and from the publication policies used in other MRC clinical trials:

- All publications are to be approved by the TMG and TSC before submission for publication. Any publication arising before the end of the trial (not by randomised groups) will also be approved by the DMC in order to ensure that the primary objective of the trial (the randomised comparison) is not compromised. In particular, no analyses by randomised group of any outcome (primary, secondary or other) in either the main trial or associated substudies will be conducted or presented before the end of the trial, other than those for interim review by the DMC. The TMG and TSC will resolve problems of authorship and maintain the quality of publications.

- In line with MRC policy that the results of publicly-funded research should be freely available, manuscripts arising from the trial will, wherever possible, be submitted to peer-reviewed journals which enable Open Access via UK PubMed Central (PMC) within six months of the official date of final publication. All conference presentations will be made available as soon as possible after the event via the REALITY website. All publications will acknowledge the trial's funding sources.

- For all publications, the TMG will nominate a chairperson or approve an individual's request to chair a manuscript writing committee. The chair will usually be the primary or senior author. The chairperson is responsible for identifying fellow authors and for determining with that group the order of authorship that will appear on the manuscript. The TSC will resolve any problems of authorship and maintain the quality of publications.

- The TMG will maintain a list of investigators to be presented in an appendix at the end of the paper. This list will include investigators who contributed to the investigation being reported but who are not members of the writing committee. In principle, substudy reports should include all investigators for the main study, although in some instances where a smaller number of investigators have made any form of contribution, it may be appropriate to abbreviate the listing. All headline authors in any publication arising from the main study or sub-studies must have a made a substantive academic or project management contribution to the work that is being presented. "Substantive" must be defined by a written declaration of exactly what the contribution of any individual is believed to have been. In addition to fulfilling the criteria based on contribution, additional features that will be considered in selecting an authorship group will include the recruitment of patients who contributed data to any set of analyses contained in the manuscript and/or the conduct of analyses (laboratory and statistical), leadership and coordination of the project in the absence of a clear academic contribution.

- The data derived from this clinical trial are considered the property of the REALITY TSC. The presentation or publication of any data collected by the participating investigators on patients entered into this trial is under the direct control of the TMG and TSC (and the DMC before the end of the trial). This is true whether the publication or presentation is concerned directly with the results of the trial or is associated with the trial in some other way. However, although individual participating investigators will not have any inherent right to perform analyses or interpretations or to make public presentations or seek publication of any of the data other than under the auspices of and with the approval of the TMG and TSC (and the DMC before the end of the trial), they will be encouraged to develop sub-studies or propose analyses subject to the approval by the TMG and TSC (and the DMC before the end of the trial). Any requests for access to raw data will be welcomed as long as they are scientifically valid and do not conflict with the integrity of the trial or ongoing analyses by the trial team

- Outcome data by randomised group will not be revealed to the participating investigators until the data collection phase and primary full analysis of the trial has been completed. This policy safeguards against possible bias affecting the data collection. The DMC will be monitoring the outcome results and may recommend that the trial be stopped for safety reasons or if a definitive answer is reached earlier than the scheduled end of the trial.

16 PROTOCOL AMENDMENTS

Amendment 1: V1.01 04 Apr 2012
Amendment 2: V1.02 06 Dec 2013

17 REFERENCES

1. Braitstein P, Brinkhof MW, Dabis F, Schechter M, Boulle A, Miotti P, et al. Mortality of HIV-1-infected patients in the first year of antiretroviral therapy: comparison between low-income and high-income countries. Lancet. 2006; **367**(9513): 817-24.
2. Cornell M, Grimsrud A, Fairall L, Fox MP, van Cutsem G, Giddy J, et al. Temporal changes in programme outcomes among adult patients initiating antiretroviral therapy across South Africa, 2002-2007. AIDS (London, England). 2010.
3. Walker AS, Mugyenyi P, Munderi P, Hakim J, Kekitiinwa A, Katabira E, et al. Early mortality following ART initiation in HIV-infected adults and children in Uganda and Zimbabwe. Tenth International Congress on Drug Therapy in HIV Infection. 7-11 November 2010 Glasgow, UK; 2010.
4. World Health Organisation. Towards universal access: Scaling up priority HIV/AIDS interventions in the health sector, progress report 2009. . Geneva: World Health Organisation; 2009.
5. Abdool Karim SS, Naidoo K, Grobler A, Padayatchi N, Baxter C, Gray A, et al. Timing of initiation of antiretroviral drugs during tuberculosis therapy. The New England journal of medicine. 2010; **362**(8): 697-706.
6. Abdool Karim SS, Naidoo K, Grobler A, Padayatchi N, Baxter C, Gray AL, et al. Integration of antiretroviral therapy with tuberculosis treatment. The New England journal of medicine. 2011; **365**(16): 1492-501.
7. Blanc FX, Sok T, Laureillard D, Borand L, Rekacewicz C, Nerrienet E, et al. Earlier versus later start of antiretroviral therapy in HIV-infected adults with tuberculosis. N Engl J Med. 2011; **365**(16): 1471-81.
8. Havlir DV, Kendall MA, Ive P, Kumwenda J, Swindells S, Qasba SS, et al. Timing of antiretroviral therapy for HIV-1 infection and tuberculosis. N Engl J Med. 2011; **365**(16): 1482-91.
9. Grinsztejn B, Nguyen BY, Katlama C, Gatell JM, Lazzarin A, Vittecoq D, et al. Safety and efficacy of the HIV-1 integrase inhibitor raltegravir (MK-0518) in treatment-experienced patients with multidrug-resistant virus: a phase II randomised controlled trial. Lancet. 2007; **369**(9569): 1261-9.
10. Markowitz M, Morales-Ramirez JO, Nguyen BY, Kovacs CM, Steigbigel RT, Cooper DA, et al. Antiretroviral activity, pharmacokinetics, and tolerability of MK-0518, a novel inhibitor of HIV-1 integrase, dosed as monotherapy for 10 days in treatment-naive HIV-1-infected individuals. J Acquir Immune Defic Syndr. 2006; **43**(5): 509-15.
11. Lennox JL, DeJesus E, Lazzarin A, Pollard RB, Madruga JV, Berger DS, et al. Safety and efficacy of raltegravir-based versus efavirenz-based combination therapy in treatment-naive patients with HIV-1 infection: a multicentre, double-blind randomised controlled trial. Lancet. 2009; **374**(9692): 796-806.
12. DHHS Panel on Antiretroviral Guidelines for Adults and Adolescents — A working group of the Office of AIDS Research Advisory Council (OARAC). Guidelines for the Use of Antiretroviral Agents in HIV-1-Infected Adults and Adolescents. 2006 [cited; Available from: http://aidsinfo.nih.gov/ContentFiles/AdultandAdolescentGL.pdf
13. Wiznia A, Samson P, Acosta E, Teppler H, Sheeran E, Graham B, et al. Safety and efficacy of raltegravir in pediatric HIV Infection: preliminary analysis from the IMPAACT Group. 16th Conference on Retroviruses and Opportunistic Infections. Montreal, 8-11th February; 2009, Abstract 874.
14. Nachman S, Acosta E, Zheng N, Teppler H, Homony B, Fenton T, et al. Interim Results from IMPAACT P1066: RAL Oral Chewable Tablet Formulation for 2- to 5-Year-olds 18th Conference on Retroviruses and Opportunistic Infections. Boston, US; 27 February-2 March. Abstract 715; 2011.

15. Haubrich RH, Riddler SA, Ribaudo H, Dirienzo AG, Klingman KL, Garren KW, et al. Initial Viral Decay to Assess the Relative Antiretroviral Potency of PI-, NNRTI- and NRTI-Sparing Regimens for First Line Therapy of HIV Infection. AIDS (London, England). 2011.
16. Grinsztejn B, Veloso VG, Friedman RK, Moreira RI, Luz PM, Campos DP, et al. Early mortality and cause of deaths in patients using HAART in Brazil and the United States. AIDS. 2009; **23**(16): 2107-14.
17. DART and ARROW trials. Personal communication.
18. Walker AS, Ford D, Gilks CF, Munderi P, Ssali F, Reid A, et al. Daily co-trimoxazole prophylaxis in severely immunosuppressed HIV-infected adults in Africa started on combination antiretroviral therapy: an observational analysis of the DART cohort. Lancet. 2010; **375**(9722): 1278-86.
19. World Health Organization. Guidelines on co-trimoxazole prophylaxis for HIV-related infections among children, adolescents and adults : recommendations for a public health approach. . Geneva: World Health Organization; 2006.
20. Badri M, Wilson D, Wood R. Effect of highly active antiretroviral therapy on incidence of tuberculosis in South Africa: a cohort study. Lancet. 2002; **359**(9323): 2059-64.
21. Woldehanna S, Volmink J. Treatment of latent tuberculosis infection in HIV infected persons. Cochrane database of systematic reviews (Online). 2004; (1): CD000171.
22. Zar HJ, Cotton MF, Strauss S, Karpakis J, Hussey G, Schaaf HS, et al. Effect of isoniazid prophylaxis on mortality and incidence of tuberculosis in children with HIV: randomised controlled trial. BMJ (Clinical research ed. 2007; **334**(7585): 136.
23. Whalen CC, Johnson JL, Okwera A, Hom DL, Huebner R, Mugyenyi P, et al. A trial of three regimens to prevent tuberculosis in Ugandan adults infected with the human immunodeficiency virus. Uganda-Case Western Reserve University Research Collaboration. The New England journal of medicine. 1997; **337**(12): 801-8.
24. Mwinga A, Hosp M, Godfrey-Faussett P, Quigley M, Mwaba P, Mugala BN, et al. Twice weekly tuberculosis preventive therapy in HIV infection in Zambia. AIDS (London, England). 1998; **12**(18): 2447-57.
25. Samandari T, Bishai D, Luteijn M, Mosimaneotsile B, Motsamai O, Postma M, et al. Costs and consequences of additional chest x-ray in a tuberculosis prevention program in Botswana. Am J Respir Crit Care Med. 2011; **183**(8): 1103-11.
26. Samandari T, Agizew TB, Nyirenda S, Tedla Z, Sibanda T, Shang N, et al. 6-month versus 36-month isoniazid preventive treatment for tuberculosis in adults with HIV infection in Botswana: a randomised, double-blind, placebo-controlled trial. Lancet. 2011; **377**(9777): 1588-98.
27. WHO. Guidelines for intensified tuberculosis case-finding and isoniazid preventive therapy for people living with HIV in resource-constrained settings. Geneva, Switzerland (http://whqlibdoc.who.int/publications/2011/9789241500708_eng.pdf): WHO; 2011.
28. French N, Gray K, Watera C, Nakiyingi J, Lugada E, Moore M, et al. Cryptococcal infection in a cohort of HIV-1-infected Ugandan adults. AIDS (London, England). 2002; **16**(7): 1031-8.
29. Chang LW, Phipps WT, Kennedy GE, Rutherford GW. Antifungal interventions for the primary prevention of cryptococcal disease in adults with HIV. Cochrane database of systematic reviews (Online). 2005; (3): CD004773.
30. Parkes-Ratanshi R, Kamali A, Wakeham K, Levin J, Nabiryo Lwanga C, Kenya-Mugisha N, et al. Successful primary prevention of cryptococcal disease using fluconazole prophylaxis in HIV-infected Ugandan adults. 16th Conference on Retroviruses and Opportunistic Infections Montreal, Feb 8-11, Abstract 32; 2009.
31. Haddow LJ, Easterbrook PJ, Mosam A, Khanyile NG, Parboosing R, Moodley P, et al. Defining immune reconstitution inflammatory syndrome: evaluation of expert opinion versus 2 case definitions in a South African cohort. Clin Infect Dis. 2009; **49**(9): 1424-32.

32. Amsden GW. Anti-inflammatory effects of macrolides--an underappreciated benefit in the treatment of community-acquired respiratory tract infections and chronic inflammatory pulmonary conditions? J Antimicrob Chemother. 2005; **55**(1): 10-21.
33. Porco TC, Gebre T, Ayele B, House J, Keenan J, Zhou Z, et al. Effect of mass distribution of azithromycin for trachoma control on overall mortality in Ethiopian children: a randomized trial. JAMA. 2009; **302**(9): 962-8.
34. Nahata M. Drug interactions with azithromycin and the macrolides: an overview. J Antimicrob Chemother. 1996; **37 Suppl C**: 133-42.
35. Blish CA, Sangare L, Herrin BR, Richardson BA, John-Stewart G, Walson JL. Changes in plasma cytokines after treatment of ascaris lumbricoides infection in individuals with HIV-1 infection. The Journal of infectious diseases. 2010; **201**(12): 1816-21.
36. Kallestrup P, Zinyama R, Gomo E, Butterworth AE, Mudenge B, van Dam GJ, et al. Schistosomiasis and HIV-1 infection in rural Zimbabwe: effect of treatment of schistosomiasis on CD4 cell count and plasma HIV-1 RNA load. The Journal of infectious diseases. 2005; **192**(11): 1956-61.
37. Nielsen NO, Simonsen PE, Dalgaard P, Krarup H, Magnussen P, Magesa S, et al. Effect of diethylcarbamazine on HIV load, CD4%, and CD4/CD8 ratio in HIV-infected adult Tanzanians with or without lymphatic filariasis: randomized double-blind and placebo-controlled cross-over trial. The American journal of tropical medicine and hygiene. 2007; **77**(3): 507-13.
38. Walson JL, Otieno PA, Mbuchi M, Richardson BA, Lohman-Payne B, Macharia SW, et al. Albendazole treatment of HIV-1 and helminth co-infection: a randomized, double-blind, placebo-controlled trial. AIDS (London, England). 2008; **22**(13): 1601-9.
39. Walson JL, John-Stewart G. Treatment of helminth co-infection in HIV-1 infected individuals in resource-limited settings. Cochrane database of systematic reviews (Online). 2008; (1): CD006419.
40. Walson JL, Herrin BR, John-Stewart G. Deworming helminth co-infected individuals for delaying HIV disease progression. Cochrane database of systematic reviews (Online). 2009; (3): CD006419.
41. Zachariah R, Fitzgerald M, Massaquoi M, Pasulani O, Arnould L, Makombe S, et al. Risk factors for high early mortality in patients on antiretroviral treatment in a rural district of Malawi. AIDS. 2006; **20**(18): 2355-60.
42. Liu E, Spiegelman D, Semu H, Hawkins C, Chalamilla G, Aveika A, et al. Nutritional Status and Mortality Among HIV-Infected Patients Receiving Antiretroviral Therapy in Tanzania. The Journal of infectious diseases. 2011; **204**(2): 282-90.
43. Koethe JR, Chi BH, Megazzini KM, Heimburger DC, Stringer JS. Macronutrient supplementation for malnourished HIV-infected adults: a review of the evidence in resource-adequate and resource-constrained settings. Clin Infect Dis. 2009; **49**(5): 787-98.
44. Ndekha MJ, van Oosterhout JJ, Zijlstra EE, Manary M, Saloojee H, Manary MJ. Supplementary feeding with either ready-to-use fortified spread or corn-soy blend in wasted adults starting antiretroviral therapy in Malawi: randomised, investigator blinded, controlled trial. BMJ (Clinical research ed. 2009; **338**: b1867.
45. Paton NI, Chua YK, Earnest A, Chee CB. Randomized controlled trial of nutritional supplementation in patients with newly diagnosed tuberculosis and wasting. The American journal of clinical nutrition. 2004; **80**(2): 460-5.
46. Lara AM, Wakholi BN, Kasirye A, Munderi P, Watera C, Lalloo DG, et al. Utility assessment of HIV/AIDS-related health states in HIV-infected Ugandans. AIDS (London, England). 2008; **22 Suppl 1**: S123-30.
47. Gandhi RT, Zheng L, Bosch RJ, Chan ES, Margolis DM, Read S, et al. The effect of raltegravir intensification on low-level residual viremia in HIV-infected patients on antiretroviral therapy: a randomized controlled trial. PLoS Med. 2010; **7**(8).

48. Hatano H, Hayes TL, Dahl V, Sinclair E, Lee TH, Hoh R, et al. A randomized, controlled trial of raltegravir intensification in antiretroviral-treated, HIV-infected patients with a suboptimal CD4+ T cell response. The Journal of infectious diseases. 2011; **203**(7): 960-8.
49. World Health Organisation. Antiretroviral therapy for HIV infection in adults and adolescents: Recommendations for a public health approach. 2010 revision: WHO Geneva 2010.
50. Burman W. Personal communication.
51. WHO. Antiretroviral therapy for HIV infection in infants and children: Towards universal access. Recommendations for a public health approach: 2010 revision Geneva; 2010.
52. Zolopa A, Andersen J, Powderly W, Sanchez A, Sanne I, Suckow C, et al. Early antiretroviral therapy reduces AIDS progression/death in individuals with acute opportunistic infections: a multicenter randomized strategy trial. PLoS One. 2009; **4**(5): e5575.
53. Fillekes Q, Natukunda E, Balungi J, Kendall L, Bwakura-Dangarembizi M, Keishanyu R, et al. Pediatric underdosing of efavirenz: a pharmacokinetic study in Uganda. J Acquir Immune Defic Syndr. 2011; **58**(4): 392-8.
54. WHO. Antiretroviral therapy for HIV infection in infants and children: Annex E: Prescribing information and weight-based dosing of available ARV formulations for infants and children (http://www.who.int/hiv/pub/paediatric/paediatric_arv_dosing.pdf). Geneva; 2010.
55. Nachman S, Frenkel LM, Samson P, Acosta E, Teppler H, Fenton T, et al. 24 Week Safety and Efficacy from IMPAACT P1066: A Phase I/II, Multicenter, Open-Label, Noncomparative Study to Evaluate Raltegravir (RAL) in HIV-1 Infected Youth. 49th ICAAC Meeting. San Francisco, CA, US; 12-15 September; 2009.
56. Nachman S, Acosta E, Samson P, Teppler H, Homony B, Fenton T, et al. Pharmacokinetic (PK), safety and efficacy data on Cohort IIA; youth aged 6-11 from IMPAACT P1066: a Phase I/II study to evaluate raltegravir (RAL) in HIV-1 infected youth. 17th Conference on Retroviruses and Opportunistic Infections. San Francisco, US; 16-19 February. Abstract 873; 2010.
57. Nachman S, Acosta E, Samson P, Teppler H, Homony B, Fenton T, et al. Interim Results from IMPAACT P1066: Raltegravir Oral Chewable Tablet Formulation in Children 6 to 11 Years. 17th Conference on Retroviruses and Opportunistic Infections. San Francisco, US; 16-19 February. Abstract LB161; 2010.
58. Markowitz M, Nguyen B-Y, Gotuzzo F. Potent antiretroviral effect of MK-0518, a novel HIV-1 integrase inhibitor as part of combination ART in treatment naive HIV-1 infected patients. Program and abstracts of the XVI International AIDS Conference; Toronto, Canada Abstract THLB0214. 2006.
59. Team TDT. Twenty-four-week safety and tolerability of nevirapine versus abacavir in combination with zidovudine/lamivudine as first-line antiretroviral therapy: a randomised double-blind trial (NORA). Trop Med Int Health. 2008; **13**(1): 6-16.
60. Nahirya-Ntege P, Musiime V, Naidoo B, Bakeera-Kitaka S, Nathoo K, Munderi P, et al. Low incidence of abacavir hypersensitivity reaction among African children initiating antiretroviral therapy. Pediatr Infect Dis J. 2011; **30**(6): 535-7.
61. Koethe JR, Blevins M, Nyirenda C, Kabagambe EK, Shepherd BE, Wester CW, et al. Nutrition and inflammation serum biomarkers are associated with 12-week mortality among malnourished adults initiating antiretroviral therapy in Zambia. J Int AIDS Soc. 2011; **14**: 19.
62. Heimburger DC, Koethe JR, Nyirenda C, Bosire C, Chiasera JM, Blevins M, et al. Serum phosphate predicts early mortality in adults starting antiretroviral therapy in Lusaka, Zambia: a prospective cohort study. PLoS One. 2010; **5**(5): e10687.
63. Cohen DD, Voss C, Taylor MJ, Stasinopoulos DM, Delextrat A, Sandercock GR. Handgrip strength in English schoolchildren. Acta Paediatr. 2010; **99**(7): 1065-72.
64. Koley S, Melton S. Age-related changes in handgrip strength among healthy Indian males and females aged 6-25 years. J Life Sci. 2010; **2**(2): 73-80.

65. DART Trial Team. Routine versus clinically driven laboratory monitoring of HIV antiretroviral therapy in Africa (DART): a randomised non-inferiority trial. Lancet. 2010; **375**(9709): 123-31.
66. Antiretroviral Pregnancy Register. Interim Report [http://www.apregistry.com/forms/interim_report.pdf, accessed 18 January 2011]; 2010.
67. Capparelli EV, et al. Raltegravir pharmacokinetics during pregnancy. 50th Interscience Conference on Antimicrobial Agents and Chemotherapy (ICAAC). 12–15 September 2010, Boston. Poster abstract H-1668a; 2010.
68. Colbers A, Moltó J, Ivanovic J, Taylor G, Branco T, van der Ende I, et al. A comparison of the pharmacokinetics of raltegravir during pregnancy and post-partum. 12th International Workshop on Clinical Pharmacology of HIV Therapy. Miami, Florida, April 13-15, 2011; 2011.
69. CDC. Managing Drug Interactions in the Treatment of HIV-Related Tuberculosis. In: CDC, editor.; 2009.
70. Hanley WD, Wenning LA, Moreau A, Kost JT, Mangin E, Shamp T, et al. Effect of tipranavir-ritonavir on pharmacokinetics of raltegravir. Antimicrobial agents and chemotherapy. 2009; **53**(7): 2752-5.
71. Babiker AG, Walker AS. Statistical issues emerging from clinical trials in HIV infection. In: Geller N, editor. Advances in clinical trial biostatistics: CRC Press; 2003.
72. Harris KA, Hartley JC. Development of broad-range 16S rDNA PCR for use in the routine diagnostic clinical microbiology service. J Med Microbiol. 2003; **52**(Pt 8): 685-91.
73. Millar BC, Xu J, Moore JE. Risk assessment models and contamination management: implications for broad-range ribosomal DNA PCR as a diagnostic tool in medical bacteriology. J Clin Microbiol. 2002; **40**(5): 1575-80.
74. Ferri E, Novati S, Casiraghi M, Sambri V, Genco F, Gulminetti R, et al. Plasma levels of bacterial DNA in HIV infection: the limits of quantitative polymerase chain reaction. J Infect Dis. 2010; **202**(1): 176-7; author reply 8.
75. Marchetti G, Bellistri GM, Borghi E, Tincati C, Ferramosca S, La Francesca M, et al. Microbial translocation is associated with sustained failure in CD4+ T-cell reconstitution in HIV-infected patients on long-term highly active antiretroviral therapy. AIDS. 2008; **22**(15): 2035-8.
76. Merlini E, Bai F, Bellistri GM, Tincati C, d'Arminio Monforte A, Marchetti G. Evidence for Polymicrobic Flora Translocating in Peripheral Blood of HIV-Infected Patients with Poor Immune Response to Antiretroviral Therapy. PLoS One. 2011; **6**(4): e18580.
77. Suau A, Bonnet R, Sutren M, Godon JJ, Gibson GR, Collins MD, et al. Direct analysis of genes encoding 16S rRNA from complex communities reveals many novel molecular species within the human gut. Appl Environ Microbiol. 1999; **65**(11): 4799-807.
78. Asboe D, Williams IG, Goodall RL, Darbyshire JH, Hooker MH, Babiker AG. A virological benefit from an induction/maintenance strategy: the Forte trial. Antivir Ther. 2007; **12**(1): 47-54.
79. Centers for Disease Control and Prevention. 1994 Revised Classification system for human immunodeficiency virus infection in children less than 13 years of age. MMWR. 1994; **43**: 1-17.
80. Centers for Disease Control and Prevention. CDC Growth Charts: United States. Advance Data number 314; December 4, 2000 (revised); 2000.
81. NIH Division of Microbiology and Infectious Diseases (DMID). Pediatric toxicity tables, November 2007: http://www3.niaid.nih.gov/research/resources/DMIDClinRsrch/toxtables; 2007.
82. Consolidated guidelines on the use of antiretroviral drugs for treating and preventing HIV infection. Recommendations for a public health approach. WHO 2013 revision.

APPENDIX I - TEMPLATE PATIENT INFORMATION SHEETS

[Each country to use its own translated Informed Consent for relevant local languages according to local regulatory requirements, on local headed paper for each site]

REALITY (Reduction of EArly mortaLITY in adults and children) trial

Information for Patients
(note: same patient information for carers/parents, with reference to "your child" rather than "you". Simplified shortened version to be used for older children)

Introduction

We are inviting you to take part in a research study called REALITY. It is being carried out in four African countries: Kenya, Malawi, Uganda and Zimbabwe. Before you decide if you want to take part, it is important for you to understand why the research is being done and what it will involve. Please take time to read this information sheet carefully or ask someone to read it to you. Please discuss it with others if you wish. We will give you a copy to keep. Please ask the clinic nurses or counsellors if there is anything that is not clear or if you would like more information. Joining the REALITY study is entirely voluntary. Take time to decide whether or not you wish to take part.

You may decide that you do not wish to take part now or you may wish to withdraw from the study later. These decisions will not influence the care you receive now or in future. We hope that if you decide to join the study but withdraw later, you would give a reason for your decision. More importantly we hope that you would continue to allow us to provide follow-up care which involves continued regular medical checkups, even if you are no longer taking the study medicines.

What is the reason for doing the REALITY study?

HIV is the virus that causes AIDS. At the moment there is no cure for HIV. However there are anti-HIV medicines, called 'anti-retrovirals' or ARVs, which can control HIV and keep people well for a long time. Unfortunately in many African countries patients often are only able to come for treatment when they are very sick and HIV has already had a big effect on their bodies. For example they may often have malnutrition, diarrhoea, infections such as tuberculosis, serious lung infections (pneumonia) and other severe infections. This means that more people die sometime during the first three months after starting ARVs; this is quite different compared to patients in rich countries.

What is the REALITY study?

We want to find out whether or not giving extra treatments during the first three months of taking ARVs will help sick people with HIV and prevent some of them from dying early, even though they are on ARVs – or whether giving extra treatments makes no difference to survival but makes it harder to take ARVs. In the REALITY study we may give extra treatments just during the first three months of starting ARVs. They are:

1. An extra ARV drug added to 3 standard ARVs (so 4 ARVs rather than 3)

2. A single pill containing cotrimoxazole (septrin) and isoniazid (to fight TB), plus fluconazole (to fight thrush) and azithromycin (to fight bacterial infections) and albendazole (to kill worms).

These are all are medicines which fight against common infections in sick people with HIV.

3. Extra food supplements to provide high energy food

Why have I been asked if I would like to take part in the REALITY study?

We have asked you because you have HIV and also low immunity. Immunity protects your body against infections, but it is attacked by HIV. Immunity is measured by a blood test called the CD4 count. Your CD4 count has been measured and has been found to be very low (less than 100). Therefore the risk of you getting sick even after you start ARVs is higher than in people starting ARVs with higher CD4 counts, and you may benefit from receiving the extra medicines and food listed above.

What treatments will I be given?

Everyone in the study will receive 3 ARVs and cotrimoxazole, together with any other medicines they are already taking or need for treatment of any infections. In addition , you will have a chance of getting or not getting each of the three extra treatments (an extra ARV, medicines to fight other infections and nutritional supplements) for the first 12 weeks (3 months) as well. Whether or not you get the extra treatments will be chosen by chance by a computer.

1) For treatment fighting the HIV virus, you will get

- EITHER 3 standard ARVs
- OR 3 standard ARVs + 1 extra ARV for 12 weeks

AND

2) For treatment fighting infections, you will get

- EITHER cotrimoxazole (septrin) tablets for the first 12 weeks and then tablets containing Cotrimoxazole (septrin) + Isoniazid after 12 weeks (this is standard care for everyone in your country) *<remove isoniazid for sites where this is not standard of care>*
- OR tablets containing Cotrimoxazole (septrin) + Isoniazid from when you start ARVs plus an extra tablet of Fluconazole for 12 weeks and an extra tablet of Azithromycin for 5 days only and a single extra tablet of albendazole (anti-worms) when you start treatment (extra medicines which may help people with very low CD4 counts)

AND

3) For nutritional support, you will get

- EITHER whatever food supplements are available generally within your clinic
- OR extra high energy food supplements for 12 weeks

The study treatments last for 12 weeks (3 months). After the first three months you will continue to take 3 standard ARVs to fight HIV, cotrimoxazole (septrin) to help to protect against infections long-term, isoniazid to protect against TB (where this is local standard of care) *<remove where this is not standard of care>* regardless of which treatment groups you are in. That means that you receive things that might be different compared to what your clinic would regularly give only for the first 12 weeks on ARVs.

What will happen if I take part?

If you are interested in taking part in the REALITY study, we will ask you to sign a consent form for screening tests and checkup to see if you can go into the study. After this the following will happen:

(1) The study doctor will examine you thoroughly to make sure you are not sick with another infection needing treatment at this time. Blood tests will be done to check there is no reason why ARVs cannot be started and to measure your CD4 cell count. It will take between 1 day to 14 days to get the results from these blood tests. If you have a low CD4 count and the other test results are OK and you still want to, you can then join the REALITY study.

(2) We will then ask you to sign another consent form agreeing to join the REALITY study. We will weigh you and measure your height and arm circumference. The computer will decide which treatment groups you are in. We will give you ARVs and tablets to fight infections and the doctor and nurses will tell you all about the medicines and how to take them. If you need extra nutrition because of your weight or because the computer has put you in the group receiving extra nutrition, we will also provide this for you.

(3) After starting the REALITY study a doctor will see you at 4, 8, 12, and then every 12 weeks by a doctor. We will take blood from you (about 3 teaspoons) to check for any side effects of the medicines. Some of the blood will be used to work out how much of the ARVs that people in REALITY are taking is actually getting into their blood, where it can fight the HIV virus. Some of the blood collected from you will be stored for tests which will be done later, such as tests to find out exactly how well your immunity was fighting HIV, and you may not be given results from these tests. New tests are being developed all the time, so we are asking for you to agree that your blood samples can be used in the future for any tests that are relevant to HIV, either during the REALITY study or after it ends. These tests would be decided by a group of HIV experts and any samples would never be identified by your name.

(4) We will give you supplies of medicines and, if necessary, food supplements, and you will see a nurse every 4 weeks up until week 12, and then every 6 weeks up until week 24, and then every 12 weeks after that, to receive more medicines and supplements, and so that you can return old bottles and any unused medicines or supplements.

What are the possible risks of taking part?
ARVs, like all medicines, have unwanted side-effects, which are sometimes serious. Serious side effects happen in fewer than 1 in 20 people. If you experience symptoms tell your clinic doctor at your next visit, or if you are feeling unwell and are worried you should come to the clinic as soon as possible. It may be necessary to stop the ARVs after which the problem usually goes away. We will replace the ARVs that are causing you problems with other ARVs.

Studies in rich countries have shown that taking four ARVs gives a very strong response to HIV so we want to see if taking an extra fourth ARV for the first 12 weeks of treatment reduces the risk of getting very sick after people start ARVs. The risks are that you could have side-effects to this extra medicine, or that you could find taking four medicines more difficult than three. The most common side-effects from the extra drug used in REALITY, raltegravir, are tummy problems, rashes, and liver problems - these occur in less than 1 in 10 people. If people don’t take all their ARVs all the time, the HIV virus can develop resistance to the drugs which stops them working over the longer term. The potential benefits are that your immune system may respond better both initially and over the long term if a fourth ARV is used in the first 12 weeks.

Similarly whilst adding other extra medicines to fight infections could reduce the risk of dying from these infections, it might also increase the risk of other side-effects, some of which can be serious, and might also make it harder to take all the ARVs. This study is designed to find out whether the potential benefits outweigh the potential risks. The most important side-effects from the main extra

non-ARVs used in REALITY, fluconazole and isoniazid, are problems with your liver, tingling or numbness in your hands or feet, tummy problems or rashes - these occur in less than 1 in 10 people. Fluconazole can also cause headaches in some people.

It is important that you understand that you are in a research study and that you may therefore find out in years to come, when you look back, that you may or may not have received what proved to be the best treatment. But at the moment we do not know whether taking the extra medicines or nutrition is better for you when you start ARVs – this is the reason we are doing the trial. The ARVs being used in the REALITY study are all recommended by the World Health Organisation (WHO), but may not all be the same as the ARVs used in the national programmes.

What are the possible benefits of taking part?

You may be taking pills that combine different ARVs and/or that combine different medicines that fight infections, so you will have fewer tablets to take than if these were given separately.

You will be watched carefully for any side effects and responses to the treatments.

Entering this study may not directly benefit you, but the information we get from the REALITY study will help to guide on the best way to treat patients like you in the future who have HIV. We also hope that the treatment and support you have in this study will help you.

What if I become pregnant?

If you are a woman of child-bearing age, a pregnancy test will be carried out before you join the REALITY study. You will not be able to join REALITY if the test reveals that you are pregnant. We strongly encourage the use of contraceptives as some ARVs (but not all) may harm the unborn child if taken early in pregnancy. If you think you might have become pregnant during the study, please tell the doctor or nurse straight away. Your pregnancy will be confirmed with a test after which your treatment will be reviewed. You will be able to stay in the study if you become pregnant.

How long will the study continue?

After about one year (48 weeks), the REALITY study will end for you and your care will be provided by the national health system including providing you with ARVs in line with the national programme. When the last REALITY patient finishes the study, we would like to find out whether you are still attending the national programme and how you are doing.

How important is it to take the treatments regularly?

It is very important that you do not miss any doses of the medicines (ARVs or the other drugs) and that you do not share your medicines with anyone else

If you do not take your ARVs as the doctor tells you they may lose their effect, as the HIV virus can become resistant to them. You should ask your doctor’s advice before you take any other medicines. If you do not attend a clinic visit, we would like to send a home visitor to your home to find out how you are.

What medicines will be used?

The doctor will give you a card explaining which medicines you will take. We know that it can be difficult taking ARVs every day. We want to know how well you take your medicines and if there are any particular problems taking them. From time to time during the study we will ask you some questions about how you manage taking these medicines.

What happens to the information collected in the REALITY study?

REALITY clinic staff will collect information whenever they see you. This information will be stored in a computer and only an independent committee, called the Data Monitoring Committee, will look at it regularly during the study. Information from the REALITY study will be analysed when it finishes and the results will be presented and published to improve the future care of adults and children with HIV in Africa. Results will also be discussed in community meetings at each centre.

What about protecting my confidentiality?

Information about you will be kept confidential and will not be made available to anyone who is not connected with the REALITY study. Your medical notes and study information will be available to study staff and may also be seen by other independent people authorised to ensure that the REALITY study is being properly carried out. Strict confidentiality will be maintained at all times. Your names will never be used for study information or for stored blood samples; these will be identified only by a study number, date of birth and initials. There will be one list which links this study number to your name, and this list will be safely kept private in a locked cabinet.

What else do I need to know?

Arrangements have been made for compensation if you come to any harm because of the treatments being used in the study.

We will give you **TRANSPORT MONEY TO COME TO AND FROM THE CLINIC**.

Blood samples that are collected during the study will be used for immediate analysis, and some samples will be stored for future work that is connected to this study. The samples will be stored at <insert name of site> in REALITY freezers.

You may withdraw from the study at any time, but if you do it would help us if you are able to tell us the reason for this.

How can I join the REALITY study?

After you have read this information sheet we will ask you to give consent to be seen by the doctor and to give a blood sample. After 1 to 14 days, the blood test results will come back to the clinic and we will tell you whether you are eligible to start ARVs in REALITY. If you are, we will ask you to sign a form giving your consent to join the REALITY study. A home visitor may come to your home with you at the time of enrolment so that during the study they can help remind you about clinic appointments, and to make sure that you are alright if you are not able to attend the clinic as planned. If your blood tests show you are not able to join the REALITY study (for example if your CD4 count is higher than expected), your doctor will talk to you about whether you need to start ARVs in another clinic.

If you would like more information or have any questions about the REALITY study please ask the doctors, nurses or counsellors. If you still need more information, please call:

Insert names and telephone numbers as appropriate:
Name:

Telephone Number: …………………………………………….

If you have an injury or illness during your participation in REALITY, please contact:

Dr…………………………………….on……………………………..………

Nurse………………………………on…………………………

[Each country to use its own translated Informed Consent according to local regulatory requirements, on local headed paper for each site]

REALITY (Reduction of EArly mortaLITY in adults and children) trial

Simplified Information for Older Children

Introduction

In this leaflet, your 'carer' means the person who takes care of you. This could be your parent, a family member or another adult.

We are inviting you to take part in a research study called called REALITY, which is being carried out in Kenya, Malawi, Uganda and Zimbabwe. Please read this information carefully or ask someone to read it to you, and please discuss it with the people who look after you. You can ask us if there is anything you don't understand or are worried about and we will be happy to give you more information. We will give you a copy of this information to keep.

If you or your carer decide you should not take part in this study you can be sure that this will not affect the medical care you will get in future. The doctors and nurses will not mind if you don't take part. If you join the study but change your mind later on, you can stop taking part and it will not affect the care you get in the future either.

What is the reason for doing the REALITY study?

HIV is the virus that causes AIDS. At the moment there is no cure for HIV. However, there are anti-HIV medicines, called 'anti-retrovirals' or ARVs, that can control HIV and keep people well for a long time. Unfortunately many patients are only able to come for treatment when they are sick and HIV has already had a big effect on their bodies.

We want to find out whether or not giving extra treatments during the first three months of taking ARVs will help sick people with HIV, even though they are on ARVs – or whether giving extra treatments doesn't make any difference and makes it harder to take ARVs. The extra treatments that we may give you during the first three months of starting ARVs in the REALITY study are:

1. An extra ARV drug added to 3 standard ARVs (so 4 ARVs not 3)

2. A single pill containing cotrimoxazole (septrin) and isoniazid (to fight TB), plus fluconazole (to fight thrush) and azithromycin (to fight bacterial infections) and albendazole (to kill worms). So all are medicines which fight against common infections in sick people people with HIV.

3. Extra food supplements to provide high energy food

What treatment will I get in the study?

Everyone in the study will receive 3 ARVs and cotrimoxazole, together with any other medicines they are already taking or need for treatment of any infections. In addition you will have a chance of getting or not getting each of the three extra treatments (an extra ARV, medicines to fight other infections and nutritional supplements) for the first 12 weeks (3 months) as well. Whether or not you get the extra treatments will be chosen by chance by a computer.

1) For treatment fighting the HIV virus, you will get

- EITHER 3 standard ARVs
- OR 3 standard ARVs + 1 extra ARV for 12 weeks

AND

2) For treatment fighting infections, you will get
- EITHER Cotrimoxazole (septrin) tablets for the first 12 weeks and then tablets containing Cotrimoxazole (septrin) + Isoniazid after 12 weeks (this is standard care for everyone in your country) *<remove isoniazid where this is not standard of care>*)
- OR tablets containing Cotrimoxazole (septrin) + Isoniazid from when you start ARVs plus an extra tablet of Fluconazole for 12 weeks and an extra tablet of Azithromycin for 5 days only and a single extra tablet of albendazole (anti-worms) when you start treatment (extra medicines which may help people with low CD4 counts starting ARVs)

AND

3) For nutritional support, you will get
- EITHER whatever food supplements are available generally within your clinic
- OR extra high energy food supplements for 12 weeks

Whichever of these treatment groups you are in, after the first three months you will continue to take 3 standard ARVs to fight HIV, cotrimoxazole (septrin) to help to protect against infections long-term, isoniazid to protect against TB *<remove where this is not standard of care>*. That is, you only receive things that might be different compared to what your clinic would regularly give for the first 12 weeks on ARVs.

What will happen if I take part?

(1) The study doctor will examined you thoroughly and we will take blood from you to make sure it is OK for you to join the trial. It will take between 1 to 14 days to get the results from these blood tests. If you have a low CD4 count and the other test results are OK and you still want to, you can then join the REALITY study. A computer will decide which treatment groups you are in.

(2) After starting the study, a doctor will see you at 4, 8, 12, and then every 12 weeks by a doctor. We will take blood each time (about 3 teaspoons) to check how you are doing. Some of the blood collected will be stored for tests which will be done later, such as tests to find out exactly how well your immunity was fighting HIV, and you may not be given results from these tests. These tests would be decided by a group of HIV experts and any samples would never be identified by your name.

(3) You will be given supplies of medicines and, if necessary, food supplements, and you will see a nurse every 4 weeks to week 12, and then every 6 weeks to week 24, and then every 12 weeks to receive these and to return old bottles and any unused medicines or supplements.

(4) You may withdraw from the study at any time, but if you do it would help us if you are able to tell us the reason for this.

Entering this study may not directly benefit you, but the information we get from the REALITY study will help to guide on the best way to treat patients like you in the future who have HIV. We also hope that the treatment and support you have in this study will help you.

How long will the study continue?

After about one year (48 weeks), the REALITY study will end for you and your care will be provided by the national health system including providing you with ARVs in line with the national programme.

How important is it to take the treatments regularly?

It is very important that you do not miss any doses of the medicines (ARVs or the other drugs) and that you do not share your medicines with anyone else

If you do not take your ARVs as the doctor tells you they may lose their effect, as the HIV virus can become resistant to them.

What about side effects?

Anti-HIV medicines, like all medicines, can have side effects, which are sometimes serious. If you are unwell you should contact the clinic as soon as possible. If not serious then tell the clinic nurse at your next visit.

Confidentiality

Information about you will be kept confidential and will never be made available to anyone who is not connected with the REALITY study. Your name will never be used for study information or stored blood samples; these will be identified only by a study number, date of birth and initials.

How can I join the REALITY study?

If you want to, you will visit the doctor who will talk to you and take a blood sample. After 1 to 14 days, the blood test results will come back to the clinic and we will tell you whether you can join REALITY. If you still want to join, we will ask your carer to sign a form giving consent for you to participate. We will also ask you to sign to show that you agree with them to join the study.

If you would like more information or have any questions about the REALITY study please ask the doctors, nurses or counsellors. If you still need more information, please call:

Insert names and telephone numbers as appropriate:

Name:

Telephone Number: ……………………………………………….

If you have an injury or illness during your participation in REALITY, please contact:

Dr…………………………………on………………………………

Nurse……………………………on…………………………

INFORMATION ABOUT ABACAVIR FOR CARERS

In the ARROW trial which was conducted in Uganda and Zimbabwe, 4 out of 1206 children (ie about 1 in 300 children) developed a hypersensitivity reaction (serious allergic reaction). This reaction can sometimes be life threatening and in rare cases has been fatal, although this was not the case for any children in the ARROW trial. It has been seen slightly more often in adults (2- 5 in every 100 adults treated with abacavir).

Although a hypersensitivity reaction (serious allergic reaction) to abacavir usually appears within the first six weeks of abacavir treatment if it is going to happen at all, symptoms can occur at any time while being treated with abacavir. You will have been given a warning card to remind you about abacavir hypersensitivity and these symptoms. You should keep this card with your child at all times.

Most, but not all, patients have fever (high temperature) and/or rash as part of the reaction. Some of the other symptoms may include tiredness (or generally ill feeling) nausea, vomiting, diarrhoea, or abdominal pain, difficulty breathing, sore throat or cough. Other signs and symptoms may include muscle and joint aches and pains, swelling, numbness or tingling, headaches and anaphylaxis (a severe form of allergic reaction that often involves facial swelling and difficulty breathing). Occasionally inflammation of the eye (conjunctivitis), ulcers in the mouth or low blood pressure may occur. Symptoms generally get worse with continued treatment.

If your child develops symptoms that may be suggestive of abacavir hypersensitivity, or if your child stops taking their study medication for any reason, and particularly due to possible side effects described above, you should contact the clinic as soon as possible and follow the detailed guidance printed on the warning card from your child’s abacavir medication.

If your child becomes hypersensitive to abacavir, which will be ultimately decided in consultation with your child's study doctor, then you should return all of his/her unused abacavir (ZIAGEN) to the study site for proper disposal.

APPENDIX II - SCREENING AND TRIAL CONSENT AND ASSENT FORMS

SCREENING Consent Form
- Trial Participant -

(To be presented on local-headed paper)
Version 1.02 Date 06 Dec 2013

REALITY

Reduction of EArly mortaLITY in HIV-infected adults and children starting antiretroviral therapy

Please initial (or mark) box if you agree:

I have read/been read the information sheet date (version 1.02 dated 06 Dec 2013) for the REALITY study and agree to being assessed to see whether I am eligible to take part in this study.	
I understand this will involve being seen by the doctor who will ask questions about my health and my household, and having some blood taken for testing	
I agree that the results of these tests, and any blood left over from them, may be kept by the REALITY team for further studies on HIV. I understand that these results and samples will not be identified by my name.	

Participant's signature (or thumbprint)	Print name	Date (day/month/year)

Witness's signature (if thumbprint used above)	Print name	Date (day/month/year)

Consenting officer's signature	Print name	Date (day/month/year)

IMPORTANT: **one signed original to be kept in REALITY trial file by the researcher**
one signed copy to be given to the patient
one signed copy to be kept in the clinic file

SCREENING Consent Form
- Parents and Carers -

(To be presented on local-headed paper)
Version 1.02 Date 06 Dec 2013

REALITY

Reduction of EArly mortaLITY in HIV-infected adults and children starting antiretroviral therapy

Please initial (or mark) box if you agree:

I have read/been read the information sheet (version 1.02, dated 06 Dec 2013) for the REALITY study and agree to my child being assessed to see whether they are eligible to take part in this study.	
I understand this will involve my child being seen by the doctor who will ask questions about my child's health and our household, and my child having some blood taken for testing	
I agree that the results of these tests, and any blood left over from them, may be kept by the REALITY team for further studies on HIV. I understand that these results and samples will not be identified by either my or my child's name.	

Parent/carer's signature (or thumbprint)	Print name	Date (day/month/year)

Witness's signature (if thumbprint used above)	Print name	Date (day/month/year)

Consenting officer's signature	Print name	Date (day/month/year)

IMPORTANT: one signed original to be kept in REALITY trial file by the researcher
one signed copy to be given to the patient
one signed copy to be kept in the clinic file

TRIAL Consent Form
- Trial Participant -

(To be presented on local-headed paper)
Version 1.02 Date 06 Dec 2013

REALITY

Reduction of EArly mortaLITY in HIV-infected adults and children starting antiretroviral therapy

Please initial (or mark) box if you agree:

I confirm that I have read/ been read the patient information sheet (version 1.02 dated 06 Dec 2013) for the REALITY study and that I understand what will be required of me if I participate in the study. The study has been explained to me and my questions have been answered.	
I understand that my participation is voluntary and that I am free to withdraw at any time, without giving any reason, without my medical care or legal rights being affected.	
I understand that sections of any of my medical notes may be looked at by responsible individuals involved in the running of the study or from regulatory authorities where it is relevant to my taking part in research. I give permission for these individuals to have access to my records, but understand that strict confidentiality will be maintained.	
I understand that I will be given anti-retroviral drugs (ARVs) for 48 weeks while I am in the REALITY study. After the study, my healthcare will be provided by the national health system.	
I agree to allow blood samples to be taken and stored for later testing. I understand that I may not be given the results of tests performed on stored samples.	
I agree to allow the REALITY care team to try and obtain information about my wellbeing from my clinic notes at the final end of the study (when the last patient enrolled has been in the study for 48 weeks).	
I agree to take part in the REALITY study	

Participant's signature (or thumbprint)	Print name	Date (day/month/year)

Witness's signature (if thumbprint used above)	Print name	Date (day/month/year)

Consenting officer's signature	Print name	Date (day/month/year)

IMPORTANT: one signed original to be kept in REALITY trial file by the researcher
one signed copy to be given to the patient
one signed copy to be kept in the clinic file

TRIAL Consent Form
- Parents and Carers -

(To be presented on local-headed paper)
Version 1.02 Date 06 Dec 2013

REALITY

Reduction of EArly mortaLITY in HIV-infected adults and children starting antiretroviral therapy

Please initial (or mark) box if you agree:

I confirm that I have read/ been read the patient information sheet (version 1.02 dated 06 Dec 2013) for the REALITY study and that I understand what will be required if my child participates in the study. The study has been explained to me and my questions have been answered.	
I understand that my child's participation is voluntary and that I am free to withdraw him or her at any time, without giving any reason, without my medical care or legal rights or my child's medical care or legal rights being affected.	
I understand that sections of any of my child's medical notes may be looked at by responsible individuals involved in the running of the study or from regulatory authorities where it is relevant to my child's participation in this research. I give permission for these individuals to have access to my child's records, but understand that strict confidentiality will be maintained.	
I understand that my child will be given anti-retroviral drugs (ARVs) for 48 weeks while he or she participates in the REALITY study. After the study, my child's healthcare will be provided by the national health system.	
I agree to allow blood samples to be taken from my child and for my child's samples to be stored for later testing. I understand that my child and I may not be given the results of tests performed on stored samples.	
I agree to allow the REALITY care team to try and obtain information about my child's wellbeing from their clinic notes at the final end of the study (when the last patient enrolled has been in the study for 48 weeks).	
I agree for my child to participate in the REALITY study	

Parent/carer's signature (or thumbprint)	Print name	Date (day/month/year)

Witness's signature (if thumbprint used above)	Print name	Date (day/month/year)

Consenting officer's signature	Print name	Date (day/month/year)

IMPORTANT: one signed original to be kept in REALITY trial file by the researcher, one signed copy to be given to the patient, one signed copy to be kept in the clinic file

TRIAL Assent Form

(To be presented on local-headed paper)
Version 1.02 Date 06 Dec 2013

REALITY

Reduction of EArly mortaLITY in HIV-infected adults and children starting antiretroviral therapy

Please initial (or mark) box if you agree:

I confirm that I have read/ been read the patient information sheet (version 1.02 dated 06 Dec 2013) for the REALITY study and that I understand what will be required of me. The study has been explained to me and my questions have been answered.	
I understand that I can decide myself if I want to or don't want to be part of the study.	
I agree to take part in the REALITY study	

Child's signature (or thumbprint) where appropriate	Print name	Date (day/month/year)

Consenting officer's signature	Print name	Date (day/month/year)

IMPORTANT: one signed original to be kept in REALITY trial file by the researcher
one signed copy to be given to the patient
one signed copy to be kept in the clinic file

APPENDIX III - TEMPLATE PATIENT INFORMATION SHEETS AND CONSENT FOR COLLECTION OF STOOL SAMPLES (HARARE, KILIFI ONLY)

[Each country to use its own translated Informed Consent for relevant local languages according to local regulatory requirements, on local headed paper for each site]

REALITY Stool Collection - Information for Patients
(note: same patient information for carers/parents, with reference to "your child" rather than "you". Simplified shortened version to be used for older children)

Introduction
We are inviting you to join an extra part of the REALITY trial. This extra study is looking at what effect the treatment you are receiving has on the gut. Before you decide it is important for you to understand why the research is being done and what it will involve. Please take time to read the following information carefully or have someone read it to you, and discuss it with others if you wish. We will give you a copy to keep. Ask the clinic nurses or counsellors if there is anything that is not clear or if you would like more information. Providing or not providing stool samples in this extra part of REALITY will not change how you are treated in REALITY. Take time to decide whether or not you wish to take part. You may decide that you do not wish to take part now or you may wish to withdraw from the study later. This will not influence the care you receive now or in future.

What is the reason for collecting stool samples in REALITY?
You have already joined the REALITY trial, which is looking at whether extra treatments (as well as antiretrovirals, ARVs) given to people with HIV infection and a low CD4 count may reduce the risk of dying early. We want to understand how these extra treatments may be working. We know that HIV attacks the lining of the gut and damages it. Some people get diarrhoea or problems absorbing food. Other people do not have any tummy problems, but they may still have damage to the gut that they are not aware of. We call this damage inflammation. Some of the extra medicines that people in REALITY are taking may help to repair the gut, and reduce the amount of inflammation. This may reduce sickness and decrease the chance of dying after starting ARVs.

We all have bacteria (bugs) living harmlessly in the gut that keep us well. We call these bugs the 'gut flora' and they are a natural part of our bodies. When we become ill, though, the types of bugs that are found in the gut may change. We think this might happen in people with HIV infection, because of the damage that the virus causes to the gut. Some of the extra medicines being given in REALITY, such as antibiotics, might also change the bugs in the gut flora, because antibiotics are designed to kill bacteria.

So we want to ask if you will allow us to test a sample of your stool (poo) at certain points in the trial to measure gut inflammation and to look at the types of bugs present in the gut flora. This information might help us to understand how HIV makes people sick and how the extra medicines in REALITY are working.

What will happen if I take part?
Joining this extra part of the REALITY trial will not make any difference to how you are managed; there will be no changes to your medicines or clinic visits. All we want to do is to collect a stool sample from you on four different visits to the clinic. We will ask you to collect a small sample of stool at home (on the morning you are due to come to clinic) or while you are in the clinic. This

sample will be collected into a container using a spatula that the clinic nurse will give to you. This is the only extra thing we will ask you to do. There are no additional questionnaires to fill in, and no extra time involved.

What are the risks and benefits of taking part?
There will be no additional risks to collecting stool samples in REALITY. None of your treatment will change; there will be no extra blood tests or change in medicines. You are encouraged to wash your hands thoroughly with soap or ash under running water after collecting this stool sample. There are no direct benefits to you of taking part. The main goal of this research study is to gain knowledge that may help people with HIV in the future.

How long will the study continue?
We will ask you to collect a total of 4 stool samples during your year in the REALITY trial. We call these visits week 0, week 4, week 12 and week 48. The clinic nurses will remind you which visits these are.

What will happen to the samples that are collected?
We will ask you to collect the stool sample into a small container which gets frozen until we are ready to do the tests. We will do two types of test on the stool specimen. Firstly, we will measure proteins in the stool that tell us how much inflammation there is in your gut. Secondly, we will find out what types of bacteria are present in your gut flora. To do this, we extract the genetic material (DNA) from bacteria in the stool and we 'sequence' the DNA. DNA is like a code made up of different letters, and sequencing allows us to read the code and work out exactly what type of bugs are present in the gut. This is a very specialized technique and your stool sample will have to be sent to a laboratory abroad to do this testing. Once this has finished, any remaining stool samples will be destroyed and thrown away.

What happens to the information collected in this study?
Information from tests done on the stools collected in REALITY will be analysed and the results stored on a computer. We will not keep details of your name on the computer. Results will be presented and published to improve the future care of people with HIV in Africa.

Benefits and/or compensation
We cannot and do not guarantee or promise that you will receive any benefits from this study. Participants will receive reimbursement for the usual REALITY visits. There will be no additional reimbursement.

Confidentiality
Strict confidentiality will be maintained at all times. Names will not be used for study information and stored stool samples; only the REALITY trial number, date of birth and initials will identify these. There is just one list which links this trial number to your name (already held by the REALITY study), and this list is safely kept private in a locked cabinet.

How can I join?
After reading this information sheet you will be asked to sign the form below giving consent to participate the next time you see the doctor.

What do I do if I have questions or problems? For questions about this study contact:
Dr x on xxxxx or Dr x on xxxx

Before you sign this form, please ask any questions on any aspect of this study that is unclear to you. You may take as much time as necessary to think it over.

SIGNATURE

If you have read this information sheet (or been read to), all your questions have been answered and you are willing to participate in this study, we ask you to write your name below.

Name ______________________________ Signature _______________________

Date ______________________________

______________________________ ______________________________
Name of Witness (If appropriate) Signature of Witness and Date

______________________________ ______________________________
Name of Research Staff who discussed this document Signature of Research Staff

YOU WILL BE GIVEN A COPY OF THIS CONSENT FORM TO KEEP.

REALITY Stool Collection – Simplified Information for Adolescents and Older Children

Introduction
In this leaflet, your ‘carer’ means the person who takes care of you; this could be your parent, a family member or another adult. We are inviting you to join an extra part of the REALITY trial. This extra study is looking at what effect the medicines you are taking have on your tummy. Please read this information carefully or have someone read it to you, and discuss it with the people who look after you. You can ask us if there is anything you don’t understand or are worried about and we will be happy to give you more information. We will give you a copy of this information to keep. The doctors and nurses will not mind if you don’t take part. If you join this extra study but change your mind later on, you can stop taking part and it will not affect the care you get in REALITY or in the future either.

What is the reason for collecting stool samples in REALITY?
You have already joined the REALITY trial, which is looking at whether extra treatments (as well as antiretrovirals, ARVs) given to people with HIV infection make them less sick. We want to understand how these extra treatments may be working. We know that HIV attacks the lining of the tummy (which we also call the gut) and damages it. We call this damage inflammation. It is a bit like having a cut or a sore on your skin – the same thing can happen in the tummy, but you can’t always see or feel that it is there. Some of the extra medicines that people in REALITY are taking may help to make the gut better. This may help people feel better more quickly after starting ARVs.

We all have lots of harmless bugs in the tummy (called gut flora) and in our poo that don’t cause us any trouble. When we become ill, though, the types of bugs that are found in the gut may change. Some of the extra medicines being given in REALITY, like antibiotics, might also change the type of bugs in the gut flora, because antibiotics are designed to kill bugs. We would like to find out exactly what types of bugs are in the poo, because it might be one of the things that affects how quickly you start to feel better after starting ARVs.

So we want to ask if it would be OK to test a sample of your stool (poo) when you come to the clinic to measure gut inflammation and to look at the types of bugs in the poo. This information might help us to understand how HIV makes people sick and how the extra medicines in REALITY are working.

What will happen if I take part?
You will carry on in the REALITY trial exactly as you are right now; there will be no changes to your medicines or clinic visits. All we want to do is to collect a stool (poo) sample from you on four different visits to the clinic. This is like going to the toilet normally to do a poo, but we will collect a small sample of the poo into a pot so we can do some tests on it later. The nurse will give you a pot and explain to you and your carer exactly how to collect the sample. It does not hurt. Nothing else will change by being in this study.

What are the risks and benefits of taking part?
There will be no extra risks to collecting stool samples in REALITY. None of your treatment will change; there will be no extra blood tests or change in medicines. You should wash your hands thoroughly with soap or ash under running water after collecting a stool sample. There are no direct benefits to you of taking part but we hope that by doing this research we may help other people in the future.

How long will the study continue?

We will ask you to bring a poo sample on four different days during the next year when you are seeing us in the REALITY clinic. The nurses will tell you which clinic visits these are.

What will happen to the samples that are collected?
We will ask you to collect the stool sample in a small container which gets frozen until we are ready to do tests on the sample. We will be looking at the amount of inflammation in the gut by measuring a type of protein in the stool. We will find out what type of bacteria are in the stool by doing a test called 'sequencing'. Bugs have something called DNA inside them, which keeps them alive. DNA is like a code made up of different letters, and sequencing allows us to read the code and work out exactly what type of bugs are in the gut. This is a very specialized test and your stool sample will have to be sent to a laboratory abroad to do this testing. Once this has finished, any remaining stool samples will be destroyed and thrown away.

What happens to the information collected in this study?
All the results that we get from these tests will be kept on a computer. We will not put your name or address on the computer. We will write about the results of the study so that doctors and nurses and other people can learn about what we have found, but we won't tell anybody the names of the people who were in the study.

Confidentiality
Information about you will be kept private and will not be made available without your consent to anyone who is not connected with the REALITY study. Names will not be used for study information and stored stool samples; these will be identified only by your study number, date of birth and initials.

How can I join?
After reading this information sheet you and your carer will be asked to sign a form giving consent to participate the next time you see the doctor.

If you would like more information, or have any questions about the REALITY study please ask the doctors, nurses or counsellors.

What do I do if I have questions or problems?For questions about this study contact:
Dr x on xxxxx or Dr x on xxxx

Before you sign this form, please ask any questions on any part of this study that is not clear to you. You may take as much time as you need to think it over.

SIGNATURE

Participant
Name (print) ________________________________ Signature ________________________

Date ________________________________

Parent or legally authorized representative
Name (print) ________________________________ Signature ________________________

Date ________________________________

Relationship to the Participant ______________________________

______________________________ ______________________________

Name of Witness<br>(If appropriate)	Signature of Witness and Date

______________________________ ______________________________

Name of Research Staff who discussed this document	Signature of Research Staff

YOU WILL BE GIVEN A COPY OF THIS CONSENT FORM TO KEEP.

APPENDIX IV- DETAILS OF ANTIRETROVIRAL THERAPY

Detailed information on all drugs will be provided on regularly updated disk from the EMEA website and in individual drug brochures

Drugs will be prescribed for children according to weight bands used in WHO tables, rather than the recommended EMEA dosing, see Section 5.1.1, p38.

DRUG (RECOMMENDED DOSING)	CLASS AND MAJOR TOXICITIES	CONTRAINDICATIONS	DRUG INTERACTIONS
Raltegravir<br><br>adults: 400mg twice daily	Integrase inhibitor<br><br>Mostly gastrointestinal side effects: Nausea, vomiting, abdomianl pain. Also dizziness, insomnia, rash, elevated liver enzymes, pancreatitis, raised triglycerides	Caution if risk factors for myopathy or rhabdomyolysis; caution in chronic hepatitis B or C (increased risk of hepatic side effects)	Omeprazole increases the plasma concentration of raltegravir. Manufacturer advises avoid concomitant use. Other proton pump inhibitors and also histamine H2 antagonists may also increase the concentration of raltegravir. Manufacturer advises avoid concomitant use. Plasma concentration of raltegravir reduced by rifampicin – consider increasing the dose of raltegravir.<br><br>Efavirenz and atazanavir/r both reduce raltegravir concentrations and tenofovir increases raltegravir concentrations; however, no change in dosing is required with any of these drugs
Tenofovir (Viread)<br><br>245 mg once daily<br><br>245 mg tablet is equivalent to 300mg of tenofovir disoproxil (as fumarate)	NRTI<br><br>Consider interruption of TDF therapy if serum creatinine > 2.0 mg/dl (177umol/l) or serum phosphate < 1.0 mg/dl (0.32 umol/l)<br><br>Most common side effects: diarrhoea, nausea, vomiting, flatulence (1 % discontinuance rate for GI side effects); grade 1 and 2 serum phosphate decreases requiring phosphate supplementation is some patients	Tenofovir should not be administered to patients with severe renal impairment.<br><br>No data on patients with mild or moderate renal impairment: caution advised.	Nephrotoxic agents should be avoided but if they have to be administered concomitantly, weekly monitoring of renal toxicity should be considered. No interaction with lamivudine, indinavir or efavirenz<br><br>In co-administration:<br><br>AUC for ddI increased by 44%, with lopinavir/ritonavir: 30% increase in Cmax and AUC for tenofovir and about 15% decrease in the same parameters for lopinavir. However, in clinical practice dose adjustments are not made.

DRUG (RECOMMENDED DOSING)	CLASS AND MAJOR TOXICITIES	CONTRAINDICATIONS	DRUG INTERACTIONS
Lamivudine (Epivir)<br>adults: 150mg twice daily or<br>300 mg once daily<br>(300 mg daily)<br>children: total daily dose = 8mg/kg	NRTI<br>Can be administered with or without food<br>Nausea, vomiting , abdominal pain or cramps, headache, arthralgia	In moderate to severe renal impairment with creatinine clearance 30-50 ml/min change to 150mg once daily and if <30ml/min: dose reduction recommended to below 150mg daily using oral solution. Switch to another drug is often practised.	High doses of co-trimoxazole for treatment of PCP should be avoided<br>Co-administration with intravenous ganciclovir or foscarnet is not recommended
Emtricitabine (Emtriva)<br>200mg once daily	NRTI<br>Can be administered with or without food. May cause abnormal dreams, pruritis, hyperpigmentation		Manufacturer advises avoid concomitant use of lamivudine
Truvada<br>1 tablet daily (contains 245mg tenofovir and 200mg emtricitabine)	See under tenofovir and emtricitabine above	See under tenofovir and emtricitabine above	See under truvada and emtricitabine above

DRUG (RECOMMENDED DOSING)	CLASS AND MAJOR TOXICITIES	CONTRAINDICATIONS	DRUG INTERACTIONS
Abacavir<br>adults: 300mg twice daily (or 600mg once daily)<br>children: total daily dose = 16mg/kg given as one or two divided doses	NRTI<br>Hypersensitivity reactions characterised by fever, rash, nausea, vomiting, diarrhoea, abdominal pain. Symptoms usually appear in the first 6 weeks but may occur at any time Discontinue and do not re-challenge.		Abacavir possibly reduces the concentration of methadone; plasma concentration of abacavir possibly reduced by rifampicin, phenytoin and phenobarbitol
Zidovudine<br>adults: 300mg twice daily<br>children: total daily dose 360 – 480 mg/m2	NRTI<br>Haematologic toxicity including anaemia and granulocytopenia. Headache, nausea. Myopathy.		
d4T<br>adults: 30mg twice daily<br>children: total daily dose = 2 mg/kg	NRTI<br>lipoatrophy/lipodystrophy (less in younger children).<br>Peripheral neuropathy and pancreatitis.<br>Lactic acidosis and severe hepatomegaly with steatosis		

DRUG (RECOMMENDED DOSING)	CLASS AND MAJOR TOXICITIES	CONTRAINDICATIONS	DRUG INTERACTIONS
Nevirapine<br>adults: 200mg twice daily<br>children: 150mg/m2 twice daily	NNRTI<br>Skin rash in 10-20%. Can treat through mild/moderate without constitutional symptoms but some are severe requiring hospitalisation and life-threatening including Stevens-Johnson syndrome, toxic epidermal necrolysis), fever, nausea, headache and abnormal liver function tests.<br>Hepatitis.		
Efavirenz<br>adults: 600 mg once daily<br>children: total daily dose (mg)<br>13 - < 15 kg 200 mg<br>>15 - < 20kg 250 mg<br>>20 - < 25kg 300 mg<br>>25 - < 32.5kg 350 mg<br>>32.5 -< 40 kg 400 mg<br>> 40kg 600	Skin rash, CNS system (somnolence, insomnia, abnormal dreams, 'Spacey kids', confusion, abnormal thinking, impaired concentration, amnesia, agitation, depersonalisation, hallucinations, euphoria) mainly reported in adults: increased transaminase levels, teratogenic in primates (use in pregnancy should be avoided).		

APPENDIX V - TABLE OF CLINICAL SIGNS, SYMPTOMS, MONITORING AND MANAGEMENT OF SYMPTOMS OF SERIOUS ADVERSE EFFECTS OF ANTIRETROVIRAL DRUGS THAT REQUIRE DRUG DISCONTINUATION (ADAPTED FROM WHO GUIDELINES ANNEX 11B)

Adverse Effect	Possible Offending Drug(s)	Clinical Signs / Symptoms	Management
Acute hepatitis	Nevirapine (NVP); more uncommon with zidovudine (ZDV), didanosine (ddI), stavudine (d4T) (<1%); and protease inhibitors (PI), most frequently with ritonavir (RTV)	Jaundice, liver enlargement, gastrointestinal symptoms, fatigue, anorexia; NVP-associated hepatitis may have hypersensitivity component (drug rash, systemic symptoms, eosinophilia)	Monitor serum transaminases, bilirubin. All ARV should be stopped until symptoms resolve. NVP may need to be permanently discontinued.
Acute pancreatitis	ddI, d4T	Nausea, vomiting, and abdominal pain	If possible, monitor serum pancreatic amylase, lipase. All ART should be stopped until symptoms resolve. Restart ART with change to different NRTI, preferably one without pancreatic toxicity (e.g., ZDV)
Lactic acidosis	All nucleoside analogue reverse transcriptase inhibitors (NRTIs)	Initial symptoms are variable: a clinical prodromal syndrome may include generalized fatigue and weakness, gastrointestinal symptoms (nausea, vomiting, diarrhea, abdominal pain, hepatomegaly, anorexia, and/or sudden unexplained weight loss), respiratory symptoms (tachypnea and dyspnea) or neurologic symptoms (including motor weakness).	Discontinue all ARV; symptoms may continue or worsen after discontinuation of ART. Supportive therapy. Regimens that can be considered for restarting ART include a PI combined with an NNRTI and tenofovir

ADVERSE EFFECT	POSSIBLE OFFENDING DRUG(S)	CLINICAL SIGNS / SYMPTOMS	MANAGEMENT
Hyper-sensitivity reaction	Abacavir (ABC) Nevirapine (NVP)	ABC: Constellation of acute onset of symptoms including: fever, fatigue, myalgia, nausea/vomiting, diarrhea, abdominal pain, pharyngitis, cough, dyspnea (with or without rash). While these symptoms overlap those of common infectious illness, the combination of acute onset of both respiratory and gastrointestinal symptoms after starting ABC is more typical of a hypersensitivity reaction.<br>NVP: Systemic symptoms of fever, myalgia, arthralgia, hepatitis, eosinophilia with or without rash.	Discontinue all ARVs until symptoms resolve. The reaction progressively worsens with drug administration and can be fatal. Administer supportive therapy. Do not rechallenge with ABC (or NVP), as anaphylactic reactions and death have been reported. Once symptoms resolve, restart ARVs with change to different NRTI if ABC-associated or to PI- or NRTI-based regimen if NVP-associated.
Severe rash / Stevens-Johnson syndrome	Non nucleoside reverse transcriptase inhibitors (NNRTIs): nevirapine (NVP)	Rash usually occurs during the first 2-4 weeks of treatment. The rash is usually erythematous, maculopapular, confluent, most prominent on the body and arms, may be pruritic and can occur with or without fever. Life-threatening Stevens-Johnson Syndrome or toxic epidermal necrolysis (SJS/TEN) has been reported in ~0.3% of infected individuals receiving NVP	Discontinue all ARVs until symptoms resolve. Permanently discontinue NVP for rash with systemic symptoms such as fever, severe rash with mucosal lesions or urticaria, or SJS/TEN; once resolves, switch ART regimen to different ARV class (e.g., 2 NRTIs and tenofovir or 2 NRTIs and PI).
Severe peripheral neuropathy	ddI, d4T, (3TC - unusual)	Pain, tingling, numbness of hands or feet; distal sensory loss, mild muscle weakness, and areflexia can occur.	Stop suspect NRTI and switch to different NRTI that does not have neurotoxicity (e.g., ZDV, ABC). Symptoms usually resolve in 2-3 weeks.
Severe anaemia	ZDV	Severe pallor, tachycardia at rest<br>Shortness of breath on exertion (SOBOE)	Stop ZDV and switch to another drug

APPENDIX VI - HANDLING AND STORAGE OF SPECIMENS

Processing of EDTA blood for plasma and cell store

For best results, centrifuge within 2 hours of drawing blood. If the time to processing is not within the same working day, do not proceed.

Centrifuge at 1500g for 15 minutes to separate cells from plasma.

Using a sterile pipette, collect the plasma more than 5mm above the buffy cell coat layer (grey layer above red cell layer).

Place the plasma, using up to four aliquots, in the cryovials provided for the trial. A minimum of two aliquots is required. Routinely three cryovials of plasma and one cryovial containing the DNA pellet (if feasible) should be stored at each visit.

Label the cryovials with the patient “PLASMA” identification labels

Store cell pellet for later DNA isolation (if feasible). After plasma has been removed transfer the cell pellet into cryovial

Label the cryovials with the patient “CELLS” identification label and store at -80°C (if not possible then -20°C)

Storage of samples

Store within 4- 6 hours of drawing blood at -70°C (temperatures to -50°C allowed if this is not available). Place upright in the cryobox provided for the trial.

Add the Patient ID label to the Specimen Storage Log for sample. Complete the details for type of specimen processed, visit month, total number of tubes and storage temperature. Start a new log sheet when either the sheet is full or when starting to fill a new cryobox.

Immunophenotyping

The Immunophenotyping assay uses two different panels of antibodies, FACS panel I (CD31 AND Ki67) and FACS panel II (CD38 AND HLA-DR). Both panels require 100 µl of heparin or EDTA anti-coagulated whole blood drawn within the last 24 hours. This blood will be taken from existing lymphocyte subset or haematology blood draws. Bloods are mixed with specific buffers and incubated with the correct antibodies and T-cell subsets identified using flow cytometry as described in the REALITY MOP.

Collection of supernatants using QuantiFERON®-TB Gold In-Tube (IT) system

The QuantiFERON®-TB Gold In-Tube (IT) system consist of three vacutainer type tubes and 1ml of blood is required for each tube. These 3mls of blood are to be taken from the amount originally allocated for plasma storage.

The three tubes consist of a Nil Control (Grey cap), TB Antigen (Red cap) and Mitogen Control (Purple cap). Antigens have been dried onto the inner wall of the blood collection tubes so it is essential that the contents of the tubes be thoroughly mixed with the blood. The tubes must be transferred to a 37°C incubator as soon as possible and within 16 hours of collection and then incubated and supernatants collected and stored as instructed in the REALITY MOP.

APPENDIX VII - EQ-5D QUESTIONNAIRE

Describing your own health today

By placing a tick in one box in each group below, please indicate which statements best describe your own health state today.

Mobility

I have no problems in walking about ❑
I have some problems in walking about ❑
I am confined to bed ❑

Self-Care

I have no problems with self-care ❑
I have some problems washing or dressing myself ❑
I am unable to wash or dress myself ❑

Usual Activities (e.g. work, study, housework, family or leisure activities)

I have no problems with performing my usual activities ❑
I have some problems with performing my usual activities ❑
I am unable to perform my usual activities ❑

Pain/Discomfort

I have no pain or discomfort ❑
I have moderate pain or discomfort ❑
I have extreme pain or discomfort ❑

Anxiety/Depression

I am not anxious or depressed ❑
I am moderately anxious or depressed ❑
I am extremely anxious or depressed ❑

APPENDIX VIII - WITHDRAWAL FORM

Please initial (or mark) box if you agree:

I/my child no longer wish to (or cannot) take trial ARVs and do not wish to (or cannot) attend further visits. I/my child agree to being contacted in the future (home visits or telephone) and to my/my child's medical records being consulted in future to obtain clinical	

Set in place procedure to follow the adult, adolescent or child up through visits and medical records and report any trial outcomes on the appropriate form. Inform the adult, adolescent or child and carer that s/he may still return for follow-up visits only or for trial drugs and follow-up visits at a later date.

I/my child no longer wish to (or cannot) take trial ARVs, do not wish to (or cannot) attend further visits and do not want to be contacted in the future. I/my child agree to my/my child's medical records being consulted in future to obtain clinical information for REALITY.	

Set in place procedure to follow the adult, adolescent or child up through medical records alone and report any trial outcomes on the appropriate form. Inform the adult, adolescent or child and carer that s/he may still return for follow-up visits only or for trial drugs and follow-up visits at a later date.

I/my child no longer wish to (or cannot) take trial ARVs and do not wish to (or cannot) attend further visits. I/my child do not agree to being contacted in the future or to my/my child's medical records being consulted in future to obtain clinical information for REALITY.	

Discontinue all follow up through medical records. The adult, adolescent or child and carer must sign a new consent form if s/he decides to rejoin the study at a later date.

Patient or carer's signature (or thumbprint)	Print name	Date (day/month/year)

Child's signature (or thumbprint) where appropriate	Print name	Date (day/month/year)

Witness's signature (if thumbprint used above)	Print name	Date (day/month/year)

Consenting officer's signature	Print name	Date (day/month/year)

IMPORTANT: One signed original to be given to patient
One signed original to be kept on file by the researcher
One signed original to be kept in the clinic notes

APPENDIX IX - CLINICAL PROGRESSION AND WHO DEFINITIONS

DIAGNOSTIC CRITERIA FOR WHO STAGE 3 AND 4 CONDITIONS EVENTS IN INFANTS AND CHILDREN UNDER 12 YEARS

Clinical event	Clinical diagnosis	Definitive diagnosis
**Clinical Stage 1**		
Asymptomatic	No HIV related symptoms reported and no clinical signs on examination.	Not applicable.
Persistent generalized lymphadenopathy	Persistent swollen or enlarged lymph nodes >1 cm at two or more non-contiguous sites (excluding inguinal) without known cause.	Clinical diagnosis
**Clinical Stage 2**		
Unexplained persistent hepatosplenomegaly	Enlarged liver and spleen without obvious cause.	Clinical diagnosis
Papular pruritic eruptions	Papular pruritic vesicular lesions. Also common in uninfected children: scabies and insect bites should be excluded.	Clinical diagnosis
Extensive wart virus infection	Characteristic warty skin lesions; small fleshy grainy bumps, often rough, flat on sole of feet (plantar warts); facial, more than 5% of body area or disfiguring.	Clinical diagnosis
Extensive molluscum contagiosum infection	Characteristic skin lesions: small flesh-coloured, pearly or pink, dome-shaped or umbilicated growths, may be inflamed or red; facial, more than 5% of body area or disfiguring. Giant molloscum may indicate more advanced immunodeficiency	Clinical diagnosis
Fungal nail infections	Fungal paronychia (painful, red and swollen nail bed) or onycholysis (painless separation of the nail from the nail bed.) Proximal white subungual onchomycosis is uncommon without immunodeficiency	Clinical diagnosis
Recurrent oral ulcerations	Current event plus at least one previous episode in past six months. Aphthous ulceration, typically with a halo of inflammation & yellow-grey pseudomembrane.	Clinical diagnosis
Unexplained persistent parotid enlargement	Asymptomatic bilateral swelling that may spontaneously resolve and recur, in absence of other known cause, usually painless	Clinical diagnosis
Lineal gingival erythema	Erythematous band that follows the contour of the free gingival line; may be associated with spontaneous bleeding	Clinical diagnosis
Herpes zoster	Painful rash with fluid-filled blisters, dermatomal distribution, can be haemorrhagic on erythematous background, and can become large and confluent.	Clinical diagnosis

CLINICAL EVENT	CLINICAL DIAGNOSIS	DEFINITIVE DIAGNOSIS
	Does not cross the midline	
Recurrent upper respiratory tract infection	Current event with at least one episode in past 6 months. Symptom complex; fever with unilateral face pain and nasal discharge (sinusitis) or painful swollen eardrum (otitis media), sore throat with productive cough (bronchitis), sore throat (pharyngitis) and barking croup-like cough (LTB). Persistent or recurrent ear discharge.	Clinical diagnosis
**Clinical Stage 3**		
Unexplained moderate malnutrition (see * at end of table)	Weight loss: low weight-for-age, not explained by poor or inadequate feeding and or other infections, and not adequately responding in two weeks to standard management, with downward crossing of at least one complete weight percentile on the CDC 2000 weight-for-age charts	Confirmed by downward crossing of at least one complete weight percentile on the CDC 2000 weight-for-age charts, not responding to standard management and with no other cause identified during investigation.
Unexplained persistent diarrhoea	Unexplained persistent (14 days or more) diarrhoea (loose or watery stool, three or more times daily), not responding to standard treatment.	Confirmed by stools observed and documented as unformed. Culture and microscopy reveal no pathogens.
Unexplained persistent fever (>37.5oC intermittent or constant for longer than one month)	Reports of fever or night sweats for longer than one month, either intermittent or constant, with reported lack of response to antibiotics or antimalarial agents. No other obvious foci of disease reported or found on examination. Malaria must be excluded in malarious areas.	Confirmed by documented fever of >37.50C with negative blood culture, negative malaria slide and normal or unchanged chest X-ray, and no other obvious foci of disease.
Oral candidiasis (after first 6-8 weeks of life)	Persistent or recurring creamy white to yellow soft small plaques which can be scraped off (pseudomembranous), or red patches on tongue, palate or lining of mouth, usually painful or tender (erythematous form).	Confirmed by microscopy or culture.
Oral hairy leukoplakia	Fine small linear patches on lateral borders of tongue, generally bilaterally, which do not scrape off.	Clinical diagnosis
Lymph node tuberculosis	Non acute, painless "cold" enlargement of peripheral lymph nodes, localized to one region. May have draining sinuses. Response to standard anti- tuberculosis treatment in one month.	Confirmed by histology or fine needle aspirate for Ziehl-Nielsen stain or culture.
Pulmonary tuberculosis	Nonspecific symptoms, e.g. chronic cough, fever, night sweats, anorexia and weight loss. In the older child also productive cough and haemoptysis. History of contact with adults with smear-positive pulmonary tuberculosis. No response to standard	Confirmed by one or more sputum positive smear for acid-fast bacilli and/or radiographic abnormalities consistent with active

CLINICAL EVENT	CLINICAL DIAGNOSIS	DEFINITIVE DIAGNOSIS
	broad-spectrum antibiotic treatment.	tuberculosis and/or culture-positive for Mycobacterium.
Severe recurrent bacterial pneumonia	Cough with fast breathing, chest indrawing, nasal flaring, wheezing, and grunting. Crackles or consolidation on auscultation. Responds to course of antibiotics. Current episode plus one or more in previous 6 months.	Confirmed by isolation of bacteria from appropriate clinical specimens (induced sputum, BAL, lung aspirate).
Acute necrotizing ulcerative gingivitis or stomatitis, or acute necrotizing ulcerative periodontitis	Severe pain, ulcerated gingival papillae, loosening of teeth, spontaneous bleeding, bad odour, and rapid loss of bone and/or soft tissue.	Clinical diagnosis
Symptomatic lymphocytic interstitial pneumonitis	No presumptive clinical diagnosis.	Diagnosed by chest X-ray: bilateral reticulonodular interstitial pulmonary infiltrates present for more than two months with no response to antibiotic treatment and no other pathogen found. Oxygen saturation persistently <90%. May present with cor pulmonale and may increased exercise-induced fatigue. Characteristic histology.
Chronic HIV-associated lung disease (including bronchiectasis)	History of cough productive of copious amounts of purulent sputum (bronchiectasis only), with or without clubbing, halitosis, and crepitations and/or wheezes on auscultation	Confirmed by chest X-ray: may show honeycomb appearance (small cysts) and/or persistent areas of opacification and/or widespread lung destruction, with fibrosis and loss of volume.
Unexplained anaemia (<8g/dl), or neutropaenia (<0.5 x 109 per litre) and/or chronic thrombocytopaenia (<50 x 109 per litre)	No presumptive clinical diagnosis.	Diagnosed on laboratory testing, not explained by other non-HIV conditions, not responding to standard therapy with haematinics, antimalarial agents or anthelmintic agents as outlined in WHO Integrated Management of Childhood Illness (IMCI) guidelines.

CLINICAL EVENT	CLINICAL DIAGNOSIS	DEFINITIVE DIAGNOSIS
**Clinical Stage 4**		
1 Unexplained severe wasting, stunting or severe malnutrition not adequately responding to standard therapy	Persistent weight loss not explained by poor or inadequate feeding, other infections and not adequately responding in two weeks to standard therapy. Characterised by:<br>visible severe wasting of muscles, with or without oedema of both feet, and/or downward crossing of at least two complete weight percentiles on the CDC 2000 weight-for-age charts	Confirmed by downward crossing of at least two complete weight percentiles on the CDC 2000 weight-for-age charts with or without oedema
Pneumocystis pneumonia	Dry cough, progressive difficulty in breathing, cyanosis, tachypnoea and fever; chest indrawing or stridor. (Severe or very severe pneumonia as in WHO IMCI guidelines). Usually rapid onset especially in infants under six months of age. Response to high-dose co-trimoxazole with or without prednisolone. Chest X-ray shows typical bilateral perihilar diffuse infiltrates	Confirmed by: cytology or immunofluorescent microscopy of induced sputum or BAL or histology of lung tissue.
Recurrent severe bacterial infection, e.g. empyema, pyomyositis, bone or joint infection or meningitis but excluding pneumonia	Fever accompanied by specific symptoms or signs that localize infection. Responds to antibiotics. Current episode plus one or more in previous 6 months	Confirmed by culture of appropriate clinical specimen.
Chronic herpes simplex infection; (orolabial or cutaneous of more than one month's duration or visceral at any site)	Severe and progressive painful orolabial, genital, or anorectal lesions caused by HSV infection present for more than one month.	Confirmed by culture and/or histology
Oesophageal candidiasis (or candidiasis of trachea, bronchi or lungs).	Difficulty in swallowing or pain on swallowing (food and fluids). In young children, suspect particularly if oral Candida observed and food refusal occurs and/or difficulties or crying when feeding.	Confirmed by macroscopic appearance at endoscopy, microscopy of specimen from tissue or macroscopic appearance at bronchoscopy or histology.
Extrapulmonary or disseminated tuberculosis	Systemic illness usually with prolonged fever, night sweats, and weight loss. Clinical features of organs involved, e.g. sterile pyuria, pericarditis, ascites, pleural effusion, meningitis, arthritis or orchiti, pericardial or abdominal	Confirmed by positive microscopy showing acid-fast bacilli or culture of Mycobacterium TB from blood or other relevant specimen except sputum or BAL. Biopsy and

CLINICAL EVENT	CLINICAL DIAGNOSIS	DEFINITIVE DIAGNOSIS
		histology.
Kaposi sarcoma	Typical appearance in skin or oropharynx of persistent, initially flat, patches with a pink or blood-bruise colour, skin lesions that usually develop into nodules.	Not required but may be confirmed by:<br>- typical red-purple lesions seen on bronchoscopy or endoscopy;<br>- dense masses in lymph nodes, viscera or lungs by palpation or radiology; and<br>- histology.
CMV retinitis or CMV infection affecting another organ, with onset at age over 1 month.	Retinitis only.<br>CMV retinitis may be diagnosed by experienced clinicians: typical eye lesions on serial fundoscopic examination; discrete patches of retinal whitening with distinct borders, spreading centrifugally, often following blood vessels, associated with retinal vasculitis, haemorrhage and necrosis.	Definitive diagnosis required for other sites. Histology. Cerebrospinal fluid polymerase chain reaction
CNS toxoplasmosis with onset at age over 1 month.	Fever, headache, focal neurological signs, convulsions. Usually responds within 10 days to specific therapy.	Not required but confirmed by computed tomography (CT) scan (or other neuroimaging) showing single or multiple lesions with mass effect or enhancing with contrast.
Extrapulmonary cryptococcosis (including meningitis)	Meningitis: usually subacute, fever with increasing severe headache, meningism, confusion and behavioural changes that respond to cryptococcal therapy.	Confirmed by CSF microscopy (India ink or Gram stain), serum or CSF CRAG test or culture.
HIV encephalopathy	At least one of the following, progressing over at least two months in the absence of another illness:<br>- failure to attain, or loss of, developmental milestones, loss of intellectual ability; OR<br>- progressive impaired brain growth demonstrated by stagnation of head circumference; OR<br>- acquired symmetric motor deficit accompanied by two or more of the following: paresis, pathological reflexes, ataxia, gait disturbances.	Confirmed by neuroimaging (brain CT scan or MRI) demonstrating atrophy and basal ganglia calcification and excluding other causes.
Disseminated mycosis (coccidiomycosis, histoplasmosis, penicilliosis)	No presumptive clinical diagnosis.	Diagnosed by:<br>Histology: usually granuloma formation.<br>Isolation: antigen detection from affected tissue; culture or microscopy from clinical specimen or blood culture.
Disseminated mycobacteriosis, other than TB	No presumptive clinical diagnosis.	Nonspecific clinical symptoms including progressive weight loss,

CLINICAL EVENT	CLINICAL DIAGNOSIS	DEFINITIVE DIAGNOSIS
		fever, anaemia, night sweats, fatigue or diarrhoea; plus culture of atypical mycobacterial species from stool, blood, body fluid or other body tissue, excluding lung.
Chronic cryptosporidiosis	No presumptive clinical diagnosis.	Confirmed by cysts identified on modified Ziehl-Nielsen microscopic examination of unformed stool
Chronic Isospora	No presumptive clinical diagnosis.	Confirmed in children with chronic diarrhoea by microscopic examination.
Cerebral or B-cell non-Hodgkin lymphoma	No presumptive clinical diagnosis.	Diagnosed by CNS neuroimaging: at least one lesion with mass effect on brain scan; histology of relevant specimen
Progressive multi focal leukoencephalopa thy	No presumptive clinical diagnosis.	Diagnosed by progressive nervous system disorder (cognitive dysfunction, gait or speech disorder, visual loss, limb weakness and cranial nerve palsies) together with hypodense white matter lesions on neuroimaging or positive polyomavirus Jacob Creutzfeldt PCR on cerebrospinal fluid
Symptomatic HIV-associated nephropathy	No presumptive clinical diagnosis.	Renal biopsy
Symptomatic HIV-associated cardiomyopathy	No presumptive clinical diagnosis.	Cardiomegaly and evidence of poor left ventricular function confirmed by echocardiography

* Moderate (grade 3) and severe (grade 4) malnutrition in children recruited to the trial will be defined using a modified CDC definition[79, 80] by the downward crossing of one or more complete percentiles because all REALITY sites report using the 2000 Centers for Disease Control (CDC) growth charts to record weight.

DIAGNOSTIC CRITERIA FOR WHO STAGE 4 AND WHO STAGE 3 CONDITIONS IN ADULTS

The diagnostic criteria for WHO Stage 4 and WHO Stage 3 conditions are based on the 2006 WHO HIV/AIDS Programme case definitions of HIV for surveillance and revised clinical staging and immunological classification of HIV-related disease in adults and children.

CLINICAL EVENT	CLINICAL DIAGNOSIS	DEFINITIVE DIAGNOSIS
**Clinical stage 3**		
Unexplained severe weight loss (more than 10% of body weight).	Reported unexplained involuntary weight loss (>10% of body weight) and visible thinning of face, waist and extremities with obvious wasting or body mass index <18.5 kg/m2; in pregnancy, the weight loss may be masked.	Documented loss of more than 10% of body weight.
Unexplained chronic diarrhoea for longer than one month.	Chronic diarrhoea (loose or watery stools three or more times daily) reported for longer than one month.	Three or more stools observed and documented as unformed, and two or more stool tests reveal no pathogens.
Unexplained persistent fever (intermittent or constant and lasting for longer than one month).	Fever or night sweats for more than one month, either intermittent or constant with reported lack of response to antibiotics or antimalarial agents, without other obvious foci of disease reported or found on examination; malaria must be excluded in malarious areas.	Documented fever >37.5°C with negative blood culture, negative Ziehl-Nielsen stain, negative malaria slide, normal or unchanged chest X-ray and no other obvious focus of infection.
Persistant oral candidiasis.	Persistent or recurring creamy white curd-like plaques that can be scraped off (pseudomembranous) or red patches on tongue, palate or lining of mouth, usually painful or tender (erythematous form).	Clinical diagnosis.
Oral hairy leukoplakia.	Fine white small linear or corrugated lesions on lateral borders of the tongue that do not scrape off.	Clinical diagnosis.
Pulmonary tuberculosis (current).	Chronic symptoms: (lasting at least 2–3 weeks) cough, haemoptysis, shortness of breath, chest pain, weight loss, fever, night sweats; PLUS EITHER<br>positive sputum smear;<br>OR<br>negative sputum smear;<br>AND<br>compatible chest radiograph (including but not restricted to upper lobe infiltrates, caritation, pulmonary fibrosist shrinkage. No evidence of	Isolation of M. Tuberculosis on sputum culture or histology of lung biopsy (with compatible symptoms).

CLINICAL EVENT	CLINICAL DIAGNOSIS	DEFINITIVE DIAGNOSIS
	extrapulmonary diseas.	
Acute necrotizing ulcerative gingivitis or necrotizing ulcerative periodontitis.	Severe pain, ulcerated gingival papillae, loosening of teeth, spontaneous bleeding, bad odour and rapid loss of bone and/or soft tissue.	Clinical diagnosis.
Severe bacterial infection<br>(such as pneumonia, meningitis, empyema, pyomyositis, bone or joint infection, bacteraemia and severe pelvic inflammatory disease).	Fever accompanied by specific symptoms or signs that localize infection and response to appropriate antibiotic.	Isolation of bacteria from appropriate clinical specimens (usually sterile sites).
Unexplained anaemia (<8 g/dl), neutropaenia (<0.5 × 109 per litre) or chronic (more than one month) thrombocytopaenia (<50 × 109 per litre).	Not presumptive clinical diagnosis.	Diagnosed on laboratory testing and not explained by other non-HIV conditions;<br>not responding to standard therapy with haematinics, antimalarial agents or anthelmintic agents as outlined in relevant national treatment guidelines, WHO Integrated Management of Childhood Illness guidelines or other relevant guidelines.
**Clinical stage 4**		
HIV wasting syndrome	Unexplained involuntary weight loss (>10% baseline body weight), with obvious wasting or body mass index <18.5;<br>PLUS EITHER<br>unexplained chronic diarrhoea (loose or watery stools three or more times daily) reported for longer than one month;<br>OR<br>reports of fever or night sweats for more than one month without other cause and lack of response to antibiotics or antimalarial agents; malaria must be excluded in malarious areas.	Documented weight loss (>10% of body weight);<br>PLUS EITHER<br>two or more unformed stools negative for pathogens;<br>OR<br>documented temperature of >37.5°C with no other cause of disease, negative blood culture, negative malaria slide and normal or unchanged chest X-ray.
Pneumocystis pneumonia	Dyspnoea on exertion or nonproductive cough of recent onset (within the past three months), tachypnoea and fever;	Cytology or immunofluorescent microscopy of induced sputum or bronchoalveolar lavage or histology of lung tissue.

CLINICAL EVENT	CLINICAL DIAGNOSIS	DEFINITIVE DIAGNOSIS
	AND<br>Chest X-ray evidence of diffuse bilateral interstitial infiltrates;<br>AND<br>No evidence of bacterial pneumonia; bilateral crepitations on auscultation with or without reduced air entry.	
Recurrent bacterial pneumonia<br>(this episode plus one or more episodes in last six months)	Current episode plus one or more previous episodes in the past six months; acute onset (<2 weeks) of severe symptoms (such as fever, cough, dyspnoea, and chest pain) PLUS new consolidation on clinical examination or chest X-ray; response to antibiotics.	Positive culture or antigen test of a compatible organism.
Chronic herpes simplex virus infection<br><br>(orolabial, genital or anorectal) of more than one month or visceral infection of any duration.	Painful, progressive anogenital or orolabial ulceration; lesions caused by recurrence of herpes simplex virus infection and reported for more than one month. History of previous episodes. Visceral herpes simplex virus requires definitive diagnosis.	Positive culture or DNA (by polymerase chain reaction) of herpes simplex virus or compatible cytology or histology.
Oesophageal candidiasis	Recent onset of retrosternal pain or difficulty on swallowing (food and fluids) together with oral candidasis.	Macroscopic appearance at endoscopy or bronchoscopy, or by microscopy or histology.
Extrapulmonary tuberculosis	Systemic illness (such as fever, night sweats, weakness and weight loss). Other evidence for extrapulmonary or disseminated tuberculosis varies by site: Pleural, pericardia, peritoneal involvement, meningitis, mediastinal or abdominal lymphadenopathy or ostetis. Discrete peripheral lymph node Mycobacterium tuberculosis infection (especially cervical) is considered a less severe form of extrapulmonary tuberculosis.	M. tuberculosis isolation or compatible histology from appropriate site or radiological evidence of miliary tuberculosis; (diffuse uniformly distributed small miliary shadows or micronodules on chest X-ray).
Kaposi sarcoma	Typical gross appearance in skin or oropharynx of persistent, initially flat, patches with a pink or violaceous colour, skin lesions that usually develop into plaques or nodules.	Macroscopic appearance at endoscopy or bronchoscopy, or by histology.
Cytomegalovirus disease (other than liver, spleen or lymph node)	Retinitis only: may be diagnosed by experienced clinicians. Typical eye lesions on fundoscopic examination: discrete patches of retinal hitening with distinct borders, spreading	Compatible histology or cytomegalovirus demonstrated in cerebrospinal fluid by culture or DNA (by polymerase chain reaction).

CLINICAL EVENT	CLINICAL DIAGNOSIS	DEFINITIVE DIAGNOSIS
	centrifugally, often following blood vessels, associated with retinal vasculitis, haemorrhage and necrosis.	
Central nervous system toxoplasmosis	Recent onset of a focal nervous system abnormality consistent with intracranial disease or reduced level of consciousness<br>AND<br>response within 10 days to specific therapy.	Positive serum toxoplasma antibody AND (if available) single or multiple intracranial mass lesion on neuroimaging (computed tomography or magnetic resonance imaging).
HIV encephalopathy	Disabling cognitive and/or motor dysfunction interfering with activities of daily living, progressing over weeks or months in the absence of a concurrent illness or condition other than HIV infection that might explain the findings.	Diagnosis of exclusion: and (if available) neuroimaging (computed tomography or magnetic resonance imaging).
Extrapulmonary cryptococcosis (including meningitis)	Meningitis: usually subacute, fever with increasing severe headache, meningism, confusion, behavioural changes that respond to cryptococcal therapy.	Isolation of Cryptococcus neoformans from extrapulmonary site or positive cryptococcal antigen test on cerebrospinal fluid or blood.
Disseminated nontuberculous mycobacteria infection	No presumptive clinical diagnosis.	Diagnosed by finding atypical mycobacterial species from stool, blood, body fluid or other body tissue, excluding the lungs.
Progressive multifocal leukoencephalopathy	No presumptive clinical diagnosis.	Progressive nervous system disorder (cognitive dysfunction, gait/speech disorder, visual loss, limb weakness and cranial nerve palsies) together with hypodense white matter lesions on neuro-imaging or positive polyomavirus JC polymerase chain reaction on cerebrospinal fluid.
Chronic cryptosporidiosis (with diarrhoea lasting more than one month)	No presumptive clinical diagnosis.	Cysts identified on modified Ziehl-Nielsen stain microscopic examination of unformed stool.
Chronic isosporiasis	No presumptive clinical diagnosis.	Identification of Isospora.
Disseminated mycosis (coccidiomycosis or histoplasmosis)	No presumptive clinical diagnosis.	Histology, antigen detection or culture from clinical specimen or blood culture.
Recurrent non-typhoid Salmonella bacteraemia	No presumptive clinical diagnosis.	Blood culture.
Lymphoma (cerebral or	No presumptive clinical diagnosis.	Histology of relevant specimen

CLINICAL EVENT	CLINICAL DIAGNOSIS	DEFINITIVE DIAGNOSIS
B-cell non-Hodgkin)		or, for central nervous system tumours, neuroimaging techniques.
Invasive cervical carcinoma	No presumptive clinical diagnosis.	Histology or cytology.
Atypical disseminated leishmaniasis	No presumptive clinical diagnosis.	Diagnosed by histology (amastigotes visualized) or culture from any appropriate clinical specimen.
Symptomatic HIV-associated nephropathy	No presumptive clinical diagnosis.	Renal biopsy.
Symptomatic HIV-associated cardiomyopathy	No presumptive clinical diagnosis.	Cardiomegaly and evidence of poor left ventricular function confirmed by echocardiography.

APPENDIX X - TOXICITY GRADINGS AND MANAGEMENT

Division of AIDS table for grading the severity of adverse events (published December 2004, clarification August 2009) apart from neutrophil gradings, which are based on WHO guidelines[51] and NIH Paediatric toxicity tables[81]. recognising the lower normal levels in African populations
ULN = Upper Limit of Normal ; LLN = Lower Limit of Normal

General Instructions:
If the need arises to grade a clinical adverse event (AE) that is not identified in the DAIDS AE grading table, us the category "Estimating Severity Grade" located at the top of the table.

If the severity of an AE could fall under either one of two grades (e.g. the severity of an AE could be either Grade 2 or Grade 3) select the higher of the two grades for the AE.

Definitions:
Basic Self-care Functions Adult: Activities such as bathing, dressing, toileting, transfer/movement, continence, and feeding.

Usual Social & Functional Activities Adult: Adaptive tasks and desirable activities, such as going to work, shopping, cooking, use of transportation, pursuing a hobby etc.

**CLINICAL**				
**PARAMETER**	**GRADE 1 MILD**	**GRADE 2 MODERATE**	**GRADE 3 SEVERE**	**GRADE 4 POTENTIALLY LIFE-THREATENING**
**ESTIMATING SEVERITY GRADE**				
Clinical adverse event NOT identified elsewhere in this DAIDS AE grading table	Symptoms causing no or minimal interference with usual social & functional activities	Symptoms causing greater than minimal interference with usual social & functional activities	Symptoms causing inability to perform usual social & functional activities	Symptoms causing inability to perform basic self-care functions OR Medical or operative intervention indicated to prevent permanent impairment, persistent disability, or death
**SYSTEMIC**				
Acute systemic allergic reaction	Localized urticaria (wheals) with no medical intervention indicated	Localized urticaria with medical intervention indicated OR Mild angioedema with no medical intervention indicated	Generalized urticaria OR Angioedema with medical intervention indicated OR Symptomatic mild bronchospasm	Acute anaphylaxis OR Life-threatening bronchospasm OR laryngeal edema

**CLINICAL**				
**PARAMETER**	**GRADE 1 MILD**	**GRADE 2 MODERATE**	**GRADE 3 SEVERE**	**GRADE 4 POTENTIALLY LIFE-THREATENING**
Chills	Symptoms causing no or minimal interference with usual social & functional activities	Symptoms causing greater than minimal interference with usual social & functional activities	Symptoms causing inability to perform usual social & functional activities	NA
Fatigue<br>Malaise	Symptoms causing no or minimal interference with usual social & functional activities	Symptoms causing greater than minimal interference with usual social & functional activities	Symptoms causing inability to perform usual social & functional activities	Incapacitating fatigue/ malaise symptoms causing inability to perform basic self-care functions
Fever (nonaxillary)	37.7 – 38.6°C	38.7 – 39.3°C	39.4 – 40.5°C	> 40.5°C
Pain (indicate body site)<br>DO NOT use for pain due to injection (See Injection Site Reactions: Injection site pain)<br>See also Headache, Arthralgia, and Myalgia	Pain causing no or minimal interference with usual social & functional activities	Pain causing greater than minimal interference with usual social & functional activities	Pain causing inability to perform usual social & functional activities	Disabling pain causing inability to perform basic self-care functions OR Hospitalization (other than emergency room visit) indicated
Unintentional weight loss	NA	5 – 9% loss in body weight from baseline	10 – 19% loss in body weight from baseline	≥ 20% loss in body weight from baseline OR Aggressive intervention indicated [e.g., tube feeding or total parenteral nutrition (TPN)]
**INFECTION**				
Infection (any other than HIV infection)	Localized, no systemic antimicrobial treatment indicated AND Symptoms causing no or minimal interference with usual social & functional activities	Systemic antimicrobial treatment indicated OR Symptoms causing greater than minimal interference with usual social & functional activities	Systemic antimicrobial treatment indicated AND Symptoms causing inability to perform usual social & functional activities OR Operative intervention (other than simple incision and drainage) indicated	Life-threatening consequences (e.g., septic shock)
INJECTION SITE REACTIONS				

CLINICAL					
PARAMETER		GRADE 1<br>MILD	GRADE 2<br>MODERATE	GRADE 3<br>SEVERE	GRADE 4<br>POTENTIALLY<br>LIFE-THREATENING
Injection site pain (pain without touching)<br>Or<br>Tenderness (pain when area is touched)		Pain/tenderness causing no or minimal limitation of use of limb	Pain/tenderness limiting use of limb OR Pain/tenderness causing greater than minimal interference with usual social & functional activities	Pain/tenderness causing inability to perform usual social & functional activities	Pain/tenderness causing inability to perform basic self-care function OR Hospitalization (other than emergency room visit) indicated for management of pain/tenderness
Injection site reaction (localized)					
	Adult > 15 years	Erythema OR Induration<br>of 5x5 cm – 9x9 cm (or 25 cm2 – 81cm2)	Erythema OR Induration OR Edema > 9 cm any diameter (or > 81 cm2)	Ulceration OR Secondary infection OR Phlebitis OR Sterile abscess OR Drainage	Necrosis (involving dermis and deeper tissue)
	Paediatric ≤ 15 years	Erythema OR Induration OR Edema present but ≤ 2.5 cm diameter	Erythema OR Induration OR Edema > 2.5 cm diameter but < 50% surface area of the extremity segment (e.g., upper arm/thigh)	Erythema OR Induration OR Edema involving<br>≥ 50% surface area of the extremity segment (e.g., upper arm/thigh) OR Ulceration OR Secondary infection OR Phlebitis OR Sterile abscess OR Drainage	Necrosis (involving dermis and deeper tissue)
Pruritis associated with injection<br>See also Skin: Pruritis (itching - no skin lesions)		Itching localized to injection site AND Relieved spontaneously or with < 48 hours treatment	Itching beyond the injection site but not generalized OR Itching localized to injection site requiring ≥ 48 hours treatment	Generalized itching causing inability to perform usual social & functional activities	NA
SKIN – DERMATOLOGICAL					
Alopecia		Thinning detectable by study participant (or by caregiver for young children and disabled adults)	Thinning or patchy hair loss detectable by health care provider	Complete hair loss	NA

CLINICAL				
**PARAMETER**	**GRADE 1<br>MILD**	**GRADE 2<br>MODERATE**	**GRADE 3<br>SEVERE**	**GRADE 4<br>POTENTIALLY LIFE-THREATENING**
Cutaneous reaction – rash	Localized macular rash	Diffuse macular, maculopapular, or morbilliform rash OR Target lesions	Diffuse macular, maculopapular, or morbilliform rash with vesicles or limited number of bullae OR Superficial ulcerations of mucous membrane limited to one site	Extensive or generalized bullous lesions OR Stevens-Johnson syndrome OR Ulceration of mucous membrane involving two or more distinct mucosal sites OR Toxic epidermal necrolysis (TEN)
Hyperpigmentation	Slight or localized	Marked or generalized	NA	NA
Hypopigmentation	Slight or localized	Marked or generalized	NA	NA
Pruritis (itching – no skin lesions)<br>(See also Injection Site Reactions: Pruritis associated with injection)	Itching causing no or minimal interference with usual social & functional activities	Itching causing greater than minimal interference with usual social & functional activities	Itching causing inability to perform usual social & functional activities	NA
**CARDIOVASCULAR**				
Cardiac arrhythmia (general)<br>(By ECG or physical exam)	Asymptomatic AND No intervention indicated	Asymptomatic AND Non-urgent medical intervention indicated	Symptomatic, non-life-threatening AND Non-urgent medical intervention indicated	Life-threatening arrhythmia OR Urgent intervention indicated
Cardiac-ischemia/infarction	NA	NA	Symptomatic ischemia (stable angina) OR Testing consistent with ischemia	Unstable angina OR Acute myocardial infarction
Hemorrhage (significant acute blood loss)	NA	Symptomatic AND No transfusion indicated	Symptomatic AND Transfusion of ≤ 2 units packed RBCs (for children ≤ 10 cc/kg) indicated	Life-threatening hypotension OR Transfusion of > 2 units packed RBCs (for children > 10 cc/kg) indicated
Hypertension				
Adult > 17 years (with repeat testing at same visit)	> 140 – 159 mmHg systolic<br>OR<br>> 90 – 99 mmHg diastolic	160 – 179 mmHg systolic<br>OR<br>100 – 109 mmHg diastolic	> 180 mmHg systolic<br>OR<br>> 110 mmHg diastolic	Life-threatening consequences (e.g., malignant hypertension) OR Hospitalization indicated (other than emergency room visit)

CLINICAL PARAMETER	GRADE 1 MILD	GRADE 2 MODERATE	GRADE 3 SEVERE	GRADE 4 POTENTIALLY LIFE-THREATENING
Paediatric ≤ 17 years (with repeat testing at same visit)	NA	91st – 94th percentile adjusted for age, height, and gender (systolic and/or diastolic)	≥ 95th percentile adjusted for age, height, and gender (systolic and/or diastolic)	Life-threatening consequences (e.g., malignant hypertension) OR Hospitalization indicated (other than emergency room visit)
Hypotension	NA	Symptomatic, corrected with oral fluid replacement	Symptomatic, IV fluids indicated	Shock requiring use of vasopressors or mechanical assistance to maintain blood pressure
Pericardial effusion	Asymptomatic, small effusion requiring no intervention	Asymptomatic, moderate or larger effusion requiring no intervention	Effusion with non-life threatening physiologic consequences OR Effusion with non-urgent intervention indicated	Life-threatening consequences (e.g., tamponade) OR Urgent intervention indicated
Prolonged PR interval				
Adult > 16 years	PR interval 0.21 – 0.25 sec	PR interval > 0.25 sec	Type II 2nd degree AV block OR Ventricular pause > 3.0 sec	Complete AV block
Paediatric ≤ 16 years	1st degree AV block (PR > normal for age and rate)	Type I 2nd degree AV block	Type II 2nd degree AV block	Complete AV block
Prolonged QTc				
Adult > 16 years	Asymptomatic, QTc interval 0.45 – 0.47 sec OR Increase interval < 0.03 sec above baseline	Asymptomatic, QTc interval 0.48 – 0.49 sec OR Increase in interval 0.03 – 0.05 sec above baseline	Asymptomatic, QTc interval ≥ 0.50 sec OR Increase in interval ≥ 0.06 sec above baseline	Life-threatening consequences, e.g. Torsade de pointes or other associated serious ventricular dysrhythmia
Paediatric ≤ 16 years	Asymptomatic, QTc interval 0.450 – 0.464 sec	Asymptomatic, QTc interval 0.465 – 0.479 sec	Asymptomatic, QTc interval ≥ 0.480 sec	Life-threatening consequences, e.g. Torsade de pointes or other associated serious ventricular dysrhythmia

CLINICAL				
**PARAMETER**	**GRADE 1 MILD**	**GRADE 2 MODERATE**	**GRADE 3 SEVERE**	**GRADE 4 POTENTIALLY LIFE-THREATENING**
Thrombosis/embolism	NA	Deep vein thrombosis AND No intervention indicated (e.g., anticoagulation, lysis filter, invasive procedure)	Deep vein thrombosis AND Intervention indicated (e.g., anticoagulation, lysis filter, invasive procedure)	Embolic event (e.g., pulmonary embolism, life-threatening thrombus)
Vasovagal episode (associated with a procedure of any kind)	Present without loss of consciousness	Present with transient loss of consciousness	NA	NA
Ventricular dysfunction (congestive heart failure)	NA	Asymptomatic diagnostic finding AND intervention indicated	New onset with symptoms OR Worsening symptomatic congestive heart failure	Life-threatening congestive heart failure
**GASTROINTESTINAL**				
Anorexia	Loss of appetite without decreased oral intake	Loss of appetite associated with decreased oral intake without significant weight loss	Loss of appetite associated with significant weight loss	Life-threatening consequences OR Aggressive intervention indicated [e.g., tube feeding or total parenteral nutrition (TPN)]
Comment: Please note that, while the grading scale provided for Unintentional Weight Loss may be used as a guideline when grading anorexia, this is not a requirement and should not be used as a substitute for clinical judgment.				
Ascites	Asymptomatic	Symptomatic AND Intervention indicated (e.g., diuretics or therapeutic paracentesis)	Symptomatic despite intervention	Life-threatening consequences
Cholecystitis	NA	Symptomatic AND Medical intervention indicated	Radiologic, endoscopic, or operative intervention indicated	Life-threatening consequences (e.g., sepsis or perforation)
Constipation	NA	Persistent constipation requiring regular use of dietary modifications, laxatives, or enemas	Obstipation with manual evacuation indicated	Life-threatening consequences (e.g., obstruction)
Diarrhea				

CLINICAL PARAMETER	GRADE 1 MILD	GRADE 2 MODERATE	GRADE 3 SEVERE	GRADE 4 POTENTIALLY LIFE-THREATENING
Adult and Paediatric ≥ 1 year	Transient or intermittent episodes of unformed stools OR Increase of ≤ 3 stools over baseline per 24-hour period	Persistent episodes of unformed to watery stools OR Increase of 4 – 6 stools over baseline per 24-hour period	Bloody diarrhea OR Increase of ≥ 7 stools per 24-hour period OR IV fluid replacement indicated	Life-threatening consequences (e.g., hypotensive shock)
Paediatric < 1 year	Liquid stools (more unformed than usual) but usual number of stools	Liquid stools with increased number of stools OR Mild dehydration	Liquid stools with moderate dehydration	Liquid stools resulting in severe dehydration with aggressive rehydration indicated OR Hypotensive shock
Dysphagia-Odynophagia	Symptomatic but able to eat usual diet	Symptoms causing altered dietary intake without medical intervention indicated	Symptoms causing severely altered dietary intake with medical intervention indicated	Life-threatening reduction in oral intake
Mucositis/stomatitis (clinical exam) Indicate site (e.g., larynx, oral) See Genitourinary for Vulvovaginitis See also Dysphagia-Odynophagia and Proctitis	Erythema of the mucosa	Patchy pseudomembranes or ulcerations	Confluent pseudomembranes or ulcerations OR Mucosal bleeding with minor trauma	Tissue necrosis OR Diffuse spontaneous mucosal bleeding OR Life-threatening consequences (e.g., aspiration, choking)
Nausea	Transient (< 24 hours) or intermittent nausea with no or minimal interference with oral intake	Persistent nausea resulting in decreased oral intake for 24 – 48 hours	Persistent nausea resulting in minimal oral intake for > 48 hours OR Aggressive rehydration indicated (e.g., IV fluids)	Life-threatening consequences (e.g., hypotensive shock)
Pancreatitis	NA	Symptomatic AND Hospitalization not indicated (other than emergency room visit)	Symptomatic AND Hospitalization indicated (other than emergency room visit)	Life-threatening consequences (e.g., circulatory failure, hemorrhage, sepsis)
Proctitis (functional-symptomatic) Also see Mucositis/stomatitis for clinical exam	Rectal discomfort AND No intervention indicated	Symptoms causing greater than minimal interference with usual social & functional activities OR Medical intervention indicated	Symptoms causing inability to perform usual social & functional activities OR Operative intervention indicated	Life-threatening consequences (e.g., perforation)

CLINICAL PARAMETER	GRADE 1 MILD	GRADE 2 MODERATE	GRADE 3 SEVERE	GRADE 4 POTENTIALLY LIFE-THREATENING
Vomiting	Transient or intermittent vomiting with no or minimal interference with oral intake	Frequent episodes of vomiting with no or mild dehydration	Persistent vomiting resulting in orthostatic hypotension OR Aggressive rehydration indicated (e.g., IV fluids)	Life-threatening consequences (e.g., hypotensive shock)
NEUROLOGIC				
Alteration in personality-behavior or in mood (e.g., agitation, anxiety, depression, mania, psychosis)	Alteration causing no or minimal interference with usual social & functional activities	Alteration causing greater than minimal interference with usual social & functional activities	Alteration causing inability to perform usual social & functional activities	Behavior potentially harmful to self or others (e.g., suicidal and homicidal ideation or attempt, acute psychosis) OR Causing inability to perform basic self-care functions
Altered Mental Status<br>For Dementia, see Cognitive and behavioral/attention al disturbance (including dementia and attention deficit disorder)	Changes causing no or minimal interference with usual social & functional activities	Mild lethargy or somnolence causing greater than minimal interference with usual social & functional activities	Confusion, memory impairment, lethargy, or somnolence causing inability to perform usual social & functional activities	Delirium OR obtundation, OR coma
Ataxia	Asymptomatic ataxia detectable on exam OR Minimal ataxia causing no or minimal interference with usual social & functional activities	Symptomatic ataxia causing greater than minimal interference with usual social & functional activities	Symptomatic ataxia causing inability to perform usual social & functional activities	Disabling ataxia causing inability to perform basic self-care functions
Cognitive and behavioral/attention al disturbance (including dementia and attention deficit disorder)	Disability causing no or minimal interference with usual social & functional activities OR Specialized resources not indicated	Disability causing greater than minimal interference with usual social & functional activities OR Specialized resources on part-time basis indicated	Disability causing inability to perform usual social & functional activities OR Specialized resources on a full-time basis indicated	Disability causing inability to perform basic self-care functions OR Institutionalization indicated
CNS ischemia (acute)	NA	NA	Transient ischemic attack	Cerebral vascular accident (CVA, stroke) with neurological deficit

CLINICAL PARAMETER	GRADE 1 MILD	GRADE 2 MODERATE	GRADE 3 SEVERE	GRADE 4 POTENTIALLY LIFE-THREATENING
Developmental delay – Paediatric ≤ 16 years	Mild developmental delay, either motor or cognitive, as determined by comparison with a developmental screening tool appropriate for the setting	Moderate developmental delay, either motor or cognitive, as determined by comparison with a developmental screening tool appropriate for the setting	Severe developmental delay, either motor or cognitive, as determined by comparison with a developmental screening tool appropriate for the setting	Developmental regression, either motor or cognitive, as determined by comparison with a developmental screening tool appropriate for the setting
Headache	Symptoms causing no or minimal interference with usual social & functional activities	Symptoms causing greater than minimal interference with usual social & functional activities	Symptoms causing inability to perform usual social & functional activities	Symptoms causing inability to perform basic self-care functions OR Hospitalization indicated (other than emergency room visit) OR Headache with significant impairment of alertness or other neurologic function
Insomnia	NA	Difficulty sleeping causing greater than minimal interference with usual social & functional activities	Difficulty sleeping causing inability to perform usual social & functional activities	Disabling insomnia causing inability to perform basic self-care functions
Neuromuscular weakness (including myopathy & neuropathy)	Asymptomatic with decreased strength on exam OR Minimal muscle weakness causing no or minimal interference with usual social & functional activities	Muscle weakness causing greater than minimal interference with usual social & functional activities	Muscle weakness causing inability to perform usual social & functional activities	Disabling muscle weakness causing inability to perform basic self-care functions OR Respiratory muscle weakness impairing ventilation
Neurosensory alteration (including paresthesia and painful neuropathy)	Asymptomatic with sensory alteration on exam or minimal paresthesia causing no or minimal interference with usual social & functional activities	Sensory alteration or paresthesia causing greater than minimal interference with usual social & functional activities	Sensory alteration or paresthesia causing inability to perform usual social & functional activities	Disabling sensory alteration or paresthesia causing inability to perform basic self-care functions

CLINICAL				
**PARAMETER**	**GRADE 1<br>MILD**	**GRADE 2<br>MODERATE**	**GRADE 3<br>SEVERE**	**GRADE 4<br>POTENTIALLY LIFE-THREATENING**
Seizure: (new onset) – Adult ≥ 18 years<br>See also Seizure: (known pre-existing seizure disorder)	NA	1 seizure	2 – 4 seizures	Seizures of any kind which are prolonged, repetitive (e.g., status epilepticus), or difficult to control (e.g., refractory epilepsy)
Seizure: (known pre-existing seizure disorder)<br>– Adult ≥ 18 years<br>For worsening of existing epilepsy the grades should be based on an increase from previous level of control to any of these levels.	NA	Increased frequency of pre-existing seizures (non-repetitive) without change in seizure character OR Infrequent break-through seizures while on stable medication in a previously controlled seizure disorder	Change in seizure character from baseline either in duration or quality (e.g., severity or focality)	Seizures of any kind which are prolonged, repetitive (e.g., status epilepticus), or difficult to control (e.g., refractory epilepsy)
Seizure<br>– Paediatric < 18 years	Seizure, generalized onset with or without secondary generalization, lasting < 5 minutes with < 24 hours post ictal state	Seizure, generalized onset with or without secondary generalization, lasting 5 – 20 minutes with < 24 hours post ictal state	Seizure, generalized onset with or without secondary generalization, lasting > 20 minutes	Seizure, generalized onset with or without secondary generalization, requiring intubation and sedation
Syncope (not associated with a procedure)	NA	Present	NA	NA
Vertigo	Vertigo causing no or minimal interference with usual social & functional activities	Vertigo causing greater than minimal interference with usual social & functional activities	Vertigo causing inability to perform usual social & functional activities	Disabling vertigo causing inability to perform basic self-care functions
**RESPIRATORY**				
Bronchospasm (acute)	FEV1 or peak flow reduced to 70 – 80%	FEV1 or peak flow 50 – 69%	FEV1 or peak flow 25 – 49%	Cyanosis OR FEV1 or peak flow < 25% OR Intubation
Dyspnea or respiratory distress				
Adult ≥ 14 years	Dyspnea on exertion with no or minimal interference with usual social & functional activities	Dyspnea on exertion causing greater than minimal interference with usual social & functional activities	Dyspnea at rest causing inability to perform usual social & functional activities	Respiratory failure with ventilatory support indicated

CLINICAL				
**PARAMETER**	**GRADE 1 MILD**	**GRADE 2 MODERATE**	**GRADE 3 SEVERE**	**GRADE 4 POTENTIALLY LIFE-THREATENING**
Paediatric < 14 years	Wheezing OR minimal increase in respiratory rate for age	Nasal flaring OR Intercostal retractions OR Pulse oximetry 90 – 95%	Dyspnea at rest causing inability to perform usual social & functional activities OR Pulse oximetry < 90%	Respiratory failure with ventilatory support indicated
**MUSCULOSKELETAL**				
Arthralgia<br>See also Arthritis	Joint pain causing no or minimal interference with usual social & functional activities	Joint pain causing greater than minimal interference with usual social & functional activities	Joint pain causing inability to perform usual social & functional activities	Disabling joint pain causing inability to perform basic self-care functions
Arthritis<br>See also Arthralgia	Stiffness or joint swelling causing no or minimal interference with usual social & functional activities	Stiffness or joint swelling causing greater than minimal interference with usual social & functional activities	Stiffness or joint swelling causing inability to perform usual social & functional activities	Disabling joint stiffness or swelling causing inability to perform basic self-care functions
Bone Mineral Loss				
Adult ≥ 21 years	BMD t-score<br>-2.5 to -1.0	BMD t-score < -2.5	Pathological fracture (including loss of vertebral height)	Pathologic fracture causing life-threatening consequences
Paediatric < 21 years	BMD z-score<br>-2.5 to -1.0	BMD z-score < -2.5	Pathological fracture (including loss of vertebral height)	Pathologic fracture causing life-threatening consequences
Myalgia<br>(non-injection site)	Muscle pain causing no or minimal interference with usual social & functional activities	Muscle pain causing greater than minimal interference with usual social & functional activities	Muscle pain causing inability to perform usual social & functional activities	Disabling muscle pain causing inability to perform basic self-care functions
Osteonecrosis	NA	Asymptomatic with radiographic findings AND No operative intervention indicated	Symptomatic bone pain with radiographic findings OR Operative intervention indicated	Disabling bone pain with radiographic findings causing inability to perform basic self-care functions
**GENITOURINARY**				

CLINICAL				
**PARAMETER**	**GRADE 1 MILD**	**GRADE 2 MODERATE**	**GRADE 3 SEVERE**	**GRADE 4 POTENTIALLY LIFE-THREATENING**
Cervicitis (symptoms) (For use in studies evaluating topical study agents) For other cervicitis see Infection: Infection (any other than HIV infection)	Symptoms causing no or minimal interference with usual social & functional activities	Symptoms causing greater than minimal interference with usual social & functional activities	Symptoms causing inability to perform usual social & functional activities	Symptoms causing inability to perform basic self-care functions
Cervicitis (clinical exam) (For use in studies evaluating topical study agents) For other cervicitis see Infection: Infection (any other than HIV infection)	Minimal cervical abnormalities on examination (erythema, mucopurulent discharge, or friability) OR Epithelial disruption < 25% of total surface	Moderate cervical abnormalities on examination (erythema, mucopurulent discharge, or friability) OR Epithelial disruption of 25 – 49% total surface	Severe cervical abnormalities on examination (erythema, mucopurulent discharge, or friability) OR Epithelial disruption 50 – 75% total surface	Epithelial disruption > 75% total surface
Inter-menstrual bleeding (IMB)	Spotting observed by participant OR Minimal blood observed during clinical or colposcopic examination	Inter-menstrual bleeding not greater in duration or amount than usual menstrual cycle	Inter-menstrual bleeding greater in duration or amount than usual menstrual cycle	Hemorrhage with life-threatening hypotension OR Operative intervention indicated
Urinary tract obstruction (e.g., stone)	NA	Signs or symptoms of urinary tract obstruction without hydronephrosis or renal dysfunction	Signs or symptoms of urinary tract obstruction with hydronephrosis or renal dysfunction	Obstruction causing life-threatening consequences
Vulvovaginitis (symptoms) (Use in studies evaluating topical study agents) For other vulvovaginitis see Infection: Infection (any other than HIV infection)	Symptoms causing no or minimal interference with usual social & functional activities	Symptoms causing greater than minimal interference with usual social & functional activities	Symptoms causing inability to perform usual social & functional activities	Symptoms causing inability to perform basic self-care functions

CLINICAL				
**PARAMETER**	**GRADE 1**<br>**MILD**	**GRADE 2**<br>**MODERATE**	**GRADE 3**<br>**SEVERE**	**GRADE 4**<br>**POTENTIALLY LIFE-THREATENING**
Vulvovaginitis (clinical exam)<br>(Use in studies evaluating topical study agents)<br>For other vulvovaginitis see Infection: Infection (any other than HIV infection)	Minimal vaginal abnormalities on examination OR Epithelial disruption < 25% of total surface	Moderate vaginal abnormalities on examination OR Epithelial disruption of 25 - 49% total surface	Severe vaginal abnormalities on examination OR Epithelial disruption 50 - 75% total surface	Vaginal perforation OR Epithelial disruption > 75% total surface
**OCULAR/VISUAL**				
Uveitis	Asymptomatic but detectable on exam	Symptomatic anterior uveitis OR Medical intervention indicated	Posterior or pan-uveitis OR Operative intervention indicated	Disabling visual loss in affected eye(s)
Visual changes (from baseline)	Visual changes causing no or minimal interference with usual social & functional activities	Visual changes causing greater than minimal interference with usual social & functional activities	Visual changes causing inability to perform usual social & functional activities	Disabling visual loss in affected eye(s)
**ENDOCRINE/METABOLIC**				
Abnormal fat accumulation<br>(e.g., back of neck, breasts, abdomen)	Detectable by study participant (or by caregiver for young children and disabled adults)	Detectable on physical exam by health care provider	Disfiguring OR Obvious changes on casual visual inspection	NA
Diabetes mellitus	NA	New onset without need to initiate medication OR Modification of current medications to regain glucose control	New onset with initiation of medication indicated OR Diabetes uncontrolled despite treatment modification	Life-threatening consequences (e.g., ketoacidosis, hyperosmolar non-ketotic coma)
Gynecomastia	Detectable by study participant or caregiver (for young children and disabled adults)	Detectable on physical exam by health care provider	Disfiguring OR Obvious on casual visual inspection	NA

CLINICAL				
**PARAMETER**	**GRADE 1 MILD**	**GRADE 2 MODERATE**	**GRADE 3 SEVERE**	**GRADE 4 POTENTIALLY LIFE-THREATENING**
Hyperthyroidism	Asymptomatic	Symptomatic causing greater than minimal interference with usual social & functional activities OR Thyroid suppression therapy indicated	Symptoms causing inability to perform usual social & functional activities OR Uncontrolled despite treatment modification	Life-threatening consequences (e.g., thyroid storm)
Hypothyroidism	Asymptomatic	Symptomatic causing greater than minimal interference with usual social & functional activities OR Thyroid replacement therapy indicated	Symptoms causing inability to perform usual social & functional activities OR Uncontrolled despite treatment modification	Life-threatening consequences (e.g., myxedema coma)
Lipoatrophy (e.g., fat loss from the face, extremities, buttocks)	Detectable by study participant (or by caregiver for young children and disabled adults)	Detectable on physical exam by health care provider	Disfiguring OR Obvious on casual visual inspection	NA

LABORATORY				
**PARAMETER**	**GRADE 1 MILD**	**GRADE 2 MODERATE**	**GRADE 3 SEVERE**	**GRADE 4 POTENTIALLY LIFE-THREATENING**
**HAEMATOLOGY**	**Standard International Units are listed in italics**			
Absolute CD4+ count – Adult and Paediatric > 13 years (HIV negative only)	300 – 400/mm^3<br>300 – 400/µL	200 – 299/mm^3<br>200 – 299/µL	100 – 199/mm^3<br>100 – 199/µL	< 100/mm^3<br>< 100/µL
Absolute lymphocyte count – Adult and Paediatric > 13 years (HIV negative only)	600 – 650/mm^3<br>0.600 x 109 – 0.650 x 109/L	500 – 599/mm^3<br>0.500 x 109 – 0.599 x 109/L	350 – 499/mm^3<br>0.350 x 109 – 0.499 x 109/L	< 350/mm^3<br>< 0.350 x 109/L
Comment: Values in children ≤ 13 years are not given for the two parameters above because the absolute counts are variable				
Absolute neutrophil count (ANC) [51, 81]				
Adult and Paediatric, > 7 days	750 –< 1,000/mm3<br>0.75 x 109 – <1.0. x 109/L	500 – 749/mm3<br>0.5 x 109 – 0.749 x 109/L	250 – 499/mm3<br>0.25 x 109 – 0.499 x 109/L	< 250/mm3<br>< 0.250 x 109/L

LABORATORY PARAMETER	GRADE 1 MILD	GRADE 2 MODERATE	GRADE 3 SEVERE	GRADE 4 POTENTIALLY LIFE-THREATENING
Infant∗†, 2 – ≤ 7 days	1,250 – 1,500/mm3<br>1.250 x 109 –<br>1.500 x 109/L	1,000 – 1,249/mm3<br>1.000 x 109 –<br>1.249 x 109/L	750 – 999/mm3<br>0.750 x 109 –<br>0.999 x 109/L	< 750/mm3<br>< 0.750 x 109/L
Infant∗†, ≤1 day	4,000 – 5,000/mm3<br>4.000 x 109 –<br>5.000 x 109/L	3,000 – 3,999/mm3<br>3.000 x 109 –<br>3.999 x109/L	1,500 – 2,999/mm3<br>1.500 x 109 –<br>2.999 x 109/L	< 1,500/mm3<br>< 1.500 x 109/L
Fibrinogen, decreased	100 – 200 mg/dL<br>1.00 – 2.00 g/L<br>OR<br>0.75 – 0.99 x LLN	75 – 99 mg/dL<br>0.75 – 0.99 g/L<br>OR<br>0.50 – 0.74 x LLN	50 – 74 mg/dL<br>0.50 – 0.74 g/L<br>OR<br>0.25 – 0.49 x LLN	< 50 mg/dL<br>< 0.50 g/L<br>OR<br>< 0.25 x LLN<br>OR<br>Associated with gross bleeding
Haemoglobin (Hgb)				
Comment: The Hgb values in mmol/L have changed because the conversion factor used to convert g/dL to mmol/L has been changed from 0.155 to 0.6206 (the most commonly used conversion factor). For grading Hgb results obtained by an analytic method with a conversion factor other than 0.6206, the result must be converted to g/dL using the appropriate conversion factor for that lab.				
Adult and Paediatric ≥ 57 days (HIV positive only)	8.5 – 10.0 g/dL<br>5.24 –6.23 mmol/L	7.5 – 8.4 g/dL<br>4.62-5.23 mmol/L	6.50 – 7.4 g/dL<br>4.03-4.61 mmol/L	< 6.5 g/dL<br>< 4.03 mmol/L
Adult and Paediatric ≥ 57 days (HIV negative only)	10.0 – 10.9 g/dL<br>6.18 – 6.79 mmol/L<br>OR<br>Any decrease<br>2.5 – 3.4 g/dL<br>1.58 – 2.13 mmol/L	9.0 – 9.9 g/dL<br>5.55 – 6.17 mmol/L<br>OR<br>Any decrease<br>3.5 – 4.4 g/dL<br>2.14 – 2.78 mmol/L	7.0 – 8.9 g/dL<br>4.34 – 5.54 mmol/L<br>OR<br>Any decrease<br>≥ 4.5 g/dL<br>≥ 2.79 mmol/L	< 7.0 g/dL<br>< 4.34 mmol/L
Comment: The decrease is a decrease from baseline				
Infant∗†, 36 – 56 days (HIV positive or negative)	8.5 – 9.4 g/dL<br>5.24 – 5.86 mmol/L	7.0 – 8.4 g/dL<br>4.31 – 5.23 mmol/L	6.0 – 6.9 g/dL<br>3.72 – 4.30 mmol/L	< 6.00 g/dL<br>< 3.72 mmol/L
Infant∗†, 22 – 35 days (HIV positive or negative)	9.5 – 10.5 g/dL<br>5.86 – 6.54 mmol/L	8.0 – 9.4 g/dL<br>4.93 – 5.86 mmol/L	7.0 – 7.9 g/dL<br>4.34 – 4.92 mmol/L	< 7.00 g/dL<br>< 4.34 mmol/L
Infant∗†, ≤21 days (HIV positive or negative)	12.0 – 13.0 g/dL<br>7.42 – 8.09 mmol/L	10.0 – 11.9 g/dL<br>6.18 – 7.41 mmol/L	9.0 – 9.9 g/dL<br>5.59 – 6.17 mmol/L	< 9.0 g/dL<br>< 5.59mmol/L
Comment: Parametre changed from “Infant <21 days” to “Infants ≤ 21 days”				

LABORATORY PARAMETER	GRADE 1 MILD	GRADE 2 MODERATE	GRADE 3 SEVERE	GRADE 4 POTENTIALLY LIFE-THREATENING
International Normalized Ratio of prothrombin time (INR)	1.1 – 1.5 x ULN	1.6 – 2.0 x ULN	2.1 – 3.0 x ULN	> 3.0 x ULN
Methemoglobin	5.0 – 10.0%	10.1 – 15.0%	15.1 – 20.0%	> 20.0%
Prothrombin Time (PT)	1.1 – 1.25 x ULN	1.26 – 1.50 x ULN	1.51 – 3.00 x ULN	> 3.00 x ULN
Partial Thromboplastin Time (PTT)	1.1 – 1.66 x ULN	1.67 – 2.33 x ULN	2.34 – 3.00 x ULN	> 3.00 x ULN
Platelets, decreased	100,000 – 124,999/mm3<br>100.000 x 109 – 124.999 x 109/L	50,000 – 99,999/mm3<br>50.000 x 109 – 99.999 x 109/L	25,000 – 49,999/mm3<br>25.000 x 109 – 49.999 x 109/L	< 25,000/mm3<br>< 25.000 x 109/L
WBC, decreased	2,000 – 2,500/mm3<br>2.000 x 109 – 2.500 x 109/L	1,500 – 1,999/mm3<br>1.500 x 109 – 1.999 x 109/L	1,000 – 1,499/mm3<br>1.000 x 109 – 1.499 x 109/L	< 1,000/mm3<br>< 1.000 x 109/L
**CHEMISTRIES**	**Standard International Units are listed in italics**			
Acidosis	NA	pH < normal, but ≥ 7.3	pH < 7.3 without life-threatening consequences	pH < 7.3 with life-threatening consequences
Albumin, serum, low	3.0 g/dL – < LLN<br>30 g/L – < LLN	2.0 – 2.9 g/dL<br>20 – 29 g/L	< 2.0 g/dL<br>< 20 g/L	NA
Alkaline Phosphatase	1.25 – 2.5 x ULN†	2.6 – 5.0 x ULN†	5.1 – 10.0 x ULN†	> 10.0 x ULN†
Alkalosis	NA	pH > normal, but ≤ 7.5	pH > 7.5 without life-threatening consequences	pH > 7.5 with life-threatening consequences
ALT (SGPT)	1.25 – 2.5 x ULN	2.6 – 5.0 x ULN	5.1 – 10.0 x ULN	> 10.0 x ULN
AST (SGOT)	1.25 – 2.5 x ULN	2.6 – 5.0 x ULN	5.1 – 10.0 x ULN	> 10.0 x ULN
Bicarbonate, serum, low	16.0 mEq/L – < LLN<br>16.0 mmol/L – < LLN	11.0 – 15.9 mEq/L<br>11.0 – 15.9 mmol/L	8.0 – 10.9 mEq/L<br>8.0 – 10.9 mmol/L	< 8.0 mEq/L<br>< 8.0 mmol/L
Comment : Some laboratories will report this value as Bicarbonate (HCO3) and others as Total Carbon Dioxide (CO2). These are the same tests, values should be graded according to the ranges for Bicarbonate as listed above.				
Bilirubin (Total)				
Adult and Paediatric > 14 days	1.1 – 1.5 x ULN	1.6 – 2.5 x ULN	2.6 – 5.0 x ULN	> 5.0 x ULN
Infant∗†, ≤ 14 days (non-hemolytic)	NA	20.0 – 25.0 mg/dL<br>342 – 428 μmol/L	25.1 – 30.0 mg/dL<br>429 – 513 μmol/L	> 30.0 mg/dL<br>> 513.0 μmol/L
Infant∗†, ≤ 14 days (hemolytic)	NA	NA	20.0 – 25.0 mg/dL<br>342 – 428 μmol/L	> 25.0 mg/dL<br>> 428 μmol/L

LABORATORY					
**PARAMETER**		**GRADE 1 MILD**	**GRADE 2 MODERATE**	**GRADE 3 SEVERE**	**GRADE 4 POTENTIALLY LIFE-THREATENING**
Calcium, serum, high (corrected for albumin)					
	Adult and Paediatric ≥ 7 days	10.6 – 11.5 mg/dL<br>2.65 – 2.88 mmol/L	11.6 – 12.5 mg/dL<br>2.89 – 3.13 mmol/L	12.6 – 13.5 mg/dL<br>3.14 – 3.38 mmol/L	> 13.5 mg/dL<br>> 3.38 mmol/L
	Infant∗†, < 7 days	11.5 – 12.4 mg/dL<br>2.88 – 3.10 mmol/L	12.5 – 12.9 mg/dL<br>3.11 – 3.23 mmol/L	13.0 – 13.5 mg/dL<br>3.245 – 3.38 mmol/L	> 13.5 mg/dL<br>> 3.38 mmol/L
Calcium, serum, low (corrected for albumin)					
	Adult and Paediatric ≥ 7 days	7.8 – 8.4 mg/dL<br>1.95 – 2.10 mmol/L	7.0 – 7.7 mg/dL<br>1.75 – 1.94 mmol/L	6.1 – 6.9 mg/dL<br>1.53 – 1.74 mmol/L	< 6.1 mg/dL<br>< 1.53 mmol/L
	Infant∗†, < 7 days	6.5 – 7.5 mg/dL<br>1.63 – 1.88 mmol/L	6.0 – 6.4 mg/dL<br>1.50 – 1.62 mmol/L	5.50 – 5.90 mg/dL<br>1.38 – 1.51 mmol/L	< 5.50 mg/dL<br>< 1.38 mmol/L
Comment: Do not adjust Calcium, serum, low or Calcium, serum, high for albumin					
Cardiac troponin I (cTnI)		NA	NA	NA	Levels consistent with myocardial infarction or unstable angina as defined by the manufacturer
Cardiac troponin T (cTnT)		NA	NA	NA	≥ 0.20 ng/mL<br>OR<br>Levels consistent with myocardial infarction or unstable angina as defined by the manufacturer
Cholesterol (fasting)					
	Adult ≥ 18 years	200 – 239 mg/dL<br>5.18 – 6.19 mmol/L	240 – 300 mg/dL<br>6.20 – 7.77 mmol/L	> 300 mg/dL<br>> 7.77 mmol/L	NA
	Paediatric < 18 years	170 – 199 mg/dL<br>4.40 – 5.15 mmol/L	200 – 300 mg/dL<br>5.16 – 7.77 mmol/L	> 300 mg/dL<br>> 7.77 mmol/L	NA
Creatine Kinase		3.0 – 5.9 x ULN†	6.0 – 9.9 x ULN†	10.0 – 19.9 x ULN†	≥ 20.0 x ULN†
Creatinine		1.1 – 1.3 x ULN†	1.4 – 1.8 x ULN†	1.9 – 3.4 x ULN†	≥ 3.5 x ULN†
Glucose, serum, high					
	Nonfasting	116 – 160 mg/dL<br>6.44 – 8.88 mmol/L	161 – 250 mg/dL<br>8.89 – 13.88 mmol/L	251 – 500 mg/dL<br>13.89 – 27.75 mmol/L	> 500 mg/dL<br>> 27.75 mmol/L
	Fasting	110 – 125 mg/dL<br>6.11 – 6.94 mmol/L	126 – 250 mg/dL<br>6.95 – 13.88 mmol/L	251 – 500 mg/dL<br>13.89 – 27.75 mmol/L	> 500 mg/dL<br>> 27.75 mmol/L
Glucose, serum, low					
	Adult and Paediatric ≥ 1 month	55 – 64 mg/dL<br>3.05 – 3.55 mmol/L	40 – 54 mg/dL<br>2.22 – 3.06 mmol/L	30 – 39 mg/dL<br>1.67 – 2.23 mmol/L	< 30 mg/dL<br>< 1.67 mmol/L
	Infant∗†, < 1 month	50 – 54 mg/dL<br>2.78 – 3.00 mmol/L	40 – 49 mg/dL<br>2.22 – 2.77 mmol/L	30 – 39 mg/dL<br>1.67 – 2.21 mmol/L	< 30 mg/dL<br>< 1.67 mmol/L

LABORATORY					
**PARAMETER**		**GRADE 1 MILD**	**GRADE 2 MODERATE**	**GRADE 3 SEVERE**	**GRADE 4 POTENTIALLY LIFE-THREATENING**
Lactate		ULN- < 2.0 x ULN without acidosis	≥ 2.0 x ULN without acidosis	Increased lactate with pH < 7.3 without life-threatening consequences	Increased lactate with pH < 7.3 with life-threatening consequences
Comment: Added ULN to grade 1 parameter					
LDL cholesterol (fasting)					
	Adult ≥ 18 years	130 – 159 mg/dL<br>3.37 – 4.12 mmol/L	160 – 190 mg/dL<br>4.13 – 4.90 mmol/L	≥ 190 mg/dL<br>≥ 4.91 mmol/L	NA
	Paediatric > 2 - < 18 years	110 – 129 mg/dL<br>2.85 – 3.34 mmol/L	130 – 189 mg/dL<br>3.35 – 4.90 mmol/L	≥ 190 mg/dL<br>≥ 4.91 mmol/L	NA
Lipase		1.1 – 1.5 x ULN	1.6 – 3.0 x ULN	3.1 – 5.0 x ULN	> 5.0 x ULN
Magnesium, serum, low		1.2 – 1.4 mEq/L<br>0.60 – 0.70 mmol/L	0.9 – 1.1 mEq/L<br>0.45 – 0.59 mmol/L	0.6 – 0.8 mEq/L<br>0.30 – 0.44 mmol/L	< 0.60 mEq/L<br>< 0.30 mmol/L
Pancreatic amylase		1.1 – 1.5 x ULN	1.6 – 2.0 x ULN	2.1 – 5.0 x ULN	> 5.0 x ULN
Phosphate, serum, low					
	Adult and Paediatric > 14 years	2.5 mg/dL – < LLN<br>0.81 mmol/L – < LLN	2.0 – 2.4 mg/dL<br>0.65 – 0.80 mmol/L	1.0 – 1.9 mg/dL<br>0.32 – 0.64 mmol/L	< 1.00 mg/dL<br>< 0.32 mmol/L
	Paediatric 1 year – 14 years	3.0 – 3.5 mg/dL<br>0.97 – 1.13 mmol/L	2.5 – 2.9 mg/dL<br>0.81 – 0.96 mmol/L	1.5 – 2.4 mg/dL<br>0.48 – 0.80 mmol/L	< 1.50 mg/dL<br>< 0.48 mmol/L
	Paediatric < 1 year	3.5 – 4.5 mg/dL<br>1.13 – 1.45 mmol/L	2.5 – 3.4 mg/dL<br>0.81 – 1.12 mmol/L	1.5 – 2.4 mg/dL<br>0.48 – 0.80 mmol/L	< 1.50 mg/dL<br>< 0.48 mmol/L
Potassium, serum, high		5.6 – 6.0 mEq/L<br>5.6 – 6.0 mmol/L	6.1 – 6.5 mEq/L<br>6.1 – 6.5 mmol/L	6.6 – 7.0 mEq/L<br>6.6 – 7.0 mmol/L	> 7.0 mEq/L<br>> 7.0 mmol/L
Potassium, serum, low		3.0 – 3.4 mEq/L<br>3.0 – 3.4 mmol/L	2.5 – 2.9 mEq/L<br>2.5 – 2.9 mmol/L	2.0 – 2.4 mEq/L<br>2.0 – 2.4 mmol/L	< 2.0 mEq/L<br>< 2.0 mmol/L
Sodium, serum, high		146 – 150 mEq/L<br>146 – 150 mmol/L	151 – 154 mEq/L<br>151 – 154 mmol/L	155 – 159 mEq/L<br>155 – 159 mmol/L	≥ 160 mEq/L<br>≥ 160 mmol/L
Sodium, serum, low		130 – 135 mEq/L<br>130 – 135 mmol/L	125 – 129 mEq/L<br>125 – 129 mmol/L	121 – 124 mEq/L<br>121 – 124 mmol/L	≤ 120 mEq/L<br>≤ 120 mmol/L
Triglycerides (fasting)		NA	500 – 750 mg/dL<br>5.65 – 8.48 mmol/L	751 – 1,200 mg/dL<br>8.49 – 13.56 mmol/L	> 1,200 mg/dL<br>> 13.56 mmol/L
Uric acid		7.5 – 10.0 mg/dL<br>0.45 – 0.59 mmol/L	10.1 – 12.0 mg/dL<br>0.60 – 0.71 mmol/L	12.1 – 15.0 mg/dL<br>0.72 – 0.89 mmol/L	> 15.0 mg/dL<br>> 0.89 mmol/L
**URINALYSIS**		**Standard International Units are listed in italics**			
Hematuria (microscopic)		6 – 10 RBC/HPF	> 10 RBC/HPF	Gross, with or without clots OR with RBC casts	Transfusion indicated
Proteinuria, random collection		1 +	2 – 3 +	4 +	NA
Proteinuria, 24 hour collection					
	Adult and Paediatric ≥ 10 years	200 – 999 mg/24 h<br>0.200 – 0.999 g/d	1,000 – 1,999 mg/24 h<br>1.000 – 1.999 g/d	2,000 – 3,500 mg/24 h<br>2.000 – 3.500 g/d	> 3,500 mg/24 h<br>> 3.500 g/d

LABORATORY PARAMETER		GRADE 1 MILD	GRADE 2 MODERATE	GRADE 3 SEVERE	GRADE 4 POTENTIALLY LIFE-THREATENING
	Paediatric > 3 mo - < 10 years	201 – 499 mg/m2/24 h<br>0.201 – 0.499 g/d	500 – 799 mg/m2/24 h<br>0.500 – 0.799 g/d	800 – 1,000 mg/m2/24 h<br>0.800 – 1.000 g/d	> 1,000 mg/ m2/24 h<br>> 1.000 g/d

∗Values are for term infants. Preterm infants should be assessed using local normal ranges. †Use age and sex appropriate values (e.g., bilirubin), including preterm infants.

APPENDIX XI – SOCIAL SCIENCE SUBSTUDY

A social science sub-study will allow more in-depth investigation of issues related to acceptability and feasibility of the interventions in the event that some/all of these strategies are found to reduce mortality. The sub-study will primarily investigate the effect of the high pill burden on acceptability and feasibility of implementation and adherence to the proposed interventions. Even if the interventions reduce mortality, they may not be widely acceptable in the community if they make people feel ill when starting ART, and poorer adherence could have long term consequences. Perceptions about the receipt of large numbers of potentially stigmatising pills that make patients feel ill could prevent patients from presenting for treatment. We will therefore explore participants' experiences around the pill burden when starting ART, and particularly increased pill burden from taking additional drugs in relation to feelings of increased well-being on ART.

We will also explore the reasons for late presentation among young people aged 10-24 and adults 25 years and older. An area of interest is what happens to supplementary feeding provided to individuals living in food insecure households so in addition we will, therefore, gather broad information to inform our understanding.

The sub-study will take place in two of the four trial countries, Uganda and Zimbabwe. The study population will comprise 48 trial participants aged 10+ years; 24 in each country (12 participants aged 10-24 and 12 aged 25+ years). In order to explore the effect of pill burden it will be necessary to include participants with both low and high pill burden, while it would be possible to explore the reasons for late presentation, for example, across all arms of the trial. Participants will therefore be purposively selected from the two arms at the extremes of pill burden (the arm with participants receiving enhanced ART + enhanced opportunistic infection prophylaxis + enhanced nutritional support for 12 weeks and the arm where participants are randomised to standard ART + standard opportunistic infection prophylaxis + standard nutritional support).

Purposively selected trial participants who consent to inclusion in the social science sub-study will be asked to take part in two in-depth interviews. The first interview will be conducted within the first 12 weeks of starting the main trial (i.e. during the enhanced intervention period) and the second at a minimum of 8 weeks and a maximum of 20 weeks after the end of the enhanced intervention period. Given the innovative nature of the trial, the anticipated recovery time and the general state of participants' health during and after the intervention period is unknown. The timing of the second interview will therefore be informed by both the state of individual participant's health as well as by ongoing data analysis.

These in-depth interviews will seek to capture the experiences of participants during and after the intervention period, including whether and how their attitudes towards the pill burden may have been influenced by changes in their health, relationships within the household and their willingness and capacity to control others' knowledge of their condition. We will also investigate the factors influencing their late presentation and their attitudes towards their situation during and after the trial. Although randomisation C (nutritional support) is not an explicit focus of our study (i.e. reflected in our sampling strategy) we envisage that the influence and effects of nutrition will be a key theme that will emerge across participants' accounts. We will therefore explore how access to food and nutritional support influences participant' experiences, as well as how food is distributed and consumed within households.

Whilst the primary source of data collection will be in-depth interviews the study may employ additional methods such as focus groups and audio diaries. A number of focus groups (maximum 4 8 per country) may be conducted in each site as a third phase of data collection to enable participants to reflect on the study's preliminary findings and discuss their ideas about the emphasis and direction of the study's dissemination activities. In particular these focus groups will explore issues around acceptability of the intervention to inform the design of both the support and 'messaging' that might accompany any roll-out of the intervention, as well as interventions to engage those at risk of presenting late. Focus groups will be composed of appropriate age and gender groups.

Audio diaries, simple audio devices that participants can use to autonomously record their experiences, may be used between the first and second interviews with some participants. Participants will be invited to keep an audio diary for approximately two weeks. Our previous research has shown this method to be particularly effective in capturing less 'public' accounts, which can provide rich complementary data. In addition their ease of use makes them especially appropriate for participants experiencing fatigue and illness. These will be used in sites in which we are able to collect, transcribe and analyse the diaries in adequate time to inform the second interview topic guides. All interviews, focus groups and audio diaries will be audio recorded, transcribed verbatim and translated into English. Fieldnotes will also be kept.

This study will generate 48 baseline interviews and at least 48 follow-up interviews and a maximum of eight focus groups. Data analysis will be conducted by the centre research teams in collaboration with the UK/Entebbe social science research team throughout the data collection period. A thematic framework will be developed for the analysis based on the research focus, drawing on existing literature, and our emerging findings from the data. This thematic framework will guide our thematic content analysis. Our comparative analyses will investigate participants' experience over time, with a particular focus on how attitudes towards their treatment and health may change over time, as well as examining any differences between intervention arms and sites.

REDUCTION OF EARLY MORTALITY IN HIV-INFECTED ADULTS AND CHILDREN STARTING ANTIRETROVIRAL THERAPY SOCIAL SCIENCE SUB STUDY

INFORMATION SHEET: ***ADULTS: QUALITATIVE STUDY BASELINE***
Version 1.02 Date 06 Dec 2013

What you should know about this research study:

- We give you this consent form so that you may read about the purpose, risks, and benefits of this research study.
- Routine care is based upon the best known treatment and is provided with the main goal of helping the individual patient. The main goal of research studies is to gain knowledge that may help future patients.
- We cannot promise that this research will benefit you. Just like regular care, this research can have side effects that can be serious or minor.
- You have the right to refuse to take part or agree to take part now and change your mind later.
- Whatever you decide will not affect your regular care.
- Please review this consent form carefully. Ask any questions before you make a decision.
- Your participation is voluntary.

INTRODUCTION

As part of the REALITY trial, we are doing a study to hear from young people (aged 10-24 and adults aged 25 and older) about their experiences of HIV. At least 24 young people and 24 adults from the REALITY trial in Uganda and Zimbabwe will take part in this study. If you agree to take part in this study you will be asked to take part in two in-depth interviews and given an opportunity to keep an audio diary. Today's interview will be the first one and the second interview will be conducted between eight and 20 weeks after you have received the intervention. Between today's interview and the follow up interview you will be given an opportunity to keep an audio diary. You may also be invited to participate in a focus group discussion at the end of this study. For the follow up interview and the focus group discussion you will be asked to fill in consent forms specific to the interviews. We are inviting you to join our study.

Before you decide if you want to take part please read and/or listen to this information sheet carefully and ask any questions you may have.

WHAT IS THE PURPOSE OF THE STUDY?

This is a study to find out whether or not giving extra treatments during the first three months of taking ARVs will help sick people with HIV and prevent some of them from dying early, even though they are on ARVs – or whether giving extra treatments makes no difference to survival but makes it harder to take ARVs. As you may or may not know HIV is the virus that causes AIDS. At the moment there is no cure for HIV. However there are anti-HIV medicines, called 'anti-retrovirals' or ARVs, which can control HIV and keep people well for a long time. Unfortunately in many African countries patients often are only able to come for treatment when they are very sick and HIV has already had a big effect on their bodies. For example they may often have malnutrition, diarrhoea, infections such as tuberculosis, serious lung infections (pneumonia) and other severe infections. This means that

more people die sometime during the first three months after starting ARVs; this is quite different compared to patients in rich countries.
You have already agreed to take part in the REALITY trial. The REALITY trial is testing three different ways to improve treatment outcomes in people starting ARVs.

In this REALITY sub study, we want to look in more depth at issues related to the acceptability and feasibility of the three strategies being used to reduce morbidity and mortality being investigated in the main REALITY trial. The study will explore participants' experiences around having to take large numbers of pills every day when they start ART as well as on the acceptability of ready to use food supplements (RTUF). We are interested to explore how the supplementary feeding is used. We will want to find out why some people only attend medical services when their HIV infection is already quite advanced so that we can try to develop programs to make it easier for people to attend.

PROCEDURE AND DURATION

If you agree to take part in the study you will meet a researcher for an interview. The interview will be like a conversation and will last for about an hour. There are no wrong or right answers. You do not have to answer any questions that you do not want to. You can stop the interview at any time, without having to explain why. We would like to record these interviews using a small audio recorder, if this is ok with you. The interview will take place somewhere that you feel comfortable, probably at a clinic. The interviewer will have received special training to conduct this research. You will be asked questions about yourself as well as information about the household in which you live. We will also ask questions about your experiences of starting on ARVs, how you feel about the number of pills you were asked to take, any changes you noticed in your health and also about your relationships with household members. We also want to ask you whether there were any reasons / circumstances that made it difficult to attend HIV services. Participants will also be asked about their nutrition, and the effects and acceptability of food supplements and how food is distributed and consumed in the household.

Audio Diary

In addition to the interview, you also have the option of keeping an audio diary. This is like a spoken diary. We would provide you with a small audio recorder and give you guidance about what topics we would like you to talk about. We would like you to keep this audio diary for two weeks. The researcher would ask you to bring the diary to the clinic. This is just an option. If you want to take part in the interview only and not keep a diary then that is absolutely okay.

RISKS AND DISCOMFORTS

The risks involved in this study are small. Some of the questions that will be asked may evoke sad or unpleasant memories or make you distressed or feel uncomfortable. If you are distressed we can stop the interview. A REALITY trained counsellor will be on site should you require someone to talk to during and after the interview.

BENEFITS

There are no direct benefits to you for participating in the study. Information obtained in this study will be used to the future care of adults and children with HIV in Africa. The findings of this study will be reported to a wide range of people who are involved in providing treatment, care and support for people living with HIV. The findings will be used to guide future interventions to support both young people and adults living with HIV. Your travel expenses for the interview will be reimbursed at not more than equivalent of USD5 per trip.

IS THE RESEARCH CONFIDENTIAL?

Yes. Any information that you share with us will be kept confidential and your name will not appear on the voice recording as only identity numbers will be used to identify the audio file that has your voice. Any written information on the interviews we will have will not have your personal details but will have participant's identity that will be assigned to you on the interview day. All study materials will be kept in a secure room in lockable cabinets. Only authorized members of the research team will have access to them. We will not use your real name whenever we write or talk about anything you have told us.

CONSENTING TO TAKE PART IN THE STUDY
REDUCTION OF EARLY MORTALITY IN HIV-INFECTED ADULTS AND CHILDREN STARTING ANTIRETROVIRAL THERAPY SOCIAL SCIENCE SUB STUDY

Version 1.02 Date 06 Dec 2013

AUTHORIZATION
You are making a decision whether or not to participate in this study. Your signature indicates that you have read and understood the information provided above, have had all your questions answered, and have decided to participate.

STATEMENT OF CONSENT TO BE AUDIO-TAPED
I understand that audio recordings will be taken during the study. (Mark either "Yes" or "No")

I agree to being audio recorded	Yes	☐
	No	☐
I agree to being keep an audio diary	Yes	☐
	No	☐

_______________________________________ ______________
Name of Research Participant (please print) Date

_______________________________________ ______________
Signature of Participant or legally authorized representative Time

_____________________________ _____________________________
Signature of Witness (*If appropriate*) Signature of Research Staff

YOU WILL BE GIVEN A COPY OF THIS CONSENT FORM TO KEEP
If you have any questions concerning this study or consent form beyond those answered by the investigator, including questions about the research, your rights as a research subject or research-related injuries; or if you feel that you have been treated unfairly and would like to talk to someone other than a member of the research team, please feel free to contact the <<***Fill in local contact for Zimbabwe/Uganda*** *using the contact information below.*
Xxx (including phone number)>>

REDUCTION OF EARLY MORTALITY IN HIV-INFECTED ADULTS AND CHILDREN STARTING ANTIRETROVIRAL THERAPY SOCIAL SCIENCE SUB STUDY

INFORMATION SHEET: YOUNG CHILDREN: QUALITATIVE STUDY BASELINE
Version 1.02 Date 06 Dec 2013

What you should know about this research study:

- We give you this consent form so that you may read about the purpose, risks, and benefits of this research study.
- Routine care is based upon the best known treatment and is provided with the main goal of helping the individual patient. The main goal of research studies is to gain knowledge that may help future patients.
- We cannot promise that this research will benefit your child. Just like regular care, this research can have side effects that can be serious or minor.
- You have the right to refuse to allow your child to take part or agree for your child to take part now and change your mind later.
- Whatever you decide will not affect your child's regular care.
- Please review this consent form carefully. Ask any questions before you make a decision.
- Your choice to allow your child to participate is voluntary.

INTRODUCTION

As part of the REALITY trial, we are doing a study to hear from young people (aged 10-24 and adults aged 25 and older) about their experiences of HIV. At least 24 young people and 24 adults from the REALITY trial in Uganda and Zimbabwe will take part in this study. If you agree to take part in this study you will be asked to take part in two in-depth interviews and given an opportunity to keep an audio diary. Today's interview will be the first one and the second interview will be conducted between eight and 20 weeks after you have received the intervention. Between today's interview and the follow up interview you will be given an opportunity to keep an audio diary. You may also be invited to participate in a focus group discussion at the end of this study. For the follow up interview and the focus group discussion you will be asked to fill in consent forms specific to the interviews. We are inviting you to join our study.

Before you decide if you want to take part please read and/or listen to this information sheet carefully and ask any questions you may have.

WHAT IS THE PURPOSE OF THE STUDY?
This is a study to find out whether or not giving extra treatments during the first three months of taking ARVs will help sick people with HIV and prevent some of them from dying early, even though they are on ARVs – or whether giving extra treatments makes no difference to survival but makes it harder to take ARVs. As you may or may not know HIV is the virus that causes AIDS. At the moment there is no cure for HIV. However there are anti-HIV medicines, called 'anti-retrovirals' or ARVs, which can control HIV and keep people well for a long time. Unfortunately in many African countries patients often are only able to come for treatment when they are very sick and HIV has already had a big effect on their bodies. For example they may often have malnutrition, diarrhoea, infections such as tuberculosis, serious lung infections (pneumonia) and other severe infections. This means that

more people die sometime during the first three months after starting ARVs; this is quite different compared to patients in rich countries.

You have already agreed to take part in the REALITY trial. The REALITY trial is testing three different ways to improve treatment outcomes in people starting ARVs.

In this REALITY sub study, we want to look in more depth at issues related to the acceptability and feasibility of the three strategies being used to reduce morbidity and mortality being investigated in the main REALITY trial. The study will explore participants' experiences around having to take large numbers of pills every day when they start ART as well as on the acceptability of ready to use food supplements (RTUF). We are interested to explore how the supplementary feeding is used. We will want to find out why some people only attend medical services when their HIV infection is already quite advanced so that we can try to develop programs to make it easier for people to attend.

PROCEDURE AND DURATION

If you agree to take part in the study you will meet a researcher for an interview. The interview will be like a conversation and will last for about an hour. There are no wrong or right answers. You do not have to answer any questions that you do not want to. You can stop the interview at any time, without having to explain why. We would like to record these interviews using a small audio recorder, if this is ok with you. The interview will take place somewhere that you feel comfortable, probably at a clinic. The interviewer will have received special training to conduct this research. You will be asked questions about yourself as well as information about the household in which you live. We will also ask questions about your experiences of starting on ARVs, how you feel about the number of pills you were asked to take, any changes you noticed in your health and also about your relationships with household members. We also want to ask you whether there were any reasons / circumstances that made it difficult to attend HIV services. Participants will also be asked about their nutrition, and the effects and acceptability of food supplements and how food is distributed and consumed in the household.

Audio Diary

In addition to the interview, you also have the option of keeping an audio diary. This is like a spoken diary. We would provide you with a small audio recorder and give you guidance about what topics we would like you to talk about. We would like you to keep this audio diary for two weeks. The researcher would ask you to bring the diary to the clinic. This is just an option. If you want to take part in the interview only and not keep a diary then that is absolutely okay.

RISKS AND DISCOMFORTS

The risks involved in this study are small. Some of the questions that will be asked may evoke sad or unpleasant memories or make you distressed or feel uncomfortable. If you are distressed we can stop the interview. A REALITY trained counsellor will be on site should you require someone to talk to during and after the interview.

BENEFITS

There are no direct benefits to you for participating in the study. Information obtained in this study will be used to the future care of adults and children with HIV in Africa. The findings of this study will be reported to a wide range of people who are involved in providing treatment, care and support for people living with HIV. The findings will be used to guide future interventions to support both young people and adults living with HIV. Your travel expenses for the interview will be reimbursed at not more than equivalent of USD5 per trip.

IS THE RESEARCH CONFIDENTIAL?

Yes. Any information that you share with us will be kept confidential and your name will not appear on the voice recording as only identity numbers will be used to identify the audio file that has your voice. Any written information on the interviews we will have will not have your personal details but will have participant's identity that will be assigned to you on the interview day. All study materials will be kept in a secure room in lockable cabinets. Only authorized members of the research team will have access to them. We will not use your real name whenever we write or talk about anything you have told us.

For any other questions that you may have about this study, please contact the Study Coordinator,

CONSENTING TO TAKE PART IN THE STUDY
REDUCTION OF EARLY MORTALITY IN HIV-INFECTED ADULTS AND CHILDREN STARTING ANTIRETROVIRAL THERAPY SOCIAL SCIENCE SUB STUDY

Version 1.02 Date 06 Dec 2013

AUTHORIZATION
You are making a decision whether or not to participate in this study. Your signature indicates that you have read and understood the information provided above, have had all your questions answered, and have decided to participate.

STATEMENT OF CONSENT TO BE AUDIO-TAPED
I understand that audio recordings will be taken during the study. (Mark either "Yes" or "No")
I agree to being audio recorded Yes ☐

No ☐

____ ____________
Name of Parent (please print) Date

____ ____________
Signature of Parent or legally authorized representative Time

____
Relationship to the Participant

____
Name of Child (please print)

______________________________ ______________________________________
Signature of Witness Signature of Research Staff
(*If appropriate*)

If you have any questions concerning this study or consent form beyond those answered by the investigator, including questions about the research, your rights as a research Participant or research-related injuries; or if you feel that you have been treated unfairly and would like to talk to someone other than a member of the research team, please feel free to contact the Medical Research Council of Zimbabwe on telephone 791792 or 791193.

Children 12 years and below

My participation in this research study is voluntary. I have read and understood the above information, asked all the questions I had and have agreed to participate. I understand that will be given a copy of this form to keep.

____

Name of Participant

Do you want to participate in the study? **Yes** ☐
No ☐

Children 13 years old to 17 years old

My participation in this research study is voluntary. I have read and understood the above information, asked all the questions I had and have agreed to participate. I understand that will be given a copy of this form to keep.

____

Name of Participant

Do you want to participate in the study? **Yes** ☐
No ☐

____

Signature of Participant

YOU WILL BE GIVEN A COPY OF THIS CONSENT FORM TO KEEP

If you have any questions concerning this study or consent form beyond those answered by the investigator, including questions about the research, your rights as a research subject or research-related injuries; or if you feel that you have been treated unfairly and would like to talk to someone other than a member of the research team, please feel free to contact the *<<**Fill in local contact for Zimbabwe/Uganda** using the contact information below.*
Xxx (including phone number)>>

REDUCTION OF EARLY MORTALITY IN HIV-INFECTED ADULTS AND CHILDREN STARTING ANTIRETROVIRAL THERAPY SOCIAL SCIENCE SUB STUDY

Version 1.02 Date 06 Dec 2013

INFORMATION SHEET: ADULTS: QUALITATIVE STUDY FOLLOW UP

What you should know about this research study:

- We give you this consent form so that you may read about the purpose, risks, and benefits of this research study.
- Routine care is based upon the best known treatment and is provided with the main goal of helping the individual patient. The main goal of research studies is to gain knowledge that may help future patients.
- We cannot promise that this research will benefit you. Just like regular care, this research can have side effects that can be serious or minor.
- You have the right to refuse to take part or agree to take part now and change your mind later.
- Whatever you decide will not affect your regular care.
- Please review this consent form carefully. Ask any questions before you make a decision.
- Your participation is voluntary.

INTRODUCTION

This is the second and last time you will be asked to participate in the REALITY social science study. We have spoken with at least 24 young people and 24 adults from the REALITY trial in Uganda and Zimbabwe, including you, in their first interviews. This has been very helpful and we would like to talk to you for the last time. Although, this interview is a follow up to what we discussed in the previous interview, new things that were not discussed in the first interview will be discussed. For example in this interview we would like to hear about your experiences having participated in this trial for this long. We are inviting you to join the study.

Before you decide if you want to take part please read and/or listen to this information sheet carefully and ask any questions you may have.

WHAT IS THE PURPOSE OF THE STUDY?

This is a study to find out whether or not giving extra treatments during the first three months of taking ARVs will help sick people with HIV and prevent some of them from dying early, even though they are on ARVs – or whether giving extra treatments makes no difference to survival but makes it harder to take ARVs. As you may or may not know HIV is the virus that causes AIDS. At the moment there is no cure for HIV. However there are anti-HIV medicines, called 'anti-retrovirals' or ARVs, which can control HIV and keep people well for a long time. Unfortunately in many African countries patients often are only able to come for treatment when they are very sick and HIV has already had a big effect on their bodies. For example they may often have malnutrition, diarrhoea, infections such as tuberculosis, serious lung infections (pneumonia) and other severe infections. This means that more people die sometime during the first three months after starting ARVs; this is quite different compared to patients in rich countries.

You have already agreed to take part in the REALITY trial. The REALITY trial is testing three different ways to improve treatment outcomes in people starting ARVs.

In this REALITY sub study, we want to look in more depth at issues related to the acceptability and feasibility of the three strategies being used to reduce morbidity and mortality being investigated in the main REALITY trial. The study will explore participants' experiences around having to take large numbers of pills every day when they start ART as well as on the acceptability of ready to use food supplements (RTUF). We are interested to explore how the supplementary feeding is used. We will want to find out why some people only attend medical services when their HIV infection is already quite advanced so that we can try to develop programs to make it easier for people to attend.

PROCEDURE AND DURATION

If you agree to take part in the study you will meet a researcher for an interview. The interview will be like a conversation and will last for about an hour. There are no wrong or right answers. You do not have to answer any questions that you do not want to. You can stop the interview at any time, without having to explain why. We would like to record these interviews using a small audio recorder, if this is ok with you. The interview will take place somewhere that you feel comfortable, probably at a clinic. The interviewer will have received special training to conduct this research. You will be asked questions about yourself as well as information about the household in which you live. We will also ask questions about your experiences of starting on ARVs, how you feel about the number of pills you were asked to take, any changes you noticed in your health and also about your relationships with household members. We also want to ask you whether there were any reasons / circumstances that made it difficult to attend HIV services. Participants will also be asked about their nutrition, and the effects and acceptability of food supplements and how food is distributed and consumed in the household.

RISKS AND DISCOMFORTS

The risks involved in this study are small. Some of the questions that will be asked may evoke sad or unpleasant memories or make you distressed or feel uncomfortable. If you are distressed we can stop the interview. A REALITY trained counsellor will be on site should you require someone to talk to during and after the interview.

BENEFITS

There are no direct benefits to you for participating in the study. Information obtained in this study will be used to the future care of adults and children with HIV in Africa. The findings of this study will be reported to a wide range of people who are involved in providing treatment, care and support for people living with HIV. The findings will be used to guide future interventions to support both young people and adults living with HIV. Your travel expenses for the interview will be reimbursed at not more than equivalent of USD5 per trip.

IS THE RESEARCH CONFIDENTIAL?

Yes. Any information that you share with us will be kept confidential and your name will not appear on the voice recording as only identity numbers will be used to identify the audio file that has your voice. Any written information on the interviews we will have will not have your personal details but will have participant's identity that will be assigned to you on the interview day. All study materials will be kept in a secure room in lockable cabinets. Only authorized members of the research team will have access to them. We will not use your real name whenever we write or talk about anything you have told us.

CONSENTING TO TAKE PART IN THE STUDY
REDUCTION OF EARLY MORTALITY IN HIV-INFECTED ADULTS AND CHILDREN STARTING ANTIRETROVIRAL THERAPY SOCIAL SCIENCE SUB STUDY

Version 1.02 Date 06 Dec 2013

AUTHORIZATION
You are making a decision whether or not to participate in this study. Your signature indicates that you have read and understood the information provided above, have had all your questions answered, and have decided to participate.

STATEMENT OF CONSENT TO BE AUDIO-TAPED
I understand that audio recordings will be taken during the study. (Mark either “Yes” or “No”)
I agree to being audio recorded Yes ☐

No ☐

______________________________________ __________
Name of Research Participant (please print) Date

______________________________________ __________
Signature of Participant or legally authorized representative Time

____________________________ ____________________________________
Signature of Witness (*If appropriate*) Signature of Research Staff

YOU WILL BE GIVEN A COPY OF THIS CONSENT FORM TO KEEP
If you have any questions concerning this study or consent form beyond those answered by the investigator, including questions about the research, your rights as a research subject or research-related injuries; or if you feel that you have been treated unfairly and would like to talk to someone other than a member of the research team, please feel free to contact the *<<**Fill in local contact for Zimbabwe/Uganda** using the contact information below.*
Xxx (including phone number)>>

REDUCTION OF EARLY MORTALITY IN HIV-INFECTED ADULTS AND CHILDREN STARTING ANTIRETROVIRAL THERAPY SOCIAL SCIENCE SUB STUDY

Version 1.02 Date 06 Dec 2013

INFORMATION SHEET: YOUNG CHILDREN–FOLLOW UP: QUALITATIVE STUDY FOLLOW UP

What you should know about this research study:

- We give you this consent form so that you may read about the purpose, risks, and benefits of this research study.
- Routine care is based upon the best known treatment and is provided with the main goal of helping the individual patient. The main goal of research studies is to gain knowledge that may help future patients.
- We cannot promise that this research will benefit your child. Just like regular care, this research can have side effects that can be serious or minor.
- You have the right to refuse to allow your child to take part or agree for your child to take part now and change your mind later.
- Whatever you decide will not affect your child's regular care.
- Please review this consent form carefully. Ask any questions before you make a decision.
- Your choice to allow your child to participate is voluntary.

INTRODUCTION

This is the second and last time you will be asked to participate in the REALITY social science study. We have spoken with at least 24 young people and 24 adults from the REALITY trial in Uganda and Zimbabwe, including you, in their first interviews. This has been very helpful and we would like to talk to you for the last time. Although, this interview is a follow up to what we discussed in the previous interview, new things that were not discussed in the first interview will be discussed. For example in this interview we would like to hear about your experiences having participated in this trial for this long. We are inviting you to join the study.

Before you decide if you want to take part please read and/or listen to this information sheet carefully and ask any questions you may have.

WHAT IS THE PURPOSE OF THE STUDY?

This is a study to find out whether or not giving extra treatments during the first three months of taking ARVs will help sick people with HIV and prevent some of them from dying early, even though they are on ARVs – or whether giving extra treatments makes no difference to survival but makes it harder to take ARVs. As you may or may not know HIV is the virus that causes AIDS. At the moment there is no cure for HIV. However there are anti-HIV medicines, called 'anti-retrovirals' or ARVs, which can control HIV and keep people well for a long time. Unfortunately in many African countries patients often are only able to come for treatment when they are very sick and HIV has already had a big effect on their bodies. For example they may often have malnutrition, diarrhoea, infections such as tuberculosis, serious lung infections (pneumonia) and other severe infections. This means that

more people die sometime during the first three months after starting ARVs; this is quite different compared to patients in rich countries.
You have already agreed to take part in the REALITY trial. The REALITY trial is testing three different ways to improve treatment outcomes in people starting ARVs.

In this REALITY sub study, we want to look in more depth at issues related to the acceptability and feasibility of the three strategies being used to reduce morbidity and mortality being investigated in the main REALITY trial. The study will explore participants' experiences around having to take large numbers of pills every day when they start ART as well as on the acceptability of ready to use food supplements (RTUF). We are interested to explore how the supplementary feeding is used. We will want to find out why some people only attend medical services when their HIV infection is already quite advanced so that we can try to develop programs to make it easier for people to attend.

PROCEDURE AND DURATION
If you agree to take part in the study you will meet a researcher for an interview. The interview will be like a conversation and will last for about an hour. There are no wrong or right answers. You do not have to answer any questions that you do not want to. You can stop the interview at any time, without having to explain why. We would like to record these interviews using a small audio recorder, if this is ok with you. The interview will take place somewhere that you feel comfortable, probably at a clinic. The interviewer will have received special training to conduct this research. You will be asked questions about yourself as well as information about the household in which you live. We will also ask questions about your experiences of starting on ARVs, how you feel about the number of pills you were asked to take, any changes you noticed in your health and also about your relationships with household members. We also want to ask you whether there were any reasons / circumstances that made it difficult to attend HIV services. Participants will also be asked about their nutrition, and the effects and acceptability of food supplements and how food is distributed and consumed in the household.

RISKS AND DISCOMFORTS
The risks involved in this study are small. Some of the questions that will be asked may evoke sad or unpleasant memories or make you distressed or feel uncomfortable. If you are distressed we can stop the interview. A REALITY trained counsellor will be on site should you require someone to talk to during and after the interview.

BENEFITS
There are no direct benefits to you for participating in the study. Information obtained in this study will be used to the future care of adults and children with HIV in Africa. The findings of this study will be reported to a wide range of people who are involved in providing treatment, care and support for people living with HIV. The findings will be used to guide future interventions to support both young people and adults living with HIV. Your travel expenses for the interview will be reimbursed at not more than equivalent of USD5 per trip.

IS THE RESEARCH CONFIDENTIAL?
Yes. Any information that you share with us will be kept confidential and your name will not appear on the voice recording as only identity numbers will be used to identify the audio file that has your voice. Any written information on the interviews we will have will not have your personal details but will have participant's identity that will be assigned to you on the interview day. All study materials will be kept in a secure room in lockable cabinets. Only authorized members of the research team will have access to them. We will not use your real name whenever we write or talk about anything you have told us.

CONSENTING TO TAKE PART IN THE STUDY
REDUCTION OF EARLY MORTALITY IN HIV-INFECTED ADULTS AND CHILDREN STARTING ANTIRETROVIRAL THERAPY SOCIAL SCIENCE SUB STUDY

Version 1.02 Date 06 Dec 2013

AUTHORIZATION
You are making a decision whether or not to participate in this study. Your signature indicates that you have read and understood the information provided above, have had all your questions answered, and have decided to participate.

STATEMENT OF CONSENT TO BE AUDIO-TAPED
I understand that audio recordings will be taken during the study. (Mark either "Yes" or "No")
I agree to being audio recorded Yes ☐

No ☐

_______________________________________ ____________
Name of Parent (please print) Date

_______________________________________ ____________
Signature of Parent or legally authorized representative Time

____
Relationship to the Participant

____
Name of Child (please print)

_____________________________ _____________________________
Signature of Witness Signature of Research Staff
(*If appropriate*)

If you have any questions concerning this study or consent form beyond those answered by the investigator, including questions about the research, your rights as a research Participant or research-related injuries; or if you feel that you have been treated unfairly and would like to talk to someone other than a member of the research team, please feel free to contact the Medical Research Council of Zimbabwe on telephone 791792 or 791193.

Children 12 years and below

My participation in this research study is voluntary. I have read and understood the above information, asked all the questions I had and have agreed to participate. I understand that will be given a copy of this form to keep.

____
Name of Participant

Do you want to participate in the study? **Yes** ☐

No ☐

Children 13 years old to 17 years old

My participation in this research study is voluntary. I have read and understood the above information, asked all the questions I had and have agreed to participate. I understand that will be given a copy of this form to keep.

____
Name of Participant

Do you want to participate in the study? **Yes** ☐

No ☐

____
Signature of Participant

YOU WILL BE GIVEN A COPY OF THIS CONSENT FORM TO KEEP
If you have any questions concerning this study or consent form beyond those answered by the investigator, including questions about the research, your rights as a research subject or research-related injuries; or if you feel that you have been treated unfairly and would like to talk to someone other than a member of the research team, please feel free to contact the *<<**Fill in local contact for Zimbabwe/Uganda** using the contact information below.*
Xxx (including phone number)>>
