# Supplementary material for: Raltegravir-intensified initial antiretroviral therapy in advanced HIV disease in Africa: A randomised controlled trial
Source: PLoS Med. 2018 Dec 4;15(12):e1002706. doi: 10.1371/journal.pmed.1002706 (PMC6279020; doi:10.1371/journal.pmed.1002706)
Supplement: S1 Text — (DOC) [file pmed.1002706.s003.doc]

# Supplementary Methods

The REALITY (Reducing EArly mortaLITY) trial recruited adults and older children from Zimbabwe (University of Zimbabwe Clinical Research Centre), Uganda (Joint Clinical Research Centre sites in Mbarara, Mbale, Gulu and Fort Portal, overseen by Kampala), Malawi (Department/College of Medicine and Malawi-Liverpool-Wellcome Trust Clinical Research Programme, Blantyre), and Kenya (KEMRI Wellcome Trust Research Programme and Academic Model for the Prevention and Treatment of HIV/AIDS (AMPATH) Centre at Moi Teaching Referral Hospital (MTRH)).

## (a) Raltegravir dosing

Raltegravir is safe and well-tolerated, without unfavourable renal or lipid profiles . It is relatively free of significant interactions with other drugs including anti-infectives and can be co-administered with NNRTI-based antiretroviral therapy (ART) without dose adjustment.

Adults received 400mg raltegravir twice-daily. Adolescents aged 12-18 years and children aged 6-11 years weighing ≥25kg received the standard adult dose of raltegravir (400mg film-coated tablet twice-daily). Children 5-11 years could also receive 6 mg/kg twice-daily of a chewable tablet which could be divided into equal halves. The chewable tablet and the film-coated tablet formulations are not bioequivalent. Children weighing 10 to <14kg received 0.5 chewable tablets am and 1 pm; 14 to <20kg 1 chewable tablets am and pm; 20 to <28kg 1.5 chewable tablets am and pm; 28 to <40kg 2 chewable tablets am and pm; and 40+kg 3 chewable tablets am and pm.

Raltegravir was a fourth drug so no dose adjustment was made in individuals with active tuberculosis treated with rifamycins.

## (b) Further details of enhanced prophylaxis randomization

Participants were also randomized using a factorial design to open-label enhanced-prophylaxis or standard-of-care co-trimoxazole prophylaxis . Enhanced-prophylaxis comprised single-dose albendazole (400mg), 5-days azithromycin (500mg once-daily), 12-weeks fluconazole (100mg once-daily), and 12-weeks of a fixed-dose-combination (FDC) of co-trimoxazole (800/160mg)/isoniazid (300mg)/pyridoxine (25mg) as a scored once-daily tablet. Doses were halved for children 5-<13 years (except albendazole). After 12 weeks, fluconazole was stopped and either co-trimoxazole or the FDC continued in the enhanced-prophylaxis group; co-trimoxazole was continued or switched to the FDC in the standard-prophylaxis group. Isoniazid/pyridoxine use beyond 12 weeks depended on national IPT guidelines. Screening for active tuberculosis disease before randomization used a WHO-based symptom checklist, with sputum examination and chest X-ray where possible. Participants already receiving or needing antimicrobial treatment or prophylaxis pragmatically received it outside the randomized design, and received other prophylaxis according to randomization.

Following national guidelines, all children received routine de-worming at 24 and 48 weeks post-enrolment irrespective of randomization.

## (c) Further details of Ready-to-use Supplementary Food (RUSF) randomization

Participants were also randomized using a factorial design to 12 weeks’ open-label RUSF or standard-of-care (ready-to-use therapeutic food provided from national programmes to those defined according to national guidelines as severely malnourished) . The RUSF intervention was a lipid-based paste made with maize-soya-sorghum aiming to provide an additional 1000 kCal daily in adults and adolescents (2 x 92g foil packets daily), and an additional 500 kCal daily in children (1 x 92g foil packet daily), and was purchased from Valid International. It was fortified with multi-vitamins and multi-minerals (approx 1xRecommended Daily Allowance (RDA)), so that centres which provided these routinely to children did not provide additional multivitamin tablets for the 12 weeks in which trial RUSF was used.

Standard-of-care nutritional support followed current local practice in each centre, based on criteria for body mass index (BMI) and/or mid-upper arm circumference (MUAC) and/or availability of supplements at the time of randomization. Adults and children with mild-moderate malnutrition did not receive any nutritional support following national guidelines at the time the trial was recruiting, except in Kilifi where those with BMI<18.5 received Plumpy Soy or Acha Mum (2 sachets daily). Management of adults with severe malnutrition initiating ART varied across centres: most were provided with micronutrients and advice on food. Additional Ready to Use Therapeutic Food (RUTF) (different multi-vitamin and multi-mineral content to RUSF) was provided to severely malnourished adults with BMI<16-18 or MUAC<16cm in some centres when it was available locally. Any adult, adolescent or child requiring therapeutic feeding according to standard-of-care received this regardless of randomization (estimated <5%). Participants eligible for or receiving food products from national programmes transitioned onto the study product when they finished it if randomized to the enhanced nutritional support and still <12 weeks from randomization.

## (d) Further details of endpoint ascertainment and adjudication

SAEs were defined following the International Committee for Harmonisation as events which led to death, were life-threatening, caused or prolonged hospitalization (excluding elective procedures), caused permanent disability, or were other medical conditions or with a real, not hypothetical risk of one of the previous categories.

HIV-1 RNA VL was assayed blinded to randomization using the Roche COBAS Ampliprep/Taqman v2.0 in Uganda (Joint Clinical Research Centre (JCRC)), the NucliSENS EasyQ HIV-1 v2.0 in Zimbabwe (UZ-CRC, samples run at the Flow Cytometry Centre, Harare) and the Abbott m2000sp/rt 0.6mls protocol in Kenya (samples from AMPATH, Eldoret and KEMRI, Kilifi) and Blantyre (samples run at John Hopkins Research Project Laboratory, Malawi), with lower limit of detection of 20-50 copies/ml. The trial funding was obtained in 2011, and due to the cost of VL and genotyping tests at the time, did not include sufficient funding to test VL at all timepoints in all participants. Because the original intention was not to do these assays in all participants, but rather on as large as possible a random subset given funding available and costs at the end of the trial, we considered that VL should not therefore be a formal secondary outcome (which would usually be assessed in all participants), and therefore clearly specified it in the protocol as an “other” outcome to be assessed in a subset of participants. Costs at the end of the trial meant that ultimately VL could be assayed in stored samples from all participants at baseline, 4, 12, 24 and 48 weeks and these results are reported.

Genotyping of reverse transcriptase was performed on week 48 samples with VL >1000 copies/ml, and of integrase on week 12 samples with VL >1000 copies/ml, using a WHO-accredited in-house assay at JCRC, Kampala. Only samples from Kenya, Uganda and Zimbabwe were available for shipping for genotyping (not Malawi).

Secondary clinical endpoints (including causes of death) and trial-drug relatedness were adjudicated against protocol-defined criteria, and compatibility of the clinical events with IRIS prospectively assessed, by an Endpoint Review Committee (ERC) (majority independent members) blind to trial drugs received, using the available clinical/laboratory data provided by the sites. To identify grade 4 AEs either definitely/probably or definitely/probably/possibly related to raltegravir, the ERC were asked to adjudicate a relationship to raltegravir for all events to avoid providing them with details of interventional drugs actually received which would unblind them. An unblinded clinical reviewer reviewed the grade 3/4 laboratory only events (no clinical event associated) for relatedness to drugs actually received. In analysis, grade 4 AEs related to raltegravir were defined as those adjudicated as related by the ERC and received within 30 days prior to the event.

## (e) Sample size calculation from the trial protocol

1800 adults and children provided at least 80% power to detect a 50% relative reduction in 24 week all-cause mortality from 7% to 3.5%, or a 60% relative reduction from a lower mortality of 5% to 2% (two-sided alpha=0.05) allowing 5% lost to follow-up by 24 weeks, and incorporating a single inflation factor to allow for the factorial design (rates in multiple groups should be lower than that in any single group) and 5% lost to follow-up at 48 weeks. The sample size calculation assumed that at least one of the three interventions tested would be ineffective and not therefore impact sample size, and therefore only a single inflation factor was used. For 90% power, the detectable reductions were 7% to 3% and 6% to 2.2% respectively. If ~10% of patients were already receiving isoniazid/fluconazole prophylaxis or ready-to-use therapeutic food at randomization, the study design retained >80% power to detect slightly larger reductions from 7% to 3% (57% reduction). Randomizing 400 children provided at least 80% power to detect a 0.29 greater absolute increase in weight-for-age from 0 to 24 weeks with any intervention, based on other assumptions above.

## (f) Implementation of the three factorial randomizations

As described in the main Methods, randomization to each of 12-weeks raltegravir-intensification vs standard ART, 12-weeks enhanced anti-infection prophylaxis vs standard-of-care co-trimoxazole prophylaxis, and 12-weeks RUSF vs no routine supplementation was stratified by centre, age (</≥13 years), and the other factorial randomizations. Practically this was implemented using permuted blocks of size 8 or 16 (randomly) to assign participants to one of 8 groups reflecting the 2x2x2 factorial randomization.

## (g) Statistical methods

Time-to-event analyses measured time from randomization, censored at the last clinical follow-up if the outcome had not occurred. The primary analyses stratified for randomization stratification factors (stratified logrank test and stratified Cox regression); results from secondary unstratified analyses were very similar (data not shown). Lost-to-follow-up was defined as not being seen in the clinic for more than 3 months (91 days).

Analyses of causes of death, and all time-to-event outcomes which did not include all-cause mortality, used competing risks methods. These estimated the probability of the event (analogous to Kaplan-Meier) using cumulative incidence functions, and estimated the effect of randomized group on the subdistribution hazard corresponding to the cumulative incidence function. These analyses were conducted unstratified, as stratification is not possible with the estimating equation approach used for estimation. All endpoints based on new disease occurrences (e.g. time to new tuberculosis disease) included events identified as cause of death by the ERC if these had not already been reported as new disease events before death.

CD4, CD8, weight and BMI were compared between randomized groups over time using generalised estimating equations (GEE) (independent correlation structure) with randomized group, adjusting for stratification factors and scheduled visit week as categorical independent variables. The closest measurement to each scheduled visit date within equally spaced windows was used as the measurement at each scheduled visit. Continuous measurements were modelled using change from baseline as the outcome in a normal GEE model. Adherence measures and VL suppression were modelled as dichotomous outcomes in a binomial GEE model. Baseline values were those nearest to but before and within 42 days of randomization. Change in log10 VL from baseline to week 4 was modelled using normal interval regression to allow for censoring by the lower limit of quantification of the assay.

Drug susceptibility prediction used the Stanford algorithm (version 8.2). Major NRTI, NNRTI and INSTI mutations were defined using IAS-USA 2015.

For each of the primary and secondary outcomes, interactions with the other two factorial randomisations were tested by including interaction terms in the final model as above (ie rather than estimating a single effect of raltegravir-intensification vs standard-ART, estimating the effect of raltegravir-intensification vs standard-ART separately in each of the control and intervention groups of the other factorial randomisation).

**(h) Death rate**

To estimate a continuously varying death rate (hazard) we used flexible parametric models based on the standard Weibull model. The underlying model has monotonic (ie always increasing or always decreasing) hazard, but the flexible parametric models introduce additional terms in the hazard linearisation (via natural cubic splines) which allow the death rate to increase and then decrease or vice versa. The Akaike Information Criterion (AIC) was used to identify the number of interior knots for the natural cubic splines (between 1 and 5). AIC-based selection of the underlying model was performed adjusted for randomized group as an explanatory factor, then also allowing the variation in death rates over time to differ according to group (AIC used to identify number of interior knots for natural cubic splines for any departures from the baseline hazard (between 1 and number of interior knots identified for baseline hazard)). This analysis was not stratified for randomization stratification factors. The best fitting model according to AIC was with 2 interior knots at the 33rd and 67th percentiles of the uncensored survival times, plus 2 boundary knots at their minimum and maximum, and a non-proportional effect of randomized group on the hazard (linear effect of log time).

## (i) Subgroup analyses

We pre-specified in the protocol subgroup analyses by the other factorial randomizations and stratification factors (age </≥13 years, centre), and also by country, baseline CD4 (0-24, 25-49, 50-99 cells/mm^3^), initial backbone NRTI, initial NNRTI, Tuberculin Skin Test (TST) status (positive vs negative), and BMI<20 (adults) or weight-for-age<-1 (children). TST was not performed and therefore this subgroup analysis was not done, leaving 9 protocol-specified subgroup analyses.

We also conducted 5 additional exploratory subgroup analyses by sex, age group (5-17, 18-29, 30-39, 40+ years), VL (<100,000, 100,000-999,999, ≥1,000,000 copies/ml), WHO stage, and backbone NRTI and NNRTI frequency (twice-daily for zidovudine and nevirapine; once-daily for all other ART drugs), giving 14 subgroup analyses in total.

Subgroup analyses (all exploratory) were also carried out to explore potential heterogeneity of VL suppression <50 copies/ml at week-4 according to factors above.

For both mortality and VL suppression, for baseline CD4 and log_10_(VL) we used both categorisation and natural cubic splines (five knots at the 10^th^, 25^th^, 50^th^, 75^th^, and 90^th^ centiles) to test for interactions with the main intervention effect of raltegravir-intensification vs standard-ART. All subgroup analyses were conducted unstratified to avoid losing information from small strata with no events in one randomized group.

## (j) Baseline predictors of IRIS

Baseline predictors of IRIS were identified using competing risks methods based on backwards elimination with exit p=0.05 including non-linearity by fractional polynomials where p<0.05 (Stata mfp), forcing randomized ART and prophylaxis groups into the model. Factors considered were centre, sex, age, CD4 count, current steroid treatment, current cryptococcus, current TB disease and VL.
