# Supplementary material for: Raltegravir-intensified initial antiretroviral therapy in advanced HIV disease in Africa: A randomised controlled trial
Source: PLoS Med. 2018 Dec 4;15(12):e1002706. doi: 10.1371/journal.pmed.1002706 (PMC6279020; doi:10.1371/journal.pmed.1002706)
Supplement: S2 Text — (DOC) [file pmed.1002706.s004.doc]

# Supplementary Results

## (a) ART received

Participants initiated ART a median 5 (IQR 2-8) days after screening, predominantly with first-line tenofovir+emtricitabine+efavirenz (1384, 76.7%). 323 (17.9%) participants initiated ART with zidovudine+lamivudine as their NRTIs. Only 11 (0.6%) initiated ART >1 day after randomization (mostly due to tuberculosis co-treatment).

## (b) Self-reported adherence according to frequency of dosing of 2NRTI+NNRTI

Self-reported adherence was poorer overall in participants receiving NRTIs or NNRTI twice-daily (zidovudine or nevirapine) compared with those receiving them once-daily (p=0.0003) (**Figure S2b**). However, in those receiving twice-daily 2NRTI+NNRTI, self-reported ART adherence to 12 weeks was similar in raltegravir-intensified-ART vs standard-ART groups (p=0.40), whereas those on once-daily NRTI and NNRTI regimens had significantly poorer self-reported adherence if they also took (twice-daily) raltegravir (p<0.0001, p_heterogeneity_=0.0005).

## (c) Genotypic resistance

Of 789 raltegravir-intensified-ART participants with VL assayed at 12-weeks, 51 (6.5%) were >1000 copies/ml. Integrase genotypes were obtained in 33 (73.3%) of 45 from Kenya, Uganda and Zimbabwe (median VL 47,724 copies/ml). The primary/major accessory mutations T97A, R263K, L74M, Y143R/C or N155H were found in seven, five, one, one and one participants respectively, translating into one participant with predicted intermediate-level (T97A+R263K) and two with predicted high-level (N155H, Y143R/C) raltegravir resistance (9% of those sequenced at week-12; 0.6% of those randomized accounting for missing genotypes using probability weights). Week-48 VLs were 22976, 212418 and <50 c/ml respectively in these three patients. No patient had predicted intermediate or high-level dolutegravir resistance.

Of those with VL assayed at 48-weeks, 93/757 (12.3%) raltegravir-intensified-ART vs 96/746 (12.9%) standard-ART were >1000 copies/ml (p=0.73). Of those from Kenya, Uganda and Zimbabwe, reverse-transcriptase genotypes were available for 75/76 (98.7%) vs 87/90 (96.7%) respectively with median VL 89,815 copies/ml. The NRTI mutation K219E/Q (p=0.004), and the NNRTI mutations K101E/P (p=0.03) and P225H (p=0.007), were less common in the raltegravir-intensified-ART group, with no evidence of difference for other mutations (p>0.1, **Figure S8**). There was no evidence of differences between raltegravir-intensified-ART vs standard-ART in predicted intermediate/high-level resistance to lamivudine (overall 72.8% (n=118), p=0.20), zidovudine (12.3% (n=20), p=0.90), efavirenz/nevirapine (87.0% (n=141), p=0.28), or etravirine (38.9% (n=63), p=0.18) (**Figure S9**), whereas there was weak evidence of less predicted intermediate or high-level resistance in the raltegravir-intensified-ART group to tenofovir (24.0% (18/75) vs 37.9% (33/87) standard-ART, p=0.06), abacavir (40.0% (30/75) vs 54.0% (47/87) respectively, p=0.08) and rilpivirine (38.7% (29/75) vs 52.9% (46/87) respectively, p=0.07).

## (d) CD4 and CD8 count and body composition

Absolute CD4 count increases were similar in both groups through 24-weeks (p=0.76; **Figure 2b**); however, there was weak evidence of a small difference at 48-weeks (+161 (standard deviation ±4.4) cells/mm^3^ raltegravir-intensified-ART vs +148 (±4.4) cells/mm^3^ standard-ART, adjusted difference +11.4 (95% CI –0.4 to +23.1) p=0.06; no evidence of variation between children/adolescents and adults, p_heterogeneity_=0.25, **Figure S13a**). Changes in weight were also similar through week-24 in both groups (p=0.52; **Figure 2c**), with small but significant differences appearing from 36-48 weeks (at 48 weeks +6.4 (±0.2) kg standard-ART vs +7.2 (±0.2) kg raltegravir-intensified-ART, adjusted difference +0.8 (+0.1,+1.4) p=0.02; no evidence of variation between children/adolescents and adults p_heterogeneity_=0.37, **Figure S13b**). Similar late differences were observed for BMI in adolescents/adults (data not shown), fat mass (**Figure S10a/b**) and muscle mass (**Figure S10c/d**). Absolute CD8 count increases were similar in both groups through 48 weeks (p=0.82, **Figure S11**). Week 0–48 changes in CD4 count, weight, fat mass and muscle mass were moderately correlated (**Table S5**).

## (e) Additional safety outcomes

Liver function tests were measured at baseline and week-4. There was no evidence of between-group differences in changes in bilirubin (p=0.12) or ALT (p=0.55) which were measured in all participants. In the five centres where AST was measured routinely, there was weak evidence towards greater increases in AST at week-4 in the raltegravir-intensified-ART group (+9.7 (±3.25) IU/L raltegravir-intensified-ART vs +2.7 (±1.98) IU/L standard-ART, adjusted difference +6.8 (95% CI –0.6 to +14.3) p=0.07).
