# Supplementary material for: Raltegravir-intensified initial antiretroviral therapy in advanced HIV disease in Africa: A randomised controlled trial
Source: PLoS Med. 2018 Dec 4;15(12):e1002706. doi: 10.1371/journal.pmed.1002706 (PMC6279020; doi:10.1371/journal.pmed.1002706)
Supplement: S1 Table — SAE, serious adverse event. (DOC) [file pmed.1002706.s005.doc]

# Table S1 Serious adverse events

|  | **Standard  N=903** | **Raltegravir-intensified N=902** | **Total**  **N=1805** | **p** |
| --- | --- | --- | --- | --- |
| **Body system** |  |  |  |  |
| **Any** | **207 (22.9%) 287** | **203 (22.5%) 251** | **410 (22.7%) 538** | **0.87** |
| **CNS** | **24 (2.7%) 26** | **25 (2.8%) 25** | **49 (2.7%) 51** | **0.89** |
| Acute altered conscious level | 0 | 1 | 1 |  |
| Acute focal neurological event without fever, Cryptococcal disease serum CRAG +ve only | 0 | 1 | 1 |  |
| Disorientated/confusion | 2 | 1 | 3 |  |
| Dizziness | 1 | 0 | 1 |  |
| Encephalitis – presumed infectious | 0 | 2 | 2 |  |
| Encephalopathy – unspecified | 1 | 0 | 1 |  |
| Encephalopathy – unspecified, Stroke, cerebrovascular accident | 1 | 0 | 1 |  |
| Epilepsy, fits, convulsions | 0 | 1 | 1 |  |
| Headache | 1 | 1 | 2 |  |
| Hemiparesis | 2 | 0 | 2 |  |
| Inter-cranial pressure | 0 | 1 | 1 |  |
| Meningitis – other | 0 | 1 | 1 |  |
| Meningitis lumber puncture diagnosed – no organism (no culture), HIV associated nephropathy, Anaemia with no clinical symptoms | 1 | 0 | 1 |  |
| Meningitis lumber puncture diagnosed – no organism (no culture), Pneumonia no organism identified, Candidiasis of oesophagus, trachea, bronchi or lungs, Neutropenia | 0 | 1 | 1 |  |
| Meningitis no lumber puncture | 1 | 0 | 1 |  |
| Myelopathy | 0 | 1 | 1 |  |
| PML | 0 | 2 | 2 |  |
| Peripheral neuropathy - motor only, Metabolic disorder - other | 1 | 0 | 1 |  |
| Peripheral neuropathy - sensory & motor | 0 | 1 | 1 |  |
| Pyogenic meningitis - no organism | 2 | 0 | 2 |  |
| Pyogenic meningitis - organism | 0 | 1 | 1 |  |
| Stroke, cerebrovascular accident | 4 | 1 | 5 |  |
| Stroke, cerebrovascular accident, Cardiomyopathy, Tuberculosis - disseminated/miliary | 1 | 0 | 1 |  |
| Stroke, cerebrovascular accident, Salmonella bacteraemia - NON typhi | 0 | 1 | 1 |  |
| Toxoplasmosis of the brain | 1 | 1 | 2 |  |
| Other CNS disease | 1 | 1 | 2 |  |
| Brain syndrome/indeterminate intracerebral lesions | 4 | 4 | 8 |  |
| Brain syndrome/indeterminate intracerebral lesions, Anaemia with clinical symptoms | 1 | 0 | 1 |  |
| Brain syndrome/indeterminate intracerebral lesions, Anaemia with clinical symptoms, Hypoglycaemia, Raised creatinine | 0 | 1 | 1 |  |
| Brain syndrome/indeterminate intracerebral lesions, Presumed septicaemia/bacteraemia - not investigated, Lung syndrome, Renal failure - acute, Anaemia with clinical symptoms | 0 | 1 | 1 |  |
| Brain syndrome/indeterminate intracerebral lesions, Tuberculosis - pulmonary - smear positive | 1 | 0 | 1 |  |
| **Psychiatric** | **11 (1.2%) 11** | **1 (0.1%) 1** | **12 (0.7%) 12** | **0.006** |
| Depression | 1 | 0 | 1 |  |
| Psychosis, mania | 8 | 1 | 9 |  |
| Psychosis, mania, Neutropenia | 1 | 0 | 1 |  |
| Psychosis, mania, Tuberculosis - pulmonary - smear positive, Pneumonia no organism identified | 1 | 0 | 1 |  |
| **Lower Respiratory Tract** | **14 (1.6%) 15** | **15 (1.7%) 18** | **29 (1.6%) 33** | **0.85** |
| Candidiasis of oesophagus, trachea, bronchi or lungs | 1 | 1 | 2 |  |
| Chest infection | 0 | 2 | 2 |  |
| Pleural effusion - other, Pneumonia no organism identified | 0 | 1 | 1 |  |
| Pneumonia - Pneumocystis carinii (PCP) | 1 | 0 | 1 |  |
| Pneumonia - Pneumocystis carinii (PCP), Anaemia with no clinical symptoms | 1 | 0 | 1 |  |
| Pneumonia - other bacterial | 1 | 0 | 1 |  |
| Pneumonia - other bacterial, Anaemia with clinical symptoms | 1 | 0 | 1 |  |
| Pneumonia no organism identified | 6 | 11 | 17 |  |
| Pneumonia no organism identified, Ascites, Hepatitis B | 0 | 1 | 1 |  |
| Pneumonia no organism identified, Hypotension/shock/toxic shock | 0 | 1 | 1 |  |
| Pulmonary embolism, Deep vein thrombosis | 2 | 1 | 3 |  |
| Lung syndrome, Anaemia with clinical symptoms | 2 | 0 | 2 |  |
| **Cardiovascular** | **8 (0.9%) 10** | **5 (0.6%) 6** | **13 (0.7%) 16** | **0.58** |
| Chest pain | 1 | 0 | 1 |  |
| Cardiomyopathy, Tuberculosis - disseminated/miliary | 1 | 0 | 1 |  |
| Congestive cardiac failure, Cardiomyopathy, Anaemia with clinical symptoms | 1 | 0 | 1 |  |
| Congestive cardiac failure, HIV Associated Cardiomyopathy , Other cardiovascular, Deep vein thrombosis | 0 | 1 | 1 |  |
| Congestive cardiac failure, Pancytopenia, bone marrow depression | 1 | 0 | 1 |  |
| Deep vein thrombosis | 4 | 2 | 6 |  |
| Deep vein thrombosis, Anaemia with clinical symptoms | 0 | 2 | 2 |  |
| Deep vein thrombosis, Candidiasis of oesophagus, trachea, bronchi or lungs, Gastroenteritis | 1 | 0 | 1 |  |
| Deep vein thrombosis, Lymphadenopathy | 0 | 1 | 1 |  |
| Deep vein thrombosis, Pleural effusion - Tuberculosis | 1 | 0 | 1 |  |
| **Gastrointestinal** | **19 (2.1%) 21** | **13 (1.4%) 14** | **32 (1.8%) 35** | **0.37** |
| Abdominal or epigastric pain | 0 | 1 | 1 |  |
| Acute abdomen, Tuberculosis - abdominal | 1 | 0 | 1 |  |
| Appendicitis | 1 | 0 | 1 |  |
| Dysphagia, difficulty swallowing | 1 | 1 | 2 |  |
| Gastroenteritis | 8 | 4 | 12 |  |
| Gastroenteritis, Hepatitis cause unknown | 0 | 1 | 1 |  |
| Gastroenteritis, Raised creatinine | 1 | 0 | 1 |  |
| Gastroenteritis, Renal failure - acute | 0 | 1 | 1 |  |
| Haematemesis | 0 | 1 | 1 |  |
| Indigestion, oesophageal reflux, gastritis, ulcerative oesophagitis | 3 | 1 | 4 |  |
| Pancreatitis | 2 | 0 | 2 |  |
| Per rectal bleeding (fresh blood and/or malaena) | 1 | 0 | 1 |  |
| Vomiting | 2 | 4 | 6 |  |
| Vomiting, Abdominal or epigastric pain | 1 | 0 | 1 |  |
| **Diarrhoeal** | **3 (0.3%) 3** | **4 (0.4%) 4** | **7 (0.4%) 7** | **0.73** |
| Acute diarrhoea not investigated | 2 | 1 | 3 |  |
| Chronic diarrhoea not investigated, Oral candida | 0 | 1 | 1 |  |
| Chronic diarrhoea not investigated, Renal failure - acute | 0 | 1 | 1 |  |
| Chronic diarrhoea not investigated, Septicaemia with organism (unspecified), Pancytopenia, bone marrow depression | 1 | 0 | 1 |  |
| Chronic diarrhoea with cryptosporidia | 0 | 1 | 1 |  |
| **Wasting Syndrome** | **1 (0.1%) 1** | **1 (0.1%) 1** | **2 (0.1%) 2** | **1.00** |
| Severe weight loss (>10%) | 0 | 1 | 1 |  |
| Severe weight loss (>10%), Anaemia with clinical symptoms, Raised creatinine | 1 | 0 | 1 |  |
| **Hepatic** | **5 (0.6%) 6** | **8 (0.9%) 8** | **13 (0.7%) 14** | **0.42** |
| Acute hepatitis | 3 | 4 | 7 |  |
| Acute hepatitis, Candidiasis of oesophagus, trachea, bronchi or lungs, Anaemia with clinical symptoms | 0 | 1 | 1 |  |
| Hepatic encephalopathy, Hepatic failure - chronic, Tuberculosis - disseminated/miliary, Hepatitis B | 0 | 1 | 1 |  |
| Hepatic failure - acute | 0 | 1 | 1 |  |
| Hepatic failure - acute, Tuberculosis - pulmonary - smear negative or not done, Wasting syndrome uninvestigated | 2 | 0 | 2 |  |
| Hepatic failure - acute, Tuberculosis - pulmonary - smear positive, Renal failure - acute, Hepatitis B, Neutropenia | 0 | 1 | 1 |  |
| Jaundice | 1 | 0 | 1 |  |
| **Renal** | **10 (1.1%) 10** | **10 (1.1%) 11** | **20 (1.1%) 21** | **1.00** |
| HIV associated nephropathy | 1 | 0 | 1 |  |
| HIV associated nephropathy, Tuberculosis - pulmonary - smear positive | 0 | 1 | 1 |  |
| Haematuria, Anaemia with clinical symptoms | 0 | 1 | 1 |  |
| Pyelonephritis | 1 | 2 | 3 |  |
| Pyelonephritis, Renal failure - acute | 0 | 1 | 1 |  |
| Renal failure - acute | 2 | 2 | 4 |  |
| Renal failure - acute, Acute diarrhoea not investigated, Intravascular haemolysis | 1 | 0 | 1 |  |
| Renal failure - acute, Acute diarrhoea not investigated, Oral candida | 0 | 2 | 2 |  |
| Renal failure - acute, Anaemia with clinical symptoms | 1 | 0 | 1 |  |
| Renal failure - acute, Anaemia with clinical symptoms, Tuberculosis - lymph nodes, Kaposi's sarcoma lymph nodes, Overdose (not suicide attempt), Peripheral neuropathy - sensory & motor | 1 | 0 | 1 |  |
| Renal failure - acute, Gastroenteritis | 0 | 1 | 1 |  |
| Renal failure - acute, Gastroenteritis, Anaemia with clinical symptoms | 1 | 0 | 1 |  |
| Renal failure - acute, Pyelonephritis | 0 | 1 | 1 |  |
| Renal failure - acute, Raised AST, Raised ALT | 1 | 0 | 1 |  |
| Renal failure - acute, Tuberculosis - other, Oral candida | 1 | 0 | 1 |  |
| **Genitourinary** | **2 (0.2%) 2** | **1 (0.1%) 1** | **3 (0.2%) 3** | **1.00** |
| Lower urinary tract infection (UTI), cystitis, Neutropenia | 1 | 0 | 1 |  |
| Lower urinary tract infection (UTI), cystitis, Raised creatinine, Hypertension | 0 | 1 | 1 |  |
| Vaginal bleeding | 1 | 0 | 1 |  |
| **Musculoskeletal** | **0 (0.0%) 0** | **1 (0.1%) 1** | **1 (0.1%) 1** | **0.50** |
| Pyomyositis - infection | 0 | 1 | 1 |  |
| **Skin** | **1 (0.1%) 1** | **2 (0.2%) 2** | **3 (0.2%) 3** | **0.62** |
| Hypersensitivity reaction | 0 | 2 | 2 |  |
| Hypersensitivity reaction, Epilepsy, fits, convulsions | 1 | 0 | 1 |  |
| **Haematological** | **23 (2.5%) 24** | **9 (1.0%) 11** | **32 (1.8%) 35** | **0.02** |
| Anaemia with clinical symptoms | 16 | 7 | 23 |  |
| Anaemia with clinical symptoms, Gastroenteritis | 1 | 0 | 1 |  |
| Anaemia with clinical symptoms, Gastroenteritis, Neutropenia | 1 | 0 | 1 |  |
| Anaemia with clinical symptoms, Headache | 0 | 1 | 1 |  |
| Anaemia with clinical symptoms, Hemiparesis | 0 | 1 | 1 |  |
| Anaemia with clinical symptoms, Neutropenia | 0 | 2 | 2 |  |
| Anaemia with clinical symptoms, Neutropenia, Tuberculosis - pulmonary - smear positive | 1 | 0 | 1 |  |
| Anaemia with clinical symptoms, Renal failure - acute | 1 | 0 | 1 |  |
| Anaemia with clinical symptoms, Skin abscess, Neutropenia | 1 | 0 | 1 |  |
| Anaemia with clinical symptoms, Thrombocytopenia | 1 | 0 | 1 |  |
| Anaemia with no clinical symptoms, Neutropenia, Thrombocytopenia | 1 | 0 | 1 |  |
| Pancytopenia, bone marrow depression | 1 | 0 | 1 |  |
| **Biochemical** | **0 (0.0%) 0** | **2 (0.2%) 2** | **2 (0.1%) 2** | **0.25** |
| Metabolic disorder - other, Candidiasis of oesophagus, trachea, bronchi or lungs | 0 | 1 | 1 |  |
| Raised bilirubin, Raised liver enzymes | 0 | 1 | 1 |  |
| **Systemic** | **5 (0.6%) 5** | **8 (0.9%) 9** | **13 (0.7%) 14** | **0.42** |
| Wasting syndrome uninvestigated | 1 | 4 | 5 |  |
| Wasting syndrome uninvestigated, Abdominal or epigastric pain | 0 | 1 | 1 |  |
| Wasting syndrome uninvestigated, Acute hepatitis | 0 | 1 | 1 |  |
| Wasting syndrome uninvestigated, Gastroenteritis | 0 | 1 | 1 |  |
| Wasting syndrome uninvestigated, Oral candida | 1 | 0 | 1 |  |
| Stevens-Johnson Syndrome | 0 | 2 | 2 |  |
| Stevens-Johnson Syndrome, Presumed septicaemia/bacteraemia - not investigated | 1 | 0 | 1 |  |
| Dehydration, Tuberculosis - abdominal | 1 | 0 | 1 |  |
| Dehydration, Vomiting | 1 | 0 | 1 |  |
| **Specific Infections** | **92 (10.2%) 111** | **85 (9.4%) 94** | **177 (9.8%) 205** | **0.63** |
| Cryptococcal meningitis | 17 | 14 | 31 |  |
| Cryptococcal meningitis, Anaemia with no clinical symptoms | 1 | 0 | 1 |  |
| Cryptococcal meningitis, Cirrhosis, Candidiasis of oesophagus, trachea, bronchi or lungs | 0 | 1 | 1 |  |
| Cryptococcal meningitis, Raised ALT | 1 | 0 | 1 |  |
| Cryptococcal meningitis, Renal failure - acute | 1 | 0 | 1 |  |
| Cryptococcal meningitis, Renal failure - acute, Anaemia with clinical symptoms | 0 | 1 | 1 |  |
| Cryptococcal meningitis, Tuberculosis - disseminated/miliary | 0 | 1 | 1 |  |
| Tuberculosis - meningitis | 0 | 2 | 2 |  |
| Tuberculosis - meningitis, Candidiasis of oesophagus, trachea, bronchi or lungs | 1 | 0 | 1 |  |
| Tuberculosis - meningitis, Pneumonia no organism identified, Oral candida | 1 | 0 | 1 |  |
| Pleural effusion - Tuberculosis | 1 | 0 | 1 |  |
| Pleural effusion - Tuberculosis, P falciparum malaria | 0 | 1 | 1 |  |
| Tuberculosis - pulmonary - smear negative or not done | 1 | 5 | 6 |  |
| Tuberculosis - pulmonary - smear negative or not done, Aspiration pneumonia | 1 | 0 | 1 |  |
| Tuberculosis - pulmonary - smear negative or not done, Hepatitis cause unknown | 1 | 0 | 1 |  |
| Tuberculosis - pulmonary - smear negative or not done, Neutropenia | 0 | 1 | 1 |  |
| Tuberculosis - pulmonary - smear negative or not done, Pneumonia - other bacterial | 1 | 0 | 1 |  |
| Tuberculosis - pulmonary - smear negative or not done, Severe malnutrition, Pneumonia - other bacterial, Anaemia with clinical symptoms | 1 | 0 | 1 |  |
| Tuberculosis - pulmonary - smear positive | 11 | 3 | 14 |  |
| Tuberculosis - pulmonary - smear positive, Anaemia with clinical symptoms | 1 | 1 | 2 |  |
| Tuberculosis - pulmonary - smear positive, Oral candida | 0 | 2 | 2 |  |
| Tuberculosis - pulmonary - smear positive, Pneumonia no organism identified | 0 | 1 | 1 |  |
| Tuberculosis - pulmonary - smear positive, Renal failure - acute, Anaemia with clinical symptoms, Hyponatraemia | 0 | 1 | 1 |  |
| Tuberculosis - pulmonary - smear positive, Tuberculosis - lymph nodes | 1 | 0 | 1 |  |
| CMV retinitis | 1 | 1 | 2 |  |
| Tuberculosis - abdominal | 4 | 2 | 6 |  |
| Tuberculosis - abdominal, Anaemia with no clinical symptoms, Acute hepatitis | 0 | 1 | 1 |  |
| Tuberculosis - abdominal, Peripheral neuropathy - sensory & motor, Ulcer, decubitus ulcer | 1 | 0 | 1 |  |
| Cutaneous warts, Human Papillomavirus | 1 | 0 | 1 |  |
| Tuberculosis - lymph nodes | 2 | 4 | 6 |  |
| Tuberculosis - lymph nodes, Acute hepatitis, Rash, urticaria | 1 | 0 | 1 |  |
| Tuberculosis - lymph nodes, Anaemia with clinical symptoms | 1 | 0 | 1 |  |
| Tuberculosis - lymph nodes, Jaundice | 1 | 0 | 1 |  |
| Tuberculosis - lymph nodes, Renal failure - acute | 1 | 0 | 1 |  |
| Mycobacterial disease - atypical disseminated | 1 | 0 | 1 |  |
| Tuberculosis - disseminated/miliary | 17 | 11 | 28 |  |
| Tuberculosis - disseminated/miliary, Acute hepatitis | 2 | 0 | 2 |  |
| Tuberculosis - disseminated/miliary, Anaemia with clinical symptoms | 2 | 1 | 3 |  |
| Tuberculosis - disseminated/miliary, Anaemia with clinical symptoms, Presumed septicaemia/bacteraemia - no organism, Renal failure - acute | 1 | 0 | 1 |  |
| Tuberculosis - disseminated/miliary, Ascites, Pleural effusion - other, P falciparum malaria | 1 | 0 | 1 |  |
| Tuberculosis - disseminated/miliary, Candidiasis of oesophagus, trachea, bronchi or lungs | 1 | 0 | 1 |  |
| Tuberculosis - disseminated/miliary, Chronic diarrhoea not investigated, Acute hepatitis | 0 | 1 | 1 |  |
| Tuberculosis - disseminated/miliary, Hepatic failure - acute, Anaemia with clinical symptoms | 0 | 1 | 1 |  |
| Tuberculosis - disseminated/miliary, Hepatic failure - acute, Renal failure - acute, Candidiasis of oesophagus, trachea, bronchi or lungs | 1 | 0 | 1 |  |
| Tuberculosis - disseminated/miliary, Kaposi's sarcoma cutaneous | 0 | 1 | 1 |  |
| Tuberculosis - disseminated/miliary, Neutropenia | 1 | 0 | 1 |  |
| Tuberculosis - disseminated/miliary, Oral candida | 1 | 0 | 1 |  |
| Tuberculosis - disseminated/miliary, Pneumonia - other bacterial | 0 | 1 | 1 |  |
| Tuberculosis - disseminated/miliary, Pneumonia no organism identified | 0 | 1 | 1 |  |
| Tuberculosis - disseminated/miliary, Pneumonia no organism identified, Anaemia with no clinical symptoms, Visceral abscess | 1 | 0 | 1 |  |
| Tuberculosis - disseminated/miliary, Pneumonia no organism identified, Jaundice | 0 | 1 | 1 |  |
| Tuberculosis - disseminated/miliary, Presumed septicaemia/bacteraemia - no organism, Hypokalaemia, Purpura, bruising, petechiae, Thrombocytopenia, Low albumin | 1 | 0 | 1 |  |
| Tuberculosis - disseminated/miliary, Presumed septicaemia/bacteraemia - not investigated | 0 | 3 | 3 |  |
| Tuberculosis - disseminated/miliary, Presumed septicaemia/bacteraemia - not investigated, Anaemia with clinical symptoms | 0 | 1 | 1 |  |
| Tuberculosis - disseminated/miliary, Presumed septicaemia/bacteraemia - not investigated, Pancytopenia, bone marrow depression | 0 | 1 | 1 |  |
| Tuberculosis - disseminated/miliary, Pure red cell aplasia | 5 | 0 | 5 |  |
| Tuberculosis - disseminated/miliary, Raised liver enzymes | 0 | 3 | 3 |  |
| Tuberculosis - disseminated/miliary, Renal failure - acute | 0 | 2 | 2 |  |
| Tuberculosis - disseminated/miliary, Thrombocytopenia | 1 | 0 | 1 |  |
| Tuberculosis - other | 0 | 1 | 1 |  |
| Tuberculosis - other, Candidiasis of oesophagus, trachea, bronchi or lungs, Hypophosphataemia | 0 | 1 | 1 |  |
| Cryptococcal fungaemia | 1 | 0 | 1 |  |
| Presumed septicaemia/bacteraemia - no organism | 3 | 2 | 5 |  |
| Presumed septicaemia/bacteraemia - no organism, Anaemia with clinical symptoms | 0 | 1 | 1 |  |
| Presumed septicaemia/bacteraemia - no organism, Anaemia with clinical symptoms, P falciparum malaria | 1 | 0 | 1 |  |
| Presumed septicaemia/bacteraemia - no organism, Candidiasis of oesophagus, trachea, bronchi or lungs | 1 | 0 | 1 |  |
| Presumed septicaemia/bacteraemia - no organism, Disorientated/confusion, Hypoglycaemia | 0 | 1 | 1 |  |
| Presumed septicaemia/bacteraemia - no organism, Renal failure - acute, Pancytopenia, bone marrow depression | 0 | 1 | 1 |  |
| Presumed septicaemia/bacteraemia - no organism, Renal failure - chronic | 1 | 0 | 1 |  |
| Presumed septicaemia/bacteraemia - no organism, Visceral abscess, Candidiasis of oesophagus, trachea, bronchi or lungs | 0 | 1 | 1 |  |
| Presumed septicaemia/bacteraemia - not investigated | 4 | 2 | 6 |  |
| Presumed septicaemia/bacteraemia - not investigated, Anaemia with clinical symptoms | 1 | 1 | 2 |  |
| Presumed septicaemia/bacteraemia - not investigated, Anaemia with clinical symptoms, Neutropenia | 0 | 1 | 1 |  |
| Presumed septicaemia/bacteraemia - not investigated, Brain syndrome/indeterminate intracerebral lesions | 0 | 1 | 1 |  |
| Presumed septicaemia/bacteraemia - not investigated, Diabetes - Type II, Disorientated/confusion | 1 | 0 | 1 |  |
| Presumed septicaemia/bacteraemia - not investigated, Lower urinary tract infection (UTI), cystitis | 1 | 0 | 1 |  |
| Presumed septicaemia/bacteraemia - not investigated, Pancytopenia, bone marrow depression | 0 | 2 | 2 |  |
| Presumed septicaemia/bacteraemia - not investigated, Pneumonia no organism identified | 1 | 0 | 1 |  |
| Presumed septicaemia/bacteraemia - not investigated, Renal failure - acute, Anaemia with clinical symptoms, Thrombocytopenia | 0 | 1 | 1 |  |
| Presumed septicaemia/bacteraemia - not investigated, Tuberculosis - abdominal | 0 | 1 | 1 |  |
| Presumed septicaemia/bacteraemia - not investigated, Tuberculosis - disseminated/miliary | 1 | 0 | 1 |  |
| Presumed septicaemia/bacteraemia - not investigated, Tuberculosis - pulmonary - smear positive | 0 | 1 | 1 |  |
| Presumed septicaemia/bacteraemia - not investigated, Wasting syndrome with investigations | 1 | 0 | 1 |  |
| P falciparum malaria | 2 | 4 | 6 |  |
| P falciparum malaria, Anaemia with no clinical symptoms, Neutropenia | 1 | 0 | 1 |  |
| P falciparum malaria, Neutropenia | 1 | 1 | 2 |  |
| Histoplasmosis, Anaemia with clinical symptoms | 0 | 1 | 1 |  |
| **Tumours** | **10 (1.1%) 13** | **12 (1.3%) 12** | **22 (1.2%) 25** | **0.68** |
| Primary CNS lymphoma | 0 | 1 | 1 |  |
| Kaposi's sarcoma pulmonary | 0 | 1 | 1 |  |
| Kaposi's sarcoma mucosal | 0 | 1 | 1 |  |
| Kaposi's sarcoma cutaneous | 5 | 2 | 7 |  |
| Kaposi's sarcoma cutaneous, Anaemia with clinical symptoms | 2 | 0 | 2 |  |
| Kaposi's sarcoma lymph nodes | 2 | 0 | 2 |  |
| Kaposi's sarcoma lymph nodes, Nephrotic syndrome | 1 | 0 | 1 |  |
| Non Hodgkin lymphoma | 0 | 1 | 1 |  |
| Benign tumour | 0 | 1 | 1 |  |
| Other Solid tumour | 1 | 2 | 3 |  |
| Kaposi's sarcoma - other | 1 | 1 | 2 |  |
| Kaposi's sarcoma - other, Acute hepatitis | 0 | 1 | 1 |  |
| Kaposi's sarcoma - other, Anaemia with clinical symptoms | 1 | 0 | 1 |  |
| Kaposi's sarcoma - other, Presumed septicaemia/bacteraemia - not investigated | 0 | 1 | 1 |  |
| **Abscess** | **1 (0.1%) 1** | **2 (0.2%) 2** | **3 (0.2%) 3** | **0.62** |
| CNS abscess | 0 | 1 | 1 |  |
| CNS abscess, Pyogenic meningitis - no organism | 1 | 0 | 1 |  |
| Skin abscess | 0 | 1 | 1 |  |
| **Non HIV Related Deaths** | **1 (0.1%) 1** | **0 (0.0%) 0** | **1 (0.1%) 1** | **1.00** |
| Traumatic | 1 | 0 | 1 |  |
| **Other** | **26 (2.9%) 26** | **28 (3.1%) 28** | **54 (3.0%) 54** | **0.78** |
| Non-fatal trauma | 0 | 3 | 3 |  |
| Death, cause unknown | 26 | 25 | 51 |  |
| **Unknown** | **0 (0.0%) 0** | **1 (0.1%) 1** | **1 (0.1%) 1** |  |
| Unknown reason for hospitalization | 0 | 1 | 1 |  |

Note: Table shows number of patients with one or more episode (% of patients) [number of episodes] (e.g., '2 (20.0%) [3],' would indicate a total of 3 episodes in 2 patients).
