# Supplementary material for: Raltegravir-intensified initial antiretroviral therapy in advanced HIV disease in Africa: A randomised controlled trial
Source: PLoS Med. 2018 Dec 4;15(12):e1002706. doi: 10.1371/journal.pmed.1002706 (PMC6279020; doi:10.1371/journal.pmed.1002706)
Supplement: S2 Table — AE, adverse event. (DOC) [file pmed.1002706.s006.doc]

# Table S2 Adverse events (any grade) leading to modification of raltegravir

|  | **Raltegravir-intensified  N (%)** |
| --- | --- |
| **Modified raltegravir due to an adverse event** | **19 (2.1%)** |
| Renal failure - acute | 6 (0.7%) |
| Hypersensitivity reaction | 3 (0.3%) |
| Acute hepatitis | 2 (0.2%) |
| Dizziness | 1 (0.1%) |
| Enlarged liver, hepatomegaly | 1 (0.1%) |
| Hepatic failure - acute | 1 (0.1%) |
| Jaundice | 1 (0.1%) |
| Pulmonary embolism | 1 (0.1%) |
| Raised AST | 1 (0.1%) |
| Stevens-Johnson Syndrome | 1 (0.1%) |
| Tuberculosis - abdominal | 1 (0.1%) |
