# Supplementary material for: Raltegravir-intensified initial antiretroviral therapy in advanced HIV disease in Africa: A randomised controlled trial
Source: PLoS Med. 2018 Dec 4;15(12):e1002706. doi: 10.1371/journal.pmed.1002706 (PMC6279020; doi:10.1371/journal.pmed.1002706)
Supplement: S3 Table — IRIS, immune reconstitution inflammatory syndrome. (DOC) [file pmed.1002706.s007.doc]

# Table S3 IRIS events

|  | **Standard ART**  **N particpants (% of 903)** | **Raltegravir-intensified ART N participants (% of 902)** | **Total**  **N participants (% of 1805)** | **p** |
| --- | --- | --- | --- | --- |
| All IRIS events (fatal and non-fatal) | 86(9.5%) | 89 (9.9%) | 175 (9.7%) | 0.79 |
| TB-IRIS | 54(6.0%) | 53(5.9%) | 107 (5.9%) | 1.00 |
| Cryptococcal IRIS | 16(1.8%) | 15(1.7%) | 31 (1.7%) | 1.00 |
| Other IRIS events of known aetiology | 14(1.6%) | 17(1.9%) | 31 (1.7%) | 0.59 |
| Other opportunistic infection-IRIS | 4 | 3 | 7 |  |
| Toxoplasmosis IRIS | 1 | 1 | 2 |  |
| PJP(PCP)-IRIS | 2 | 0 | 2 |  |
| Cytomegalovirus | 1 | 2 | 3 |  |
| Kaposi’s sarcoma | 4 | 8 | 12 |  |
| Hepatitis IRIS +/- viral hepatitis | 3 | 1 | 4 |  |
| Central Nervous System-IRIS (unknown pathogen) | 1 | 3 | 4 |  |
| Lung IRIS (unknown pathogen) | 2 | 0 | 2 |  |
| Other | 0 | 3 | 3 |  |
| IRIS events of unknown aetiology | 2 (0.2%) | 4 (0.4%) | 6 (0.3%) |  |

Note: Compatibility of clinical events with IRIS was adjudicated by an Endpoint Review Committee (ERC) (majority independent members) blind to trial drugs received.
