# Supplementary material for: Raltegravir-intensified initial antiretroviral therapy in advanced HIV disease in Africa: A randomised controlled trial
Source: PLoS Med. 2018 Dec 4;15(12):e1002706. doi: 10.1371/journal.pmed.1002706 (PMC6279020; doi:10.1371/journal.pmed.1002706)
Supplement: S4 Table — IRIS, immune reconstitution inflammatory syndrome. (DOC) [file pmed.1002706.s008.doc]

# Table S4 Baseline predictors of IRIS

|  | **sHR [95% CI]** | **p** |
| --- | --- | --- |
| Raltegravir-intensified vs standard ART | 1.08 [0.80-1.45] | 0.63 |
| Enhanced-prophylaxis vs standard-prophylaxis | 0.60 [0.44-0.82] | 0.001 |
| CD4 count (per 10 cells/mm^3^ higher) | 0.87 [0.82-0.93] | <0.001 |
| Age at last birthday (per year older) |  | 0.03 |
| ≤29 years | 1.07 [1.02-1.22] | 0.008 |
| ≥30 years | 0.99 [0.97-1.01] | 0.44 |
| Current TB disease at ART initiation | 1.62 [1.11-2.37] | 0.01 |
| Note: sHR=subhazard ratio. Adjusted for other factors in column and centre (p=0.002) | | |
