# Supplementary material for: Raltegravir-intensified initial antiretroviral therapy in advanced HIV disease in Africa: A randomised controlled trial
Source: PLoS Med. 2018 Dec 4;15(12):e1002706. doi: 10.1371/journal.pmed.1002706 (PMC6279020; doi:10.1371/journal.pmed.1002706)
Supplement: S5 Table — CD4, cluster of differentiation 4. (DOC) [file pmed.1002706.s009.doc]

# Table S5 Pairwise Spearman correlations between week 0 to 48 changes in CD4, weight, fat mass and muscle mass

#

|  | **Change in CD4** | **Change in weight** | **Change in fat mass** | **Change in muscle mass** |
| --- | --- | --- | --- | --- |
| **Change in CD4** | - |  |  |  |
| **Change in weight** | +0.34 (n=1457) | - |  |  |
| **Change in fat mass** | +0.28 (n=1430) | +0.78 (n=1431) | - |  |
| **Change in muscle mass** | +0.21 (n=1431) | +0.65 (n=1432) | +0.25 (n=1431) | - |
