# Supplementary figures and images for: Raltegravir-intensified initial antiretroviral therapy in advanced HIV disease in Africa: A randomised controlled trial
Source: PLoS Med. 2018 Dec 4;15(12):e1002706. doi: 10.1371/journal.pmed.1002706 (PMC6279020; doi:10.1371/journal.pmed.1002706)

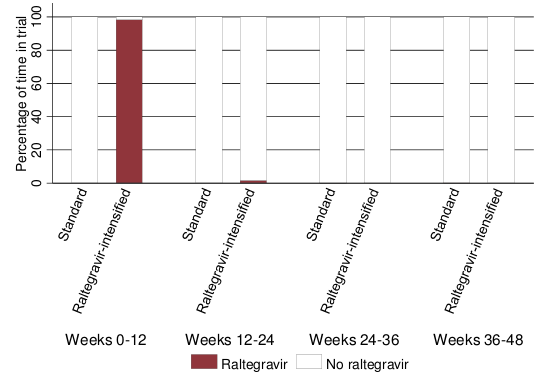

Supplement: S1 Fig — (TIF) [file pmed.1002706.s010.tif]

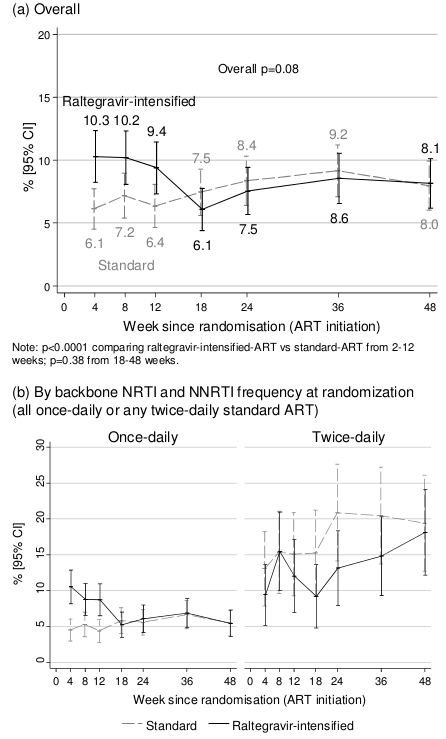

Supplement: S2 Fig — Self-reported percentage reporting missing doses of any ART in the last 4 weeks (a) overall and (b) by backbone NRTI and NNRTI frequency. ART, antiretroviral therapy; NNRTI, non-nucleoside reverse transcriptase inhibitor; NRTI, nucleoside reverse transcriptase inhibitor. (TIF) [file pmed.1002706.s011.tif]

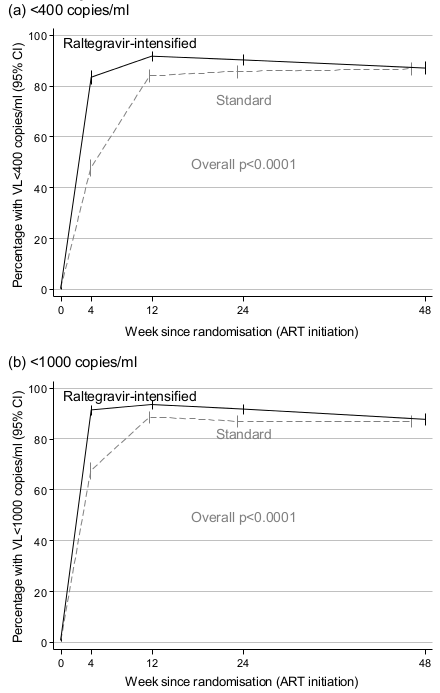

Supplement: S3 Fig — VL suppression (a) <400 copies/mL and (b) <1,000 copies/mL. VL, viral load. (TIF) [file pmed.1002706.s012.tif]

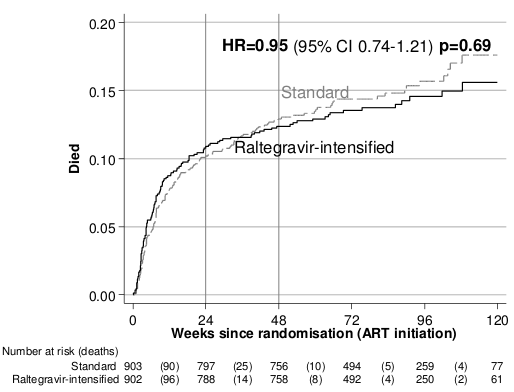

Supplement: S4 Fig — (TIF) [file pmed.1002706.s013.tif]

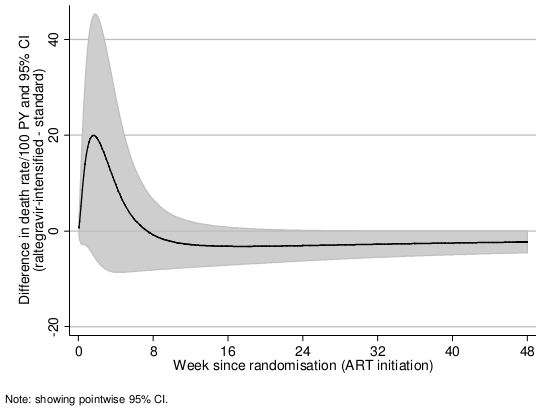

Supplement: S5 Fig — ART, antiretroviral therapy. (TIF) [file pmed.1002706.s014.tif]

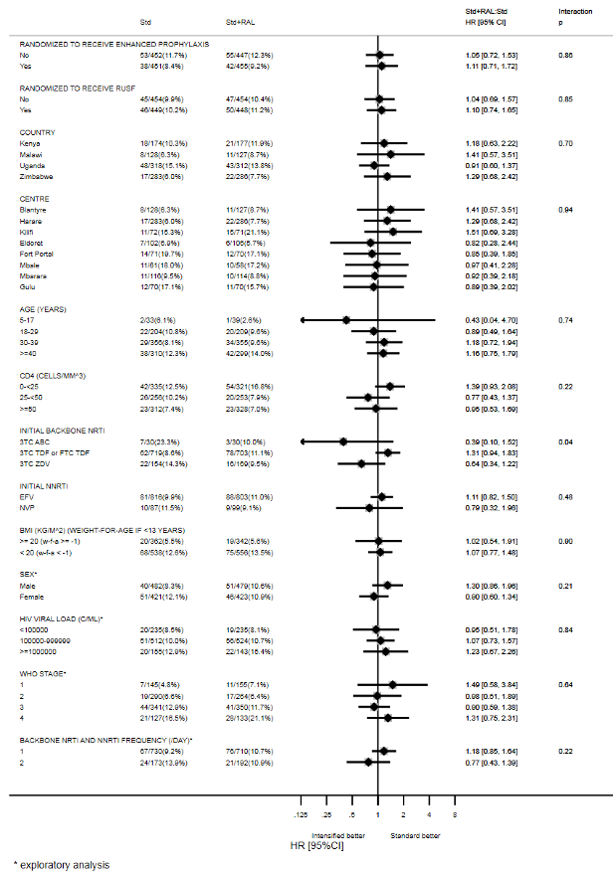

Supplement: S6 Fig — (TIF) [file pmed.1002706.s015.tif]

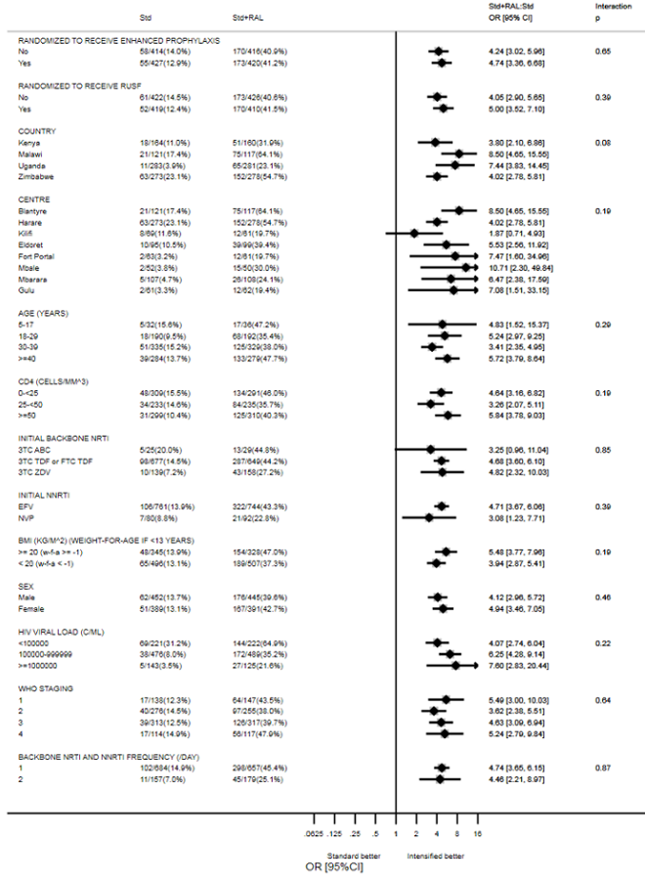

Supplement: S7 Fig — VL, viral load. (TIF) [file pmed.1002706.s016.tif]

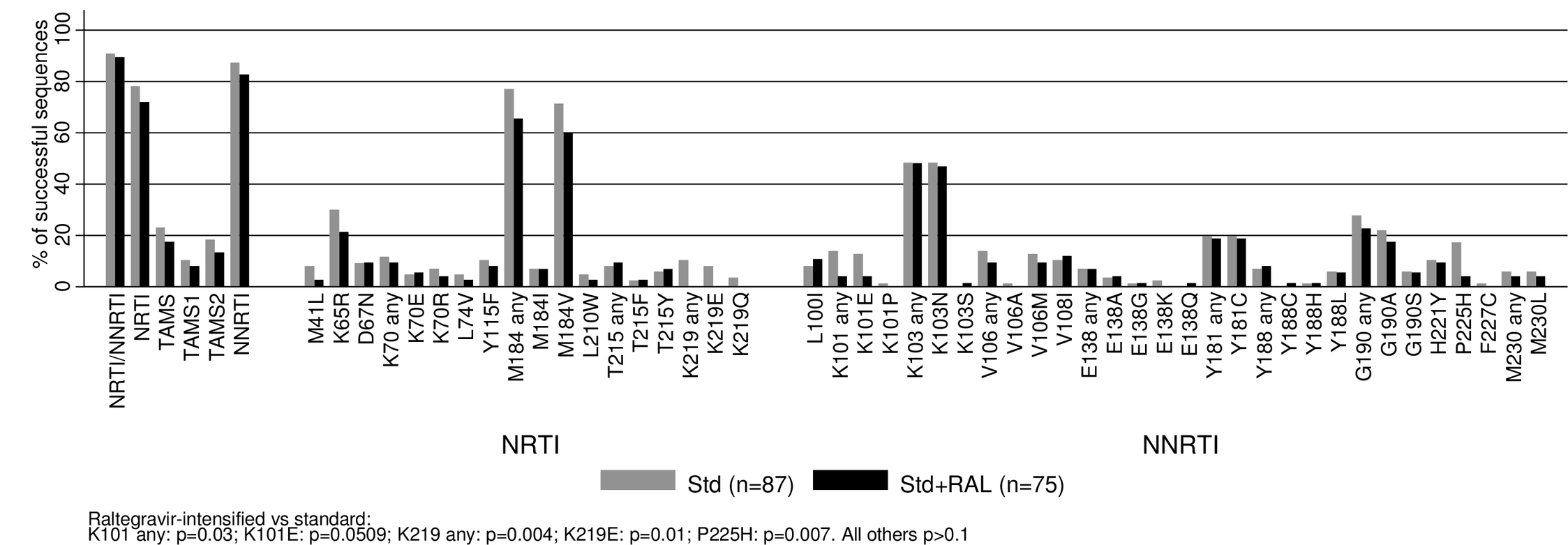

Supplement: S8 Fig — NNRTI, non-nucleoside reverse transcriptase inhibitor; NRTI, nucleoside reverse transcriptase inhibitor; VL, viral load. (TIF) [file pmed.1002706.s017.tif]

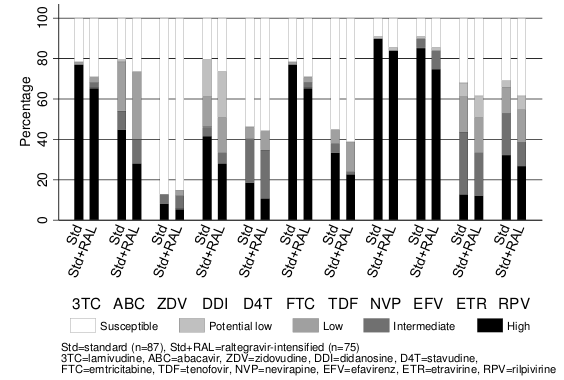

Supplement: S9 Fig — VL, viral load. (TIF) [file pmed.1002706.s018.tif]

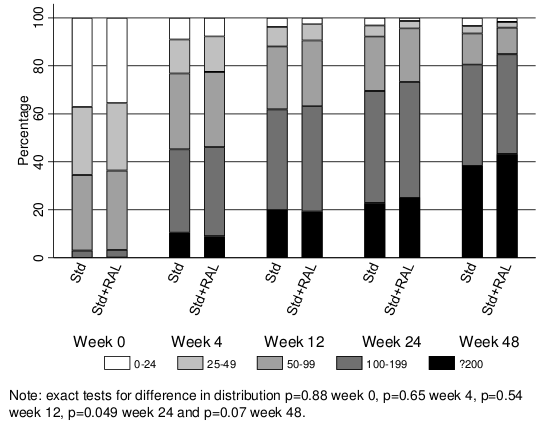

Supplement: S10 Fig — CD4, cluster of differentiation 4. (TIF) [file pmed.1002706.s019.tif]

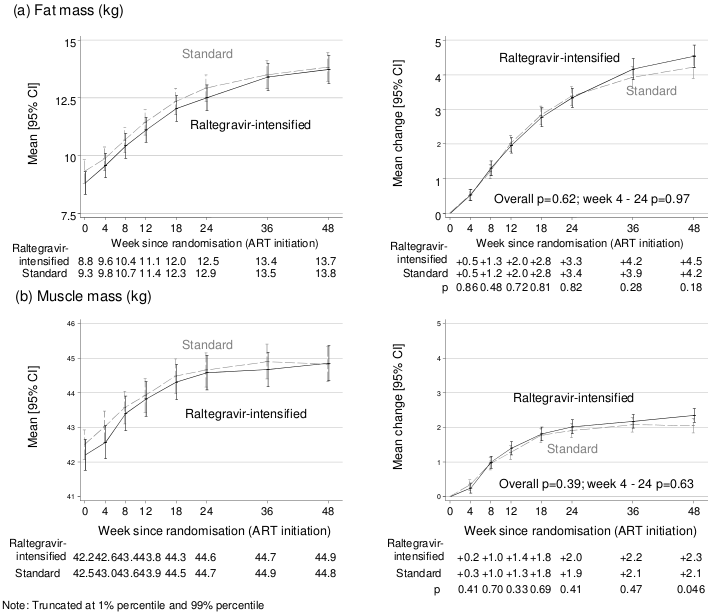

Supplement: S11 Fig — Changes in body composition, (a) fat mass and (b) muscle mass. (TIF) [file pmed.1002706.s020.tif]

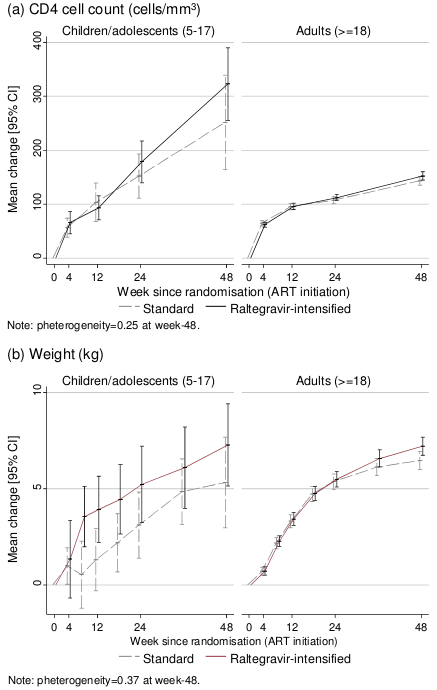

Supplement: S12 Fig — Changes in (a) CD4 cell count and (b) weight in children/adolescents (5–17 years) versus adults (18 years or older) at ART initiation. ART, antiretroviral therapy; CD4, cluster of differentiation 4. (TIF) [file pmed.1002706.s021.tif]

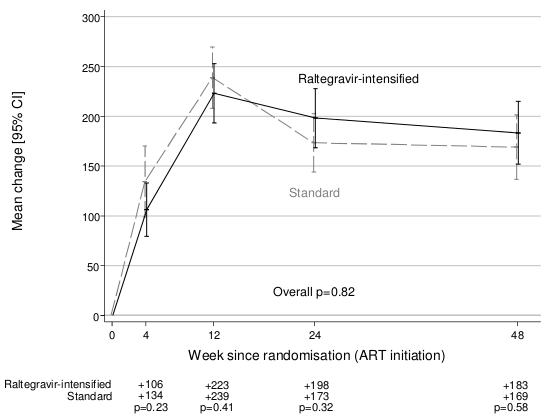

Supplement: S13 Fig — CD8, cluster of differentiation 8. (TIF) [file pmed.1002706.s022.tif]

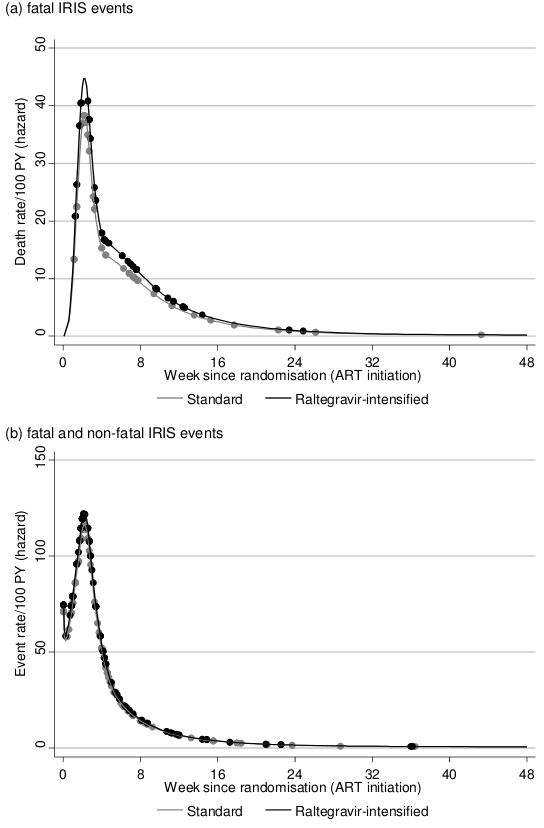

Supplement: S14 Fig — IRIS, immune reconstitution inflammatory syndrome. (TIF) [file pmed.1002706.s023.tif]
